# Catalytic Enantioselective Synthesis of 3-Piperidines from Arylboronic Acids and Pyridine

**DOI:** 10.1021/jacs.3c05044

**Published:** 2023-06-22

**Authors:** Sourabh Mishra, Sedef Karabiyikoglu, Stephen P. Fletcher

**Affiliations:** Chemistry Research Laboratory, Department of Chemistry, University of Oxford, Oxford OX1 3TA, United Kingdom

## Abstract

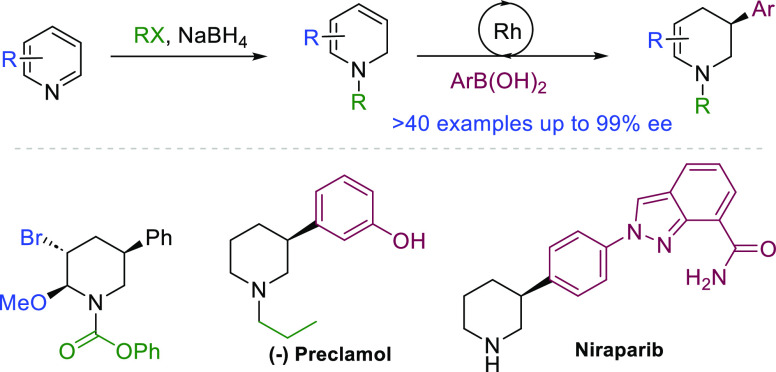

Piperidines are frequently found in natural products and are of importance to the pharmaceutical industry. A generally useful asymmetric route to enantiomerically enriched 3-substituted piperidines remains elusive. Here we report a cross-coupling approach to enantioenriched 3-piperidines from pyridine- and sp^2^-hybridized boronic acids. The key step involves a Rh-catalyzed asymmetric reductive Heck reaction of aryl, heteroaryl, or vinyl boronic acids and phenyl pyridine-1(2*H*)-carboxylate to provide 3-substituted tetrahydropyridines in high yield and excellent enantioselectivity with a wide functional group tolerance. A three-step process involving i) partial reduction of pyridine, ii) Rh-catalyzed asymmetric carbometalation, and then iii) another reduction provides access to a wide variety of enantioenriched 3-piperidines, including clinically used materials such as Preclamol and Niraparib.

Nitrogen-containing heterocycles are prevalent in natural products and drug candidates. Specifically, piperidines are frequently found in pharmaceutically relevant molecules.^1−3^ For instance, Niraparib is an anticancer drug,^4^ Preclamol and OSU-6162 are antipsychotic agents,^5,6^ Tiagbine is an anticonvulsant drug,^7^ Pergolide has been used to treat Parkinson’s disease,^6^ and CP-868388 is a potential PPAR agonist from Pfizer^8^ (Figure 1). A number of recent papers have also uncovered further important biological activities associated with piperidines.^9−12^

**Figure 1:**
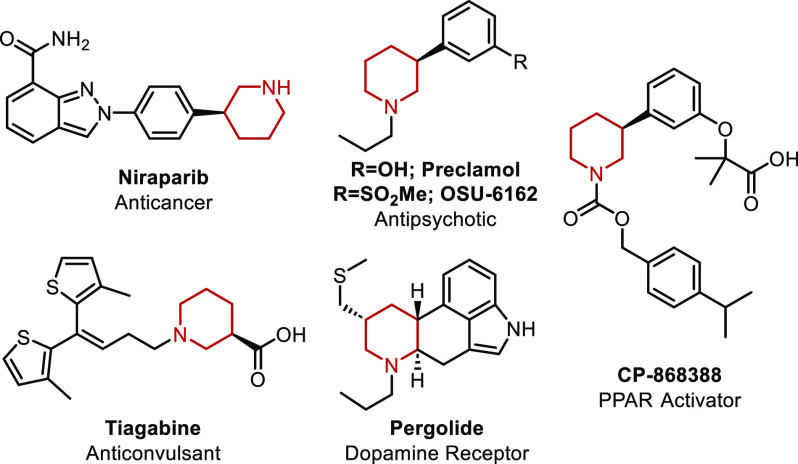
Drug candidates featuring chiral piperidine moieties.

Synthetic access to enantioenriched 3-substituted piperidines has been explored;^13−15^ however, a generally useful asymmetric route for the synthesis of such piperidines has not been reported. Stepwise construction of the piperidine ring suffers from lengthy synthesis and requires either stoichiometric chiral building blocks or resolution to control the absolute stereochemistry. For example, in Merck’s reported process routes for the synthesis of Niraparib, enantiopure piperidines are accessed by chiral HPLC separation or via a biocatalytic transaminase-based dynamic kinetic resolution followed by seven additional steps.^16,17^

A straightforward approach to 3-piperidines would rely on enantioselective reduction or functionalization of pyridine. While the dearomatization of pyridine itself is a high-energy process, a widely used strategy to circumvent this is to reduce and/or functionalize pyridiniums.^18^ Recently, Turner reported that a mixture of oxidase and reductase enzymes^10^ can be used to chemo-enzymatically dearomatize pyridiniums to access enantioenriched 3- and 4-substituted products.

The inherent reactivity of pyridiniums allows for nucleophilic addition at the 2-, 4-, or 6- positions to provide access to 2- or 4-substituted piperidines (Scheme 1A).^18^ However, access to 3-substituted piperidines is not viable using this approach. Rhodium-catalyzed asymmetric Suzuki–Miyaura-type coupling of aryl boronic acids to racemic piperidine-based allyl chlorides provides access to substituted tetrahydropiperidines and, after further reduction, piperidines with high enantioselectivity, though this method requires multiple steps to access the starting allyl chloride.^19,20^

**Scheme 1:**
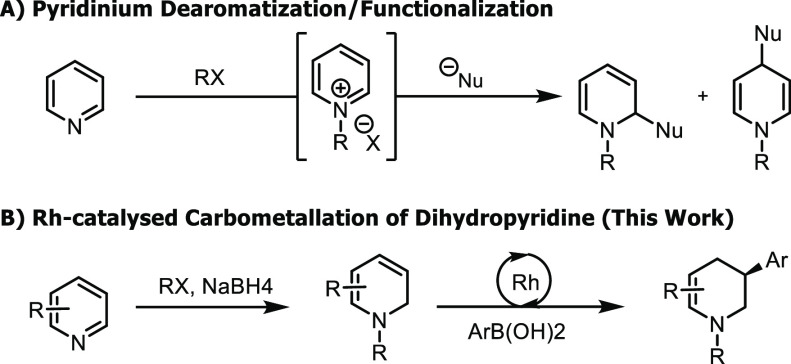
Pyridinium Functionalization and Rh-Catalyzed Carbometalation Approaches to Piperidines

We envisioned that dihydropyridine would serve as an ideal coupling partner to access 3-substituted piperidines through a cross-coupling approach. A Rh-catalyzed asymmetric carbometalation of dihydropyridines could furnish 3-substituted tetrahydropyridines (Scheme 1B) via a reductive Heck-type process and offer access to libraries of enantioenriched 3-piperidines via a three-step process: i) partial reduction of pyridine, ii) Rh-catalyzed asymmetric carbometalation, and then iii) another reduction.

The functionalization of terminal dienes has been extensively studied. However, relatively few studies have reported the successful functionalization of internal and substituted dienes.^21−23^ Dihydropyridines have been used in Diels–Alder reactions to provide complex structures,^24−26^ but catalytic asymmetric functionalization of dihydropyridine is rare and limited to the copper-catalyzed protoborylation reported by Ito.^27^ To the best of our knowledge carbometalation of dienes is unknown.

Rh-catalyzed carbometalation reactions have been reported on strained and bicyclic alkenes.^28−32^ Recently, we have reported a cross-coupling approach for the asymmetric synthesis of cyclobutanes.^28^ The method involved Rh-catalyzed carbometalation of cyclobutenes, and we wondered if carbometalation could be used to selectively modify dihydropyridines.

Initial studies evaluated dihydropyridines as viable starting materials for Rh-catalyzed carbometalation. We attempted synthesis of dihydropyridines (**1**) bearing diverse protecting groups via partial reduction of *in situ*-generated pyridiniums (Scheme 2). Many protecting groups either were incompatible with the reduction conditions or underwent oxidation to provide pyridine. Major challenges with starting material synthesis include the isolation of mixtures of diene isomers, doubly reduced side products, and pyridine impurities. Carbamate protecting groups gave the best results in terms of synthesis and scalability. Specifically, phenyl carbamate-protected dihydropyridine (**1a**) was isolated as a white solid and could be recrystallized to provide high-purity starting material in 72% yield.

**Scheme 2:**
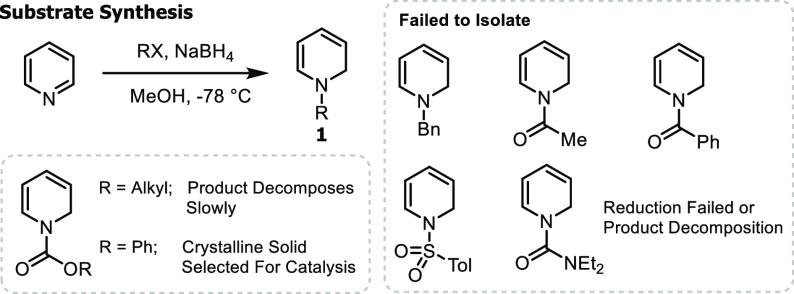
Synthesis of Dihydropyridines

After extensive exploratory work and optimization of the rhodium-catalyzed carbometalation of phenyl carbamate dihydropyridine (**1a**) with phenyl boronic acid (**2a**), we found that the combination of [Rh(cod)(OH)]_2_, (*S*)-Segphos, and aq. CsOH in a THP:toluene:H_2_O (1:1:1) solvent mixture at 70 °C provided tetrahydropyridine **3a** in 81% isolated yield and 96% ee (Table 1, entry 1). Examined deviations from these conditions led to reduced yield and/or enantioselectivity. Poor conversion with recovered starting material was observed at less than 1 M concentration (Table 1, entry 2). Chiral diene- and ferrocene-based ligands provided poor reactivity, whereas other *C*_2_-symmetric bisphosphines examined give lower yields (Table 1, entries 3–5). Aqueous cesium hydroxide appears essential for high yields, as lower conversion was observed using aqueous cesium carbonate (Table 1, entry 6), and cesium carbonate in the absence of water gave only trace product (Table 1, Entry 8). The rhodium source and ligand are crucial for reactivity (Table 1, entries 9 and 10).

**Table 1:**
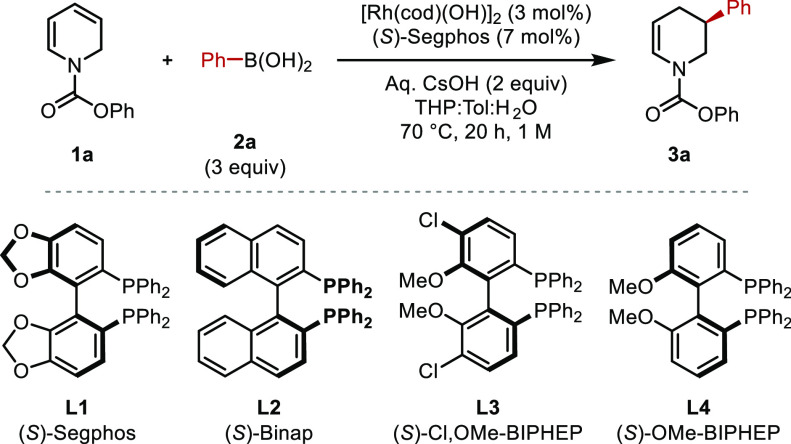
Optimized Reaction Conditions: Standard Deviation Studies

Entry	Deviation from Standard Reaction Condition^a^	Conversion (%)	ee (%)
1	None	95 (81^b^)	96
2	0.5 M instead of 1 M	60	96
3	**L2** instead of **L1**	43	83
4	**L3** instead of **L1**	50	92
5	**L4** instead of **L1**	70	92
6	Cs_2_CO_3_ instead of aq. CsOH	75	94
7	No aq. CsOH	40	96
8	Cs_2_CO_3_ instead of aq. CsOH; no H_2_O	<5	–
9	No [Rh(cod)(OH)]_2_	<5	–
10	No Ligand	<5	–

a Reaction conditions: **1** (0.5 mmol, 1 equiv), **2a** (1.5 mmol, 3 equiv), [Rh(cod)(OH)]_2_ (0.015 mmol, 3 mol%), ligand (0.035 mmol, 7 mol%), aq. CsOH (1 mmol, 2 equiv), THP:Tol (1:1, 0.5 mL), H_2_O (0.25 mL), 70 °C, 20 h.

b Isolated yield (%).

We explored this reaction with a variety of boronic acids (Scheme 3). 4- and 3-substituted phenyl boronic acids featuring diverse functional groups were tested. Electron-withdrawing (**3d**, **3l**, and **3m**) and -donating groups (**3e**–**3h**) in 4-substituted nucleophiles were well tolerated, providing the corresponding products in good yield and high enantioselectivity. Functional groups such as ethers (**3e–3g**), thiols (**3h**), halides (**3i–3k)**, esters (**3l**), and nitriles (**3m**) are compatible with the reaction. Furthermore, 4-pyrazole phenyl boronic acid provided **3n** in 58% yield and 96% ee. The pyrazole motif is featured in Niraparib, and **3n** could potentially provide access to Niraparib and analogues.

**Scheme 3:**
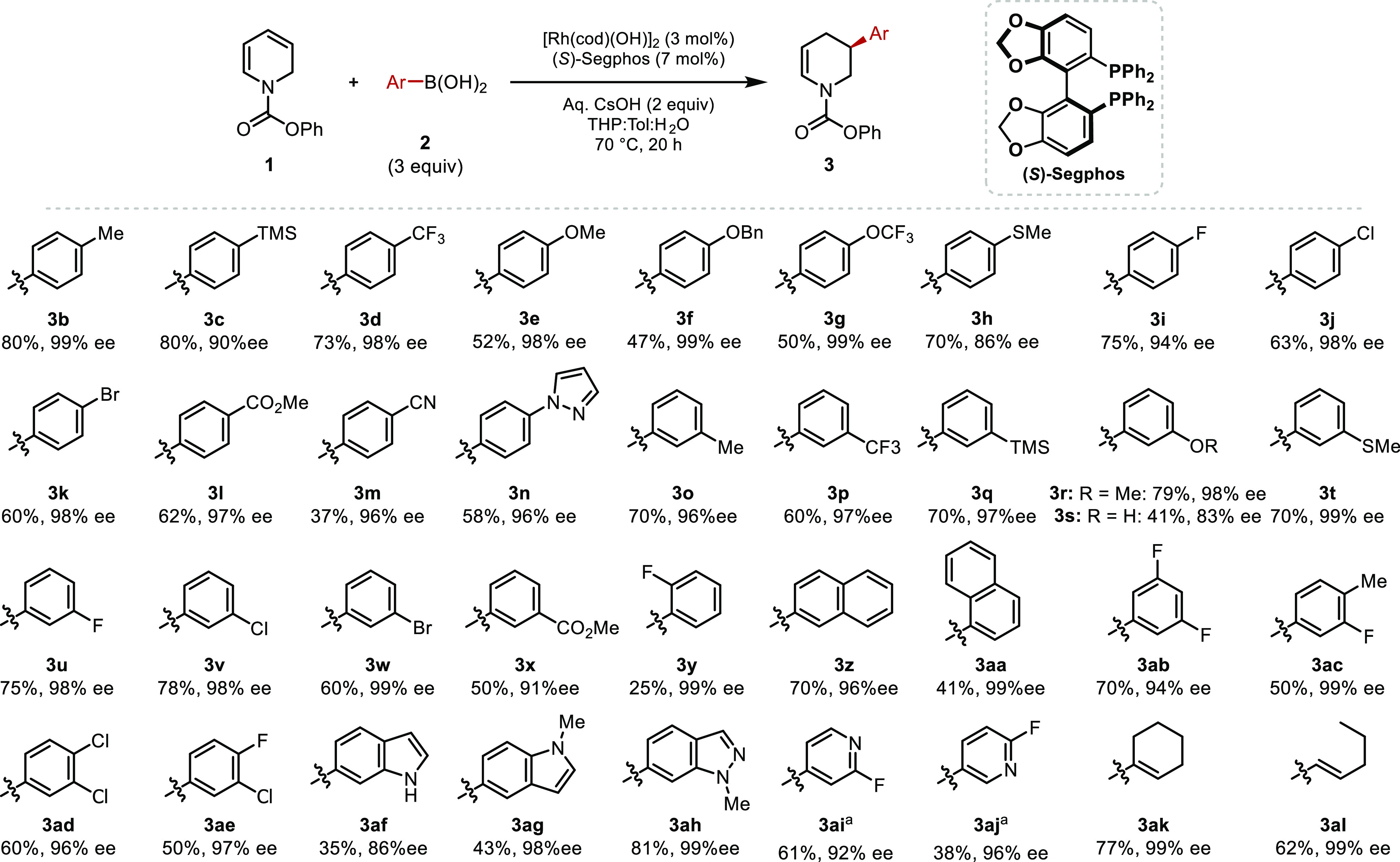
Rhodium-Catalyzed Carbometalation of Dihydropyridines with Aryl Boronic Acids Reaction conducted with [Rh(cod)(OH)]_2_ (0.025 mmol, 5 mol%) and ligand (0.06 mmol, 12 mol%).

A similar selectivity trend was observed for a range of 3-substituted phenyl boronic acids, where broad functional group tolerance was observed, including with an unprotected phenol (**3s**), albeit with reduced yield and ee. The corresponding products (**3o**–**3x**) were isolated in high yields and enantioselectivities. **3r** and **3s** are potential intermediates in syntheses of Preclamol, and they could also serve as precursors for the synthesis of CP-868388 (Figure 1). Ortho-substituted phenyl boronic acids such as 2-methyl phenyl boronic acid and 2-chloro phenyl boronic acid showed poor reactivity; however, we were able to use 2-fluoro phenyl boronic acid and 1-naphthyl boronic acid and isolated **3y** and **3aa** in 25% yield, 99% ee and 41% yield, 99% ee, respectively. Disubstituted phenyl boronic acids were also tested, and **3ab**–**3ad** were isolated in high yields and enantioselectivities (Scheme 3).

Transition metal catalysis with heterocycles is challenging due to catalyst poising. We were delighted to observe that the carbometalation proceeded with boronic acids featuring indole (**3af** and **3ag**) and indazole motifs (**3ah)**. Furthermore, some heterocyclic boronic acids are prone to undergo rapid protodeborylation.^33^ We were able to add 2-fluoro-pyridine boronic acids and isolated **3ai** and **3aj** with high levels of stereoinduction, though a higher (10 mol% Rh) catalyst loading was required for good conversion. Addition of cyclohexenyl and pentenyl boronic acids proceeded smoothly to provide **3ak** and **3al** in high yields and excellent enantioselectivities (Scheme 3).

Next, we tried addition to dihydropyridines featuring other carbamate groups, such as 4-methoxyphenyl carbamate, methyl carbamate, isopropyl carbamate, and benzyl carbamate, that may allow for selective protecting group manipulation, if required, which is relevant to natural product synthesis and drug discovery. The dihydropyridines featuring alkyl carbamates were freshly prepared for the best results. Addition of phenyl boronic acid provided **3am**, **3an**, **3ao**, and **3ap** in high yields and enantioselectivities (Scheme 4).

**Scheme 4:**
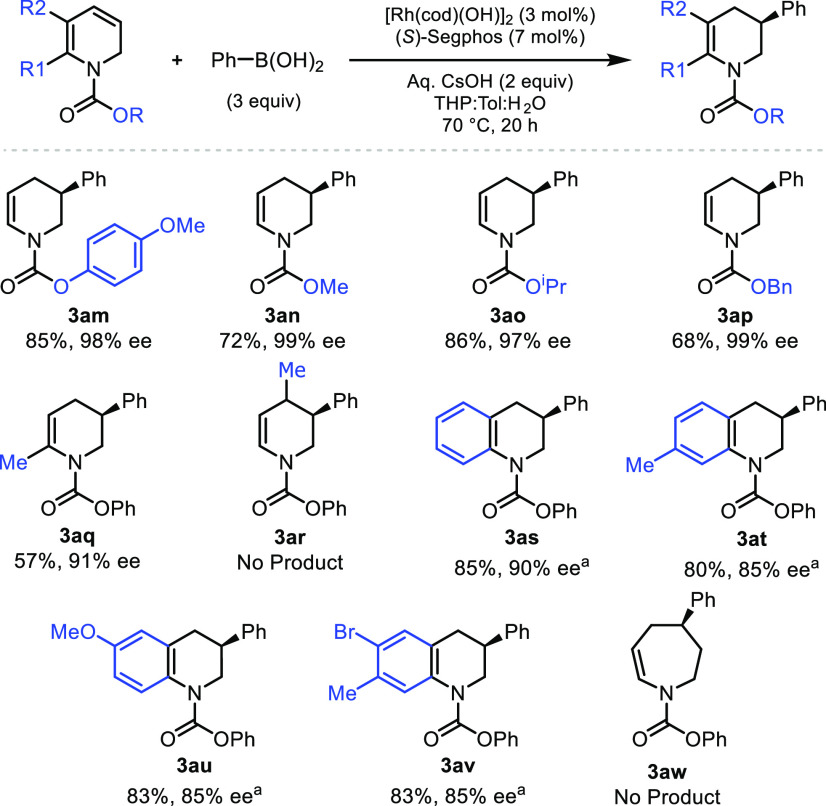
Evaluation of Other Dihydropyridines and Dihydroquinolines Reaction conducted at room temperature for 48 h.

We next examined whether the method developed above was suitable for the addition of other diene–amine partners. However, as anticipated, the synthesis for substituted dihydropyridines is more challenging than starting from the parent pyridine itself, but we were able to prepare suitably protected 2- and 4-methyl dihydropyridines. Asymmetric addition to 2-methyl dihydropyridine gave **3aq** (57%, 91% ee, Scheme 4). However, attempts to use 4-methyl dihydropyridine did not provide **3ar**, even at higher temperatures. Addition to dihydroquinoline provided the asymmetric arylated product (**3as**), though poor enantioselectivity was observed using our standard conditions, and we note that these substrates are qualitatively more reactive than dihydropyridines. Performing reactions on dihydroquinolines at 30 °C, however, allows **3as** to be isolated in 85% yield with 90% ee after 48 h. Substituted dihydroquinolines were also successfully employed, and **3at** to **3av** could be isolated with >80% yield and ∼85% ee (Scheme 4). We attempted addition to 7-membered ring (azepine) featuring diene–amino carbamate, though no desired product (**3aw**) was observed.

To test the scalability of the method, we performed the reaction on a 5 mmol scale, and 1.10 g of **3a** was isolated (81% yield, 96% ee, Scheme 5A). Multiply substituted dihydropyridines can also potentially be accessed by manipulating tetrahydropyridine products **3** obtained in the reductive Heck reaction. As a proof of concept, we treated **3a** with NBS in the presence of methanol to give trisubstituted piperidine **4** in 86% yield in a 4:1 diastereomeric ratio (Scheme 5A).

**Scheme 5:**
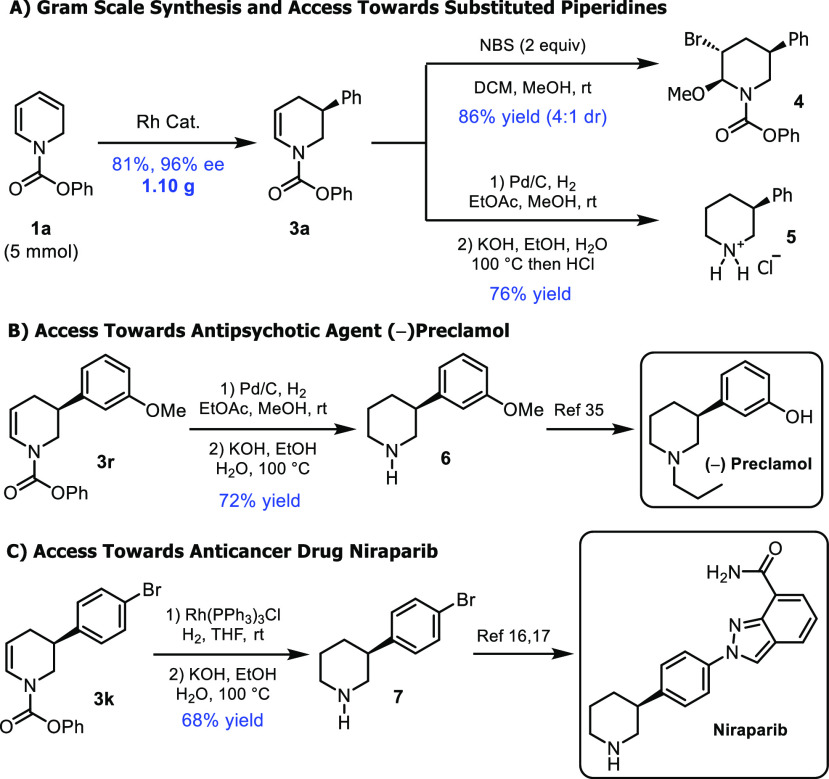
Synthesis of Enantioenriched Piperidines

As described above, simple (monosubstituted) 3-substituted piperidines exhibit a vast array of important bioactivities. To access these targets, a simple hydrogenation/deprotection strategy was used. Palladium-on-carbon-mediated hydrogenation followed by carbamate deprotection using aqueous potassium hydroxide in methanol led to piperidines **5** and **6** in 76% and 72% yields, respectively (over two steps). **6** is a known precursor to (−)-Preclamol (Scheme 5B). The absolute configuration of the products was assigned by comparing the optical rotation of **5**(34) and **6**(34−36) to literature values. Wilkinson’s catalyst-mediated hydrogenation of **3k** and basic deprotection gave **7**, a Niraparib precursor,^16,17^ in 68% yield over two steps (Scheme 5C).

To get a preliminary mechanistic understanding of the reaction, we conducted deuterium labeling experiments (Scheme 6A). Reaction with d5-phenyl boronic acid (**d**_**5**_**-2a**) led to **8**, with no deuterium incorporation observed in the tetrahydropyridine ring. Next, we replaced the aqueous cesium hydroxide with cesium carbonate and conducted the reaction in D_2_O to produce tetrahydropyridine **9** (Scheme 6A), featuring exclusive deuterium incorporation in the 4-position. In prior reports of Rh-catalyzed carbometalations of bicyclic strained alkenes,^29−31^ and in our prior work on the carbometalation on cyclobutene, 1,4-Rh-shifts were often observed after carbometalation,^28^ but here no 1,4-Rh shift is observed for dihydropyridines.

**Scheme 6:**
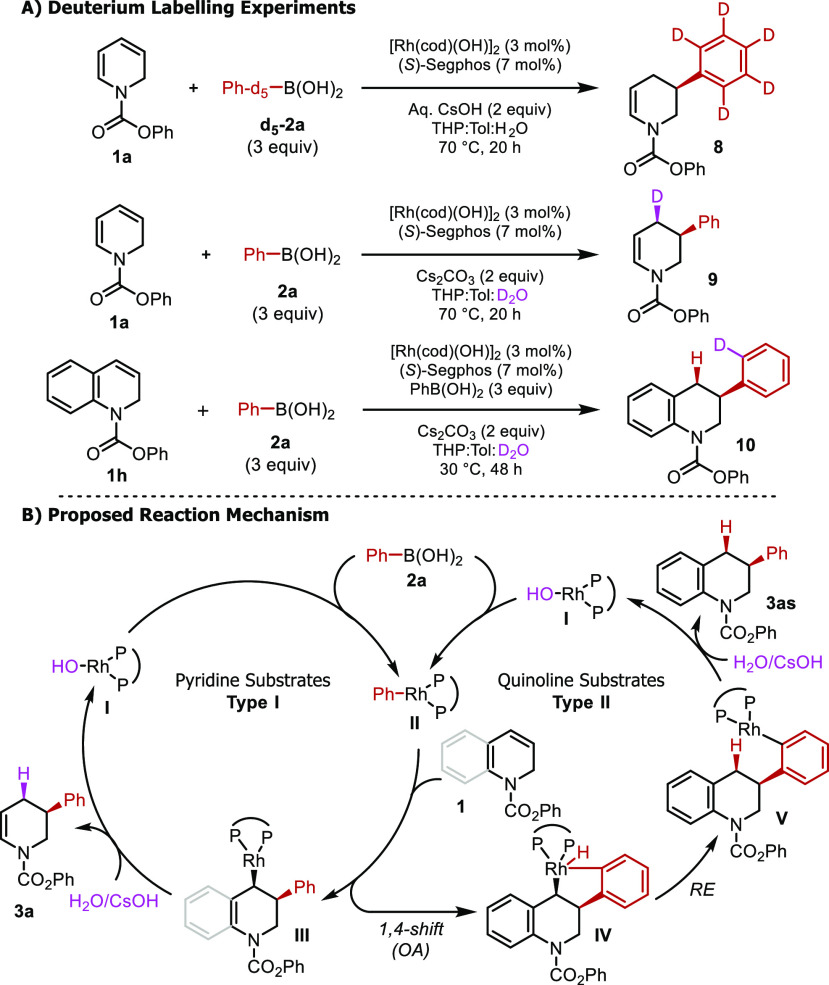
Mechanistic Hypothesis and Studies

A prior report of Rh-catalyzed carbometalation with chromenes did note 1,4-Rh shifts, and the noticeable difference in the kinetics and enantioselectivity that we observed with dihydropyridine and dihydroquinoline substrates inspired us to conduct further experiments on **1h**. Interestingly, **10** was observed when D_2_O was used, featuring deuterium incorporation exclusively on the benzene ring, clearly indicating that 1,4-Rh shifts occur with the dihydroquinolines (Scheme 6A).

Based on these deuterium studies, we propose two different mechanistic pathways, depending on the starting material. First, coordination of **L1** with Rh(cod)(OH)]_2_ provides **I**. Transmetalation with phenyl boronic acid gives Rh-complex **II**. Carbometalation of **1a** or **1h** provides complex **III**. We postulate that the dihydropyridine-derived complex **III** undergoes regioselective protodemetalation in the presence of water to provide **3a** and regenerates **I** (Scheme 6B, Mechanism Type 1). Complexes of type **III** that are dihydroquinoline derived, however, undergo a 1,4-Rh shift to give **V**, possibly via **IV**. Finally, protodemetalation in the presence of water leads to product **3as** and regenerates **I** (Scheme 6B, Mechanism Type II).

We present a new synthetic strategy to access enantioenriched piperidines involving highly regio- and enantioselective Rh-catalyzed carbometalation of dihydropyridine to provide 3-substituted tetrahydropyridines. The method has broad functional group tolerance and can be performed on a gram scale, and the products are valuable precursors to enantioenriched piperidines. The utility of the reaction was demonstrated by the formal syntheses of Preclamol and Niraparib. Further, the tetrahydropyridine products contain useful functional groups which should allow them to serve as precursors to complex synthetic targets, for example by producing heavily functionalized trisubstituted piperidines such as **4**.

## Supporting Information Available

Experimental procedures, compound synthesis and characterization, and supporting discussion (PDF)
