# Supplementary material for: Catalytic Enantioselective Synthesis of 3-Piperidines from Arylboronic Acids and Pyridine
Source: J Am Chem Soc. 2023 Jun 22;145(26):14221–6. doi: 10.1021/jacs.3c05044 (PMC10326878; doi:10.1021/jacs.3c05044)
Supplement: Supplementary file 1 — ja3c05044_si_001.pdf [file ja3c05044_si_001.pdf]

Catalytic Enantioselective Synthesis of 3-Piperidines from Arylboronic Acids and Pyridine

Sourabh Mishra, Sedef Karabiyikoglu, Stephen P. Fletcher*

Department of Chemistry, Chemistry Research Laboratory, University of Oxford, Oxford OX1 3TA, United Kingdom

Supporting Information – Experimental procedures and Data

Table of Contents

1. General Information 2

2. Synthesis of Dihydropyridines.. 3

3. General Procedure for Rh-Catalyzed Cross-Coupling 6

4. Derivatization of Chiral Tetrahydropyridines 54

5. References 59

6. NMR Spectra..60

1. General Information

All reactions were carried out in anhydrous solvents with continuous magnetic stirring under an inert argon atmosphere. Heating was performed using DrySyn heating blocks.

Nuclear magnetic resonance (NMR) spectroscopy measurements were carried out at room temperature. ^{1}H NMR, ^{13}C NMR, ^{19}F NMR, COSY, HSQC, HMBC and NOESY experiments were carried out using Bruker AVIII HD 400 (400/100 MHz) or AVIII HD 400 (500/125 MHz) spectrometers. Chemical shifts (δ) are reported in ppm relative to the residual solvent peak with corresponding coupling constants (*J*) in Hertz (Hz) and multiplicities (s: singlet, d: doublet, t: triplet, q: quartet, m: multiplet and combinations of these). Assignment follows HSQC, COSY, HMBC or/and NOESY spectra, chemical shift and coupling constant analysis.

Optical rotations ($[\alpha]_{25}^{D}$) were recorded using a Perkin Elmer-241 Polarimeter. Concentrations (c) are reported in g/100 mL.

Chiral SFC (supercritical fluid chromatography) separations were conducted on a Waters Acquity UPC2 system using Waters Empower software. Solvents used were of HPLC grade (Fisher Scientific, Sigma Aldrich or Rathburn). Chiralpak columns (150x3 mm, particle size 3 μm) were used at 1500 PSI, 30 °C, flow: 1.5 mL/min under one of the following gradients- **Gradient 1**: 1% to 30% MeOH in 5 min, 30% to 50% MeOH in 0.5 min, hold 50% MeOH for 2 min; **Gradient 2**: 1% to 50% MeOH in 2 min, hold 50% MeOH for 5 min; **Gradient 3**: 0% to 15% MeOH in 7.5 min

High Resolution Mass spectra were carried out on a Walters BioAccord LC-MS System using Electron spray ionisation (ESI^{+}) loop injection MS at the University of Oxford.

Commercially available reagents and ligands were purchased from Sigma Aldrich, Alfa Aesar, Acros Organics, Fluorochem and Strem Chemicals and were used without further purification unless stated otherwise. $[Rh(cod)OH]_2$ was bought from Sigma Aldrich. All aryl and heteroarylboronic acids were purchased and used without additional purification unless stated otherwise. Dry solvents were purchased from Thermo Scientific™, Extra Dry over Molecular Sieve, Stabilized, AcroSeal™ and were degassed with argon prior to usage. Deuterated solvents were purchased from Sigma Aldrich.

2. Synthesis of Dihydropyridines

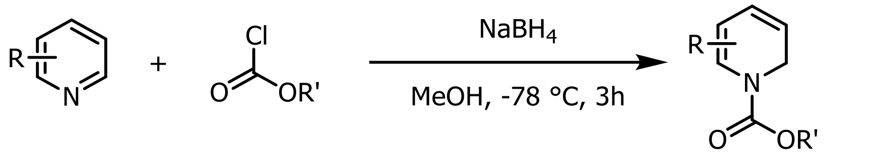

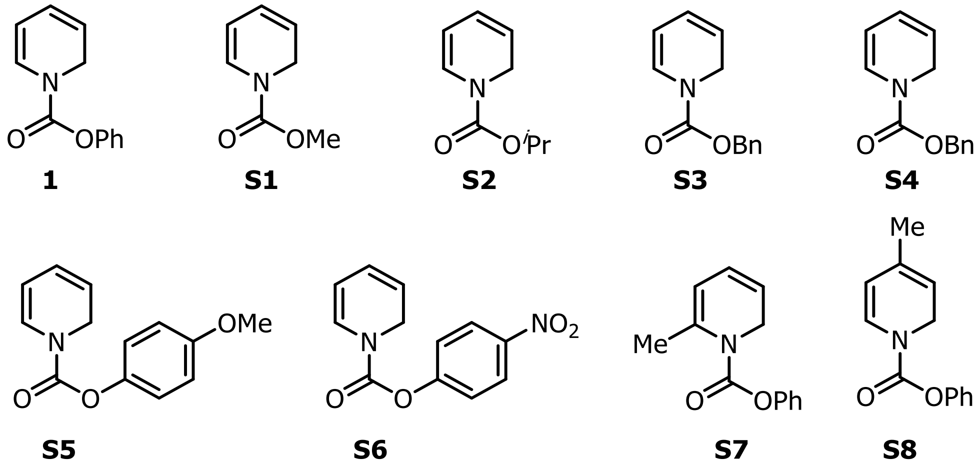

Chloroformate (20 mmol, 1 equiv) was added dropwise under nitrogen to a MeOH solution (50 mL) of $NaBH_4$ (20.0 mmol), pyridine (20 mmol) at −78 °C. The reaction was maintained at −78 °C for 3 h and then quenched by water (50 ml). The mixture was extracted with Et_2O (30 ml) two times. The combined organic layer was washed with 1N NaOH (two times) followed by 1N HCl (two times) then dried over sodium sulfate. After filtration, the solvents were removed by evaporation. The crude mixture was purified by a short pad of silica gel with acetone/hexane (2% to 10% gradient) as an eluent. The solvent was removed by evaporation under reduced pressure to obtain **1** as white solid. The product was then recrystallized in methanol providing **1** (72% yield) as white crystal.

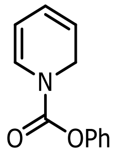

1

Phenyl pyridine-1(2H)-carboxylate (1): ^{1}H NMR (400 MHz, $CDCl_3$) (2 rotamers): δ (ppm) 7.42 – 7.33 (m, 2H), 7.25 – 7.19 (m, 1H), 7.18 – 7.10 (m, 2H), 6.92 – 6.76 (m, 1H), 5.95 – 5.86 (m, 1H), 5.65 – 5.53 (m, 1H), 5.33 – 5.17 (m, 1H), 4.67 – 4.38 (m, 2H). The spectroscopic data satisfactorily matched previously reported data.[1,2]

Other 1,2-dihydropyridines S1−S8 were prepared from the corresponding pyridines according to the procedure described above. The spectroscopic data for 1,2-dihydropyridines S1−S8 satisfactorily matched previously reported data.[1,2] 1,2-dihydropyridines (**S1**−**S8**) were used immediately in order to prevent decomposition.

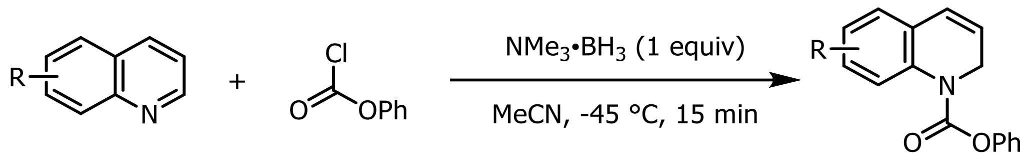

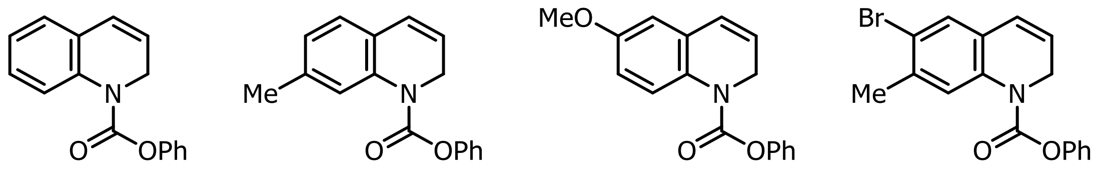

S9 **S10** **S11** **S12**

A solution of NMe_3BH_3 (5.5 mmol, 1.1 equiv) in MeCN (5 mL) was added dropwise to a flask containing quinoline (5 mmol, 1 equiv) and phenyl chloroformate (6 mmol, 1.2 equiv) in MeCN (5 mL) at –40 °C and stirred for 5 minutes. The reaction was stirred for 10 minutes at room temperature. The product was directly purified by column chromatography on silica gel. The spectroscopic data for Phenyl quinoline-1(2H)-carboxylate (**S9**) satisfactorily matched previously reported data.[3]

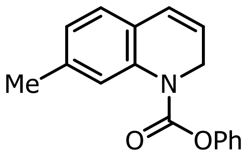

Phenyl 7-methylquinoline-1(2H)-carboxylate (S10): The corresponding compound was prepared following the procedure above using phenyl chloroformate and 7-methylquinoline. Purification by flash chromatography (2% acetone/petrol to 25% acetone/petrol) afforded compound **S10** as white solid (82% yield).

^{1}H NMR ($CDCl_3$, 400 MHz): δ (ppm) 7.50 (dd, *J* = 17.4, 4.2 Hz, 1H), 7.32 (tt, *J* = 7.6, 2.2 Hz, 3H), 7.21 – 7.03 (m, 5H), 6.94 (d, *J* = 7.6 Hz, 1H), 6.72 (dq, *J* = 9.7, 1.0 Hz, 2H), 6.10 (dt, *J* = 9.7, 4.2 Hz, 1H), 4.43 (dd, *J* = 4.2, 1.4 Hz, 3H), 2.32 (s, 3H). **^{13}C NMR** (101 MHz, $CDCl_3$): δ (ppm) 152.7, 151.2, 136.1, 133.9, 129.4, 127.0, 126.9, 126.7, 125.6, 123.7, 122.0, 121.7, 115.4, 43.2, 19.2. **HRMS** (ESI): m/z calcd for $C_{17}H_{16}O_2N^+$ $[M + H]^+$ 266.1176 found 266.1194. **IR** (ν_{max}/cm^{-1}) 2361, 1731, 1642, 1608, 1495, 1435, 1408, 1379, 1349, 1257, 1237, 1207, 1082, 1059, 900, 773, 754, 713.

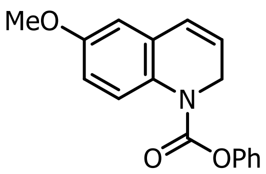

Phenyl-6-methoxyquinoline-1(2H)-carboxylate (S11): The corresponding compound was prepared following general procedure **2** using phenyl chloroformate and 6-methoxyquinoline. Purification by flash chromatography (2% acetone/petrol to 25% acetone/petrol) afforded compound **S11** as white solid (76% yield).

^{1}H NMR ($CDCl_3$, 400 MHz) δ (ppm) 7.61 (bs, 1H), 7.43 – 7.34 (m, 2H), 7.26 – 7.14 (m, 3H), 6.80 (dd, *J* = 8.9, 2.9 Hz, 1H), 6.68 (d, *J* = 2.9 Hz, 1H), 6.54 (d, *J* = 9.6 Hz, 1H), 6.11 (dt, *J* = 8.8, 3.9 Hz, 1H), 4.53 (s, 2H), 3.81 (s, 2H). **^{13}C NMR** (126 MHz, $CDCl_3$) δ (ppm) 156.8, 151.2, 129.5, 129.4, 128.6, 126.6, 125.6, 124.9, 121.7, 120.2, 115.4, 112.9, 111.4, 55.5, 44.0. **IR** (ν_{max}/cm^{-1}) 2361, 1729, 1593, 1557, 1494, 1463, 1372, 1339, 1281, 1227, 1204, 1163, 1123, 772, 752, 687.

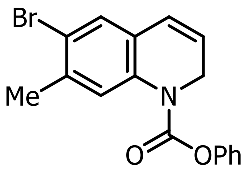

phenyl 6-bromo-7-methylquinoline-1(2H)-carboxylate (S12): The corresponding compound was prepared following general procedure **2** using phenyl chloroformate and 6-bromo-7-methylquinoline. Purification by flash chromatography (2% acetone/petrol to 25% acetone/petrol) afforded compound **S12** as viscous liquid (80% yield).

^{1}H NMR ($CDCl_3$, 500 MHz) δ (ppm) 7.64 (bs, 1H), 7.40 (tt, *J* = 7.7, 2.2 Hz, 2H), 7.29 – 7.20 (m, 2H), 7.17 (dt, *J* = 8.9, 1.8 Hz, 2H), 6.47 (d, *J* = 9.6 Hz, 1H), 6.05 (dt, *J* = 9.0, 4.1 Hz, 1H), 4.54 (s, 2H), 2.37 (s, 3H). **^{13}C NMR** (126 MHz, $CDCl_3$) δ (ppm) 152.6, 151.1, 137.2, 135.1, 129.7, 129.5, 127.7, 125.9, 125.5, 121.8, 120.3, 44.0, 23.2. **HRMS** (ESI): m/z calcd for $C_{17}H_{15}O_2NBr^+$ [M + H]$^+$ 344.0281 found 344.0285. **IR** (ν_{max}/cm^{-1}) 2361, 1725, 1595, 1494, 1390, 1365, 1332, 1276, 1231, 1204, 1161, 1048, 1030, 885, 771, 749, 689.

3. General Procedure for Rh-Catalyzed Cross-Coupling

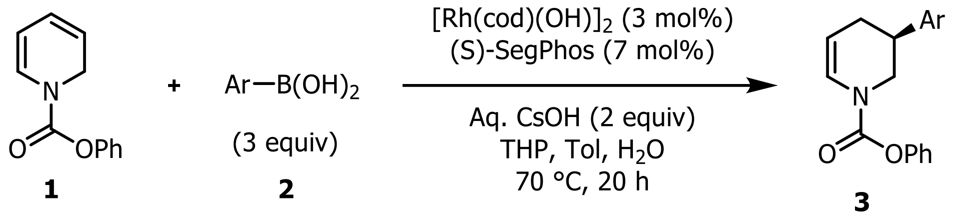

General Procedure A: $[Rh(cod)OH]_2$ (6.9 mg, 0.015 mmol, 3 mol%) and (S)-Segphos (21.4 mg, 0.035 mmol, 7 mol%) were added to a 7 mL dram vial equipped with a magnetic stir bar and sealed with a rubber septum. The vial was put under reduced pressure then purged with argon, this was repeated three times. Toluene (0.25 mL), THP (0.25 mL), H_2O (0.25 mL) followed by aq. CsOH (50 wt%, 180 µL, 1 mmol, 2.0 equiv) was added to the vial and the catalyst solution was stirred at 70 °C. After 10 min, boronic acid **2** (1.5 mmol, 3.0 equiv) then dihydropyridine **1** (0.5 mmol, 1 equiv) was added and the resulting mixture was stirred at 70 °C for 20 hours unless stated otherwise. Upon completion of reaction, the mixture was cooled to room temperature and diluted with Et_2O (5 mL) before passing through a plug of SiO_2. The plug was washed will additional 20 mL of Et_2O and the solvents were removed in vacuo. Purification by flash chromatography afforded the desired product **3**.

Racemates: Racemic samples were synthesized with (±)-SegPhos instead of (S)-Segphos.

Upscale: Larger-scale experiments was performed in 5 mmol scale in direct analogy to the general procedure B in a 25 ml round-bottom flask.

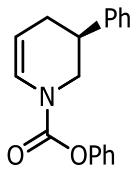

Phenyl-(S)-3-phenyl-3,4-dihydropyridine-1(2H)-carboxylate (3a): The corresponding compound was prepared following general procedure **A** using phenyl boronic acid and phenyl pyridine-1(2H)-carboxylate. The mixture was stirred at 70 °C for 20 hours. Purification by flash chromatography (2% acetone/petrol to 10% acetone/petrol) afforded compound **3a** as viscous liquid (80% yield, 96% ee).

^{1}H NMR (400 MHz, $CDCl_3$) (2 rotamers): δ (ppm) 7.44 – 7.33 (m, 4H), 7.32 – 7.01 (m, 7H), 5.25 – 5.14 (m, 1H), 4.35 – 4.29 (m, 1H), 3.55 – 3.29 (m, 1H), 3.11 – 3.07 (m, 1H), 2.45 – 2.26 (m, 2H). **^{13}C NMR** (101 MHz, $CDCl_3$) (2 rotamers): δ (ppm) 152.1, 151.8, 151.3, 151.1, 142.8, 142.7, 129.5, 129.4, 128.9, 128.85, 127.4, 127.3, 127.2, 127.1, 125.7, 125.7, 125.3, 125.0, 121.8, 121.7, 107.9, 107.5, 48.6, 48.0, 38.8, 38.6, 29.5, 29.3.

HRMS (ESI): m/z calcd for $C_{18}H_{18}O_2N^+$ [M + H]$^+$ 280.1332 found 280.1328.

IR (ν_{max}/cm^{-1}) 1723, 1406, 1362, 1252, 1200, 751, 700, 689.

SFC Conditions: Chiralpak IC; Gradient 1; 99:1 er (major enantiomer t_R = 4.08 min; minor enantiomer t_R = 4.28 min), **98% ee.** **[α]^{25}$_D$ =** −41.8 (c = 2.0, $CHCl_3$).

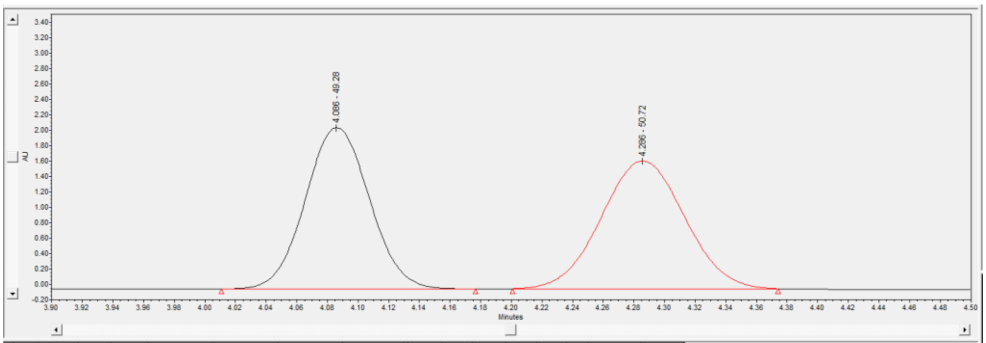

	Name	Retention Time (min)	Purity1 Angle	Purity1 Threshold	PDA/FLR Match1 Spect. Name	PDA/FLR Match1 Angle	PDA/FLR Match1 Threshold	PDA/FLR Match1 Lib. Name	Area (μV*sec)	% Area	Height (μV)	Int Type	Amount	Units	Peak Type	Peak Codes
1		4.086							6091822	49.28	2089413	bb			Unknown	
2		4.286							6270918	50.72	1655276	bb			Unknown	

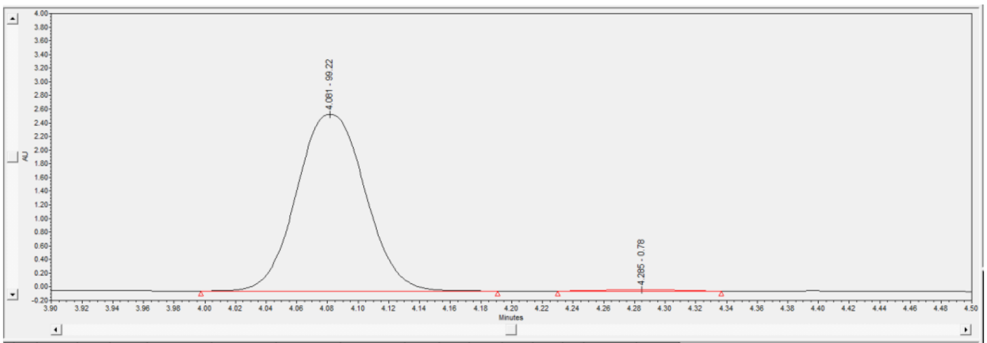

	Name	Retention Time (min)	Purity1 Angle	Purity1 Threshold	PDA/FLR Match1 Spect. Name	PDA/FLR Match1 Angle	PDA/FLR Match1 Threshold	PDA/FLR Match1 Lib. Name	Area (μV*sec)	% Area	Height (μV)	Int Type	Amount	Units	Peak Type	Peak Codes
1		4.081							8002223	99.22	2586421	bb			Unknown	
2		4.285							62947	0.78	19302	bb			Unknown	

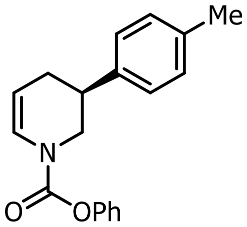

Phenyl-(S)-3-(p-tolyl)-3,4-dihydropyridine-1(2H)-carboxylate (3b): The corresponding compound was prepared following general procedure **A** using *p*-tolyl boronic acid and phenyl pyridine-1(2H)-carboxylate. The mixture was stirred at 70 °C for 20 hours. Purification by flash chromatography (2% acetone/petrol to 10% acetone/petrol) afforded compound **3b** as white solid (80% yield, 99% ee).

^{1}H NMR (400 MHz, $CDCl_3$) (2 rotamers): δ (ppm) 7.38 (tdd, *J* = 9.3, 6.3, 1.8 Hz, 2H), 7.26 – 7.00 (m, 8H), 5.27 – 5.10 (m, 1H), 4.36 – 4.25 (m, 1H), 3.53 – 3.23 (m, 1H), 3.18 – 3.00 (m, 1H), 2.44 – 2.27 (m, 5H). **^{13}C NMR** (101 MHz, $CDCl_3$) (2 rotamers): δ (ppm) 152.1, 151.8, 151.3, 151.1, 139.8, 139.7, 136.8, 136.7, 129.6, 129.5, 129.48, 129.4, 127.2, 127.18, 125.7, 125.6, 125.2, 124.9, 121.8, 121.7, 107.9, 107.5, 48.7, 48.1, 38.3, 38.2, 29.5, 29.4, 21.1.

HRMS (ESI): m/z calcd for $C_{19}H_{20}O_2N^+$ $[M + H]^+$ 294.1489 found 294.1491.

IR (ν_{max}/cm^{-1}) 1724, 1405, 1359, 1251, 1199, 1163, 1074, 974, 813, 751, 728, 689.

SFC Conditions: Chiralpak IE; Gradient 1; 99.5:0.5 er (major enantiomer t_R = 4.31 min; minor enantiomer t_R = 4.19 min), **99% ee**. **$[\alpha]^{25}_D$ =** −54.5 (c = 2.0, $CHCl_3$).

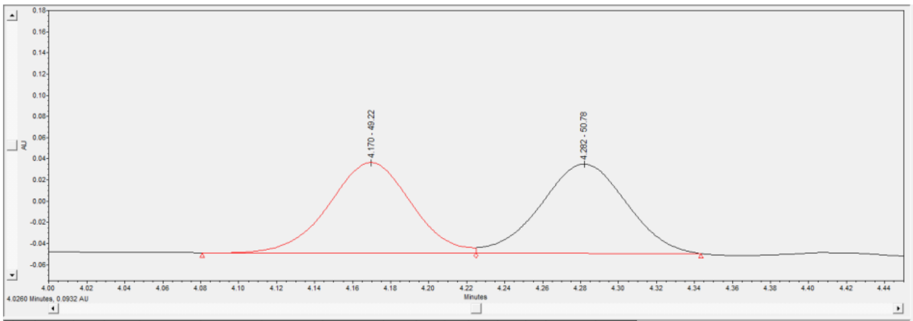

	Name	Retention Time (min)	Purity1 Angle	Purity1 Threshold	PDA/FLR Match1 Spect. Name	PDA/FLR Match1 Angle	PDA/FLR Match1 Threshold	PDA/FLR Match1 Lib. Name	Area (μV*sec)	% Area	Height (μV)	Int Type	Amount	Units	Peak Type	Peak Codes
1		4.170							257287	49.22	85466	bv			Unknown	
2		4.282							265439	50.78	84302	vb			Unknown	

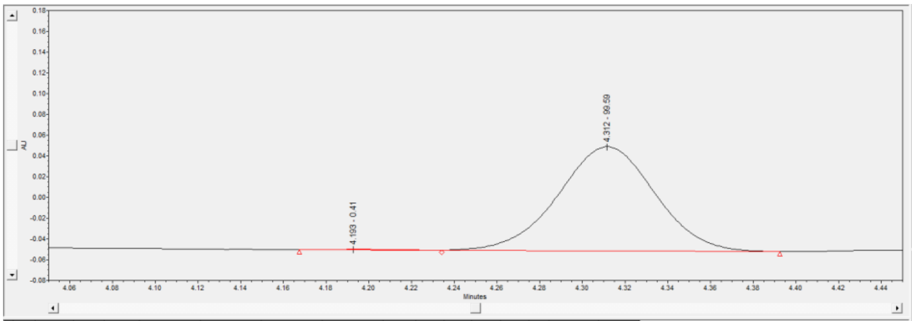

	Name	Retention Time (min)	Purity1 Angle	Purity1 Threshold	PDA/FLR Match1 Spect. Name	PDA/FLR Match1 Angle	PDA/FLR Match1 Threshold	PDA/FLR Match1 Lib. Name	Area (μV*sec)	% Area	Height (μV)	Int Type	Amount	Units	Peak Type	Peak Codes
1		4.193							1299	0.41	652	bv			Unknown	
2		4.312							316370	99.59	100426	vb			Unknown	

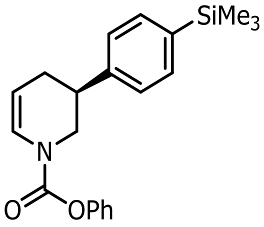

Phenyl-(S)-3-(4-(trimethylsilyl)phenyl)-3,4-dihydropyridine-1(2H)-carboxylate (3c): The corresponding compound was prepared following general procedure **A** using 4-(trimethylsilyl)phenyl boronic acid and phenyl pyridine-1(2H)-carboxylate. The mixture was stirred at 70 °C for 20 hours. Purification by flash chromatography (100% petrol to 5% acetone/petrol) afforded compound **3c** as viscous liquid (80% yield, 90% ee).

^{1}H NMR (500 MHz, $CDCl_3$) (2 rotamers): δ (ppm) 7.55 – 7.49 (m, 2H), 7.35 (dt, J = 14.3, 8.0 Hz, 2H), 7.29 – 6.97 (m, 6H), 5.23 – 5.12 (m, 1H), 4.31 (dt, J = 11.7, 4.8 Hz, 1H), 3.51 – 3.31 (m, 1H), 3.16 – 3.00 (m, 1H), 2.43 – 2.26 (m, 2H), 0.27 (s, 9H). **^{13}C NMR** (126 MHz, $CDCl_3$) (2 rotamers): δ (ppm) 152.1, 151.8, 151.2, 151.1, 143.4, 143.2, 139.2, 139.1, 133.9, 133.89, 129.5, 129.4, 126.8, 126.78, 125.7, 125.6, 125.3, 124.9, 121.8, 121.7, 107.9, 107.5, 48.5, 47.9, 38.7, 38.6, 29.4, 29.2, -1.0.

HRMS (ESI): m/z calcd for $C_{21}H_{26}O_2NSi^+$ [M + H]$^+$ 352.1727 found 352.1721.

IR (ν_{max}/cm^{-1}) 1727, 1656, 1495, 1406, 1360, 1249, 1202, 1163, 840, 816, 752, 724, 689.

SFC Conditions: Chiralpak IB; Gradient 1; 95:5 er (major enantiomer t_R = 2.98 min; minor enantiomer t_R = 2.86 min), **90% ee**. **$[\alpha]^{25}_D$ =** −65.5 (c = 2.0, $CHCl_3$).

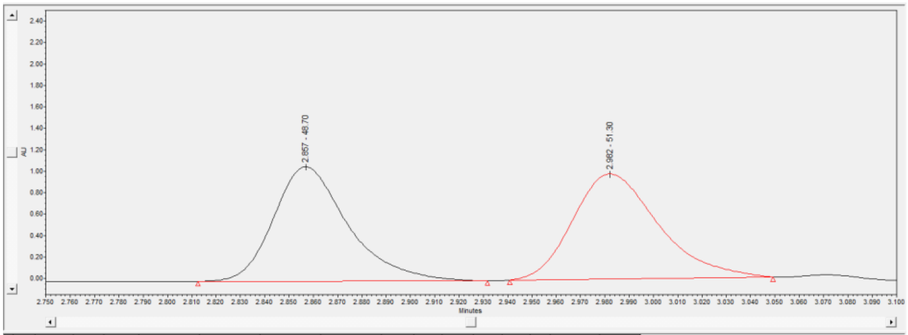

	Name	Retention Time (min)	Purity1 Angle	Purity1 Threshold	PDA/FLR Match1 Spect. Name	PDA/FLR Match1 Angle	PDA/FLR Match1 Threshold	PDA/FLR Match1 Lib. Name	Area (µV*sec)	% Area	Height (µV)	Int Type	Amount	Units	Peak Type	Peak Codes
1		2.857							2272025	48.70	1066600	bb			Unknown	
2		2.982							2392993	51.30	978027	bb			Unknown	

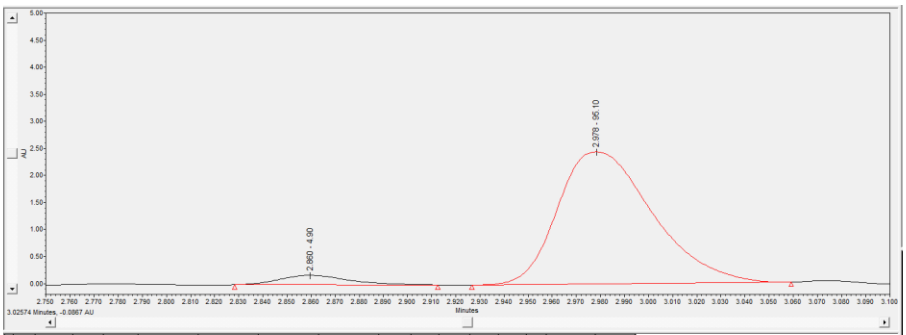

	Name	Retention Time (min)	Purity1 Angle	Purity1 Threshold	PDA/FLR Match1 Spect. Name	PDA/FLR Match1 Angle	PDA/FLR Match1 Threshold	PDA/FLR Match1 Lib. Name	Area (µV*sec)	% Area	Height (µV)	Int Type	Amount	Units	Peak Type	Peak Codes
1		2.860							343711	4.90	174833	bb			Unknown	
2		2.978							6668319	95.10	2441620	bb			Unknown	

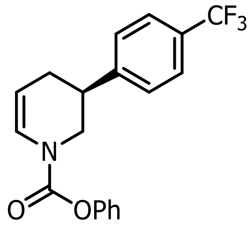

Phenyl-(S)-3-(4-(trifluoromethyl)phenyl)-3,4-dihydropyridine-1(2H)-carboxylate (3d): The corresponding compound was prepared following general procedure **A** using 4-(trifluoromethyl)phenyl boronic acid and phenyl pyridine-1(2H)-carboxylate. The mixture was stirred at 70 °C for 20 hours. Purification by flash chromatography (2% acetone/petrol to 15% acetone/petrol) afforded compound **3d** as viscous liquid (73% yield, 98% ee).

^{1}H NMR (400 MHz, $CDCl_3$) (2 rotamers): δ (ppm) 7.59 (dt, J = 8.8, 4.7 Hz, 2H), 7.44 – 7.29 (m, 4H), 7.23 – 6.99 (m, 4H), 5.25 – 5.11 (m, 1H), 4.27 (ddt, J = 12.5, 5.1, 2.5 Hz, 1H), 3.56 – 3.33 (m, 1H), 3.17 (dddd, J = 20.2, 10.0, 7.6, 3.8 Hz, 1H), 2.46 – 2.26 (m, 2H). **^{13}C NMR** (101 MHz, $CDCl_3$) (2 rotamers): δ (ppm) 152.0, 151.8, 151.2, 151.0, 146.9, 146.7, 129.5, 129.48, 127.8, 127.7, 125.8, 125.8, 125.6, 125.2, 121.8, 121.6, 107.4, 107.0, 48.2, 47.5, 38.6, 38.5, 29.2, 29.1. **^{19}F NMR** (376 MHz, $CDCl_3$) (2 rotamers) δ (ppm) −62.4, −62.5.

HRMS (ESI): m/z calcd for $C_{19}H_{17}F_3O_2N^+$ $[M + H]^+$ 348.1206 found 348.1206.

IR (ν_{max}/cm^{-1}) 1725, 1408, 1327, 1202, 1164, 1122, 1068, 1018, 834, 755.

HPLC Conditions: Chiralpak IE; Isocratic 1% IPA/Hexane 1ml/min; 99:1 er (major enantiomer t_R = 19.5 min; minor enantiomer t_R = 22.1 min), **98% ee**. **$[\alpha]^{25}_D$ =** −33.7 (c = 2.0, $CHCl_3$).

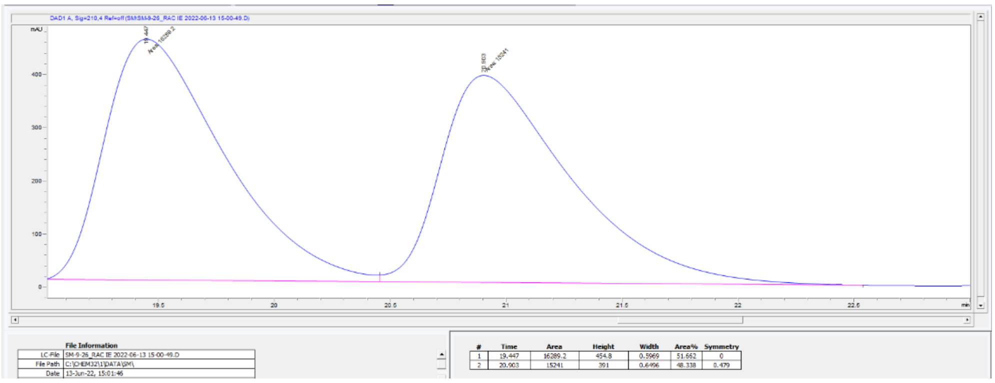

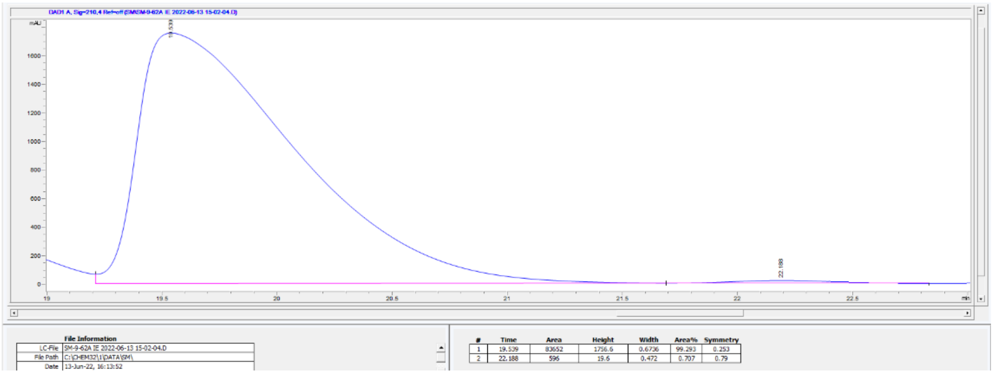

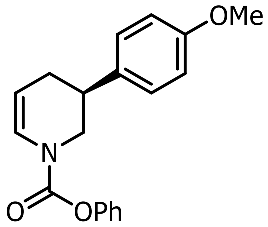

Phenyl-(S)-3-(4-methoxyphenyl)-3,4-dihydropyridine-1(2H)-carboxylate (3e): The corresponding compound was prepared following general procedure **A** using 4-methoxyphenyl boronic acid and phenyl pyridine-1(2H)-carboxylate. The mixture was stirred at 70 °C for 20 hours. Purification by flash chromatography (2% acetone/petrol to 20% acetone/petrol) afforded compound **3e** as viscous liquid (52% yield, 98% ee).

^{1}H NMR (400 MHz, $CDCl_3$) (2 rotamers): δ (ppm) 7.37 (td, *J* = 8.7, 7.2 Hz, 2H), 7.26 – 6.99 (m, 6H), 6.92 – 6.87 (m, 2H), 5.25 – 5.11 (m, 1H), 4.27 (ddd, *J* = 13.7, 6.8, 2.8 Hz, 1H), 3.81 (d, *J* = 1.1 Hz, 4H), 3.49 – 3.25 (m, 1H), 3.12 – 3.01 (m, 1H), 2.43 – 2.24 (m, 2H). **^{13}C NMR** (101 MHz, $CDCl_3$) (2 rotamers): δ (ppm) 158.7, 158.68, 152.1, 151.8, 151.3, 151.1, 134.9, 134.8, 129.5, 129.4, 128.3, 128.2, 125.7, 125.65, 125.3, 124.9, 121.8, 121.7, 114.3, 114.2, 107.9, 107.5, 55.4, 55.41, 48.8, 48.2, 37.9, 37.8, 29.6, 29.5.

HRMS (ESI): m/z calcd for $C_{19}H_{20}O_3N^+$ $[M + H]^+$ 310.1438 found 310.1457.

IR (ν_{max}/cm^{-1}) 1723, 1514, 1406, 1360, 1250, 1200, 1074, 1036, 828, 751, 722, 689.

SFC Conditions: Chiralpak IC; Gradient 1; 99:1 er (major enantiomer t_R = 4.92 min; minor enantiomer t_R = 5.08 min), **98% ee. $[\alpha]^{25}_{D}$ =** −45.9 (c = 2.0, $CHCl_3$).

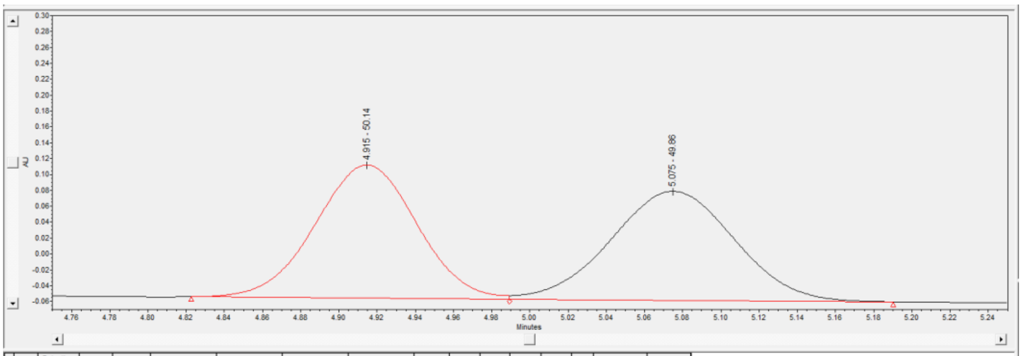

	Name	Retention Time (min)	Purity1 Angle	Purity1 Threshold	PDA/FLR Match1 Spect. Name	PDA/FLR Match1 Angle	PDA/FLR Match1 Threshold	PDA/FLR Match1 Lib. Name	Area (µV*sec)	% Area	Height (µV)	Int Type	Amount	Units	Peak Type	Peak Codes
1		4.915							622235	50.14	167557	bv			Unknown	
2		5.075							618677	49.86	137317	vb			Unknown	

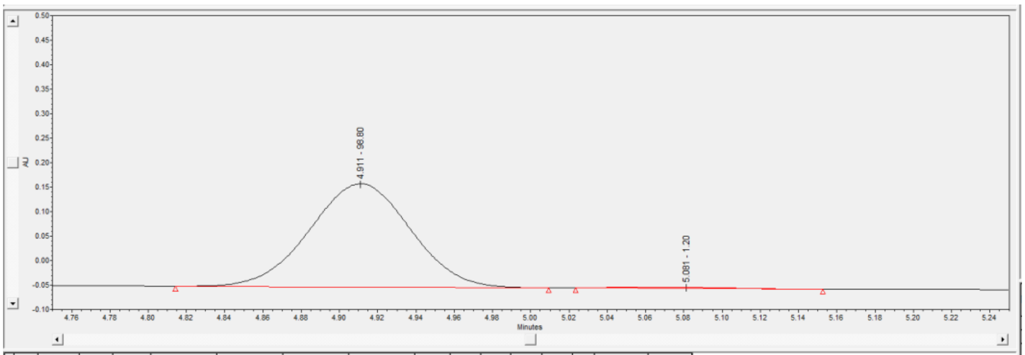

	Name	Retention Time (min)	Purity1 Angle	Purity1 Threshold	PDA/FLR Match1 Spect. Name	PDA/FLR Match1 Angle	PDA/FLR Match1 Threshold	PDA/FLR Match1 Lib. Name	Area (µV*sec)	% Area	Height (µV)	Int Type	Amount	Units	Peak Type	Peak Codes
1		4.911							774874	98.80	210772	bb			Unknown	
2		5.081							9438	1.20	2541	bb			Unknown	

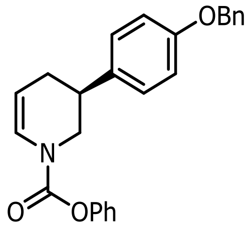

Phenyl-(S)-3-(4-(benzyloxy)phenyl)-3,4-dihydropyridine-1(2H)-carboxylate (3f): The corresponding compound was prepared following general procedure **A** using 4-(benzyloxy)phenyl boronic acid and phenyl pyridine-1(2H)-carboxylate. The mixture was stirred at 70 °C for 20 hours. Purification by flash chromatography (2% acetone/petrol to 20% acetone/petrol) afforded compound **3f** as white solid (47% yield, 99% ee).

^{1}H NMR (400 MHz, $CDCl_3$) (2 rotamers): δ (ppm) 7.48 – 7.29 (m, 7H), 7.27 – 6.91 (m, 8H), 5.25 – 5.10 (m, 1H), 5.07 (s, 3H), 4.41 – 4.24 (m, 1H), 3.46 – 3.24 (m, 1H), 3.12 – 2.96 (m, 1H), 2.43 – 2.24 (m, 2H). **^{13}C NMR** (101 MHz, $CDCl_3$) (2 rotamers): δ (ppm) 157.9, 152.1, 151.8, 151.3, 151.1, 137.2, 135.2, 135.1, 129.5, 129.4, 128.7, 128.3, 128.27, 128.1, 127.6, 127.56, 125.7, 125.6, 125.3, 124.9, 121.8, 121.7, 115.25, 115.19, 107.9, 107.5, 70.2, 48.8, 48.2, 37.9, 37.8, 29.6, 29.4.

HRMS (ESI): m/z calcd for $C_{25}H_{24}O_3N^+$ $[M + H]^+$ 386.1751 found 386.1761.

IR (ν_{max}/cm^{-1}) 1721, 1512, 1361, 1250, 1200, 1075, 1024, 827, 749, 691, 690.

SFC Conditions: Chiralpak IE; Gradient 1; 99.5:0.5 er (major enantiomer t_R = 6.97 min; minor enantiomer t_R = 6.78 min), **99% ee. [α]$^{25}_{D}$ =** −59.1 (c = 2.0, $CHCl_3$).

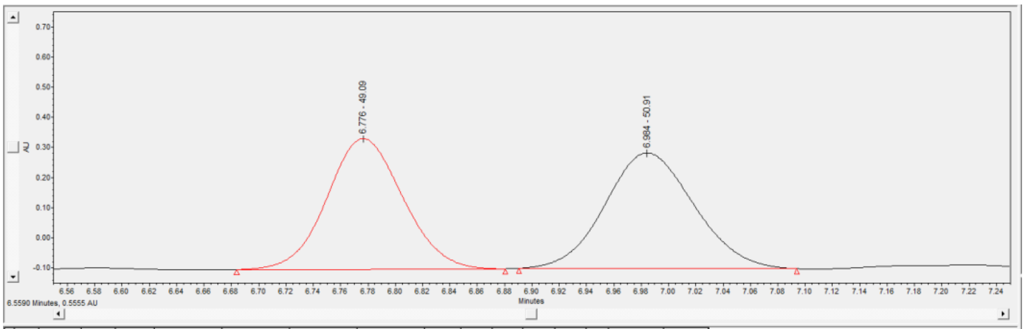

	Name	Retention Time (min)	Purity1 Angle	Purity1 Threshold	PDA/FLR Match1 Spect. Name	PDA/FLR Match1 Angle	PDA/FLR Match1 Threshold	PDA/FLR Match1 Lib. Name	Area (µV*sec)	% Area	Height (µV)	Int Type	Amount	Units	Peak Type	Peak Codes
1		6.776							1672972	49.09	433295	bb			Unknown	
2		6.984							1734931	50.91	382941	bb			Unknown	

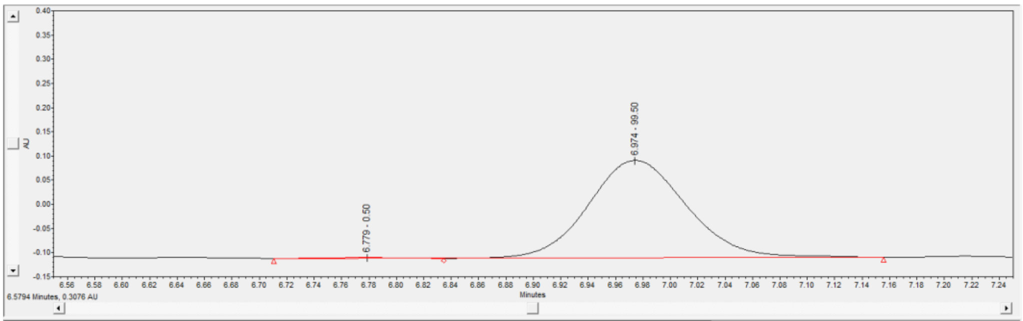

	Name	Retention Time (min)	Purity1 Angle	Purity1 Threshold	PDA/FLR Match1 Spect. Name	PDA/FLR Match1 Angle	PDA/FLR Match1 Threshold	PDA/FLR Match1 Lib. Name	Area (µV*sec)	% Area	Height (µV)	Int Type	Amount	Units	Peak Type	Peak Codes
1		6.779							5045	0.50	1603	bv			Unknown	
2		6.974							1001344	99.50	201285	vb			Unknown	

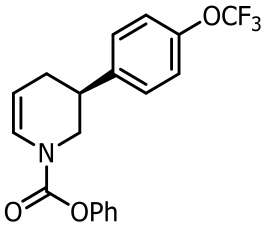

Phenyl-(S)-3-(4-(trifluoromethoxy)phenyl)-3,4-dihydropyridine-1(2H)-carboxlate (3g): The corresponding compound was prepared following general procedure **A** using 4-(trifluoromethoxy)phenyl boronic acid and phenyl pyridine-1(2H)-carboxylate. The mixture was stirred at 70 °C for 20 hours. Purification by flash chromatography (2% acetone/petrol to 20% acetone/petrol) afforded compound **3g** as viscous liquid (50% yield, 99% ee).

^{1}H NMR (500 MHz, $CDCl_3$) (2 rotamers): δ (ppm) 7.40 – 7.00 (m, 10H), 5.25 – 5.09 (m, 1H), 4.27 (t, *J* = 10.7 Hz, 1H), 3.56 – 3.28 (m, 1H), 3.13 (ddd, *J* = 15.6, 10.6, 6.0 Hz, 1H), 2.46 – 2.27 (m, 2H). **^{13}C NMR** (126 MHz, $CDCl_3$) (2 rotamers): δ (ppm) 152.1, 151.8, 151.2, 151.0, 148.3, 141.6, 141.4, 129.54, 129.5, 128.71, 128.7, 125.8, 125.5, 125.1, 121.8, 121.7, 121.5, 121.4, 107.5, 107.1, 48.4, 47.8, 38.1, 38.0, 29.4, 29.3. **^{19}F NMR** (376 MHz, $CDCl_3$) (2 rotamers): δ (ppm) −57.88, −57.90.

HRMS (ESI): m/z calcd for $C_{19}H_{17}O_3NF_3^+$ [M + H]$^+$ 364.1155 found 364.1162.

IR (ν_{max}/cm^{-1}) 1724, 1656, 1510, 1407, 1361, 1263, 1198, 1163, 1075, 846, 750, 689.

SFC Conditions: Chiralpak IA; Gradient 3; 93:7 er (major enantiomer t_R = 5.35 min; minor enantiomer t_R = 5.19 min), **86% ee. [α]$^{25}_{D}$ =** −39.7 (c = 2.0, $CHCl_3$).

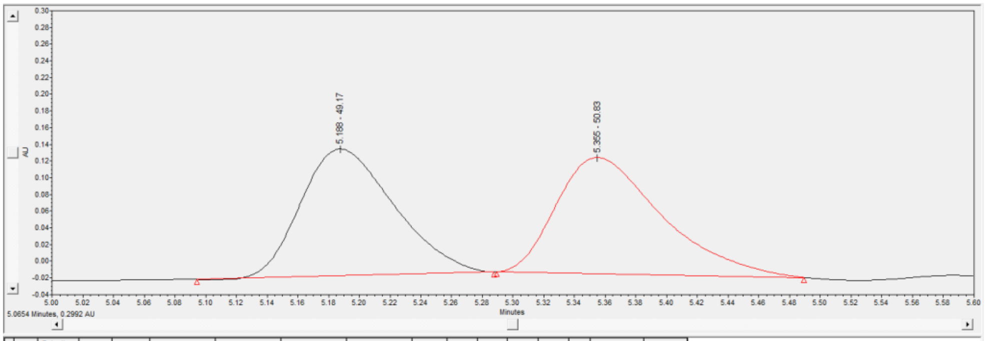

	Name	Retention Time (min)	Purity1 Angle	Purity1 Threshold	PDA/FLR Match1 Spect. Name	PDA/FLR Match1 Angle	PDA/FLR Match1 Threshold	PDA/FLR Match1 Lib. Name	Area (µV*sec)	% Area	Height (µV)	Int Type	Amount	Units	Peak Type	Peak Codes
1		5.188							640994	49.17	152008	bb			Unknown	
2		5.355							662750	50.83	139113	bb			Unknown	

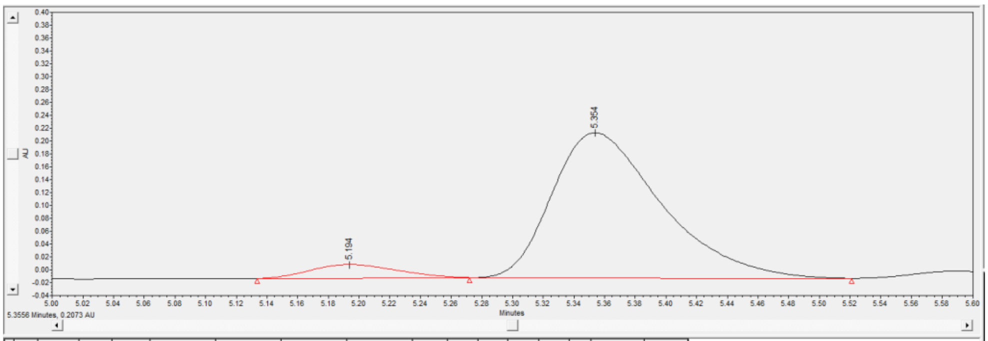

	Name	Retention Time (min)	Purity1 Angle	Purity1 Threshold	PDA/FLR Match1 Spect. Name	PDA/FLR Match1 Angle	PDA/FLR Match1 Threshold	PDA/FLR Match1 Lib. Name	Area (µV*sec)	% Area	Height (µV)	Int Type	Amount	Units	Peak Type	Peak Codes
1		5.194							82293	6.86	21115	bb			Unknown	
2		5.354							1116871	93.14	225486	bb			Unknown	

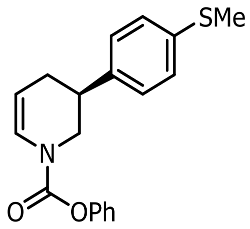

Phenyl-(S)-3-(4-(methylthio)phenyl)-3,4-dihydropyridine-1(2H)-carboxylate (3h): The corresponding compound was prepared following general procedure **A** using 4-(methylthio)phenyl boronic acid and phenyl pyridine-1(2H)-carboxylate. The mixture was stirred at 70 °C for 20 hours. Purification by flash chromatography (2% acetone/petrol to 15% acetone/petrol) afforded compound **3h** as viscous liquid (70% yield, 86% ee).

^{1}H NMR (400 MHz, $CDCl_3$) (2 rotamers): δ (ppm) 7.34 (dtd, J = 8.7, 7.2, 1.9 Hz, 2H), 7.25 – 6.90 (m, 8H), 5.24 – 5.04 (m, 1H), 4.32 – 4.14 (m, 1H), 3.51 – 3.17 (m, 1H), 3.13 – 2.93 (m, 1H), 2.46 (s, 3H), 2.37 – 2.22 (m, 2H). **^{13}C NMR** (101 MHz, $CDCl_3$) (2 rotamers): δ (ppm) 152.1, 151.8, 151.2, 151.1, 139.7, 139.6, 137.1, 137.0, 129.5, 129.4, 127.83, 127.80, 127.3, 127.2, 125.7, 125.68, 125.3, 124.9, 121.8, 121.7, 107.7, 107.4, 48.5, 47.9, 38.2, 38.0, 29.4, 29.2, 16.2, 16.1.

HRMS (ESI): m/z calcd for $C_{19}H_{20}O_2SN^+$ $[M + H]^+$ 326.1209 found 326.1198.

IR (ν_{max}/cm^{-1}) 1721, 1495, 1406, 1353, 1266, 1200, 1163, 1095, 1074, 973, 817, 748, 690.

SFC Conditions: Chiralpak IB; Gradient 1; 93:7 er (major enantiomer t_R = 4.93 min; minor enantiomer t_R = 4.52 min), **86% ee**. **$[\alpha]^{25}_D$ =** −77.3 (c = 2.0, $CHCl_3$).

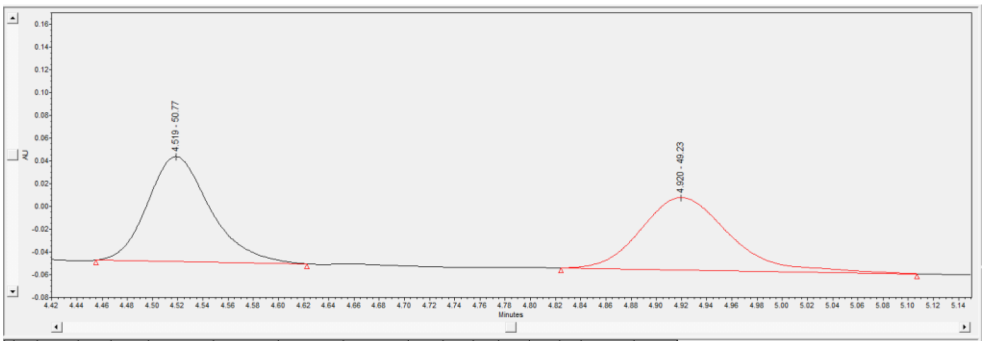

	Name	Retention Time (min)	Purity1 Angle	Purity1 Threshold	PDA/FLR Match1 Spect. Name	PDA/FLR Match1 Angle	PDA/FLR Match1 Threshold	PDA/FLR Match1 Lib. Name	Area (µV*sec)	% Area	Height (µV)	Int Type	Amount	Units	Peak Type	Peak Codes
1		4.519							318435	50.77	92288	bb			Unknown	
2		4.920							308730	49.23	63365	bb			Unknown	

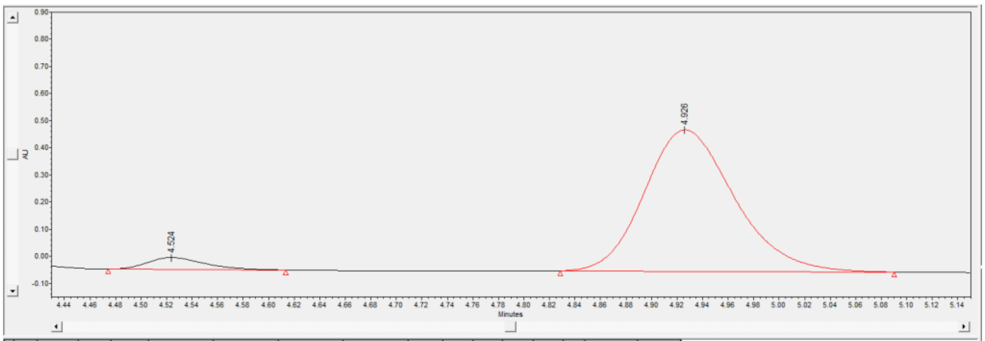

	Name	Retention Time (min)	Purity1 Angle	Purity1 Threshold	PDA/FLR Match1 Spect. Name	PDA/FLR Match1 Angle	PDA/FLR Match1 Threshold	PDA/FLR Match1 Lib. Name	Area (µV*sec)	% Area	Height (µV)	Int Type	Amount	Units	Peak Type	Peak Codes
1		4.524							135116	5.03	43513	bb			Unknown	
2		4.926							2550610	94.97	521898	bb			Unknown	

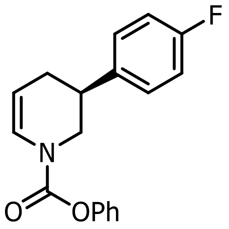

Phenyl-(S)-3-(4-fluorophenyl)-3,4-dihydropyridine-1(2H)-carboxylate (3i): The corresponding compound was prepared following general procedure **A** using 4-fluorophenyl boronic acid and phenyl pyridine-1(2H)-carboxylate. The mixture was stirred at 70 °C for 20 hours. Purification by flash chromatography (2% acetone/petrol to 12% acetone/petrol) afforded compound **3i** as viscous liquid (75% yield, 94% ee).

^{1}H NMR (500 MHz, $CDCl_3$) (2 rotamers): δ (ppm) 7.37 (dt, J = 10.8, 8.0 Hz, 2H), 7.25 – 6.98 (m, 8H), 5.25 – 5.09 (m, 1H), 4.26 (ddt, J = 16.4, 12.4, 2.3 Hz, 1H), 3.51 – 3.25 (m, 1H), 3.10 (dtt, J = 15.8, 10.5, 4.5 Hz, 1H), 2.43 – 2.25 (m, 2H). **^{13}C NMR** (126 MHz, $CDCl_3$) (^{19}F decoupled) (2 rotamers): δ (ppm) 163.1, 160.7, 152.1, 151.8, 151.2, 151.0, 138.5, 138.4, 129.5, 129.46, 128.8, 128.78, 128.74, 128.70, 125.7, 125.4, 125.0, 121.8, 121.7, 115.8, 115.76, 115.6, 115.5, 107.6, 107.3, 48.6, 48.0, 38.0, 37.9, 29.5, 29.4. **^{19}F NMR** (471 MHz, $CDCl_3$) (2 rotamers): δ (ppm) −115.8, −115.9.

HRMS (ESI): m/z calcd for $C_{18}H_{17}O_2FN^+$ $[M + H]^+$ 298.1238 found 298.1244.

IR (ν_{max}/cm^{-1}) 1724, 1511, 1406, 1361, 1200, 1162, 1074, 832, 753, 730, 689.

SFC Conditions: Chiralpak ID; Gradient 1; 97:3 er (major enantiomer t_R = 3.04 min; minor enantiomer t_R = 2.97 min), **94% ee. $[\alpha]^{25}_D$ =** −39.3 (c = 2.0, $CHCl_3$).

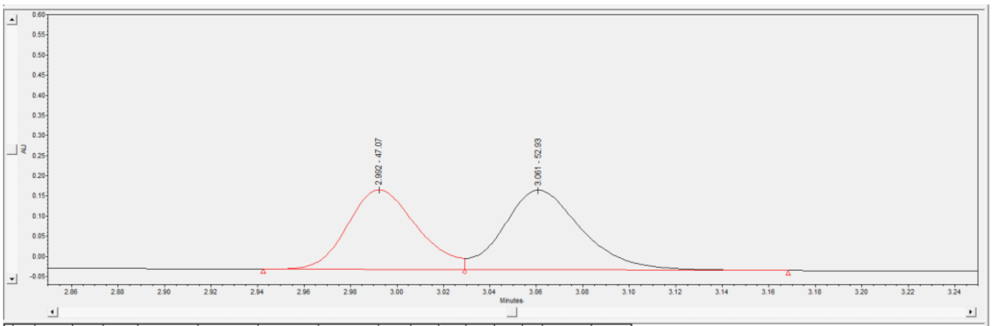

	Name	Retention Time (min)	Purity1 Angle	Purity1 Threshold	PDA/FLR Match1 Spect. Name	PDA/FLR Match1 Angle	PDA/FLR Match1 Threshold	PDA/FLR Match1 Lib. Name	Area (µV*sec)	% Area	Height (µV)	Int Type	Amount	Units	Peak Type	Peak Codes
1		2.992							408589	47.07	197622	bv			Unknown	
2		3.061							459491	52.93	197689	vb			Unknown	

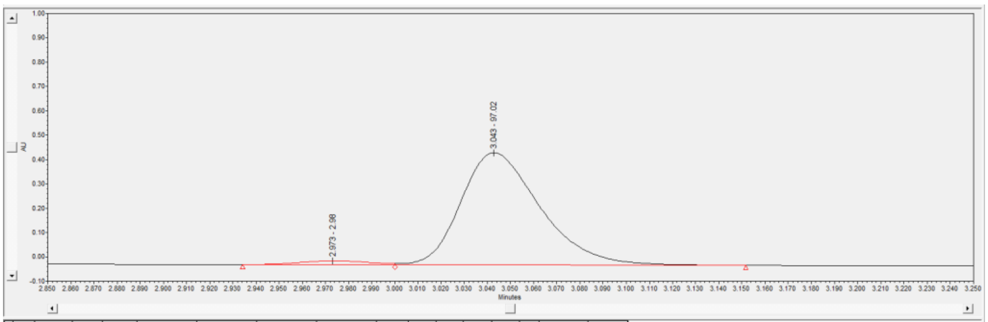

	Name	Retention Time (min)	Purity1 Angle	Purity1 Threshold	PDA/FLR Match1 Spect. Name	PDA/FLR Match1 Angle	PDA/FLR Match1 Threshold	PDA/FLR Match1 Lib. Name	Area (µV*sec)	% Area	Height (µV)	Int Type	Amount	Units	Peak Type	Peak Codes
1		2.973							33733	2.98	16202	bv			Unknown	
2		3.043							1098581	97.02	461350	vb			Unknown	

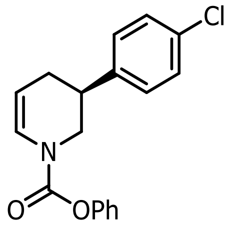

Phenyl-(S)-3-(4-chlorophenyl)-3,4-dihydropyridine-1(2H)-carboxylate (3j): The corresponding compound was prepared following general procedure **A** using 4-chlorophenyl boronic acid and phenyl pyridine-1(2H)-carboxylate. The mixture was stirred at 70 °C for 20 hours. Purification by flash chromatography (2% acetone/petrol to 20% acetone/petrol) afforded compound **3j** as viscous liquid (63% yield, 98% ee).

^{1}H NMR (400 MHz, $CDCl_3$) (2 rotamers): δ (ppm) 7.42 – 7.28 (m, 4H), 7.26 – 6.99 (m, 6H), 5.26 – 5.10 (m, 1H), 4.32 – 4.20 (m, 1H), 3.52 – 3.24 (m, 1H), 3.09 (qdd, J = 10.3, 7.6, 3.7 Hz, 1H), 2.44 – 2.23 (m, 2H). **^{13}C NMR** (101 MHz, $CDCl_3$) (2 rotamers): δ (ppm) 151.8, 151.2, 151.0, 141.3, 141.2, 132.9, 129.5, 129.48, 129.04, 129.0, 128.7, 128.67, 125.8, 125.5, 125.1, 121.8, 121.7, 107.5, 107.1, 48.4, 47.8, 38.1, 38.0, 29.4, 29.2.

HRMS (ESI): m/z calcd for $C_{18}H_{17}O_2ClN^+$ $[M + H]^+$ 314.0942 found 314.0937.

IR (ν_{max}/cm^{-1}) 1723, 1494, 1405, 1352, 1199, 1074, 821, 749, 689.

SFC Conditions: Chiralpak IA; Gradient 1; 99:1 er (major enantiomer t_R = 4.31 min; minor enantiomer t_R = 4.17 min), **98% ee**. **$[\alpha]^{25}_D$ =** −50.1 (c = 2.0, $CHCl_3$).

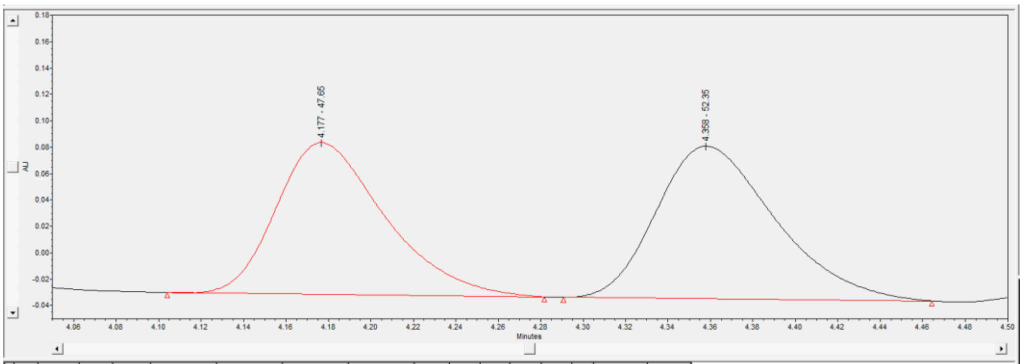

	Name	Retention Time (min)	Purity1 Angle	Purity1 Threshold	PDA/FLR Match1 Spect. Name	PDA/FLR Match1 Angle	PDA/FLR Match1 Threshold	PDA/FLR Match1 Lib. Name	Area (µV*sec)	% Area	Height (µV)	Int Type	Amount	Units	Peak Type	Peak Codes
1		4.177							408908	47.65	114932	bb			Unknown	
2		4.358							449283	52.35	115653	bb			Unknown	

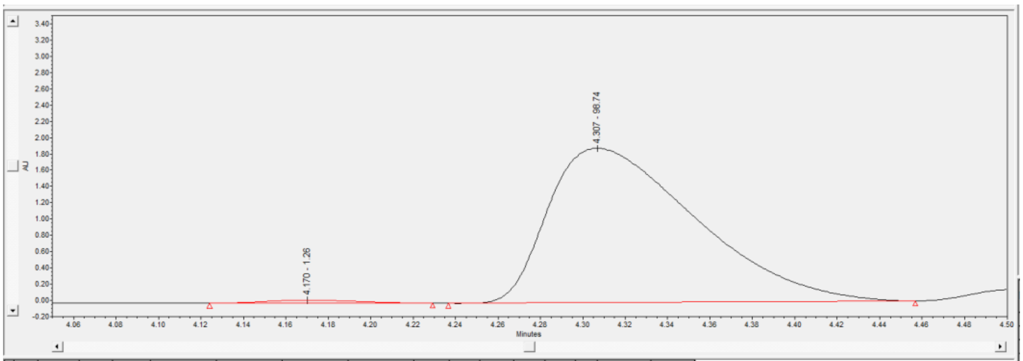

	Name	Retention Time (min)	Purity1 Angle	Purity1 Threshold	PDA/FLR Match1 Spect. Name	PDA/FLR Match1 Angle	PDA/FLR Match1 Threshold	PDA/FLR Match1 Lib. Name	Area (µV*sec)	% Area	Height (µV)	Int Type	Amount	Units	Peak Type	Peak Codes
1		4.170							116851	1.26	38792	bb			Unknown	
2		4.307							9175664	98.74	1900051	bb			Unknown	

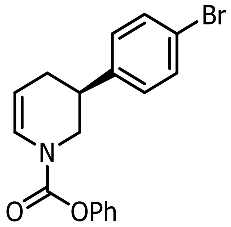

Phenyl-(S)-3-(4-bromophenyl)-3,4-dihydropyridine-1(2H)-carboxylate (3k): The corresponding compound was prepared following general procedure **A** using 4-bromophenyl boronic acid and phenyl pyridine-1(2H)-carboxylate. The mixture was stirred at 70 °C for 20 hours. Purification by flash chromatography (2% acetone/petrol to 20% acetone/petrol) afforded compound **3k** as white solid (60% yield, 98% ee).

^{1}H NMR (400 MHz, $CDCl_3$) (2 rotamers): δ (ppm) 7.51 – 7.32 (m, 5H), 7.25 – 7.00 (m, 5H), 5.25 – 5.08 (m, 1H), 4.37 – 4.15 (m, 1H), 3.52 – 3.24 (ddd, J = 70.1, 12.6, 11.0 Hz, 1H), 3.16 – 3.01 (m, 1H), 2.53 – 2.13 (m, 2H). **^{13}C NMR** (101 MHz, $CDCl_3$) (2 rotamers): δ (ppm) 152.0, 151.8, 151.2, 151.0, 141.8, 141.7, 132.0, 131.9, 129.5, 129.47, 129.1, 129.0, 125.8, 125.5, 125.1, 121.8, 121.7, 107.5, 107.1, 48.3, 47.7, 38.2, 38.1, 29.3, 29.2.

HRMS (ESI): m/z calcd for $C_{18}H_{17}O_2BrN^+$ [M + H]$^+$ 358.0437 found 358.0430.

IR (ν_{max}/cm^{-1}) 1721, 1492, 1405, 1361, 1254, 1199, 1072, 1009, 816, 749, 689, 689.

SFC Conditions: Chiralpak IE; Gradient 1; 99:1 er (major enantiomer t_R = 5.34 min; minor enantiomer t_R = 5.11 min), **98% ee. [α]^{25}$_D$ =** −60.1 (c = 2.0, $CHCl_3$).

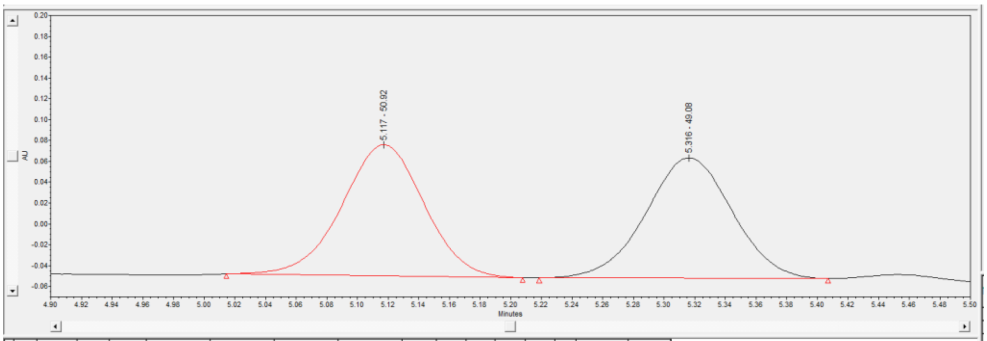

	Name	Retention Time (min)	Purity1 Angle	Purity1 Threshold	PDA/FLR Match1 Spect. Name	PDA/FLR Match1 Angle	PDA/FLR Match1 Threshold	PDA/FLR Match1 Lib. Name	Area (µV*sec)	% Area	Height (µV)	Int Type	Amount	Units	Peak Type	Peak Codes
1		5.117							469598	50.92	125919	bb			Unknown	
2		5.316							452586	49.08	115498	bb			Unknown	

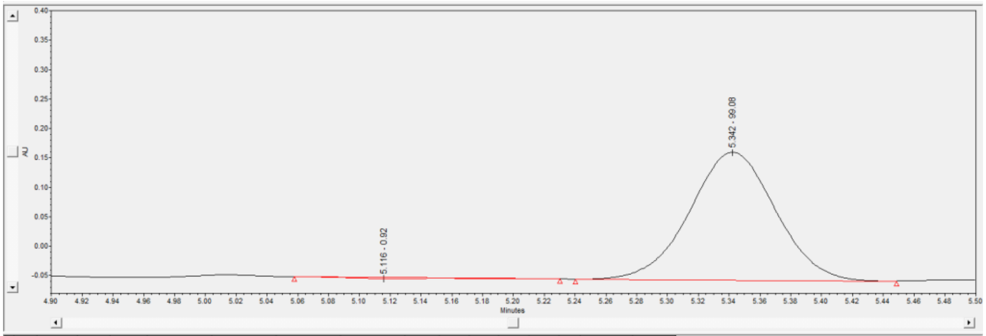

	Name	Retention Time (min)	Purity1 Angle	Purity1 Threshold	PDA/FLR Match1 Spect. Name	PDA/FLR Match1 Angle	PDA/FLR Match1 Threshold	PDA/FLR Match1 Lib. Name	Area (µV*sec)	% Area	Height (µV)	Int Type	Amount	Units	Peak Type	Peak Codes
1		5.116							7768	0.92	-1933	bb			Unknown	
2		5.342							836437	99.08	217007	bb			Unknown	

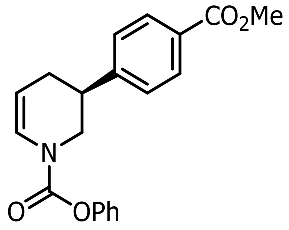

Phenyl-(S)-3-(4-(methoxycarbonyl)phenyl)-3,4-dihydropyridine-1(2H)-carboxylate (3l): The corresponding compound was prepared following general procedure **A** using 4-(methoxycarbonyl)phenyl boronic acid and phenyl pyridine-1(2H)-carboxylate. The mixture was stirred at 70 °C for 20 hours. Purification by flash chromatography (5% acetone/petrol to 20% acetone/petrol) afforded compound **3l** as viscous liquid (62% yield, 97% ee).

^{1}H NMR (500 MHz, $CDCl_3$) (2 rotamers): δ (ppm) 8.07 – 7.97 (m, 2H), 7.43 – 7.30 (m, 5H), 7.24 – 7.01 (m, 3H), 5.20 – 5.12 (m, 1H), 4.34 – 4.24 (m, 1H), 3.94 – 3.90 (m, 3H), 3.56 – 3.31 (m, 1H), 3.16 (ddt, J = 15.6, 10.0, 4.5 Hz, 1H), 2.45 – 2.29 (m, 2H). **^{13}C NMR** (126 MHz, $CDCl_3$) (2 rotamers): δ (ppm) 167.0, 152.1, 151.8, 151.2, 151.0, 148.0, 147.9, 130.2, 130.17, 129.5, 129.48, 129.1, 129.06, 127.4, 127.38, 125.8, 125.5, 125.1, 121.8, 121.7, 107.5, 107.1, 52.3, 52.2, 48.1, 47.5, 38.8, 38.6, 29.2, 29.1.

HRMS (ESI): m/z calcd for $C_{20}H_{20}O_4N^+$ $[M + H]^+$ 338.1387 found 338.1386.

IR (ν_{max}/cm^{-1}) 1721, 1406, 1360, 1281, 1200, 1112, 1075, 846, 751, 707, 690.

SFC Conditions: Chiralpak IC; Gradient 1; 98.5:1.5 er (major enantiomer t_R = 6.41 min; minor enantiomer t_R = 6.58 min), **97% ee**. **$[\alpha]^{25}_D$ =** −60.7 (c = 2.0, $CHCl_3$).

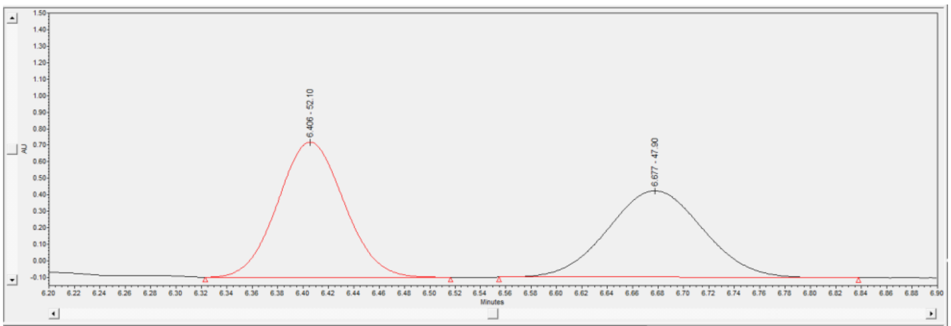

	Name	Retention Time (min)	Purity1 Angle	Purity1 Threshold	PDA/FLR Match1 Spect. Name	PDA/FLR Match1 Angle	PDA/FLR Match1 Threshold	PDA/FLR Match1 Lib. Name	Area (µV*sec)	% Area	Height (µV)	Int Type	Amount	Units	Peak Type	Peak Codes
1		6.406							2971098	52.10	817981	bb			Unknown	
2		6.677							2731854	47.90	523975	bb			Unknown	

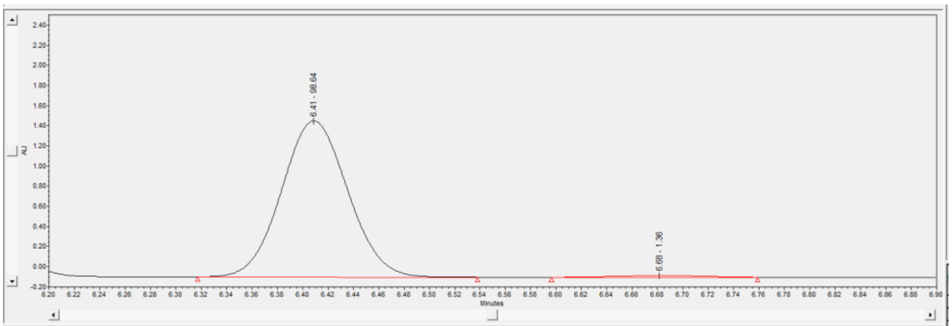

	Name	Retention Time (min)	Purity1 Angle	Purity1 Threshold	PDA/FLR Match1 Spect. Name	PDA/FLR Match1 Angle	PDA/FLR Match1 Threshold	PDA/FLR Match1 Lib. Name	Area (µV*sec)	% Area	Height (µV)	Int Type	Amount	Units	Peak Type	Peak Codes
1		6.409							5618867	98.64	1547372	bb			Unknown	
2		6.682							77630	1.36	16203	bb			Unknown	

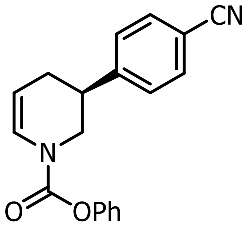

Phenyl-(S)-3-(4-cyanophenyl)-3,4-dihydropyridine-1(2H)-carboxylate (3m): The corresponding compound was prepared following general procedure **A** using 4-cyanophenyl boronic acid and phenyl pyridine-1(2H)-carboxylate. The mixture was stirred at 70 °C for 20 hours. Purification by flash chromatography (2% acetone/petrol to 25% acetone/petrol) afforded compound **3m** as viscous liquid (37% yield, 96% ee).

^{1}H NMR (500 MHz, $CDCl_3$) (2 rotamers): δ (ppm) 7.64 (dt, J = 8.1, 5.7 Hz, 2H), 7.37 (qd, J = 8.1, 4.4 Hz, 4H), 7.25 – 7.00 (m, 4H), 5.25 – 5.10 (m, 1H), 4.26 (dddd, J = 14.6, 12.6, 3.9, 1.7 Hz, 1H), 3.58 – 3.32 (m, 1H), 3.18 (qdd, J = 10.0, 7.5, 4.4 Hz, 1H), 2.50 – 2.25 (m, 2H). **^{13}C NMR** (126 MHz, $CDCl_3$) (2 rotamers): δ (ppm) 152.0, 151.7, 151.1, 150.9, 148.2, 148.1, 132.7, 132.68, 129.53, 129.5, 128.2, 128.18, 125.9, 125.6, 125.2, 121.7, 121.6, 118.8, 107.1, 106.7, 47.8, 47.2, 38.8, 38.6, 29.0, 28.9.

HRMS (ESI): m/z calcd for $C_{19}H_{17}O_2N_2^+$ [M + H]$^+$ 305.1285 found 305.1290.

IR (ν_{max}/cm^{-1}) 2229, 1720, 1408, 1343, 1266, 1200, 1164, 1073, 831, 752, 689.

SFC Conditions: Chiralpak IE; Gradient 1; 99.5:0.5 er (major enantiomer t_R = 6.16 min; minor enantiomer t_R = 6.07 min), **96% ee**. **[α]$^{25}_D$ =** −75.6 (c = 2.0, $CHCl_3$).

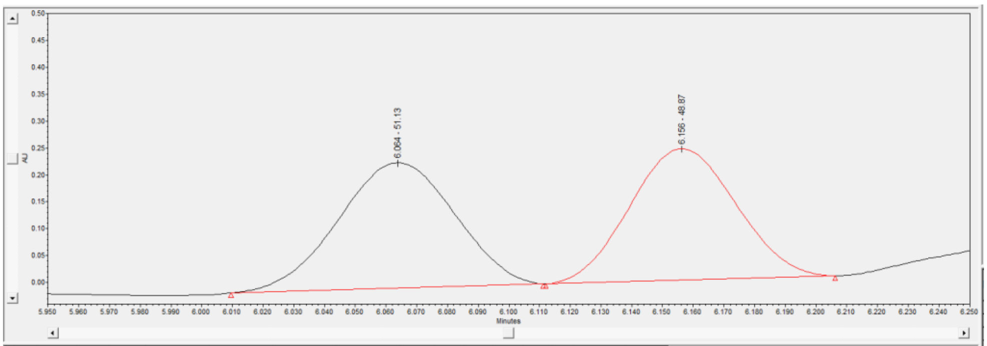

	Name	Retention Time (min)	Purity1 Angle	Purity1 Threshold	PDA/FLR Match1 Spect. Name	PDA/FLR Match1 Angle	PDA/FLR Match1 Threshold	PDA/FLR Match1 Lib. Name	Area (µV*sec)	% Area	Height (µV)	Int Type	Amount	Units	Peak Type	Peak Codes
1		6.064							611565	51.13	232776	bb			Unknown	
2		6.156							584470	48.87	243782	bb			Unknown	

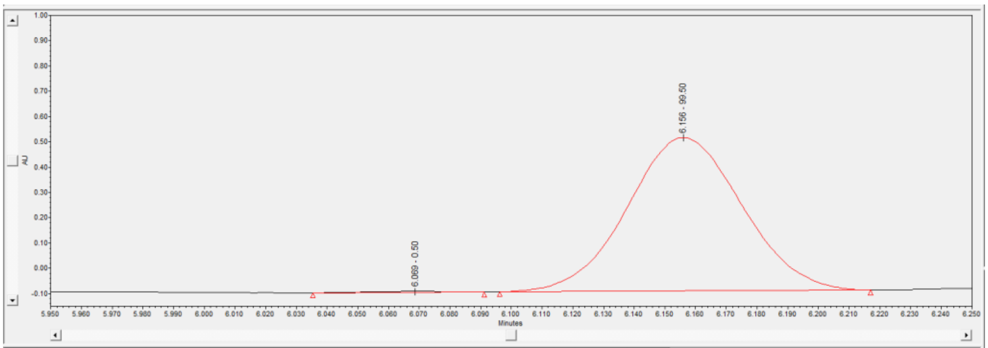

	Name	Retention Time (min)	Purity1 Angle	Purity1 Threshold	PDA/FLR Match1 Spect. Name	PDA/FLR Match1 Angle	PDA/FLR Match1 Threshold	PDA/FLR Match1 Lib. Name	Area (µV*sec)	% Area	Height (µV)	Int Type	Amount	Units	Peak Type	Peak Codes
1		6.069							7912	0.50	4186	bb			Unknown	I08
2		6.156							1572764	99.50	606937	bb			Unknown	

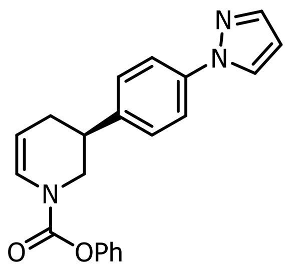

Phenyl-(S)-3-(4-(1H-pyrazol-1-yl)phenyl)-3,4-dihydropyridine-1(2H)-carboxylate (3n): The corresponding compound was prepared following general procedure **A** using 4-(1H-pyrazol-1-yl)phenyl boronic acid and phenyl pyridine-1(2H)-carboxylate. The mixture was stirred at 70 °C for 20 hours. Purification by flash chromatography (2% acetone/petrol to 25% acetone/petrol) afforded compound **3n** as viscous liquid (58% yield, 96% ee).

^{1}H NMR (500 MHz, $CDCl_3$) (2 rotamers): δ (ppm) 7.92 (d, *J* = 2.4 Hz, 1H), 7.73 (d, *J* = 2.5 Hz, 1H), 7.68 (dt, *J* = 8.3, 6.2 Hz, 2H), 7.40 – 7.31 (m, 4H), 7.25 – 7.01 (m, 4H), 6.47 (q, *J* = 2.4 Hz, 1H), 5.27 – 5.10 (m, 1H), 4.39 – 4.22 (m, 1H), 3.57 – 3.29 (m, 1H), 3.24 – 3.05 (m, 1H), 2.52 – 2.27 (m, 2H). **^{13}C NMR** (126 MHz, $CDCl_3$) (2 rotamers): δ (ppm) 152.1, 151.8, 151.2, 151.0, 141.2, 141.0, 140.9, 139.2, 129.5, 129.4, 128.3, 128.28, 126.8, 125.7, 125.4, 125.0, 121.8, 121.7, 119.6, 119.59, 107.7, 107.6, 107.2, 48.4, 47.8, 38.2, 38.0, 29.4, 29.3.

HRMS (ESI): m/z calcd for $C_{21}H_{20}O_2N_3^+$ $[M + H]^+$ 346.1550 found 346.1553.

IR (ν_{max}/cm^{-1}) 1717, 1526, 1396, 1335, 1254, 1200, 1164, 1074, 1047, 937, 833, 751, 690.

SFC Conditions: Chiralpak IF; Gradient 2; 98:2 er (major enantiomer t_R = 4.28 min; minor enantiomer t_R = 4.66 min), **96% ee**. **$[\alpha]^{25}_D$ =** −56.6 (c = 2.0, $CHCl_3$).

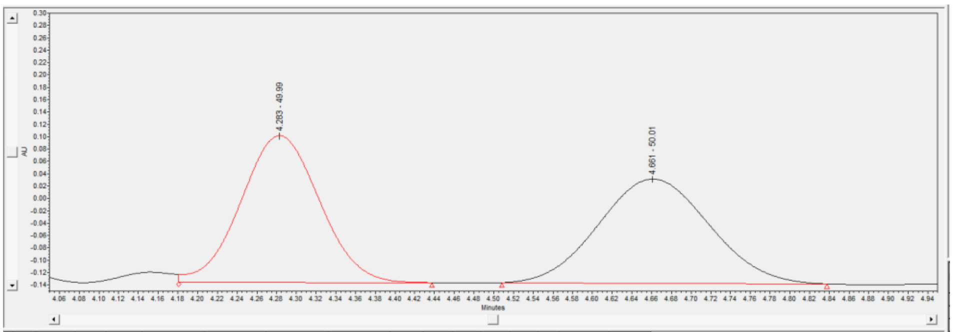

	Name	Retention Time (min)	Purity1 Angle	Purity1 Threshold	PDA/FLR Match1 Spect. Name	PDA/FLR Match1 Angle	PDA/FLR Match1 Threshold	PDA/FLR Match1 Lib. Name	Area (µV*sec)	% Area	Height (µV)	Int Type	Amount	Units	Peak Type	Peak Codes
1		4.283							1315804	49.99	237326	vb			Unknown	
2		4.661							1316593	50.01	168863	bb			Unknown	

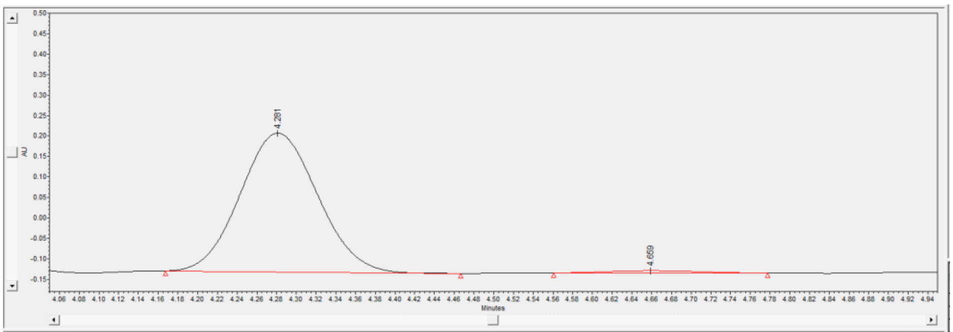

	Name	Retention Time (min)	Purity1 Angle	Purity1 Threshold	PDA/FLR Match1 Spect. Name	PDA/FLR Match1 Angle	PDA/FLR Match1 Threshold	PDA/FLR Match1 Lib. Name	Area (µV*sec)	% Area	Height (µV)	Int Type	Amount	Units	Peak Type	Peak Codes
1		4.281							1845302	98.00	338852	bb			Unknown	
2		4.659							37597	2.00	5589	bb			Unknown	

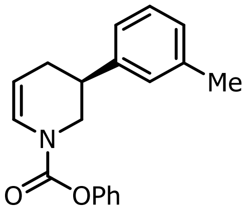

Phenyl-(S)-3-(m-tolyl)-3,4-dihydropyridine-1(2H)-carboxylate (3o): The corresponding compound was prepared following general procedure **A** using *m*-tolyl boronic acid and phenyl pyridine-1(2H)-carboxylate. The mixture was stirred at 70 °C for 20 hours. Purification by flash chromatography (2% acetone/petrol to 25% acetone/petrol) afforded compound **3o** as viscous liquid (70% yield, 96% ee).

^{1}H NMR (400 MHz, $CDCl_3$) (2 rotamers): δ (ppm) 7.44 – 7.31 (m, 2H), 7.31 – 6.98 (m, 8H), 5.26 – 5.10 (m, 1H), 4.37 – 4.25 (m, 1H), 3.53 – 3.18 (m, 1H), 3.06 (dddd, *J* = 15.2, 10.6, 8.8, 3.7 Hz, 1H), 2.41 – 2.29 (m, 5H). **^{13}C NMR** (101 MHz, $CDCl_3$) (2 rotamers): δ (ppm) 152.1, 151.7, 151.2, 151.1, 142.8, 142.6, 138.5, 138.4, 129.5, 129.4, 128.8, 128.7, 128.1, 127.9, 127.8, 125.7, 125.6, 125.2, 124.9, 124.3, 124.3, 121.8, 121.7, 107.9, 107.5, 48.6, 48.0, 38.7, 38.5, 29.5, 29.4, 21.6.

HRMS (ESI): m/z calcd for $C_{19}H_{20}O_2N^+$ $[M + H]^+$ 294.1489 found 294.1496.

IR (ν_{max}/cm^{-1}) 1724, 1406, 1360, 1203, 1074, 786, 750, 725, 704, 689.

SFC Conditions: Chiralpak IC; Gradient 1; 98:2 er (major enantiomer t_R = 4.01 min; minor enantiomer t_R = 4.22 min), **96% ee. [α]$^{25}_{D}$ =** −52.3 (c = 2.0, $CHCl_3$).

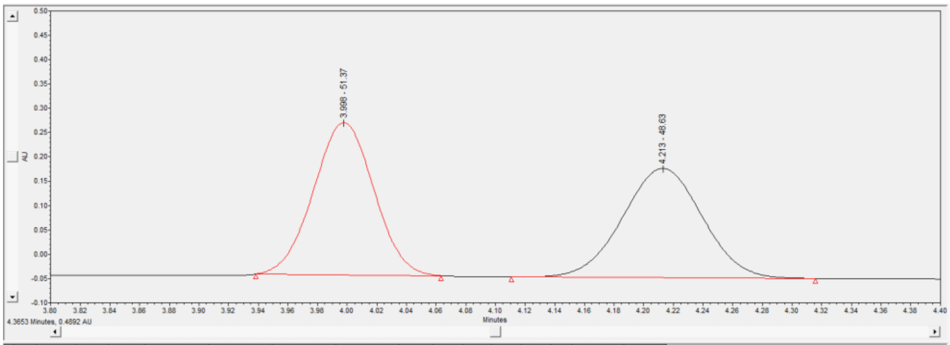

	Name	Retention Time (min)	Purity1 Angle	Purity1 Threshold	PDA/FLR Match1 Spect. Name	PDA/FLR Match1 Angle	PDA/FLR Match1 Threshold	PDA/FLR Match1 Lib. Name	Area (μV*sec)	% Area	Height (μV)	Int Type	Amount	Units	Peak Type	Peak Codes
1		3.998							886426	51.37	313151	bb			Unknown	
2		4.213							839173	48.63	224244	bb			Unknown	

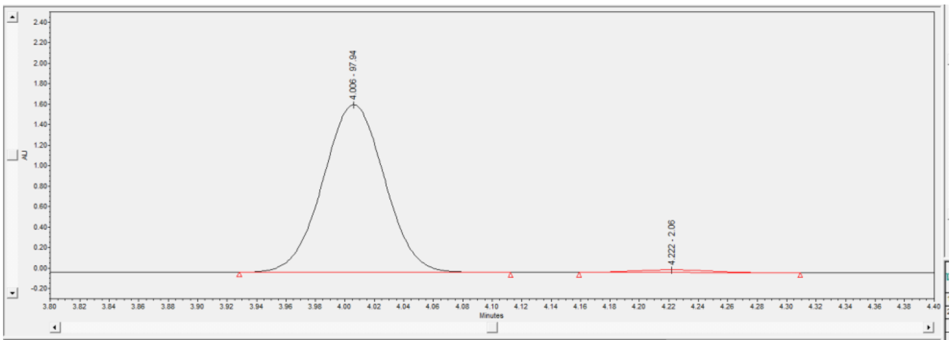

	Name	Retention Time (min)	Purity1 Angle	Purity1 Threshold	PDA/FLR Match1 Spect. Name	PDA/FLR Match1 Angle	PDA/FLR Match1 Threshold	PDA/FLR Match1 Lib. Name	Area (μV*sec)	% Area	Height (μV)	Int Type	Amount	Units	Peak Type	Peak Codes
1		4.006							4748512	97.94	1637413	bb			Unknown	
2		4.222							100095	2.06	27863	bb			Unknown	

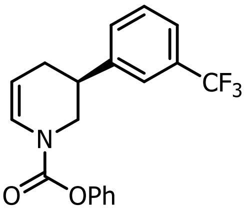

Phenyl-(S)-3-(3-(trifluoromethyl)phenyl)-3,4-dihydropyridine-1(2H)-carboxylate (3p): The corresponding compound was prepared following general procedure **A** using 3-(trifluoromethyl)phenyl boronic acid and phenyl pyridine-1(2H)-carboxylate. The mixture was stirred at 70 °C for 20 hours. Purification by flash chromatography (2% acetone/petrol to 20% acetone/petrol) afforded compound **3p** as viscous liquid (60% yield, 97% ee).

^{1}H NMR (500 MHz, $CDCl_3$) (2 rotamers): δ (ppm) 7.57 – 7.44 (m, 5H), 7.38 (dt, J = 11.3, 8.0 Hz, 2H), 7.26 – 7.02 (m, 4H), 5.28 – 5.11 (m, 1H), 4.36 – 4.25 (m, 1H), 3.59 – 3.30 (m, 1H), 3.19 (dddd, J = 20.1, 10.2, 7.7, 3.7 Hz, 1H), 2.48 – 2.31 (m, 2H). **^{13}C NMR** (126 MHz, $CDCl_3$) (2 rotamers): δ (ppm) 152.0, 151.8, 151.2, 151.0, 143.7, 143.6, 130.8, 129.5, 129.48, 129.4, 129.3, 125.8, 125.5, 125.2, 124.1, 124.07, 121.8, 121.7, 107.3, 107.0, 48.2, 47.6, 38.6, 38.4, 29.3, 29.2. **^{19}F NMR** (471 MHz, $CDCl_3$) (2 rotamers): δ (ppm) −62.5, −62.6.

HRMS (ESI): m/z calcd for $C_{19}H_{16}O_2F_3N^+$ $[M + H]^+$ 348.1206 found 348.1210.

IR (ν_{max}/cm^{-1}) 1724, 1408, 1331, 1257, 1202, 1164, 1124, 1074, 803, 752, 703, 689.

SFC Conditions: Chiralpak IA; Gradient 1; 98.5:1.5 er (major enantiomer t_R = 2.45 min; minor enantiomer t_R = 2.38 min), **97% ee. $[\alpha]^{25}_D$ =** −39.4 (c = 2.0, $CHCl_3$).

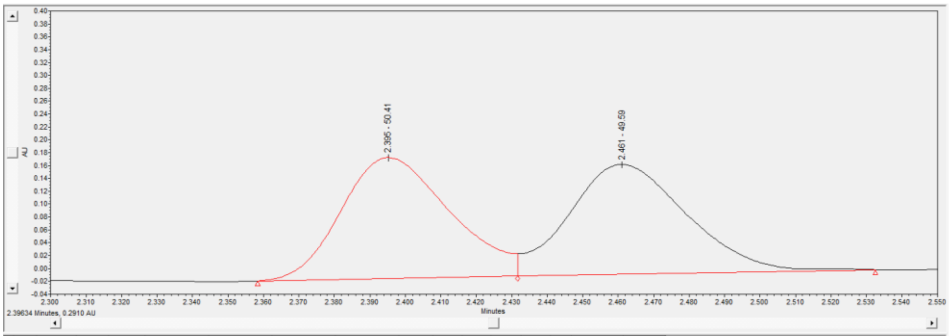

	Name	Retention Time (min)	Purity1 Angle	Purity1 Threshold	PDA/FLR Match1 Spect. Name	PDA/FLR Match1 Angle	PDA/FLR Match1 Threshold	PDA/FLR Match1 Lib. Name	Area (µV*sec)	% Area	Height (µV)	Int Type	Amount	Units	Peak Type	Peak Codes
1		2.395							400050	50.41	188149	bv			Unknown	
2		2.461							393552	49.59	170839	vb			Unknown	

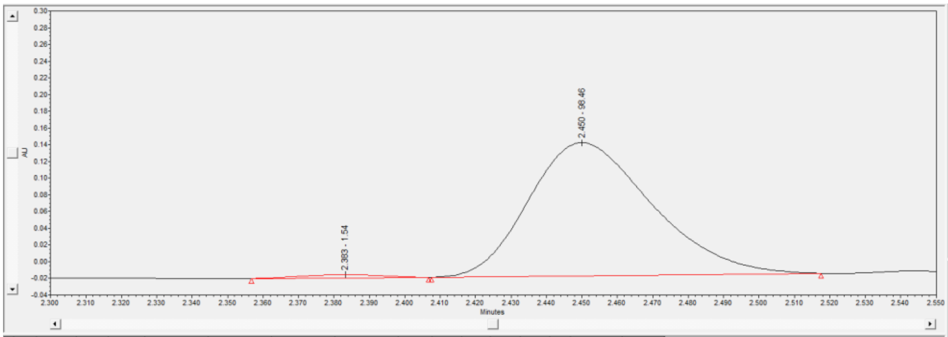

	Name	Retention Time (min)	Purity1 Angle	Purity1 Threshold	PDA/FLR Match1 Spect. Name	PDA/FLR Match1 Angle	PDA/FLR Match1 Threshold	PDA/FLR Match1 Lib. Name	Area (µV*sec)	% Area	Height (µV)	Int Type	Amount	Units	Peak Type	Peak Codes
1		2.383							6021	1.54	3830	bb			Unknown	
2		2.450							384722	98.46	159410	bb			Unknown	

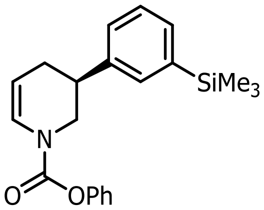

Phenyl-(S)-3-(3-(trimethylsilyl)phenyl)-3,4-dihydropyridine-1(2H)-carboxylate (3q): The corresponding compound was prepared following general procedure **A** using 3-(trimethylsilyl)phenyl boronic acid and phenyl pyridine-1(2H)-carboxylate. The mixture was stirred at 70 °C for 20 hours. Purification by flash chromatography (2% acetone/petrol to 10% acetone/petrol) afforded compound **3q** as viscous liquid (70% yield, 96% ee).

^{1}H NMR (500 MHz, $CDCl_3$) (2 rotamers): δ (ppm) 7.49 – 7.34 (m, 5H), 7.29 – 7.03 (m, 5H), 5.29 – 5.13 (m, 1H), 4.39 – 4.28 (m, 1H), 3.57 – 3.29 (m, 1H), 3.13 (pd, *J* = 11.8, 3.8 Hz, 1H), 2.46 – 2.31 (m, 2H), 0.34 – 0.28 (m, 9H). **^{13}C NMR** (101 MHz, $CDCl_3$) (2 rotamers): δ (ppm) 152.1, 151.8, 151.2, 151.1, 142.0, 141.9, 141.3, 141.2, 132.4, 132.4, 132.2, 132.1, 129.5, 128.3, 128.2, 127.7, 127.6, 125.7, 125.6, 125.3, 124.9, 121.8, 121.7, 107.9, 107.6, 48.6, 48.0, 38.9, 38.8, 29.5, 29.49, -1.0.

HRMS (ESI): m/z calcd for $C_{21}H_{26}O_2NSi^+$ $[M + H]^+$ 352.1727 found 352.1734.

IR (ν_{max}/cm^{-1}) 1727, 1656, 1405, 1358, 1249, 1202, 858, 839, 751, 689, 690.

SFC Conditions: Chiralpak IG; Gradient 1; 98:2 er (major enantiomer t_R = 3.00 min; minor enantiomer t_R = 2.84 min), **96% ee. [α]$^{25}_D$ =** −22.7 (c = 2.0, $CHCl_3$).

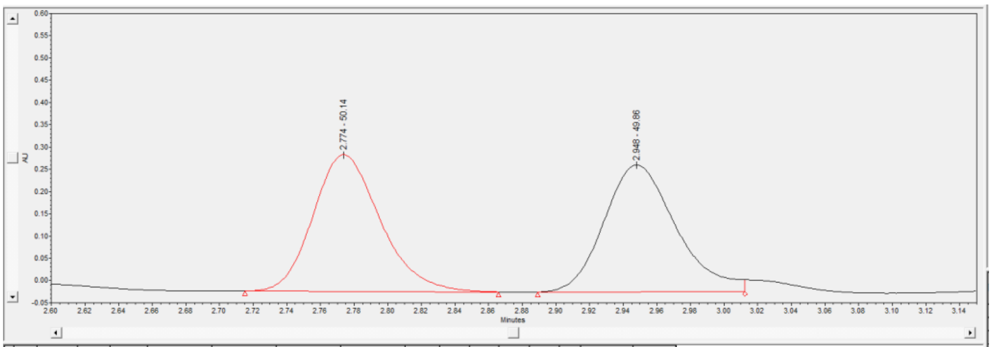

	Name	Retention Time (min)	Purity1 Angle	Purity1 Threshold	PDA/FLR Match1 Spect. Name	PDA/FLR Match1 Angle	PDA/FLR Match1 Threshold	PDA/FLR Match1 Lib. Name	Area (µV*sec)	% Area	Height (µV)	Int Type	Amount	Units	Peak Type	Peak Codes
1		2.774							852619	50.14	307172	bb			Unknown	
2		2.948							847934	49.86	285157	bv			Unknown	

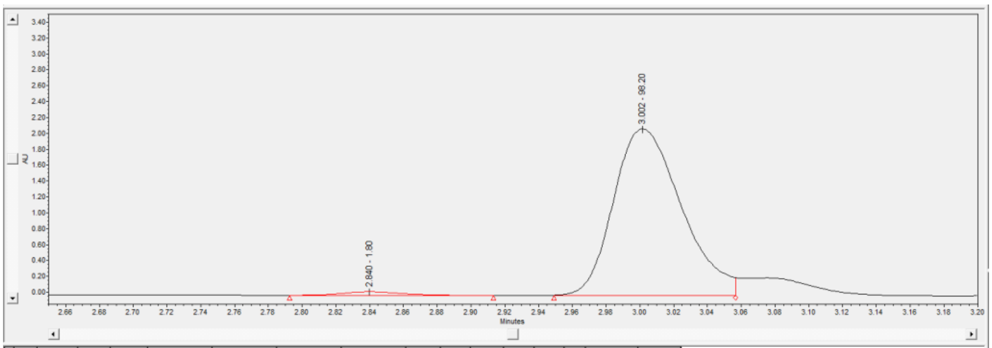

	Name	Retention Time (min)	Purity1 Angle	Purity1 Threshold	PDA/FLR Match1 Spect. Name	PDA/FLR Match1 Angle	PDA/FLR Match1 Threshold	PDA/FLR Match1 Lib. Name	Area (µV*sec)	% Area	Height (µV)	Int Type	Amount	Units	Peak Type	Peak Codes
1		2.840							107104	1.80	44139	bb			Unknown	
2		3.002							5833850	98.20	2092035	bv			Unknown	

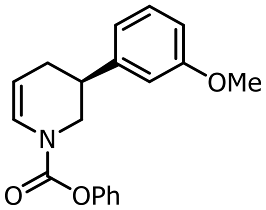

Phenyl-(S)-3-(3-methoxyphenyl)-3,4-dihydropyridine-1(2H)-carboxylate (3r): The corresponding compound was prepared following general procedure **A** using 3-methoxyphenyl boronic acid and phenyl pyridine-1(2H)-carboxylate. The mixture was stirred at 70 °C for 20 hours. Purification by flash chromatography (2% acetone/petrol to 20% acetone/petrol) afforded compound **3r** as viscous liquid (79% yield, 98% ee).

^{1}H NMR (400 MHz, $CDCl_3$) (2 rotamers): δ (ppm) 7.42 – 7.33 (m, 2H), 7.32 – 6.99 (m, 5H), 6.90 – 6.78 (m, 3H), 5.26 – 5.10 (m, 1H), 4.32 (ddd, J = 11.5, 9.7, 3.7 Hz, 1H), 3.85 – 3.72 (m, 3H), 3.53 – 3.26 (m, 1H), 3.08 (qdd, J = 10.2, 6.1, 3.8 Hz, 1H), 2.45 – 2.27 (m, 2H). **^{13}C NMR** (126 MHz, $CDCl_3$) (2 rotamers): δ (ppm) 160.0, 159.96, 152.1, 151.7, 151.2, 151.0, 144.5, 144.3, 129.9, 129.8, 129.5, 129.4, 125.7, 125.6, 125.3, 124.9, 121.8, 121.7, 119.6, 113.5, 113.3, 112.1, 112.08, 107.8, 107.4, 55.32, 55.3, 48.5, 47.9, 38.8, 38.6, 29.4, 29.3.

HRMS (ESI): m/z calcd for $C_{19}H_{20}O_3N^+$ $[M + H]^+$ 310.1438 found 310.1441.

IR (ν_{max}/cm^{-1}) 1723, 1655, 1600, 1494, 1406, 1376, 1360, 1266, 1204, 784, 751.

SFC Conditions: Chiralpak IC; Gradient 1; 99:1 er (major enantiomer t_R = 4.65 min; minor enantiomer t_R = 4.89 min), **98% ee. [α]$^{25}_D$ =** −29.8 (c = 2.0, $CHCl_3$).

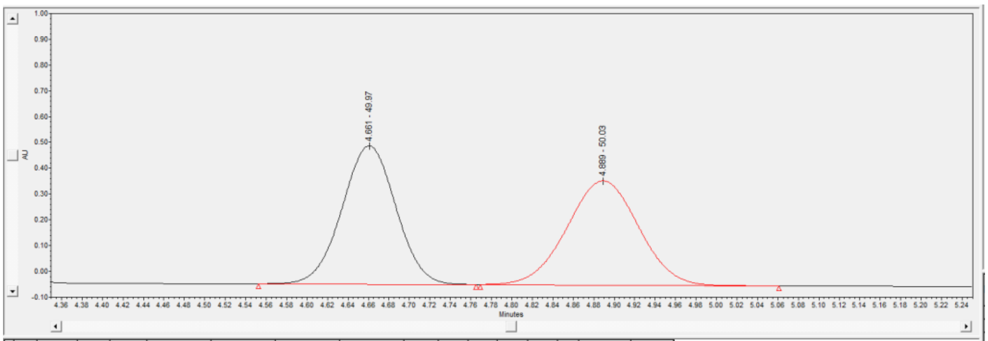

	Name	Retention Time (min)	Purity1 Angle	Purity1 Threshold	PDA/FLR Match1 Spect. Name	PDA/FLR Match1 Angle	PDA/FLR Match1 Threshold	PDA/FLR Match1 Lib. Name	Area (µV*sec)	% Area	Height (µV)	Int Type	Amount	Units	Peak Type	Peak Codes
1		4.661							1967215	49.97	535996	bb			Unknown	
2		4.889							1969204	50.03	404262	bb			Unknown	

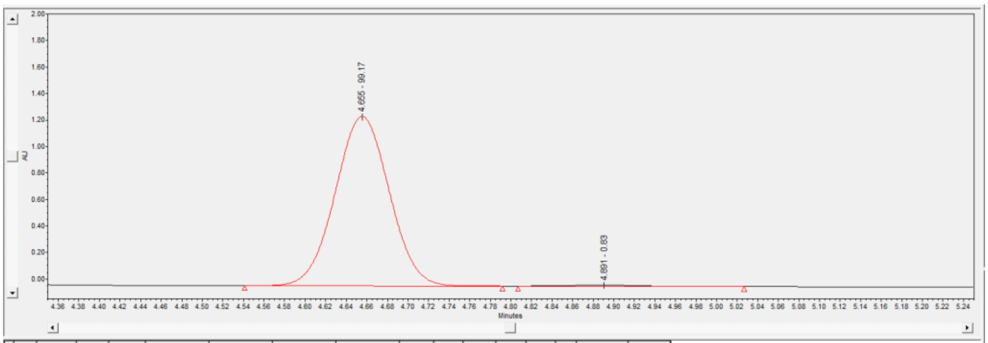

	Name	Retention Time (min)	Purity1 Angle	Purity1 Threshold	PDA/FLR Match1 Spect. Name	PDA/FLR Match1 Angle	PDA/FLR Match1 Threshold	PDA/FLR Match1 Lib. Name	Area (µV*sec)	% Area	Height (µV)	Int Type	Amount	Units	Peak Type	Peak Codes
1		4.655							4674488	99.17	1278233	bb			Unknown	
2		4.891							38957	0.83	8584	bb			Unknown	

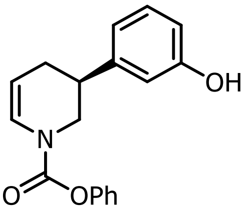

Phenyl-(S)-3-(3-hydroxyphenyl)-3,4-dihydropyridine-1(2H)-carboxylate (3s): The corresponding compound was prepared following general procedure **A** using 3-hydroxyphenyl boronic acid and phenyl pyridine-1(2H)-carboxylate. The mixture was stirred at 70 °C for 20 hours. Purification by flash chromatography (5% acetone/petrol to 30% acetone/petrol) afforded compound **3s** as viscous liquid (41% yield, 83% ee).

^{1}H NMR (400 MHz, $CDCl_3$) (2 rotamers): δ (ppm) 7.36 (dtd, *J* = 9.1, 7.4, 1.9 Hz, 2H), 7.26 – 6.95 (m, 5H), 6.82 (ddt, *J* = 10.5, 7.4, 1.4 Hz, 1H), 6.74 – 6.67 (m, 2H), 5.25 – 5.09 (m, 2H), 4.34 – 4.22 (m, 1H), 3.51 – 3.22 (m, 1H), 3.12 – 2.93 (m, 1H), 2.42 – 2.22 (m, 2H). **^{13}C NMR** (101 MHz, $CDCl_3$) (2 rotamers): δ (ppm) 156.2, 156.1, 152.3, 151.9, 151.2, 151.0, 144.7, 144.5, 130.05, 130.0, 129.5, 129.49, 125.81, 125.8, 125.2, 124.9, 121.8, 121.7, 119.62, 119.6, 114.30, 114.29, 114.1, 114.06, 108.1, 107.7, 48.5, 47.9, 38.6, 38.4, 29.4, 29.2.

HRMS (ESI): m/z calcd for $C_{18}H_{18}O_3N^+$ $[M + H]^+$ 296.1281 found 296.1280.

IR (ν_{max}/cm^{-1}) 3393, 1699, 1656, 1591, 1494, 1456, 1409, 1361, 1203, 787, 753.

SFC Conditions: Chiralpak IE; Gradient 1; 91.5:8.5 er (major enantiomer t_R = 5.35 min; minor enantiomer t_R = 5.12 min), **83% ee. $[\alpha]^{25}_{D}$ =** −37.3 (c = 2.0, $CHCl_3$).

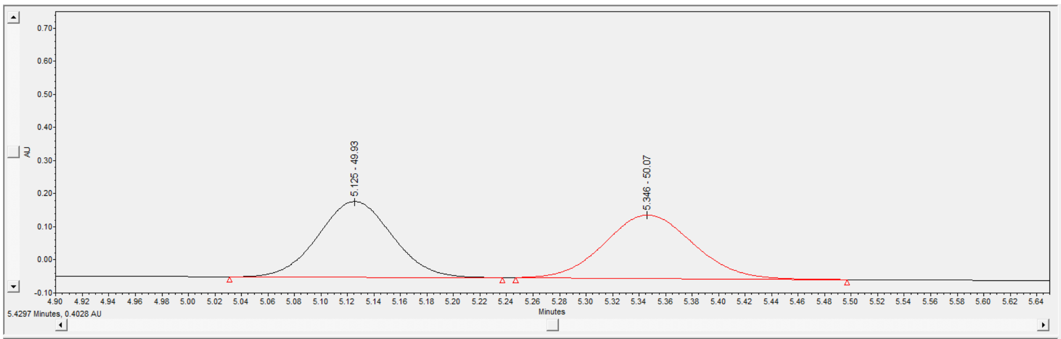

	Name	Retention Time (min)	Purity1 Angle	Purity1 Threshold	PDA/FLR Match1 Spect. Name	PDA/FLR Match1 Angle	PDA/FLR Match1 Threshold	PDA/FLR Match1 Lib. Name	Area (µV*sec)	% Area	Height (µV)	Int Type	Amount	Units	Peak Type	Peak Codes
1		5.125							886610	49.93	228470	bb			Unknown	
2		5.346							889258	50.07	191185	bb			Unknown	

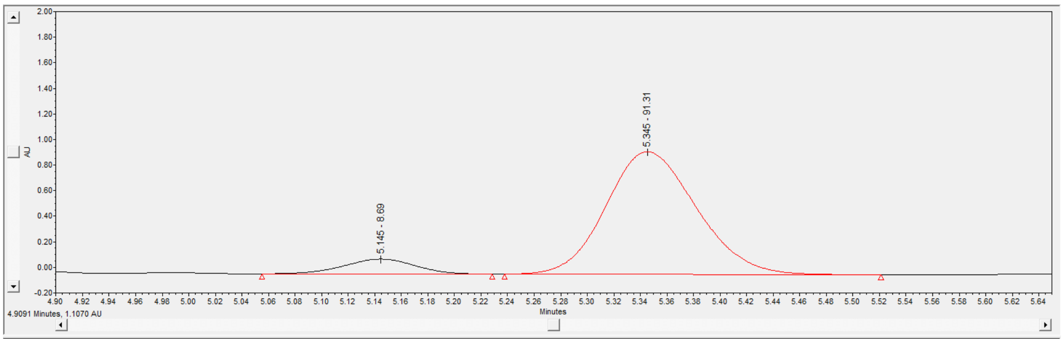

	Name	Retention Time (min)	Purity1 Angle	Purity1 Threshold	PDA/FLR Match1 Spect. Name	PDA/FLR Match1 Angle	PDA/FLR Match1 Threshold	PDA/FLR Match1 Lib. Name	Area (µV*sec)	% Area	Height (µV)	Int Type	Amount	Units	Peak Type	Peak Codes
1		5.145							432694	8.69	116161	bb			Unknown	
2		5.345							4544308	91.31	958081	bb			Unknown	

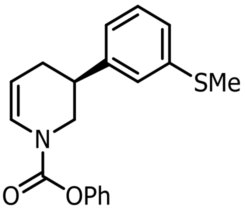

Phenyl-(S)-3-(3-(methylthio)phenyl)-3,4-dihydropyridine-1(2H)-carboxylate(3t): The corresponding compound was prepared following general procedure **A** using 3-(methylthio)phenyl boronic acid and phenyl pyridine-1(2H)-carboxylate. The mixture was stirred at 70 °C for 20 hours. Purification by flash chromatography (2% acetone/petrol to 25% acetone/petrol) afforded compound **3t** as viscous liquid (70% yield, 99% ee).

^{1}H NMR (500 MHz, $CDCl_3$) (2 rotamers): δ (ppm) 7.42 – 7.33 (m, 2H), 7.31 – 7.00 (m, 8H), 5.25 – 5.09 (m, 1H), 4.35 – 4.25 (m, 1H), 3.52 – 3.26 (m, 1H), 3.53 – 3.25 (m, 1H), 2.51 – 2.47 (m, 3H), 2.43 – 2.26 (m, 2H). **^{13}C NMR** (126 MHz, $CDCl_3$) (2 rotamers): δ (ppm) 152.1, 151.7, 151.2, 151.0, 143.5, 143.4, 139.1, 139.0, 129.5, 129.4, 129.36, 129.3, 125.7, 125.69, 125.64, 125.6, 125.3, 125.2, 125.15, 125.0, 124.1, 121.8, 121.7, 107.7, 107.3, 48.4, 47.8, 38.7, 38.6, 29.4, 29.3, 15.9.

HRMS (ESI): m/z calcd for $C_{19}H_{20}O_2SN^+$ $[M + H]^+$ 326.1209 found 326.1211.

IR (ν_{max}/cm^{-1}) 1722, 1406, 1359, 1254, 1201, 1074, 786, 748, 690.

SFC Conditions: Chiralpak IC; Gradient 1; 99.5:0.5 er (major enantiomer t_R = 5.11 min; minor enantiomer t_R = 5.30 min), **99% ee. $[\alpha]^{25}_D$ =** −40.2 (c = 2.0, $CHCl_3$).

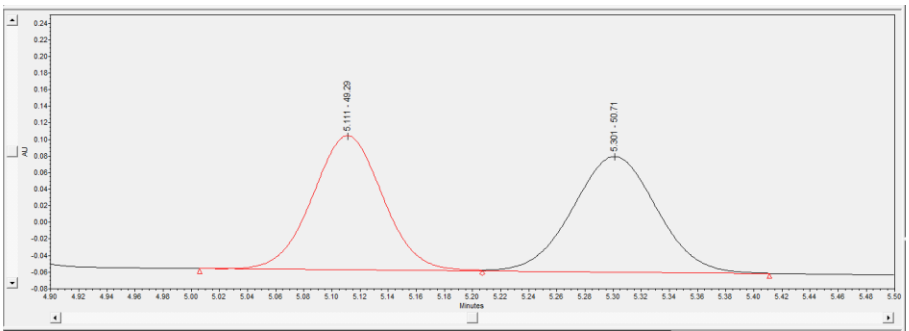

	Name	Retention Time (min)	Purity1 Angle	Purity1 Threshold	PDA/FLR Match1 Spect. Name	PDA/FLR Match1 Angle	PDA/FLR Match1 Threshold	PDA/FLR Match1 Lib. Name	Area (µV*sec)	% Area	Height (µV)	Int Type	Amount	Units	Peak Type	Peak Codes
1		5.111							562680	49.29	161511	bv			Unknown	
2		5.301							578998	50.71	139270	vb			Unknown	

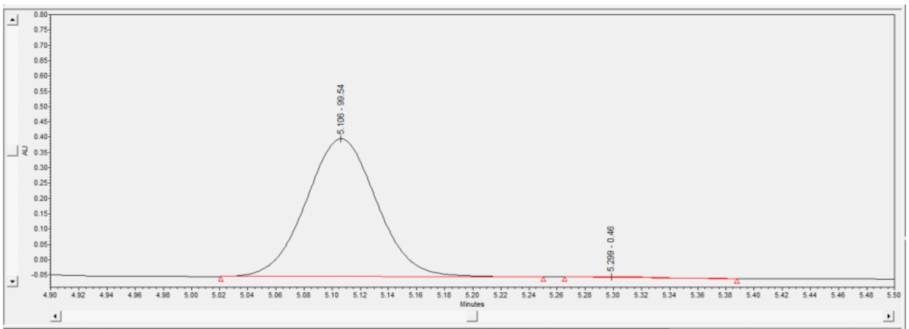

	Name	Retention Time (min)	Purity1 Angle	Purity1 Threshold	PDA/FLR Match1 Spect. Name	PDA/FLR Match1 Angle	PDA/FLR Match1 Threshold	PDA/FLR Match1 Lib. Name	Area (µV*sec)	% Area	Height (µV)	Int Type	Amount	Units	Peak Type	Peak Codes
1		5.106							1571297	99.54	450363	bb			Unknown	
2		5.299							7253	0.46	2511	bb			Unknown	I08

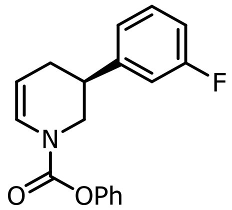

Phenyl-(S)-3-(3-fluorophenyl)-3,4-dihydropyridine-1(2H)-carboxylate (3u): The corresponding compound was prepared following general procedure **A** using 3-fluorophenyl boronic acid and phenyl pyridine-1(2H)-carboxylate. The mixture was stirred at 70 °C for 20 hours. Purification by flash chromatography (2% acetone/petrol to 20% acetone/petrol) afforded compound **3u** as viscous liquid (75% yield, 98% ee).

^{1}H NMR (500 MHz, $CDCl_3$) (2 rotamers): δ (ppm) 7.42 – 7.29 (m, 3H), 7.25 – 6.93 (m, 7H), 5.18 (dddd, *J* = 37.5, 8.1, 5.2, 2.6 Hz, 1H), 4.30 (ddq, *J* = 14.4, 12.7, 1.6 Hz, 1H), 3.41 (ddd, *J* = 86.7, 12.7, 11.2 Hz, 1H), 3.19 – 3.06 (m, 1H), 2.45 – 2.27 (m, 2H). **^{13}C NMR** (126 MHz, $CDCl_3$) (2 rotamers): δ (ppm) 163.2, 151.7, 151.2, 151.0, 145.4, 145.3, 130.4, 130.3, 129.5, 129.46, 125.8, 125.4, 125.0, 123.0, 121.8, 121.7, 114.3, 114.2, 114.0, 113.98, 107.5, 107.1, 48.3, 47.7, 38.5, 38.3, 29.3, 29.1. **^{19}F NMR** (471 MHz, $CDCl_3$) (2 rotamers): δ (ppm) −112.68, −112.81.

HRMS (ESI): m/z calcd for $C_{18}H_{17}O_2FN^+$ $[M + H]^+$ 298.1238 found 298.1250.

IR (ν_{max}/cm^{-1}) 1723, 1656, 1591, 1407, 1361, 1266, 1203, 1076, 977, 860, 751, 729, 690.

SFC Conditions: Chiralpak IC; Gradient 1; 99:1 er (major enantiomer t_R = 3.62 min; minor enantiomer t_R = 3.83 min), **98% ee**. **$[\alpha]^{25}_D$ =** −45.0 (c = 2.0, $CHCl_3$).

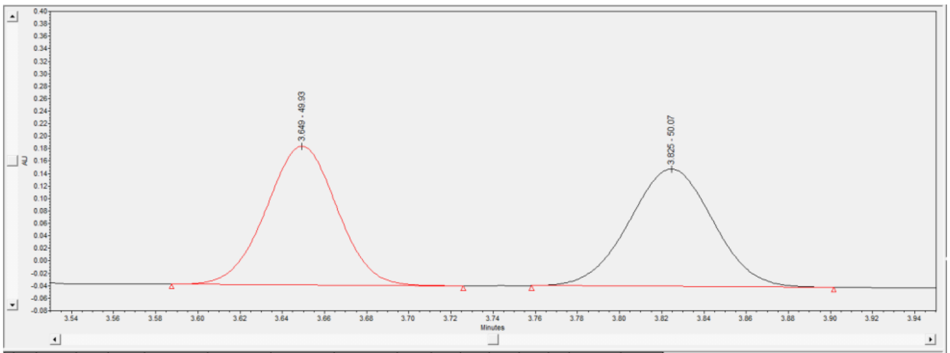

	Name	Retention Time (min)	Purity1 Angle	Purity1 Threshold	PDA/FLR Match1 Spect. Name	PDA/FLR Match1 Angle	PDA/FLR Match1 Threshold	PDA/FLR Match1 Lib. Name	Area (μV*sec)	% Area	Height (μV)	Int Type	Amount	Units	Peak Type	Peak Codes
1		3.649							520469	49.93	222455	bb			Unknown	
2		3.825							522014	50.07	188206	bb			Unknown	

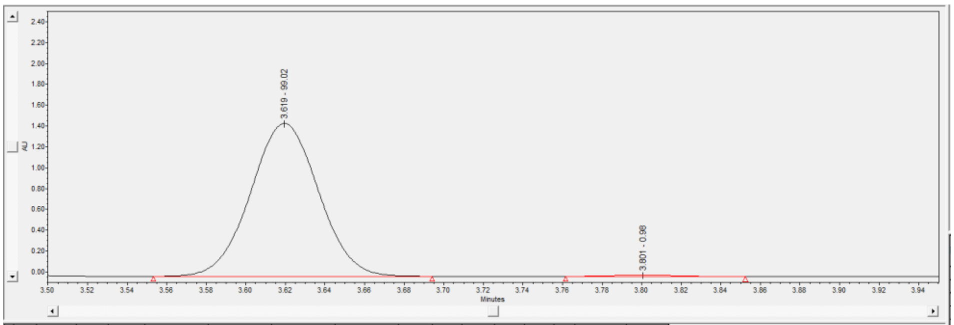

	Name	Retention Time (min)	Purity1 Angle	Purity1 Threshold	PDA/FLR Match1 Spect. Name	PDA/FLR Match1 Angle	PDA/FLR Match1 Threshold	PDA/FLR Match1 Lib. Name	Area (μV*sec)	% Area	Height (μV)	Int Type	Amount	Units	Peak Type	Peak Codes
1		3.619							3507229	99.02	1466780	bb			Unknown	
2		3.801							34820	0.98	13344	bb			Unknown	

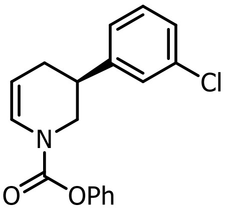

Phenyl-(S)-3-(3-chlorophenyl)-3,4-dihydropyridine-1(2H)-carboxylate (3v): The corresponding compound was prepared following general procedure **A** using 3-chlorophenyl boronic acid and phenyl pyridine-1(2H)-carboxylate. The mixture was stirred at 70 °C for 20 hours. Purification by flash chromatography (2% acetone/petrol to 20% acetone/petrol) afforded compound **3v** as viscous liquid (78% yield, 98% ee).

^{1}H NMR (500 MHz, $CDCl_3$) (2 rotamers): δ (ppm) 7.38 (td, *J* = 9.1, 7.5 Hz, 2H), 7.31 – 7.19 (m, 4H), 7.18 – 7.01 (m, 4H), 5.25 – 5.09 (m, 1H), 4.29 (dddd, *J* = 14.3, 12.6, 4.3, 1.6 Hz, 1H), 3.53 – 3.26 (m, 1H), 3.09 (dddd, *J* = 20.1, 10.4, 5.8, 2.7 Hz, 1H), 2.47 – 2.23 (m, 2H).**^{13}C NMR** (126 MHz, $CDCl_3$) (2 rotamers): δ (ppm) 152.0, 151.7, 151.2, 151.0, 144.8, 144.7, 134.7, 134.6, 130.2, 130.1, 129.5, 129.47, 127.6, 127.5, 127.3, 127.29, 125.8, 125.6, 125.4, 125.1, 121.8, 121.7, 107.4, 107.1, 48.2, 47.6, 38.5, 38.3, 29.3, 29.1.

HRMS (ESI): m/z calcd for $C_{18}H_{16}O_2ClN^+$ $[M + H]^+$ 314.0942 found 314.0953.

IR (ν_{max}/cm^{-1}) 1722, 1406, 1359, 1258, 1201, 1164, 1074, 786, 749, 690.

SFC Conditions: Chiralpak IF; Gradient 1; 99:1 er (major enantiomer t_R = 4.81 min; minor enantiomer t_R = 5.00 min), **98% ee. [α]$^{25}_D$ =** −35.3 (c = 2.0, $CHCl_3$).

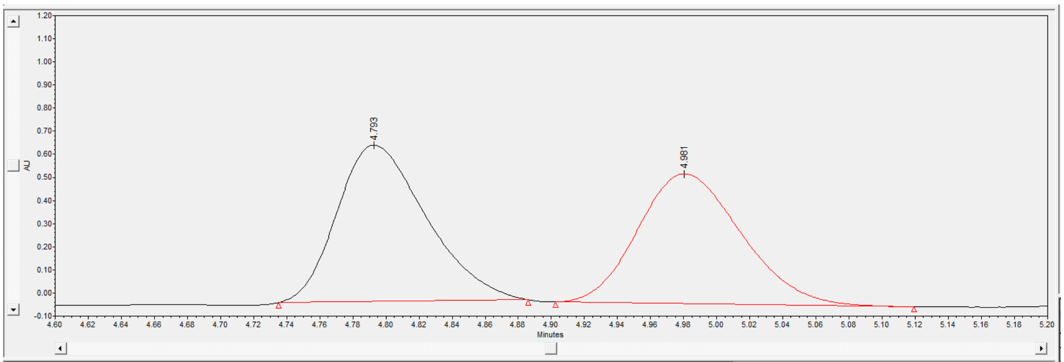

	Name	Retention Time (min)	Purity1 Angle	Purity1 Threshold	PDA/FLR Match1 Spect. Name	PDA/FLR Match1 Angle	PDA/FLR Match1 Threshold	PDA/FLR Match1 Lib. Name	Area (μV*sec)	% Area	Height (μV)	Int Type	Amount	Units	Peak Type	Peak Codes
1		4.793							2543298	51.03	676780	bb			Unknown	
2		4.981							2440795	48.97	562042	bb			Unknown	

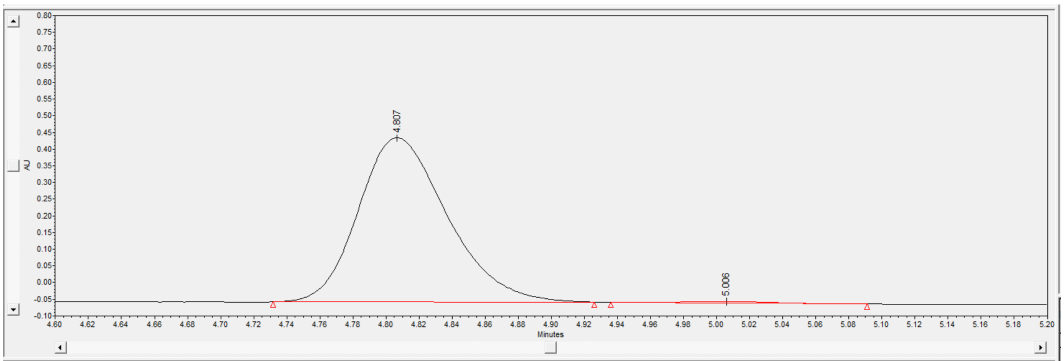

	Name	Retention Time (min)	Purity1 Angle	Purity1 Threshold	PDA/FLR Match1 Spect. Name	PDA/FLR Match1 Angle	PDA/FLR Match1 Threshold	PDA/FLR Match1 Lib. Name	Area (μV*sec)	% Area	Height (μV)	Int Type	Amount	Units	Peak Type	Peak Codes
1		4.807							1839901	98.95	492308	bb			Unknown	
2		5.006							19508	1.05	5394	bb			Unknown	I08

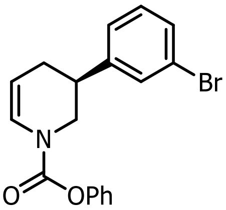

Phenyl-(S)-3-(3-bromophenyl)-3,4-dihydropyridine-1(2H)-carboxylate (3w): The corresponding compound was prepared following general procedure **A** using 3-bromophenyl boronic acid and phenyl pyridine-1(2H)-carboxylate. The mixture was stirred at 70 °C for 20 hours. Purification by flash chromatography (2% acetone/petrol to 20% acetone/petrol) afforded compound **3w** as viscous liquid (60% yield, 99% ee).

^{1}H NMR (400 MHz, $CDCl_3$) (2 rotamers): δ (ppm) 7.45 – 7.33 (m, 4H), 7.25 – 7.00 (m, 6H), 5.26 – 5.01 (m, 1H), 4.31 – 4.24 (m, 1H), 3.60 – 3.24 (m, 1H), 3.08 (tp, J = 10.4, 5.4 Hz, 1H), 2.46 – 2.25 (m, 2H). **^{13}C NMR** (126 MHz, $CDCl_3$) (2 rotamers): δ (ppm) 152.0, 151.7, 151.2, 151.0, 145.1, 145.0, 130.52, 130.5, 130.42, 130.4, 130.31, 130.3, 129.5, 129.48, 129.46, 129.4, 126.0, 125.8, 125.4, 125.1, 123.0, 122.9, 121.8, 121.7, 107.4, 107.1, 48.3, 47.7, 38.5, 38.3, 29.3, 29.2.

HRMS (ESI): m/z calcd for $C_{18}H_{17}O_2BrN^+$ $[M + H]^+$ 358.0437 found 358.0434.

IR (ν_{max}/cm^{-1}) 1723, 1406, 1360, 1257, 1201, 1072, 783, 750, 690.

SFC Conditions: Chiralpak IG; Gradient 1; 99:1 er (major enantiomer t_R = 5.80 min; minor enantiomer t_R = 6.00 min), **98% ee. $[\alpha]^{25}_D$ =** −34.5 (c = 2.0, $CHCl_3$).

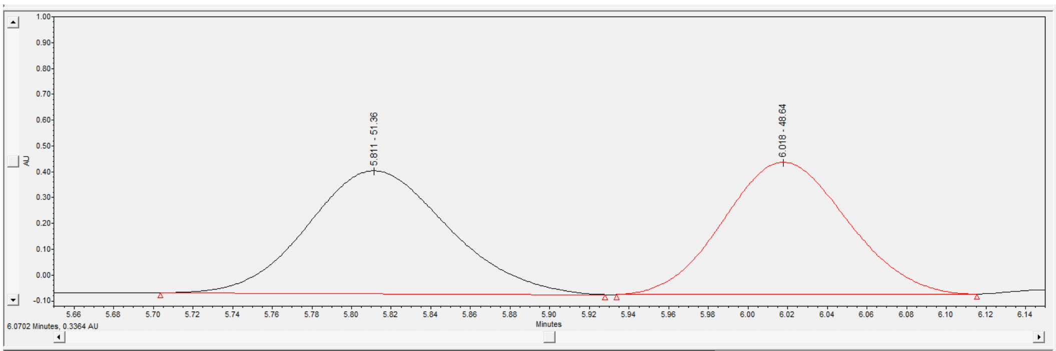

	Name	Retention Time (min)	Purity1 Angle	Purity1 Threshold	PDA/FLR Match1 Spect. Name	PDA/FLR Match1 Angle	PDA/FLR Match1 Threshold	PDA/FLR Match1 Lib. Name	Area (μV*sec)	% Area	Height (μV)	Int Type	Amount	Units	Peak Type	Peak Codes
1		5.811							2339740	51.36	475395	bb			Unknown	
2		6.018							2215582	48.64	510932	bb			Unknown	

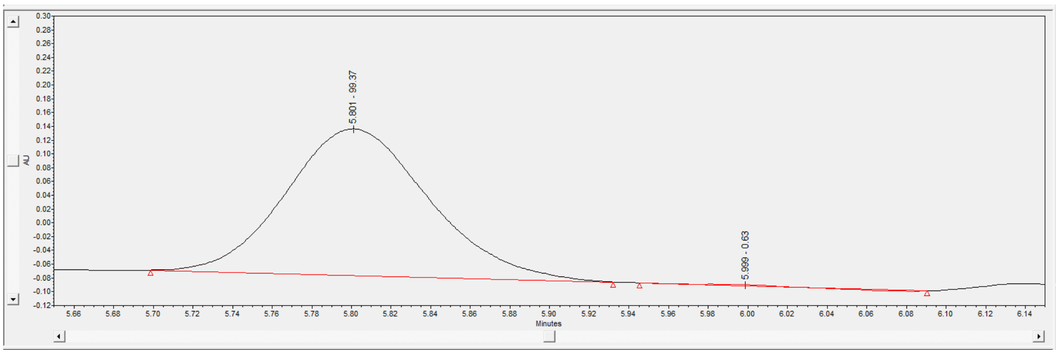

	Name	Retention Time (min)	Purity1 Angle	Purity1 Threshold	PDA/FLR Match1 Spect. Name	PDA/FLR Match1 Angle	PDA/FLR Match1 Threshold	PDA/FLR Match1 Lib. Name	Area (μV*sec)	% Area	Height (μV)	Int Type	Amount	Units	Peak Type	Peak Codes
1		5.801							1064876	99.37	212681	bb			Unknown	
2		5.999							6784	0.63	1832	bb			Unknown	I08

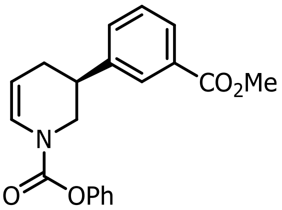

Phenyl-(S)-3-(3-(methoxycarbonyl)phenyl)-3,4-dihydropyridine-1(2H)-carboxylate (3x): The corresponding compound was prepared following general procedure **A** using 3-(methoxycarbonyl)phenyl boronic acid and phenyl pyridine-1(2H)-carboxylate. The mixture was stirred at 70 °C for 20 hours. Purification by flash chromatography (2% acetone/petrol to 20% acetone/petrol) afforded compound **3x** as viscous liquid (50% yield, 96% ee).

^{1}H NMR (500 MHz, $CDCl_3$) (2 rotamers): δ (ppm) 8.00 – 7.89 (m, 2H), 7.50 – 7.32 (m, 4H), 7.26 – 7.00 (m, 4H), 5.26 – 5.11 (m, 1H), 4.35 – 4.25 (m, 1H), 3.96 – 3.87 (m, 3H), 3.58 – 3.31 (m, 1H), 3.23 – 3.10 (m, 1H), 2.45 – 2.32 (m, 2H). **^{13}C NMR** (126 MHz, $CDCl_3$) (2 rotamers): δ (ppm) 167.1, 152.1, 151.8, 151.2, 151.0, 143.2, 143.0, 132.0, 130.8, 130.7, 129.51, 129.5, 129.0, 128.9, 128.5, 128.43, 128.42, 128.40, 125.8, 125.4, 125.1, 121.8, 121.7, 107.6, 107.2, 52.3, 48.3, 47.7, 38.6, 38.4, 29.3, 29.2.

HRMS (ESI): m/z calcd for $C_{20}H_{20}O_4N^+$ $[M + H]^+$ 338.1387 found 338.1390.

IR (ν_{max}/cm^{-1}) 1721, 1406, 1361, 1290, 1254, 1202, 752, 690.

SFC Conditions: Chiralpak IF; Gradient 1; 98:2 er (major enantiomer t_R = 5.77 min; minor enantiomer t_R = 5.97 min), **96% ee. [α]$^{25}_D$ =** −44.3 (c = 2.0, $CHCl_3$).

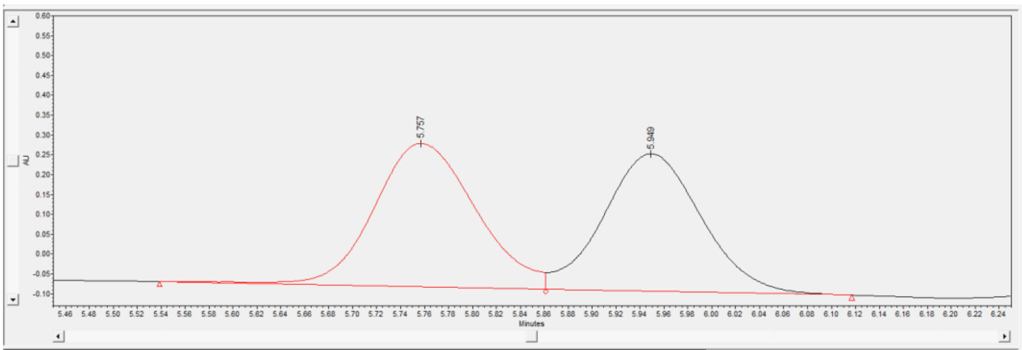

	Name	Retention Time (min)	Purity1 Angle	Purity1 Threshold	PDA/FLR Match1 Spect. Name	PDA/FLR Match1 Angle	PDA/FLR Match1 Threshold	PDA/FLR Match1 Lib. Name	Area (µV*sec)	% Area	Height (µV)	Int Type	Amount	Units	Peak Type	Peak Codes
1		5.757							2128451	52.07	360978	bv			Unknown	
2		5.949							1959477	47.93	345962	vb			Unknown	

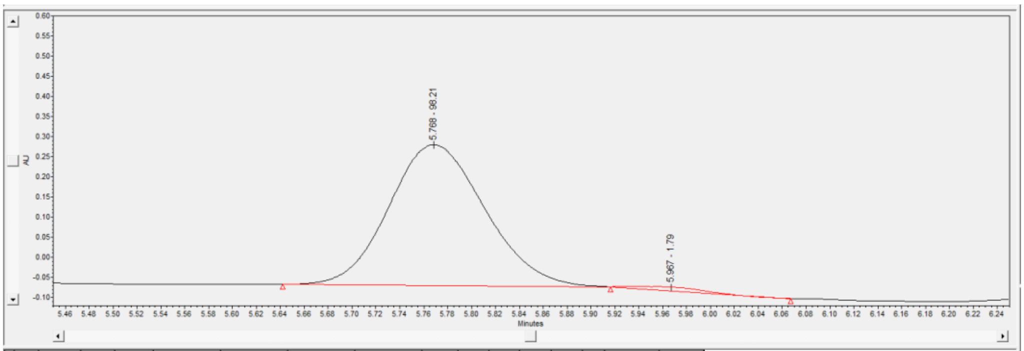

	Name	Retention Time (min)	Purity1 Angle	Purity1 Threshold	PDA/FLR Match1 Spect. Name	PDA/FLR Match1 Angle	PDA/FLR Match1 Threshold	PDA/FLR Match1 Lib. Name	Area (µV*sec)	% Area	Height (µV)	Int Type	Amount	Units	Peak Type	Peak Codes
1		5.768							1954199	98.21	349706	bb			Unknown	
2		5.967							35665	1.79	9185	bb			Unknown	I08

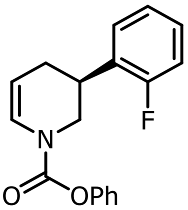

Phenyl-(S)-3-(2-fluorophenyl)-3,4-dihydropyridine-1(2H)-carboxylate (3y): The corresponding compound was prepared following general procedure **A** using 2-fluoro phenyl boronic acid and phenyl pyridine-1(2H)-carboxylate. The mixture was stirred at 70 °C for 20 hours. Purification by flash chromatography (2% acetone/petrol to 15% acetone/petrol) afforded compound **3y** as viscous liquid (25% yield, 99% ee).

^{1}H NMR (500 MHz, $CDCl_3$) (2 rotamers): δ (ppm) ^{1}H NMR (500 MHz, $CDCl_3$) δ 7.42 – 7.28 (m, 4H), 7.25 – 6.93 (m, 9H), 5.30 – 5.05 (m, 1H), 4.30 (t, *J* = 12.4 Hz, 1H), 3.60 – 3.24 (m, 1H), 3.12 (dtt, *J* = 15.0, 10.5, 4.6 Hz, 1H), 2.45 – 2.27 (m, 3H). **^{13}C NMR** (101 MHz, $CDCl_3$) (2 rotamers): δ (ppm) ^{13}C NMR (126 MHz, $CDCl_3$) δ 163.18, 151.75, 151.18, 151.00, 145.36, 145.26, 130.35, 130.29, 129.50, 129.46, 125.76, 125.40, 125.05, 123.03, 121.77, 121.67, 114.29, 114.22, 114.03, 113.98, 107.46, 107.09, 48.28, 47.67, 38.45, 38.33, 29.29, 29.15. **^{19}F NMR** (471 MHz, $CDCl_3$) (2 rotamers): δ (ppm) −112.7, −112.8.

HRMS (ESI): m/z calcd for $C_{20}H_{20}O_4N^+$ $[M + H]^+$ 338.1387 found 338.1390.

IR (ν_{max}/cm^{-1}) 1723, 1493, 1407, 1363, 1257, 1204, 755, 689.

SFC Conditions: Chiralpak IF; Gradient 1; 99.5:0.5 er (major enantiomer t_R = 3.57 min; minor enantiomer t_R = 3.71 min), **99% ee**. **$[\alpha]^{25}_{D}$ =** −46.2 (c = 2.0, $CHCl_3$).

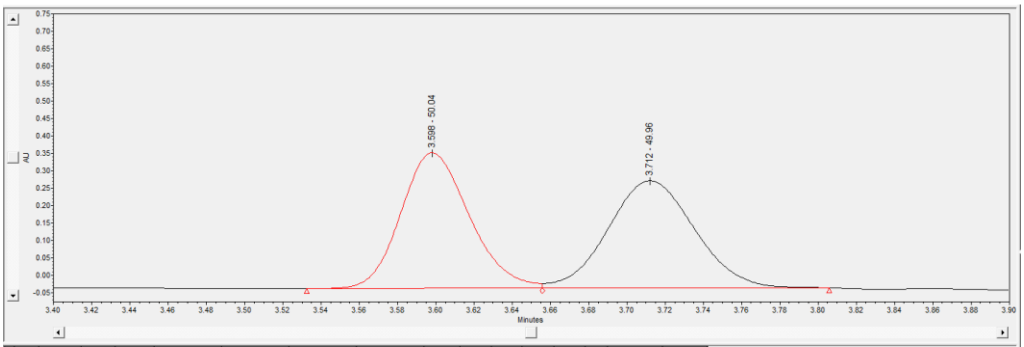

	Name	Retention Time (min)	Purity1 Angle	Purity1 Threshold	PDA/FLR Match1 Spect. Name	PDA/FLR Match1 Angle	PDA/FLR Match1 Threshold	PDA/FLR Match1 Lib. Name	Area (µV*sec)	% Area	Height (µV)	Int Type	Amount	Units	Peak Type	Peak Codes
1		3.598							969232	50.04	388556	bv			Unknown	
2		3.712							967517	49.96	307732	vb			Unknown	

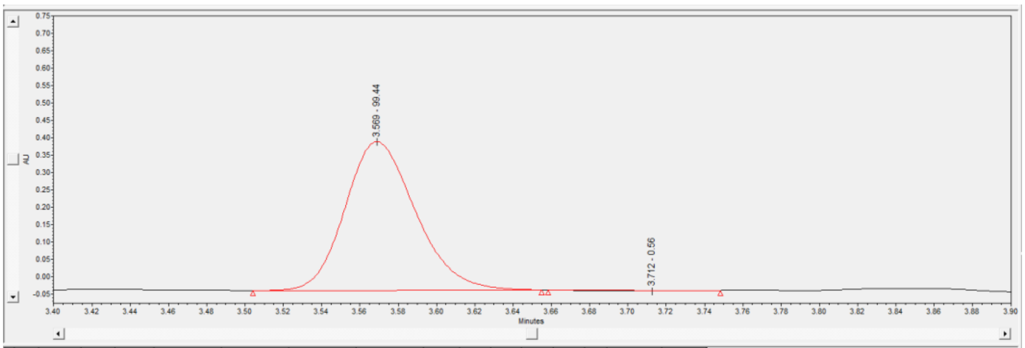

	Name	Retention Time (min)	Purity1 Angle	Purity1 Threshold	PDA/FLR Match1 Spect. Name	PDA/FLR Match1 Angle	PDA/FLR Match1 Threshold	PDA/FLR Match1 Lib. Name	Area (µV*sec)	% Area	Height (µV)	Int Type	Amount	Units	Peak Type	Peak Codes
1		3.569							1090592	99.44	426948	bb			Unknown	
2		3.712							6167	0.56	-1712	bb			Unknown	I08

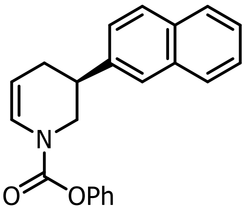

Phenyl-(S)-3-(naphthalen-2-yl)-3,4-dihydropyridine-1(2H)-carboxylate (3z): The corresponding compound was prepared following general procedure **A** using naphthalen-2-yl boronic acid and phenyl pyridine-1(2H)-carboxylate. The mixture was stirred at 70 °C for 20 hours. Purification by flash chromatography (2% acetone/petrol to 25% acetone/petrol) afforded compound **3z** as viscous liquid (79% yield, 96% ee).

^{1}H NMR (400 MHz, $CDCl_3$) (2 rotamers): δ (ppm) 7.90 – 7.64 (m, 4H), 7.54 – 7.44 (m, 2H), 7.44 – 7.33 (m, 3H), 7.29 – 7.03 (m, 4H), 5.32 – 5.15 (m, 1H), 4.41 (ddd, *J* = 12.1, 7.5, 3.7 Hz, 1H), 3.67 – 3.39 (m, 1H), 3.29 (ttd, *J* = 11.3, 7.5, 3.7 Hz, 1H), 2.48 (ddd, *J* = 8.5, 3.7, 2.0 Hz, 2H). **^{13}C NMR** (101 MHz, $CDCl_3$) (2 rotamers): δ (ppm) 152.1, 151.8, 151.2, 151.1, 140.2, 140.1, 133.7, 132.6, 129.5, 129.4, 128.6, 128.5, 127.8, 127.8, 127.76, 126.4, 126.3, 125.9, 125.85, 125.83, 125.81, 125.7, 125.68, 125.6, 125.4, 125.0, 121.8, 121.7, 107.9, 107.5, 48.5, 47.9, 38.8, 38.7, 29.4, 29.3.

HRMS (ESI): m/z calcd for $C_{22}H_{20}O_2N^+$ $[M + H]^+$ 330.1489 found 330.1493.

IR (ν_{max}/cm^{-1}) 1721, 1406, 1368, 1351, 1202, 1075, 975, 854, 818, 748, 720, 689.

SFC Conditions: Chiralpak IE; Gradient 1; 98:2 er (major enantiomer t_R = 6.12 min; minor enantiomer t_R = 6.00 min), **96% ee**. **[α]$^{25}_{D}$ =** −89.3 (c = 2.0, $CHCl_3$).

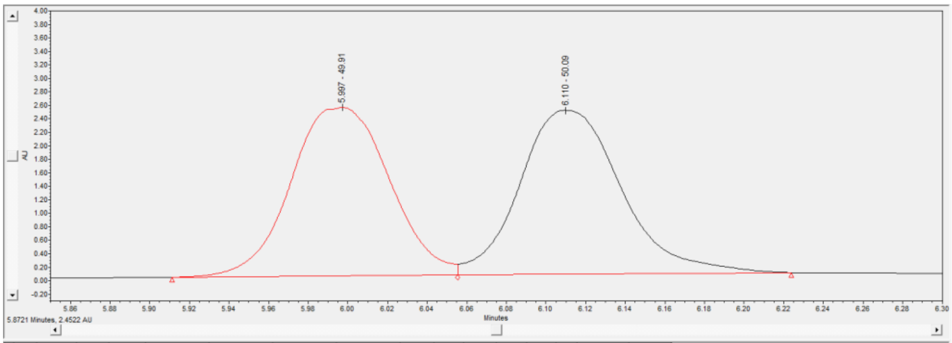

	Name	Retention Time (min)	Purity1 Angle	Purity1 Threshold	PDA/FLR Match1 Spect. Name	PDA/FLR Match1 Angle	PDA/FLR Match1 Threshold	PDA/FLR Match1 Lib. Name	Area (µV*sec)	% Area	Height (µV)	Int Type	Amount	Units	Peak Type	Peak Codes
1		5.997							8639939	49.91	2499721	bv			Unknown	
2		6.110							8670501	50.09	2430919	vb			Unknown	

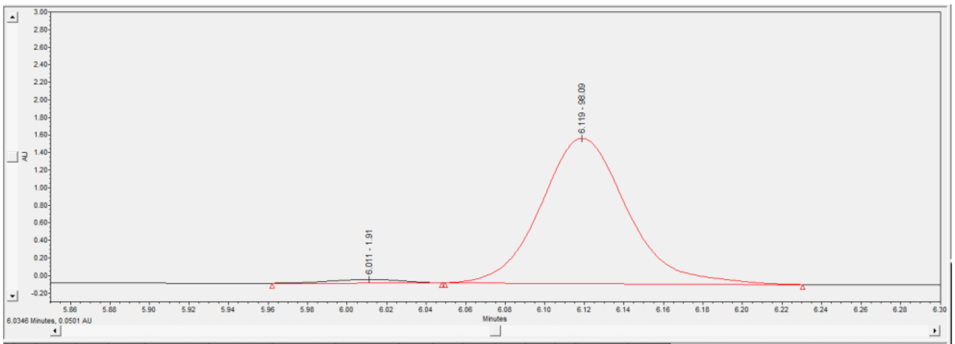

	Name	Retention Time (min)	Purity1 Angle	Purity1 Threshold	PDA/FLR Match1 Spect. Name	PDA/FLR Match1 Angle	PDA/FLR Match1 Threshold	PDA/FLR Match1 Lib. Name	Area (µV*sec)	% Area	Height (µV)	Int Type	Amount	Units	Peak Type	Peak Codes
1		6.011							97930	1.91	40582	bb			Unknown	
2		6.119							5016185	98.09	1652353	bb			Unknown	

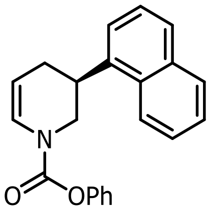

Phenyl-(S)-3-(naphthalen-1-yl)-3,4-dihydropyridine-1(2H)-carboxylate (3aa): The corresponding compound was prepared following general procedure **A** using naphthalen-1-yl boronic acid and phenyl pyridine-1(2H)-carboxylate. The mixture was stirred at 70 °C for 20 hours. Purification by flash chromatography (2% acetone/petrol to 25% acetone/petrol) afforded compound **3aa** as viscous liquid (41% yield, 99% ee).

^{1}H NMR (500 MHz, $CDCl_3$) (2 rotamers): δ (ppm) 8.18 (dd, *J* = 16.9, 8.5 Hz, 1H), 7.91 (t, *J* = 8.2 Hz, 1H), 7.83 – 7.77 (m, 1H), 7.63 – 7.46 (m, 3H), 7.38 (dq, *J* = 16.2, 8.2 Hz, 3H), 7.26 – 7.14 (m, 3H), 7.10 (t, *J* = 7.4 Hz, 1H), 5.38 – 5.15 (m, 1H), 4.50 (t, *J* = 9.1 Hz, 1H), 3.96 (ddt, *J* = 16.1, 9.9, 5.9 Hz, 1H), 3.74 – 3.39 (m, 1H), 2.60 – 2.44 (m, 2H). **^{13}C NMR** (126 MHz, $CDCl_3$) (2 rotamers): δ (ppm) 152.1, 151.7, 151.2, 151.1, 138.7, 138.6, 134.1, 134.0, 131.5, 131.48, 129.5, 129.4, 129.3, 129.2, 127.6, 127.6, 126.5, 126.46, 125.8, 125.79, 125.7, 125.67, 125.4, 125.0, 123.2, 123.1, 122.9, 122.8, 121.8, 121.7, 108.3, 107.8, 48.4, 47.8, 33.5, 33.4, 29.6, 29.4.

HRMS (ESI): m/z calcd for $C_{22}H_{20}O_2N^+$ $[M + H]^+$ 330.1489 found 330.1487.

IR (ν_{max}/cm^{-1}) 1723, 1406, 1369, 1260, 1235, 1204, 798, 749, 689.

SFC Conditions: Chiralpak ID; Gradient 1; 99.5:0.5 er (major enantiomer t_R = 4.51 min; minor enantiomer t_R = 4.22 min), **99% ee**. **$[\alpha]^{25}_D$ =** −100.1 (c = 2.0, $CHCl_3$).

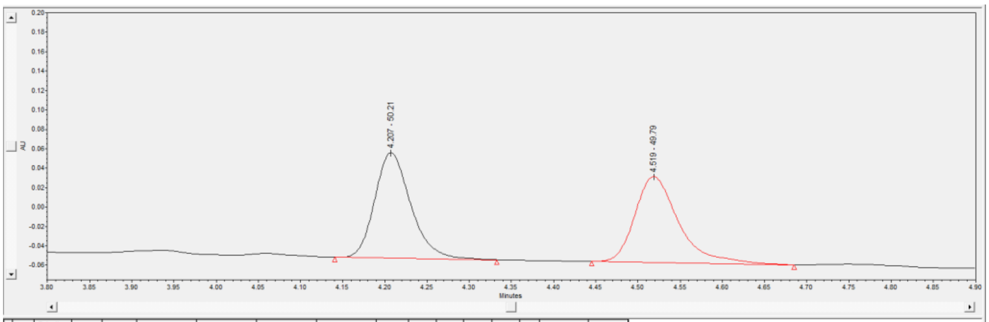

	Name	Retention Time (min)	Purity1 Angle	Purity1 Threshold	PDA/FLR Match1 Spect. Name	PDA/FLR Match1 Angle	PDA/FLR Match1 Threshold	PDA/FLR Match1 Lib. Name	Area (µV*sec)	% Area	Height (µV)	Int Type	Amount	Units	Peak Type	Peak Codes
1		4.207							321780	50.21	108651	bb			Unknown	
2		4.519							319052	49.79	88466	bb			Unknown	

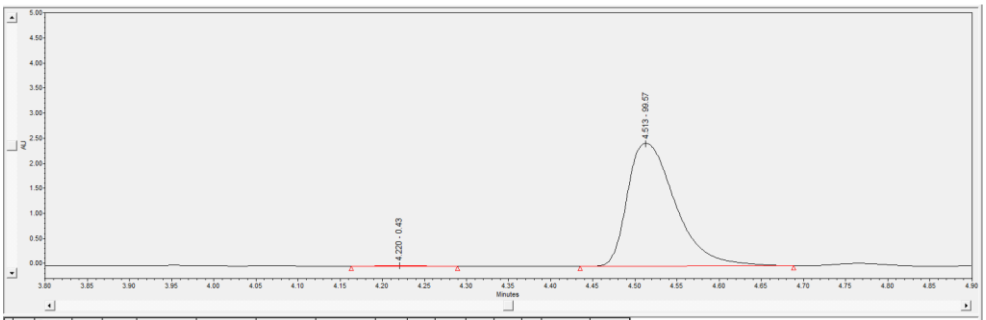

	Name	Retention Time (min)	Purity1 Angle	Purity1 Threshold	PDA/FLR Match1 Spect. Name	PDA/FLR Match1 Angle	PDA/FLR Match1 Threshold	PDA/FLR Match1 Lib. Name	Area (µV*sec)	% Area	Height (µV)	Int Type	Amount	Units	Peak Type	Peak Codes
1		4.220							41398	0.43	14998	bb			Unknown	
2		4.513							9677089	99.57	2450305	bb			Unknown	

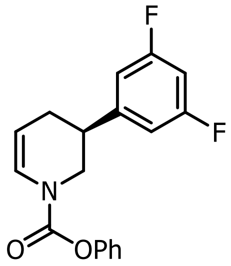

Phenyl-(S)-3-(3,5-difluorophenyl)-3,4-dihydropyridine-1(2H)-carboxylate (3ab): The corresponding compound was prepared following general procedure **A** using 3,5-difluorophenyl boronic acid and phenyl pyridine-1(2H)-carboxylate. The mixture was stirred at 70 °C for 20 hours. Purification by flash chromatography (2% acetone/petrol to 25% acetone/petrol) afforded compound **3ab** as viscous liquid (70% yield, 94% ee).

^{1}H NMR (500 MHz, $CDCl_3$) (2 rotamers): δ (ppm) 7.37 (q, J = 8.0 Hz, 2H), 7.26 – 6.99 (m, 4H), 6.85 – 6.65 (m, 3H), 5.26 – 5.08 (m, 1H), 4.33 – 4.21 (m, 1H), 3.55 – 3.24 (m, 1H), 3.11 (ddt, J = 14.5, 9.5, 5.1 Hz, 1H), 2.46 – 2.20 (m, 2H). **^{13}C NMR** (126 MHz, $CDCl_3$) (^{19}F decoupled) (2 rotamers): δ (ppm) 163.4, 163.3, 152.0, 151.7, 151.1, 150.9, 146.7, 146.6, 129.53, 129.5, 125.8, 125.5, 125.2, 121.8, 121.7, 110.3, 110.27, 107.1, 106.7, 102.6, 102.58, 48.0, 47.4, 38.5, 38.3, 29.1, 29.0. **^{19}F NMR** (471 MHz, $CDCl_3$) (2 rotamers): δ (ppm) −109.4, −109.5.

HRMS (ESI): m/z calcd for $C_{18}H_{15}O_2F_2N^+$ $[M + H]^+$ 338.0963 found 338.0976.

IR (ν_{max}/cm^{-1}) 1722, 1625, 1598, 1408, 1379, 1345, 1204, 1118, 982, 851, 750, 690.

SFC Conditions: Chiralpak IC; Gradient 1; 97:3 er (major enantiomer t_R = 3.26 min; minor enantiomer t_R = 3.40 min), **94% ee**. **$[\alpha]^{25}_D$ =** −51.2 (c = 2.0, $CHCl_3$).

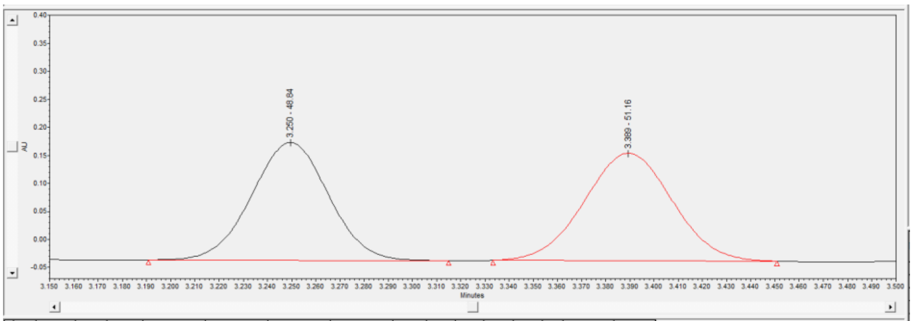

	Name	Retention Time (min)	Purity1 Angle	Purity1 Threshold	PDA/FLR Match1 Spect. Name	PDA/FLR Match1 Angle	PDA/FLR Match1 Threshold	PDA/FLR Match1 Lib. Name	Area (µV*sec)	% Area	Height (µV)	Int Type	Amount	Units	Peak Type	Peak Codes
1		3.250							464285	48.84	209946	bb			Unknown	
2		3.389							486359	51.16	191822	bb			Unknown	

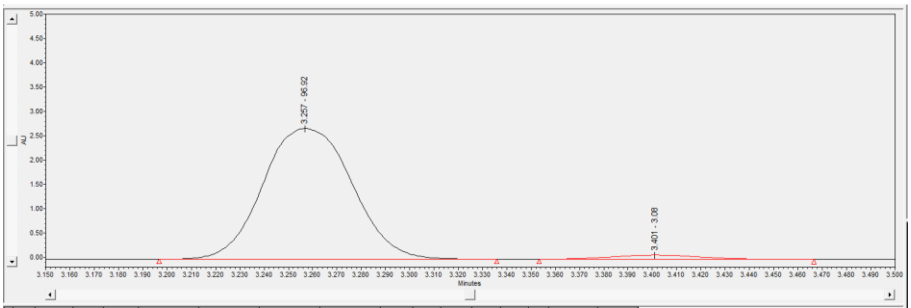

	Name	Retention Time (min)	Purity1 Angle	Purity1 Threshold	PDA/FLR Match1 Spect. Name	PDA/FLR Match1 Angle	PDA/FLR Match1 Threshold	PDA/FLR Match1 Lib. Name	Area (µV*sec)	% Area	Height (µV)	Int Type	Amount	Units	Peak Type	Peak Codes
1		3.257							6858004	96.92	2686890	bb			Unknown	
2		3.401							218200	3.08	85830	bb			Unknown	

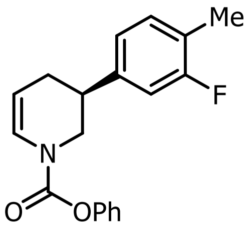

Phenyl-(S)-3-(3-fluoro-4-methylphenyl)-3,4-dihydropyridine-1(2H)-carboxylate (3ac): The corresponding compound was prepared following general procedure **A** using 3-fluoro-4-methylphenyl boronic acid and phenyl pyridine-1(2H)-carboxylate. The mixture was stirred at 70 °C for 20 hours. Purification by flash chromatography (2% acetone/petrol to 25% acetone/petrol) afforded compound **3ac** as viscous liquid (50% yield, 99% ee).

^{1}H NMR ($CDCl_3$, 400 MHz) (2 rotamers): δ (ppm) 7.37 (q, *J* = 8.1 Hz, 2H), 7.24 – 6.95 (m, 7H), 5.25 – 5.09 (m, 1H), 4.32 – 4.20 (m, 1H), 3.49 – 3.19 (m, 1H), 3.05 (tp, *J* = 10.8, 5.5 Hz, 1H), 2.37 – 2.23 (m, 5H). **^{13}C NMR** (126 MHz, $CDCl_3$) (2 rotamers): δ (ppm) 160.4, 152.1, 151.8, 151.2, 151.0, 138.2, 138.1, 130.3, 129.5, 129.46, 126.0, 125.9, 125.74, 125.7, 125.3, 125.2, 125.1, 124.9, 121.8, 121.7, 115.3, 115.2, 107.7, 107.4, 48.7, 48.1, 38.0, 37.9, 29.6, 29.5, 14.8. **^{19}F NMR** (471 MHz, $CDCl_3$) (2 rotamers): δ (ppm) −120.09, −120.24.

HRMS (ESI): m/z calcd for $C_{18}H_{15}O_2F_2N^+$ $[M + H]^+$ 316.1144 found 316.1168.

IR (ν_{max}/cm^{-1}) 1723, 1504, 1406, 1359, 1251, 1202, 866, 819, 750, 721, 689.

SFC Conditions: Chiralpak IA; Gradient 3; 99.5:0.5 er (major enantiomer t_R = 6.30 min; minor enantiomer t_R = 6.16 min), **99% ee**. **$[\alpha]^{25}_D$ =** −50.8 (c = 2.0, $CHCl_3$).

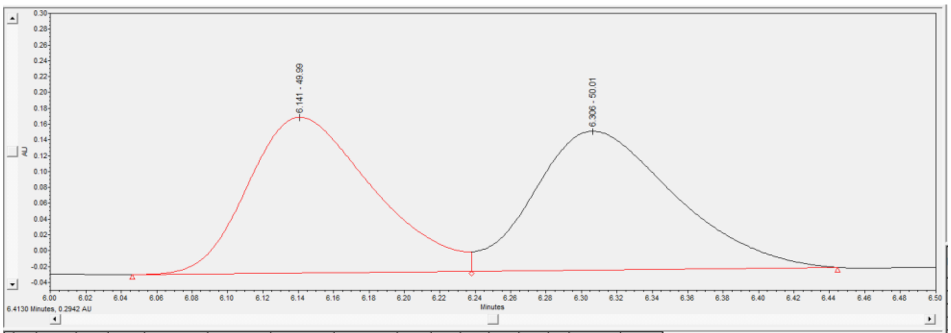

	Name	Retention Time (min)	Purity1 Angle	Purity1 Threshold	PDA/FLR Match1 Spect. Name	PDA/FLR Match1 Angle	PDA/FLR Match1 Threshold	PDA/FLR Match1 Lib. Name	Area (µV*sec)	% Area	Height (µV)	Int Type	Amount	Units	Peak Type	Peak Codes
1		6.141							965775	49.99	196453	bv			Unknown	
2		6.306							966264	50.01	175197	vb			Unknown	

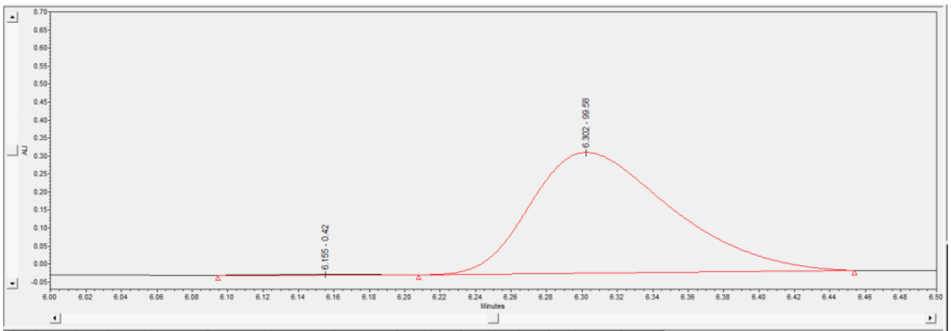

	Name	Retention Time (min)	Purity1 Angle	Purity1 Threshold	PDA/FLR Match1 Spect. Name	PDA/FLR Match1 Angle	PDA/FLR Match1 Threshold	PDA/FLR Match1 Lib. Name	Area (µV*sec)	% Area	Height (µV)	Int Type	Amount	Units	Peak Type	Peak Codes
1		6.155							7735	0.42	2203	bb			Unknown	
2		6.302							1844914	99.58	334989	bb			Unknown	

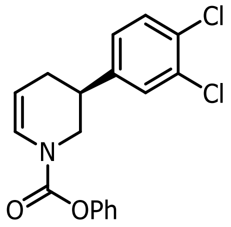

Phenyl-(S)-3-(3,4-dichlorophenyl)-3,4-dihydropyridine-1(2H)-carboxylate (3ad): The corresponding compound was prepared following general procedure **A** using 3,4-dichlorophenyl boronic acid and phenyl pyridine-1(2H)-carboxylate. The mixture was stirred at 70 °C for 20 hours. Purification by flash chromatography (2% acetone/petrol to 20% acetone/petrol) afforded compound **3ad** as viscous liquid (60% yield, 96% ee).

^{1}H NMR ($CDCl_3$, 400 MHz) (2 rotamers): δ (ppm) 7.46 – 7.32 (m, 4H), 7.25 – 6.99 (m, 5H), 5.23 – 5.10 (m, 1H), 4.32 – 4.19 (m, 1H), 3.55 – 3.27 (m, 1H), 3.07 (dp, J = 14.0, 4.9 Hz, 1H), 2.47 – 2.21 (m, 2H). **^{13}C NMR** (101 MHz, $CDCl_3$) (2 rotamers): δ (ppm) 151.9, 151.7, 151.1, 150.9, 143.0, 142.9, 132.9, 132.8, 131.1, 131.05, 130.8, 130.7, 129.5, 129.46, 129.4, 129.3, 126.8, 125.8, 125.5, 125.1, 121.7, 121.6, 107.1, 106.8, 48.1, 47.4, 37.9, 37.8, 29.1, 29.0.

HRMS (ESI): m/z calcd for $C_{22}H_{20}O_2N^+$ $[M + H]^+$ 348.0553 found 348.0561.

IR (ν_{max}/cm^{-1}) 1724, 1407, 1374, 1357, 1255, 1202, 749, 689.

SFC Conditions: Chiralpak ID; Gradient 1; 98:2 er (major enantiomer t_R = 4.14 min; minor enantiomer t_R = 4.28 min), **96% ee. $[\alpha]^{25}_D$ =** −50.9 (c = 2.0, $CHCl_3$).

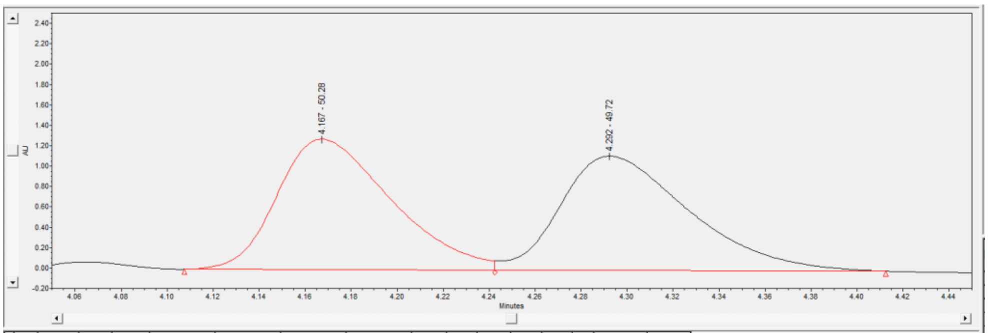

	Name	Retention Time (min)	Purity1 Angle	Purity1 Threshold	PDA/FLR Match1 Spect. Name	PDA/FLR Match1 Angle	PDA/FLR Match1 Threshold	PDA/FLR Match1 Lib. Name	Area (µV*sec)	% Area	Height (µV)	Int Type	Amount	Units	Peak Type	Peak Codes
1		4.167							4409858	50.28	1279625	bv			Unknown	
2		4.292							4360460	49.72	1121581	vb			Unknown	

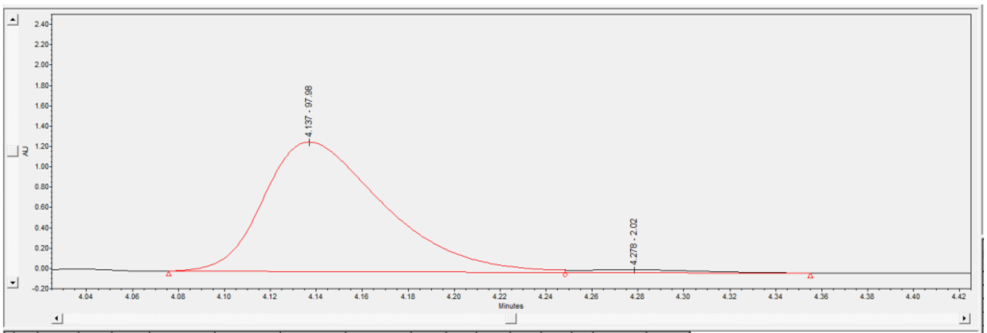

	Name	Retention Time (min)	Purity1 Angle	Purity1 Threshold	PDA/FLR Match1 Spect. Name	PDA/FLR Match1 Angle	PDA/FLR Match1 Threshold	PDA/FLR Match1 Lib. Name	Area (µV*sec)	% Area	Height (µV)	Int Type	Amount	Units	Peak Type	Peak Codes
1		4.137							4640014	97.98	1275419	bv			Unknown	
2		4.278							95753	2.02	26589	vb			Unknown	I08

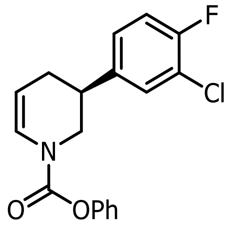

Phenyl-(S)-3-(3-chloro-4-fluorophenyl)-3,4-dihydropyridine-1(2H)-carboxylate (3ae): The corresponding compound was prepared following general procedure **A** using 3-chloro-4-fluorophenyl boronic acid and phenyl pyridine-1(2H)-carboxylate. The mixture was stirred at 70 °C for 20 hours. Purification by flash chromatography (2% acetone/petrol to 25% acetone/petrol) afforded compound **3ae** as viscous liquid (50% yield, 97% ee).

^{1}H NMR ($CDCl_3$, 400 MHz) (2 rotamers): δ (ppm) 7.42 – 7.27 (m, 2H), 7.25 – 6.99 (m, 7H), 5.24 – 5.09 (m, 1H), 4.32 – 4.19 (m, 1H), 3.52 – 3.23 (m, 1H), 3.08 (qt, J = 10.6, 5.2 Hz, 1H), 2.44 – 2.22 (m, 2H). **^{13}C NMR** (101 MHz, $CDCl_3$) (2 rotamers): δ (ppm) 158.44, 155.97, 152.00, 151.74, 151.14, 150.95, 139.84, 139.81, 139.74, 139.70, 129.52, 129.49, 129.47, 129.41, 127.05, 126.98, 125.80, 125.47, 125.13, 121.75, 121.66, 116.98, 116.78, 107.23, 106.89, 48.30, 47.69, 37.82, 37.70, 29.32, 29.22. **^{19}F NMR** (471 MHz, $CDCl_3$) (2 rotamers): δ (ppm) −117.9, −118.0.

HRMS (ESI): m/z calcd for $C_{18}H_{16}O_2FClN^+$ $[M + H]^+$ 332.0848 found 332.0851.

IR (ν_{max}/cm^{-1}) 1724, 1502, 1407, 1376, 1258, 1202, 860, 821, 751, 690.

SFC Conditions: Chiralpak ID; Gradient 1; 98.5:1.5 er (major enantiomer t_R = 3.39 min; minor enantiomer t_R = 3.48 min), **97% ee. [α]$^{25}_{D}$ =** −46.8 (c = 2.0, $CHCl_3$).

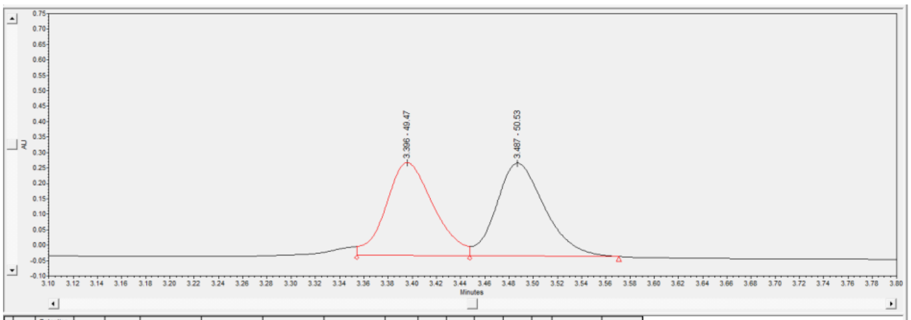

	Name	Retention Time (min)	Purity1 Angle	Purity1 Threshold	PDA/FLR Match1 Spect. Name	PDA/FLR Match1 Angle	PDA/FLR Match1 Threshold	PDA/FLR Match1 Lib. Name	Area (µV*sec)	% Area	Height (µV)	Int Type	Amount	Units	Peak Type	Peak Codes
1		3.396							816333	49.47	300475	vv			Unknown	
2		3.487							833821	50.53	301054	vb			Unknown	

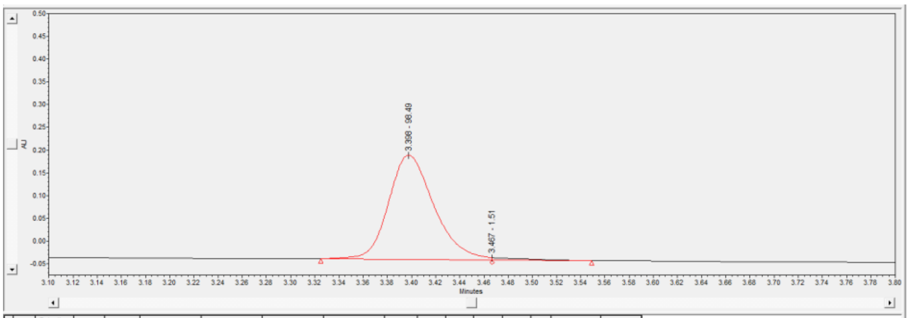

	Name	Retention Time (min)	Purity1 Angle	Purity1 Threshold	PDA/FLR Match1 Spect. Name	PDA/FLR Match1 Angle	PDA/FLR Match1 Threshold	PDA/FLR Match1 Lib. Name	Area (µV*sec)	% Area	Height (µV)	Int Type	Amount	Units	Peak Type	Peak Codes
1		3.398							600405	98.49	229123	bv			Unknown	
2		3.467							9227	1.51	5031	vb			Unknown	I06

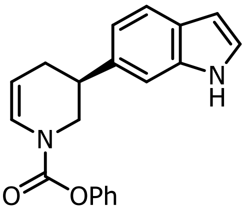

Phenyl-(S)-3-(1H-indol-6-yl)-3,4-dihydropyridine-1(2H)-carboxylate (3af): The corresponding compound was prepared following general procedure **A** using 1H-indol-6-yl boronic acid and phenyl pyridine-1(2H)-carboxylate. The mixture was stirred at 70 °C for 20 hours. Purification by flash chromatography (2% acetone/petrol to 25% acetone/petrol) afforded compound **3af** as viscous liquid (35% yield, 86% ee).

^{1}H NMR ($CDCl_3$, 500 MHz) (2 rotamers): δ (ppm) 8.22 (s, 1H), 7.63 (t, *J* = 7.8 Hz, 1H), 7.45 – 7.30 (m, 2H), 7.30 – 7.13 (m, 5H), 7.15 – 6.99 (m, 2H), 6.53 (s, 1H), 5.31 – 5.11 (m, 1H), 4.38 (dd, *J* = 12.5, 2.9 Hz, 1H), 3.60 – 3.31 (m, 1H), 3.21 (ddt, *J* = 17.6, 11.7, 6.0 Hz, 1H), 2.48 – 2.39 (m, 2H). **^{13}C NMR** (126 MHz, $CDCl_3$) (2 rotamers): δ (ppm) 152.3, 151.9, 151.3, 151.1, 136.7, 136.6, 136.22, 136.2, 129.5, 129.4, 127.05, 126.0, 125.7, 125.6, 125.2, 124.8, 124.6, 124.5, 121.8, 121.7, 121.03, 121.0, 119.6, 119.57, 109.51, 109.5, 108.3, 107.9, 102.51, 102.5, 49.1, 48.5, 39.0, 38.8, 30.0, 29.8.

HRMS (ESI): m/z calcd for $C_{20}H_{19}O_2N_2^+$ [M + H]$^+$ 319.1441 found 319.1435.

IR (ν_{max}/cm^{-1}) 3354, 1706, 1407, 1352, 1203, 1074, 873, 811, 753, 731, 689.

SFC Conditions: Chiralpak IB; Gradient 1; 93:7 er (major enantiomer t_R = 6.42 min; minor enantiomer t_R = 6.22 min), **86% ee**. **[α]$^{25}_{D}$ =** −76.8 (c = 2.0, $CHCl_3$).

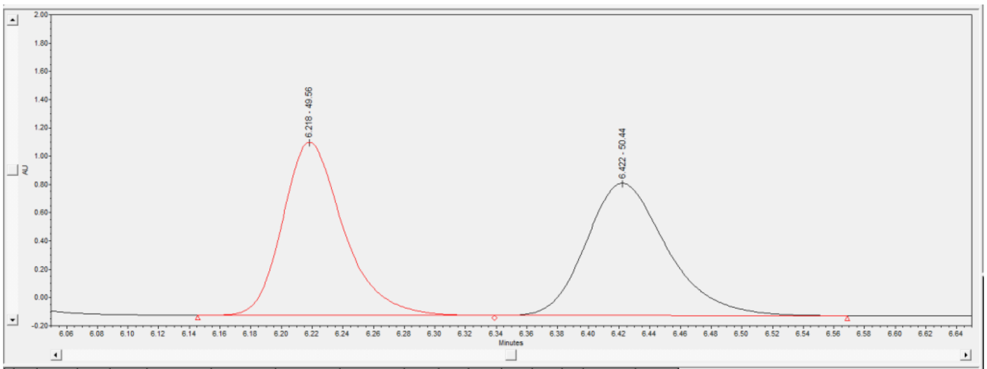

	Name	Retention Time (min)	Purity1 Angle	Purity1 Threshold	PDA/FLR Match1 Spect. Name	PDA/FLR Match1 Angle	PDA/FLR Match1 Threshold	PDA/FLR Match1 Lib. Name	Area (μV*sec)	% Area	Height (μV)	Int Type	Amount	Units	Peak Type	Peak Codes
1		6.218							3366861	49.56	1222750	bv			Unknown	
2		6.422							3426324	50.44	935237	vb			Unknown	

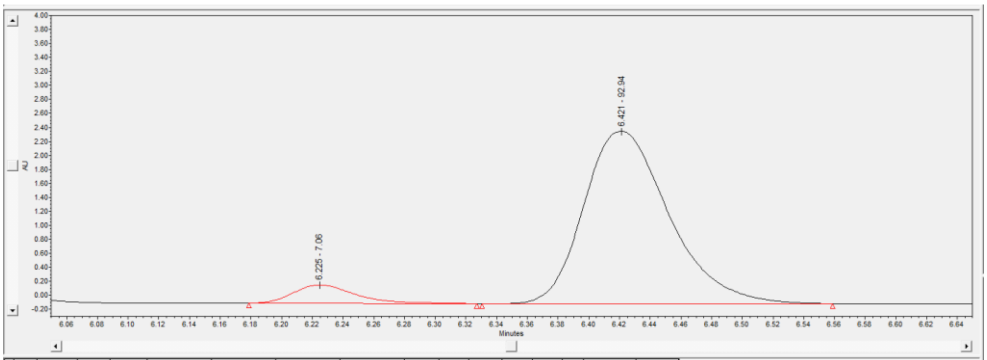

	Name	Retention Time (min)	Purity1 Angle	Purity1 Threshold	PDA/FLR Match1 Spect. Name	PDA/FLR Match1 Angle	PDA/FLR Match1 Threshold	PDA/FLR Match1 Lib. Name	Area (μV*sec)	% Area	Height (μV)	Int Type	Amount	Units	Peak Type	Peak Codes
1		6.225							721449	7.06	261610	bb			Unknown	
2		6.421							9498132	92.94	2465099	bb			Unknown	

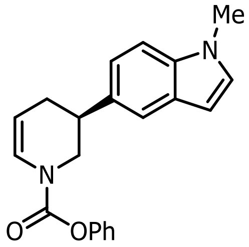

Phenyl-(S)-3-(1-methyl-1H-indol-5-yl)-3,4-dihydropyridine-1(2H)-carboxylate (3ag): The corresponding compound was prepared following general procedure **A** using 1-methyl-1H-indol-5-yl boronic acid and phenyl pyridine-1(2H)-carboxylate. The mixture was stirred at 70 °C for 20 hours. Purification by flash chromatography (2% acetone/petrol to 25% acetone/petrol) afforded compound **3ag** as viscous liquid (43% yield, 98% ee).

^{1}H NMR ($CDCl_3$, 400 MHz) (2 rotamers): δ (ppm) 7.64 – 7.52 (m, 1H), 7.47 – 7.30 (m, 3H), 7.30 – 7.04 (m, 6H), 6.51 (t, J = 3.0 Hz, 1H), 5.35 – 5.16 (m, 1H), 4.42 (td, J = 12.9, 3.6 Hz, 1H), 3.82 – 3.71 (m, 3H), 3.64 – 3.34 (m, 1H), 3.24 (ddt, J = 15.6, 11.6, 5.7 Hz, 1H), 2.47 (dt, J = 9.2, 3.0 Hz, 2H). **^{13}C NMR** (101 MHz, $CDCl_3$) (2 rotamers): δ (ppm) 156.1, 152.2, 151.8, 151.3, 151.1, 136.0, 135.97, 133.7, 133.6, 129.4, 129.38, 125.64, 125.6, 125.1, 124.8, 121.8, 121.7, 121.2, 121.15, 119.05, 119.0, 115.4, 109.6, 109.5, 108.4, 108.0, 100.8, 49.3, 48.7, 38.8, 38.6, 32.9, 30.1, 30.0.

HRMS (ESI): m/z calcd for $C_{21}H_{20}O_2N_2^+$ [M + H]$^+$ 333.1598 found 333.1603.

IR (ν_{max}/cm^{-1}) 1720, 1494, 1406, 1348, 1304, 1247, 1203, 1074, 750, 722, 690.

SFC Conditions: Chiralpak IB; Gradient 2; 99:1 er (major enantiomer t_R = 3.56 min; minor enantiomer t_R = 3.11 min), **98% ee. [α]$^{25}_D$ =** −68.2 (c = 2.0, $CHCl_3$).

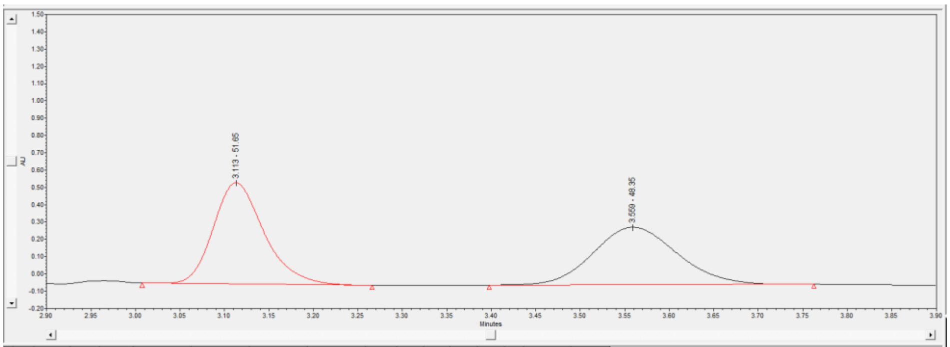

	Name	Retention Time (min)	Purity1 Angle	Purity1 Threshold	PDA/FLR Match1 Spect. Name	PDA/FLR Match1 Angle	PDA/FLR Match1 Threshold	PDA/FLR Match1 Lib. Name	Area (µV*sec)	% Area	Height (µV)	Int Type	Amount	Units	Peak Type	Peak Codes
1		3.113							2325689	51.65	585106	bb			Unknown	
2		3.559							2177179	48.35	331631	bb			Unknown	

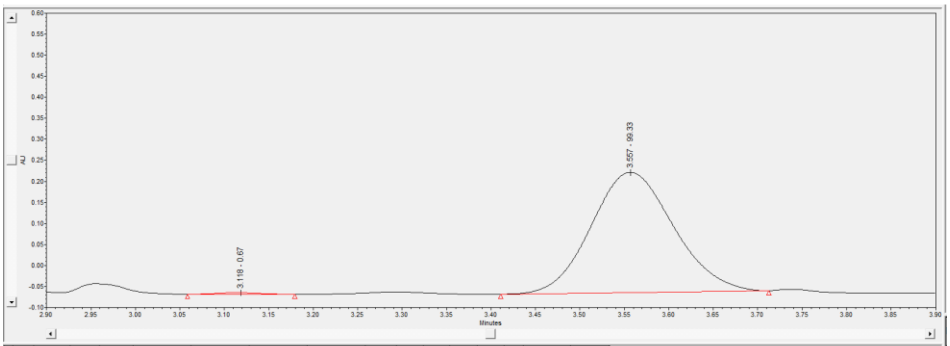

	Name	Retention Time (min)	Purity1 Angle	Purity1 Threshold	PDA/FLR Match1 Spect. Name	PDA/FLR Match1 Angle	PDA/FLR Match1 Threshold	PDA/FLR Match1 Lib. Name	Area (µV*sec)	% Area	Height (µV)	Int Type	Amount	Units	Peak Type	Peak Codes
1		3.118							12413	0.67	3873	bb			Unknown	
2		3.557							1837335	99.33	285665	bb			Unknown	

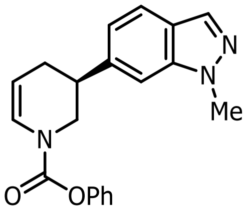

Phenyl-(S)-3-(1-methyl-1H-indazol-6-yl)-3,4-dihydropyridine-1(2H)-carboxylate (3ah): The corresponding compound was prepared following general procedure **A** using 1-methyl-1H-indazol-6-yl boronic acid and phenyl pyridine-1(2H)-carboxylate. The mixture was stirred at 70 °C for 20 hours. Purification by flash chromatography (2% acetone/petrol to 25% acetone/petrol) afforded compound **3ah** as viscous liquid (81% yield, 99% ee).

^{1}H NMR ($CDCl_3$, 400 MHz) (2 rotamers): δ (ppm) 7.96 (d, *J* = 2.2 Hz, 1H), 7.75 – 7.65 (m, 1H), 7.44 – 7.30 (m, 1H), 7.35 – 7.01 (m, 6H), 5.32 – 5.12 (m, 1H), 4.38 (dt, *J* = 12.3, 4.6 Hz, 1H), 3.68 – 3.34 (m, 1H), 3.27 (pd, *J* = 11.1, 3.6 Hz, 1H), 2.52 – 2.40 (m, 2H).**^{13}C NMR** (101 MHz, $CDCl_3$) (2 rotamers): δ (ppm) 152.1, 151.8, 151.2, 151.0, 141.4, 141.3, 140.3, 132.7, 129.51, 129.5, 125.8, 125.7, 125.4, 125.0, 123.32, 123.3, 121.8, 121.6, 121.5, 121.48, 120.6, 107.8, 107.4, 107.1, 107.0, 48.7, 48.1, 39.2, 39.1, 35.6, 29.8, 29.6.

HRMS (ESI): m/z calcd for $C_{20}H_{20}O_2N_3^+$ [M + H]$^+$ 334.1550 found 334.1577.

IR (ν_{max}/cm^{-1}) 1721, 1406, 1367, 1350, 1201, 1098, 973, 839, 750.

SFC Conditions: Chiralpak ID; Gradient 1; 99.5:0.5 er (major enantiomer t_R = 5.86 min; minor enantiomer t_R = 5.56 min), **99% ee. [α]$^{25}_D$ =** −63.8 (c = 2.0, $CHCl_3$).

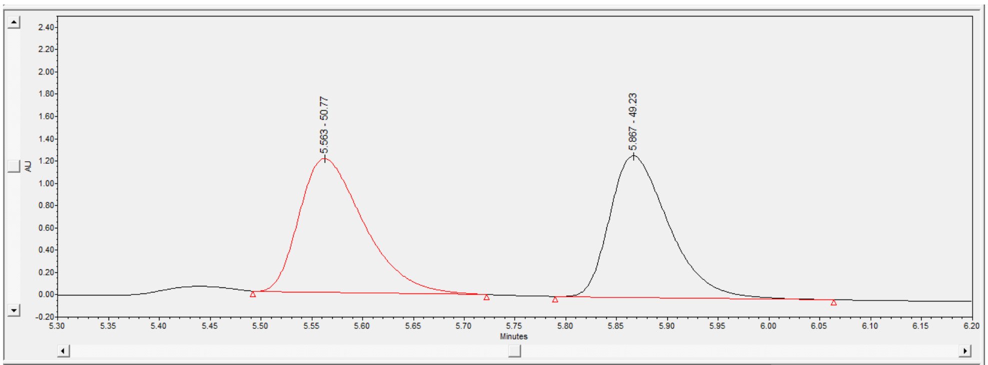

	Name	Retention Time (min)	Purity1 Angle	Purity1 Threshold	PDA/FLR Match1 Spect. Name	PDA/FLR Match1 Angle	PDA/FLR Match1 Threshold	PDA/FLR Match1 Lib. Name	Area (μV*sec)	% Area	Height (μV)	Int Type	Amount	Units	Peak Type	Peak Codes
1		5.563							5302891	50.77	1199769	bb			Unknown	
2		5.867							5141558	49.23	1270331	bb			Unknown	

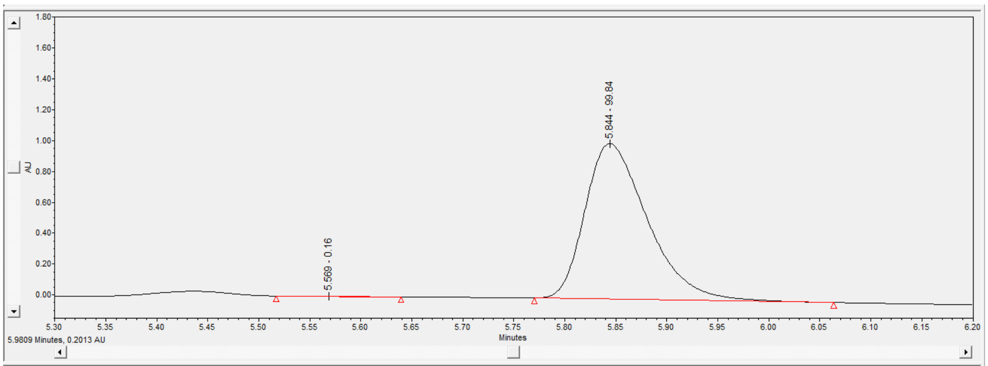

	Name	Retention Time (min)	Purity1 Angle	Purity1 Threshold	PDA/FLR Match1 Spect. Name	PDA/FLR Match1 Angle	PDA/FLR Match1 Threshold	PDA/FLR Match1 Lib. Name	Area (μV*sec)	% Area	Height (μV)	Int Type	Amount	Units	Peak Type	Peak Codes
1		5.569							6796	0.16	2294	bb			Unknown	I08
2		5.844							4293991	99.84	1007227	bb			Unknown	

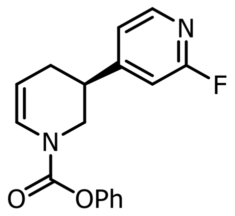

Phenyl-(S)-2'-fluoro-3,4-dihydro-[3,4'-bipyridine]-1(2H)-carboxylate (3ai): The corresponding compound was prepared following general procedure **A** using (6-fluoropyridin-4-yl) boronic acid and phenyl pyridine-1(2H)-carboxylate. The mixture was stirred at 70 °C for 20 hours. Purification by flash chromatography (2% acetone/petrol to 25% acetone/petrol) afforded compound **3ai** as viscous liquid (61% yield, 92% ee).

^{1}H NMR ($CDCl_3$, 500 MHz) (2 rotamers): δ (ppm) 8.19 (t, *J* = 5.7 Hz, 1H), 7.37 (q, *J* = 7.9 Hz, 2H), 7.22 (q, *J* = 7.2 Hz, 1H), 7.16 – 6.96 (m, 4H), 6.83 (d, *J* = 10.4 Hz, 1H), 5.27 – 5.04 (m, 1H), 4.36 – 4.13 (m, 1H), 3.65 – 3.30 (m, 1H), 3.18 (ddp, *J* = 14.6, 9.6, 5.0 Hz, 1H), 2.44 (dt, *J* = 17.5, 5.2 Hz, 1H), 2.37 – 2.27 (m, 1H). **^{13}C NMR** (126 MHz, $CDCl_3$) (2 rotamers): δ (ppm) 165.3, 163.32, 157.5, 157.4, 151.9, 151.7, 151.0, 150.8, 148.1, 148.0, 129.5, 125.9, 125.7, 125.4, 121.7, 121.6, 120.4, 108.3, 108.1, 106.7, 106.3, 47.3, 46.6, 37.9, 37.7, 28.4, 28.3. **^{19}F NMR** (471 MHz, $CDCl_3$) (2 rotamers): δ (ppm) −67.6, −67.8.

HRMS (ESI): m/z calcd for $C_{17}H_{16}O_2N_2^+$ $[M + H]^+$ 299.1190 found 299.1194.

IR (ν_{max}/cm^{-1}) 1722, 1656, 1613, 1567, 1409, 1363, 1257, 1203, 1163, 1077, 751, 690.

SFC Conditions: Chiralpak IC; Gradient 1; 96:4 er (major enantiomer t_R = 6.22 min; minor enantiomer t_R = 6.46 min), **92% ee**. **$[\alpha]^{25}_D$ =** −49.4 (c = 2.0, $CHCl_3$).

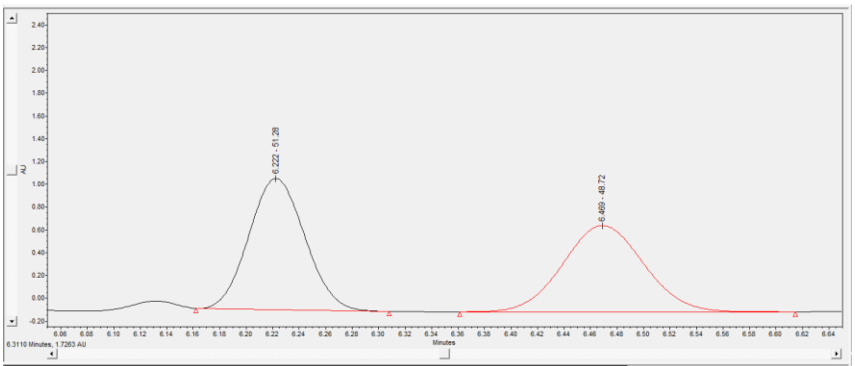

	Name	Retention Time (min)	Purity1 Angle	Purity1 Threshold	PDA/FLR Match1 Spect. Name	PDA/FLR Match1 Angle	PDA/FLR Match1 Threshold	PDA/FLR Match1 Lib. Name	Area (μV*sec)	% Area	Height (μV)	Int Type	Amount	Units	Peak Type	Peak Codes
1		6.222							3391452	51.28	1152931	bb			Unknown	
2		6.469							3222606	48.72	756183	bb			Unknown	

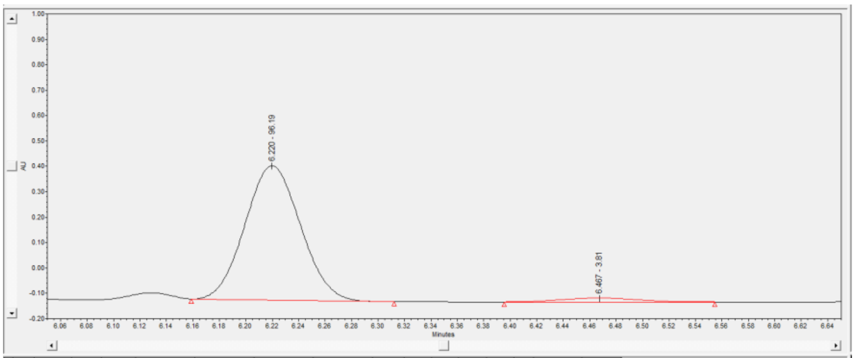

	Name	Retention Time (min)	Purity1 Angle	Purity1 Threshold	PDA/FLR Match1 Spect. Name	PDA/FLR Match1 Angle	PDA/FLR Match1 Threshold	PDA/FLR Match1 Lib. Name	Area (μV*sec)	% Area	Height (μV)	Int Type	Amount	Units	Peak Type	Peak Codes
1		6.220							1545217	96.19	529045	bb			Unknown	
2		6.467							61196	3.81	15165	bb			Unknown	

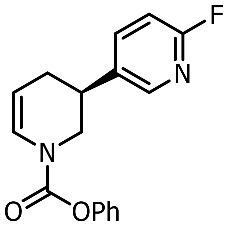

Phenyl-(S)-6'-fluoro-3,4-dihydro-[3,3'-bipyridine]-1(2H)-carboxylate (3aj): The corresponding compound was prepared following general procedure **A** using (6-fluoropyridin-3-yl) boronic acid and phenyl pyridine-1(2H)-carboxylate. The mixture was stirred at 70 °C for 20 hours. Purification by flash chromatography (2% acetone/petrol to 25% acetone/petrol) afforded compound **3ai** as viscous liquid (38% yield, 96% ee).

^{1}H NMR ($CDCl_3$, 500 MHz) (2 rotamers): δ (ppm) 8.15 (d, *J* = 11.9 Hz, 1H), 7.67 (qd, *J* = 9.4, 2.4 Hz, 1H), 7.37 (q, *J* = 8.6 Hz, 2H), 7.23 (t, *J* = 7.5 Hz, 1H), 7.17 – 7.01 (m, 3H), 6.94 (dt, *J* = 8.3, 3.9 Hz, 1H), 5.27 – 5.08 (m, 1H), 4.24 (t, *J* = 14.2 Hz, 1H), 3.69 – 3.32 (m, 1H), 3.18 (ddq, *J* = 15.3, 9.9, 4.8 Hz, 1H), 2.43 (dt, *J* = 17.5, 5.2 Hz, 1H), 2.31 (ddt, *J* = 17.5, 9.9, 2.5 Hz, 1H). **^{13}C NMR** (126 MHz, $CDCl_3$) (2 rotamers): δ (ppm) 151.8, 151.1, 150.9, 146.7, 146.6, 139.9, 139.87, 135.9, 135.7, 129.6, 129.5, 125.9, 125.7, 125.3, 121.7, 121.6, 109.8, 107.0, 106.6, 48.1, 47.5, 35.4, 29.2. **^{19}F NMR** (471 MHz, $CDCl_3$) (2 rotamers): δ (ppm) −70.2, −70.4.

HRMS (ESI): m/z calcd for $C_{17}H_{16}O_2N_2^+$ $[M + H]^+$ 299.1190 found 299.1244.

IR (ν_{max}/cm^{-1}) 1727, 1657, 1598, 1489, 1409, 1376, 1255, 1205, 1078, 855, 751, 751, 690.

SFC Conditions: Chiralpak IB; Gradient 1; 98:2 er (major enantiomer t_R = 4.22 min; minor enantiomer t_R = 3.97 min), **96% ee**. **$[\alpha]^{25}_D$ =** −46.3 (c = 2.0, $CHCl_3$).

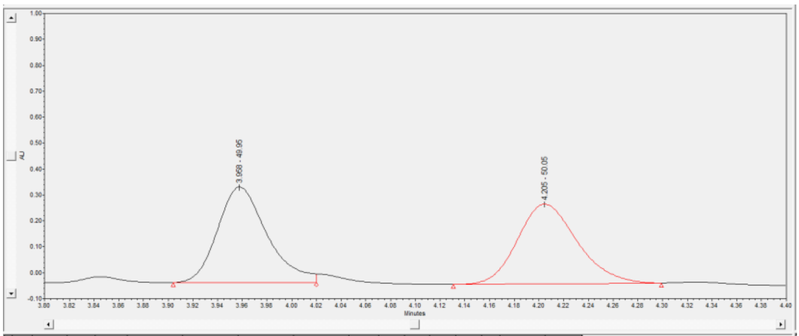

	Name	Retention Time (min)	Purity1 Angle	Purity1 Threshold	PDA/FLR Match1 Spect. Name	PDA/FLR Match1 Angle	PDA/FLR Match1 Threshold	PDA/FLR Match1 Lib. Name	Area (µV*sec)	% Area	Height (µV)	Int Type	Amount	Units	Peak Type	Peak Codes
1		3.958							1027172	49.95	368598	bv			Unknown	
2		4.205							1029237	50.05	307256	bb			Unknown	

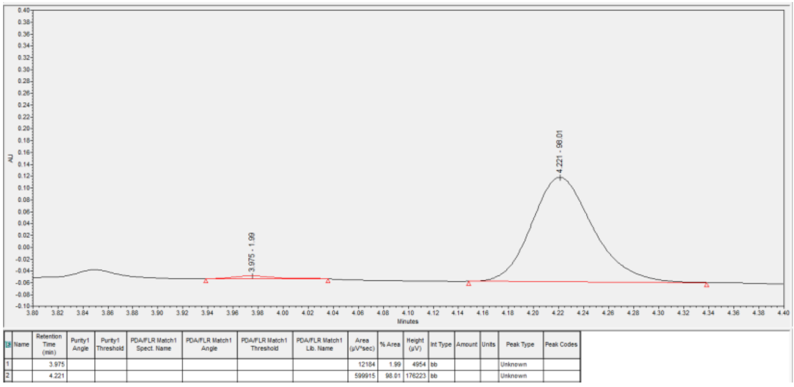

	Name	Retention Time (min)	Purity1 Angle	Purity1 Threshold	PDA/FLR Match1 Spect. Name	PDA/FLR Match1 Angle	PDA/FLR Match1 Threshold	PDA/FLR Match1 Lib. Name	Area (µV*sec)	% Area	Height (µV)	Int Type	Amount	Units	Peak Type	Peak Codes
1		3.975							12184	1.99	4954	bb			Unknown	
2		4.221							599915	98.01	176223	bb			Unknown	

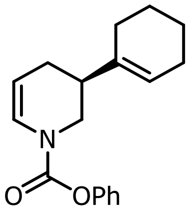

Phenyl-(S)-3-(cyclohex-1-en-1-yl)-3,4-dihydropyridine-1(2H)-carboxylate (3ak): The corresponding compound was prepared following general procedure **A** using cyclohex-1-en-1-yl boronic acid and phenyl pyridine-1(2H)-carboxylate. The mixture was stirred at 70 °C for 20 hours. Purification by flash chromatography (100% petrol to 5% acetone/petrol) afforded compound **3ak** as viscous liquid (77% yield, 99% ee).

^{1}H NMR ($CDCl_3$, 400 MHz) (2 rotamers): δ (ppm) 7.37 (tt, J = 7.6, 2.2 Hz, 2H), 7.25 – 7.19 (m, 1H), 7.16 – 7.11 (m, 1H), 7.02 – 6.86 (m, 1H), 5.56 – 5.46 (m, 1H), 5.16 – 5.00 (m, 1H), 4.23 – 4.10 (m, 1H), 3.35 – 2.96 (m, 1H), 2.34 (ddd, J = 15.1, 8.8, 3.5 Hz, 1H), 2.24 – 1.85 (m, 6H), 1.72 – 1.52 (m, 4H). **^{13}C NMR** (101 MHz, $CDCl_3$) (2 rotamers): δ (ppm) 152.2, 151.8, 151.3, 151.2, 138.1, 138.0, 129.4, 125.6, 125.0, 124.6, 122.0, 121.9, 121.8, 108.0, 107.7, 47.0, 46.5, 39.64, 39.6, 29.8, 27.3, 27.1, 27.0, 25.3, 23.1, 22.6.

HRMS (ESI): m/z calcd for $C_{18}H_{21}O_2NNa^+$ [M + Na]$^+$ 306.1465 found 306.1468.

IR (ν_{max}/cm^{-1}) 1726, 1656, 1407, 1361, 1252, 1205, 750, 689.

SFC Conditions: Chiralpak IF; Gradient 1; 99.5:0.5 er (major enantiomer t_R = 3.94 min; minor enantiomer t_R = 4.16 min), **99% ee. [α]$^{25}_D$ =** −48.8 (c = 2.0, $CHCl_3$).

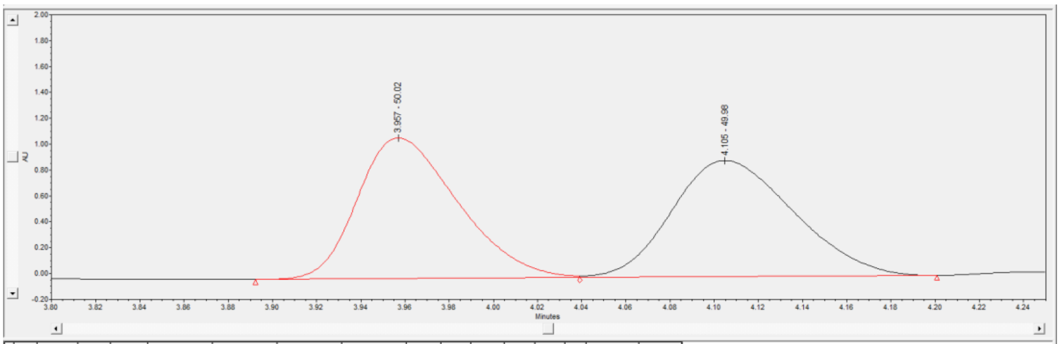

	Name	Retention Time (min)	Purity1 Angle	Purity1 Threshold	PDA/FLR Match1 Spect. Name	PDA/FLR Match1 Angle	PDA/FLR Match1 Threshold	PDA/FLR Match1 Lib. Name	Area (µV*sec)	% Area	Height (µV)	Int Type	Amount	Units	Peak Type	Peak Codes
1		3.957							3447843	50.02	1089949	bv			Unknown	
2		4.105							3445712	49.98	899446	vb			Unknown	

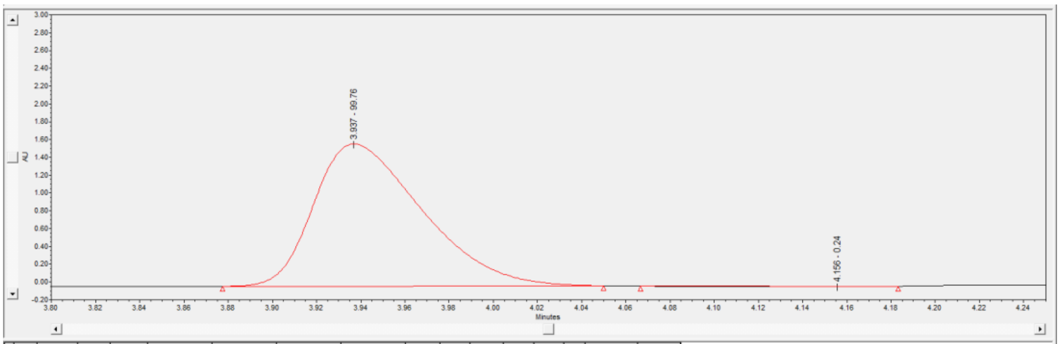

	Name	Retention Time (min)	Purity1 Angle	Purity1 Threshold	PDA/FLR Match1 Spect. Name	PDA/FLR Match1 Angle	PDA/FLR Match1 Threshold	PDA/FLR Match1 Lib. Name	Area (µV*sec)	% Area	Height (µV)	Int Type	Amount	Units	Peak Type	Peak Codes
1		3.937							5433903	99.76	1595295	bb			Unknown	
2		4.156							13240	0.24	-4075	bb			Unknown	

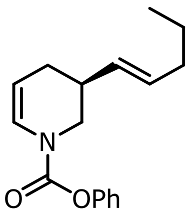

Phenyl-(S,E)-3-(pent-1-en-1-yl)-3,4-dihydropyridine-1(2H)-carboxylate (3al): The corresponding compound was prepared following general procedure **A** using pent-1-en-1-yl boronic acid and phenyl pyridine-1(2H)-carboxylate. The mixture was stirred at 70 °C for 20 hours. Purification by flash chromatography (100% petrol to 5% acetone/petrol) afforded compound **3al** as viscous liquid (62% yield, 99% ee).

^{1}H NMR ($CDCl_3$, 400 MHz) (2 rotamers): δ (ppm) 7.37 (t, *J* = 8.0 Hz, 2H), 7.22 (t, *J* = 7.4 Hz, 1H), 7.13 (d, *J* = 8.0 Hz, 2H), 7.02 – 6.85 (m, 1H), 5.65 – 5.51 (m, 1H), 5.39 (dt, *J* = 15.3, 7.4 Hz, 1H), 5.11 – 4.96 (m, 1H), 4.05 (td, *J* = 14.4, 2.8 Hz, 1H), 3.38 – 3.02 (m, 1H), 2.64 – 2.42 (m, 1H), 2.20 (dt, *J* = 17.5, 5.0 Hz, 1H), 2.06 – 1.90 (m, 3H), 1.50 – 1.31 (m, 2H), 0.90 (td, *J* = 7.4, 3.5 Hz, 3H). **^{13}C NMR** (101 MHz, $CDCl_3$) (2 rotamers): δ (ppm) 152.3, 151.9, 151.3, 151.1, 131.64, 131.6, 130.9, 130.8, 129.5, 125.6, 125.1, 124.8, 121.8, 121.78, 107.2, 106.9, 47.8, 47.2, 35.3, 35.2, 34.8, 29.8, 28.3, 28.2, 22.6, 13.7.

HRMS (ESI): m/z calcd for $C_{17}H_{21}O_2NNa^+$ $[M + Na]^+$ 294.1465 found 294.1471s.

IR (ν_{max}/cm^{-1}) 1728, 1408, 1364, 1204, 972, 750, 689.

SFC Conditions: Chiralpak IA; Gradient 1; 99.5:0.5 er (major enantiomer t_R = 2.36 min; minor enantiomer t_R = 2.25 min), **99% ee**. **$[\alpha]^{25}_D$ =** −34.2 (c = 2.0, $CHCl_3$).

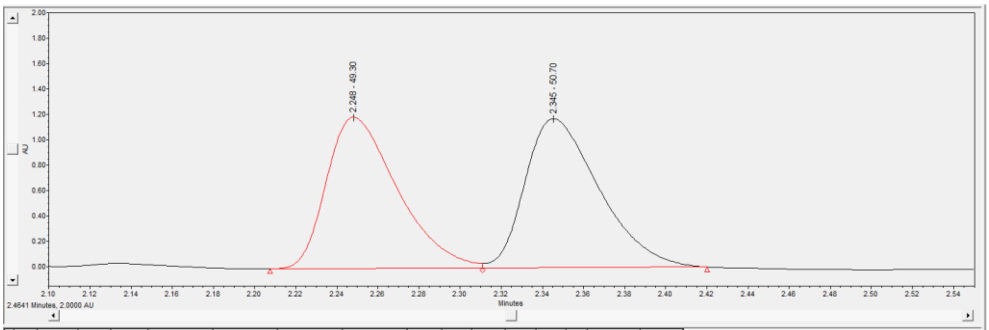

	Name	Retention Time (min)	Purity1 Angle	Purity1 Threshold	PDA/FLR Match1 Spect. Name	PDA/FLR Match1 Angle	PDA/FLR Match1 Threshold	PDA/FLR Match1 Lib. Name	Area (µV*sec)	% Area	Height (µV)	Int Type	Amount	Units	Peak Type	Peak Codes
1		2.248							2855597	49.30	1191002	bv			Unknown	
2		2.345							2936190	50.70	1172415	vb			Unknown	

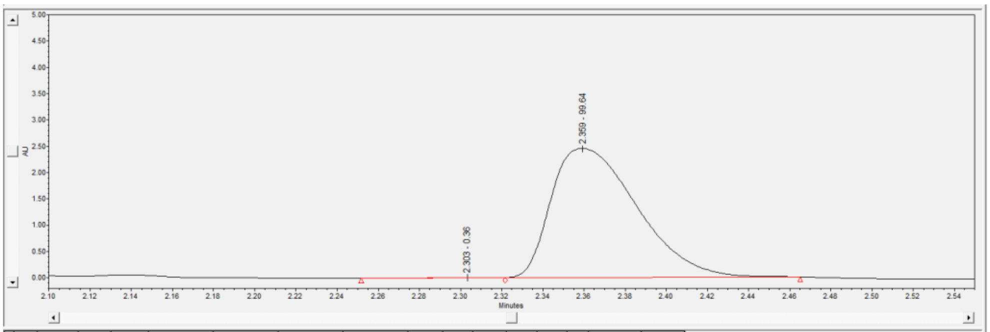

	Name	Retention Time (min)	Purity1 Angle	Purity1 Threshold	PDA/FLR Match1 Spect. Name	PDA/FLR Match1 Angle	PDA/FLR Match1 Threshold	PDA/FLR Match1 Lib. Name	Area (µV*sec)	% Area	Height (µV)	Int Type	Amount	Units	Peak Type	Peak Codes
1		2.303							25775	0.36	13270	bv			Unknown	I08
2		2.359							7134859	99.64	2456277	vb			Unknown	

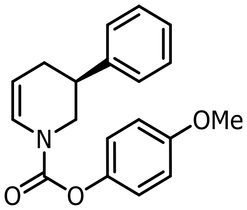

4-Methoxyphenyl-(S)-3-phenyl-3,4-dihydropyridine-1(2H)-carboxylate (3am): The corresponding compound was prepared following general procedure **A** using phenyl boronic acid and 4-methoxyphenyl pyridine-1(2H)-carboxylate. The mixture was stirred at 70 °C for 20 hours. Purification by flash chromatography (2% acetone/petrol to 25% acetone/petrol) afforded compound **3am** as viscous liquid (85% yield, 98% ee).

^{1}H NMR ($CDCl_3$, 400 MHz) (2 rotamers): δ (ppm) 7.36 – 7.16 (m, 5H), 7.10 – 6.92 (m, 3H), 6.90 – 6.78 (m, 2H), 5.21 – 5.05 (m, 1H), 4.31 – 4.20 (m, 1H), 3.77 – 3.70 (m, 3H), 3.50 – 3.17 (m, 1H), 3.05 (ddq, J = 14.3, 7.4, 3.7 Hz, 1H), 2.40 – 2.23 (m, 2H). **^{13}C NMR** (126 MHz, $CDCl_3$) (2 rotamers): δ (ppm) 157.2, 152.5, 152.1, 144.7, 144.6, 142.8, 142.7, 128.9, 128.8, 127.3, 127.1, 127.06, 125.3, 125.0, 122.6, 122.5, 114.51, 114.5, 107.7, 107.3, 55.7, 48.5, 48.0, 38.7, 38.6, 29.5, 29.3.

HRMS (ESI): m/z calcd for $C_{19}H_{19}O_3NNa^+$ $[M + Na]^+$ 332.1257 found 332.1257.

IR (ν_{max}/cm^{-1}) 1721, 1508, 1406, 1362, 1249, 1198, 1070, 1034, 974, 862, 817, 757, 701.

SFC Conditions: Chiralpak IB; Gradient 1; 99:1 er (major enantiomer t_R = 4.10 min; minor enantiomer t_R = 3.94 min), **98% ee. [α]$^{25}_D$ =** −46.8 (c = 2.0, $CHCl_3$).

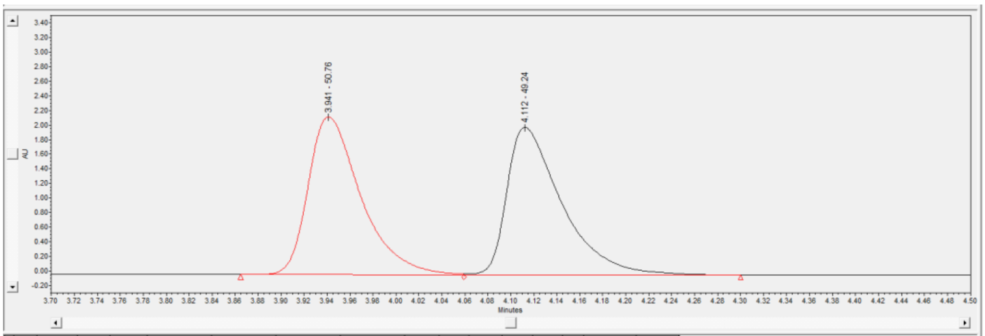

	Name	Retention Time (min)	Purity1 Angle	Purity1 Threshold	PDA/FLR Match1 Spect. Name	PDA/FLR Match1 Angle	PDA/FLR Match1 Threshold	PDA/FLR Match1 Lib. Name	Area (μV*sec)	% Area	Height (μV)	Int Type	Amount	Units	Peak Type	Peak Codes
1		3.941							6895303	50.76	2158908	bv			Unknown	
2		4.112							6690136	49.24	2019564	vb			Unknown	

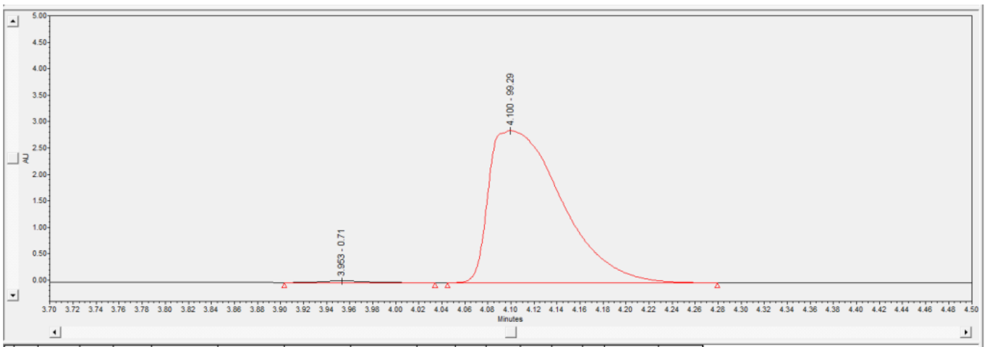

	Name	Retention Time (min)	Purity1 Angle	Purity1 Threshold	PDA/FLR Match1 Spect. Name	PDA/FLR Match1 Angle	PDA/FLR Match1 Threshold	PDA/FLR Match1 Lib. Name	Area (μV*sec)	% Area	Height (μV)	Int Type	Amount	Units	Peak Type	Peak Codes
1		3.953							90741	0.71	33513	bb			Unknown	
2		4.100							12621692	99.29	2881076	bb			Unknown	

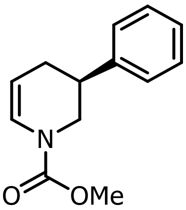

Methyl-(S)-3-phenyl-3,4-dihydropyridine-1(2H)-carboxylate (3an): The corresponding compound was prepared following general procedure **A** using phenyl boronic acid and methyl pyridine-1(2H)-carboxylate. The mixture was stirred at 70 °C for 20 hours. Purification by flash chromatography (2% acetone/petrol to 15% acetone/petrol) afforded compound **3an** as viscous liquid (72% yield, 99% ee).

^{1}H NMR ($CDCl_3$, 500 MHz) (2 rotamers): δ (ppm) 7.43 – 7.16 (m, 5H), 7.07 – 6.80 (m, 1H), 5.23 – 4.95 (m, 1H), 4.34 – 4.03 (m, 1H), 3.86 – 3.72 (m, 3H), 3.32 – 3.20 (m, 1H), 3.01 (ddt, *J* = 15.2, 6.3, 3.8 Hz, 1H), 2.32 (dt, *J* = 9.8, 2.5 Hz, 2H). **^{13}C NMR** (126 MHz, $CDCl_3$) (2 rotamers): δ (ppm) δ 154.1, 153.8, 143.1, 142.9, 128.8, 127.3, 127.0, 125.4, 125.0, 106.4, 106.2, 53.1, 48.0, 47.7, 38.7, 38.6, 29.4, 29.3.

HRMS (ESI): m/z calcd for $C_{13}H_{15}O_2N^+$ $[M + H]^+$ 218.1176 found 218.1181.

IR (ν_{max}/cm^{-1}) 1704, 1447, 1405, 1355, 1268, 1204, 1121, 1052, 980, 762, 701.

SFC Conditions: Chiralpak IC; Gradient 1; 99.5:0.5 er (major enantiomer t_R = 2.82 min; minor enantiomer t_R = 2.92 min), **99% ee. $[\alpha]^{25}_D$ =** −30.7 (c = 2.0, $CHCl_3$).

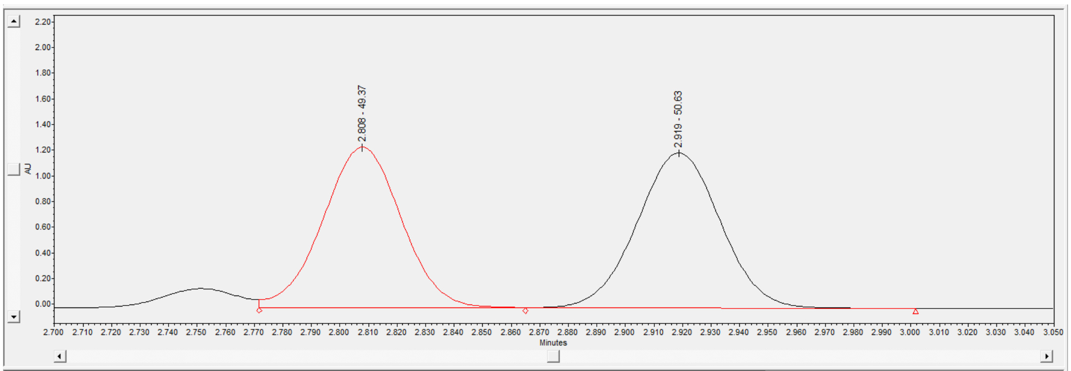

	Name	Retention Time (min)	Purity1 Angle	Purity1 Threshold	PDA/FLR Match1 Spect. Name	PDA/FLR Match1 Angle	PDA/FLR Match1 Threshold	PDA/FLR Match1 Lib. Name	Area (µV*sec)	% Area	Height (µV)	Int Type	Amount	Units	Peak Type	Peak Codes
1		2.808							2410349	49.37	1251839	vv			Unknown	
2		2.919							2471549	50.63	1210358	vb			Unknown	

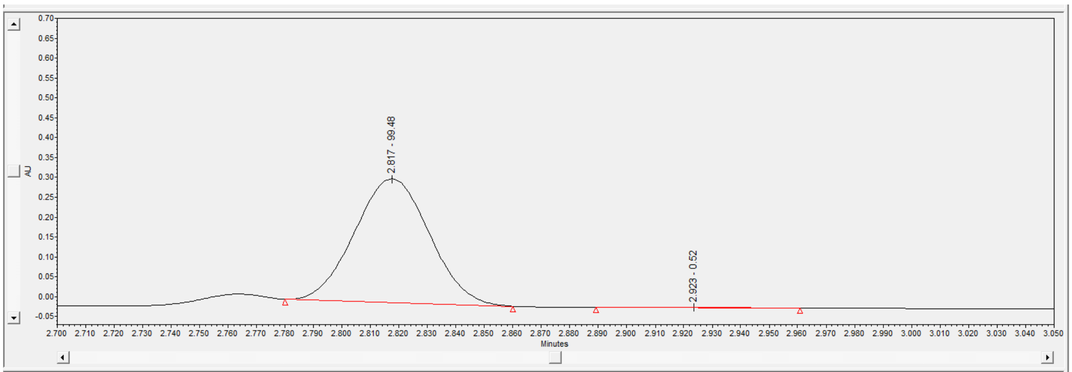

	Name	Retention Time (min)	Purity1 Angle	Purity1 Threshold	PDA/FLR Match1 Spect. Name	PDA/FLR Match1 Angle	PDA/FLR Match1 Threshold	PDA/FLR Match1 Lib. Name	Area (µV*sec)	% Area	Height (µV)	Int Type	Amount	Units	Peak Type	Peak Codes
1		2.817							569341	99.48	311510	bb			Unknown	
2		2.923							2971	0.52	1588	bb			Unknown	

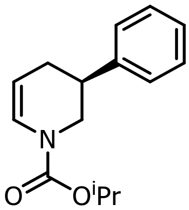

Isopropyl-(S)-3-phenyl-3,4-dihydropyridine-1(2H)-carboxylate (3ao): The corresponding compound was prepared following general procedure **A** using phenyl boronic acid and isopropyl pyridine-1(2H)-carboxylate. The mixture was stirred at 70 °C for 20 hours. Purification by flash chromatography (2% acetone/petrol to 15% acetone/petrol) afforded compound **3ao** as viscous liquid (86% yield, 97% ee).

^{1}H NMR ($CDCl_3$, 400 MHz) (2 rotamers): δ (ppm) 7.40 – 7.13 (m, 5H), 7.04 – 6.82 (m, 1H), 5.14 – 5.04 (m, 2H), 4.30 – 4.00 (m, 1H), 3.22 (dt, J = 23.4, 12.0 Hz, 1H), 2.99 (tdd, J = 10.4, 6.5, 3.7 Hz, 1H), 2.38 – 2.20 (m, 2H), 1.37 – 1.18 (m, 6H). **^{13}C NMR** (101 MHz, $CDCl_3$) (2 rotamers): δ (ppm) 153.3, 153.0, 143.3, 143.1, 128.8, 128.7, 127.4, 127.3, 127.0, 126.9, 125.5, 125.2, 106.0, 105.7, 69.5, 47.9, 47.5, 38.8, 38.7, 29.6, 22.3.

HRMS (ESI): m/z calcd for $C_{15}H_{20}O_2N^+$ $[M + H]^+$ 246.1489 found 246.1493.

IR (ν_{max}/cm^{-1}) 1702, 1413, 1385, 1320, 1266, 1180, 1110, 924, 759, 701.

SFC Conditions: Chiralpak IC; Gradient 1; 98.5:1.5 er (major enantiomer t_R = 2.41 min; minor enantiomer t_R = 2.58 min), **97% ee. $[\alpha]^{25}_D$ =** −27.0 (c = 2.0, $CHCl_3$).

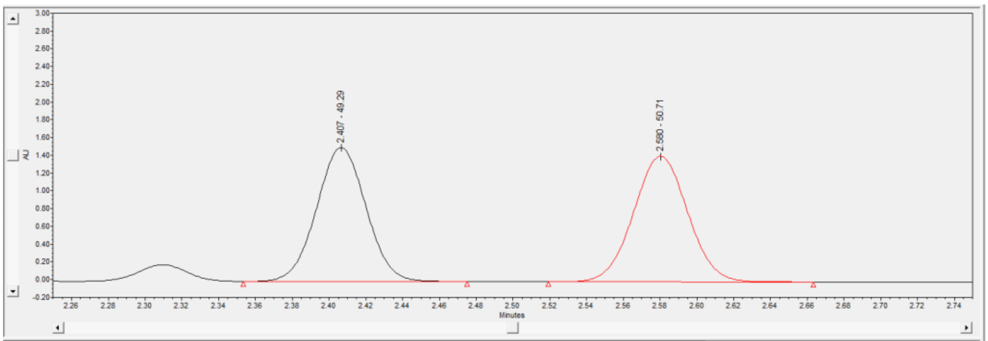

	Name	Retention Time (min)	Purity1 Angle	Purity1 Threshold	PDA/FLR Match1 Spect. Name	PDA/FLR Match1 Angle	PDA/FLR Match1 Threshold	PDA/FLR Match1 Lib. Name	Area (µV*sec)	% Area	Height (µV)	Int Type	Amount	Units	Peak Type	Peak Codes
1		2.407							2823476	49.29	1508285	bb			Unknown	
2		2.580							2904594	50.71	1414796	bb			Unknown	

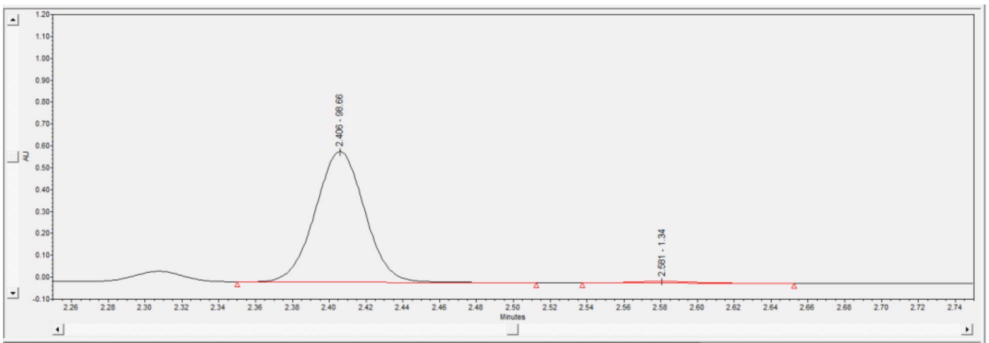

	Name	Retention Time (min)	Purity1 Angle	Purity1 Threshold	PDA/FLR Match1 Spect. Name	PDA/FLR Match1 Angle	PDA/FLR Match1 Threshold	PDA/FLR Match1 Lib. Name	Area (µV*sec)	% Area	Height (µV)	Int Type	Amount	Units	Peak Type	Peak Codes
1		2.406							1113438	98.66	596809	bb			Unknown	
2		2.581							15131	1.34	7193	bb			Unknown	

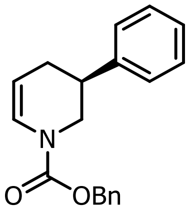

Benzyl-(S)-3-phenyl-3,4-dihydropyridine-1(2H)-carboxylate (3ap): The corresponding compound was prepared following general procedure **A** using phenyl boronic acid and benzyl pyridine-1(2H)-carboxylate. The mixture was stirred at 70 °C for 20 hours. Purification by flash chromatography (2% acetone/petrol to 15% acetone/petrol) afforded compound **3ap** as viscous liquid (68% yield, 99% ee).

^{1}H NMR ($CDCl_3$, 400 MHz) (2 rotamers): δ (ppm) 7.46 – 7.19 (m, 10H), 7.09 – 6.89 (m, 1H), 5.29 – 4.97 (m, 3H), 4.34 – 4.08 (m, 1H), 3.31 (dt, *J* = 23.9, 11.8 Hz, 1H), 3.10 – 2.97 (m, 1H), 2.33 (d, *J* = 7.2 Hz, 2H). **^{13}C NMR** (101 MHz, $CDCl_3$) (2 rotamers): δ (ppm) 153.6, 153.2, 143.1, 142.9, 136.4, 128.8, 128.7, 128.4, 128.3, 128.2, 127.4, 127.3, 127.1, 127.0, 125.5, 125.0, 106.7, 106.4, 67.8, 67.7, 48.0, 47.8, 38.7, 38.6, 29.5.

HRMS (ESI): m/z calcd for $C_{19}H_{20}O_2N^+$ $[M + H]^+$ 294.1489 found 294.1488.

IR (ν_{max}/cm^{-1}) 1704, 1411, 1342, 1265, 1115, 757, 699.

SFC Conditions: Chiralpak IF; Gradient 1; 99.5:0.5 er (major enantiomer t_R = 3.81 min; minor enantiomer t_R = 4.02 min), **99% ee.** **$[\alpha]^{25}_D$ =** −23.5 (c = 2.0, $CHCl_3$).

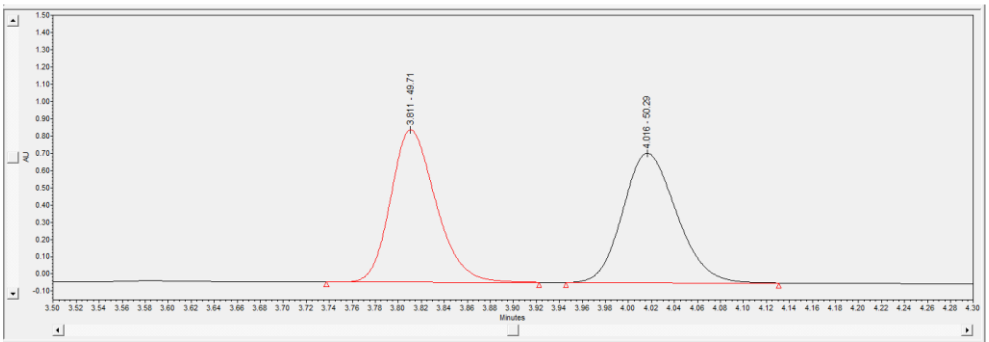

	Name	Retention Time (min)	Purity1 Angle	Purity1 Threshold	PDA/FLR Match1 Spect. Name	PDA/FLR Match1 Angle	PDA/FLR Match1 Threshold	PDA/FLR Match1 Lib. Name	Area (µV*sec)	% Area	Height (µV)	Int Type	Amount	Units	Peak Type	Peak Codes
1		3.811							2405526	49.71	883635	bb			Unknown	
2		4.016							2433229	50.29	750175	bb			Unknown	

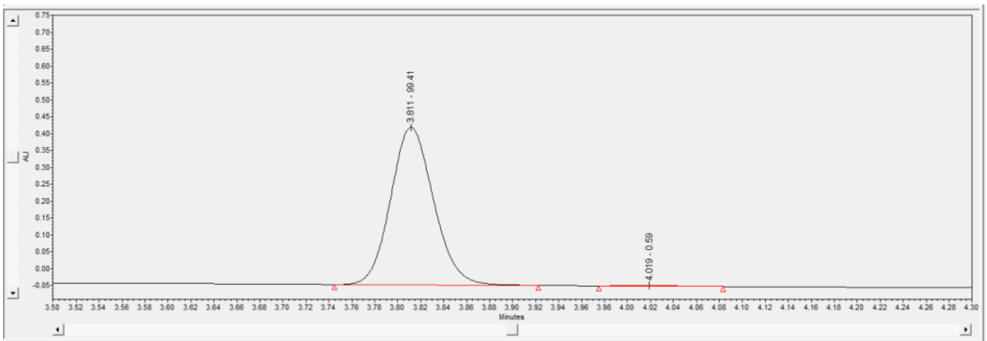

	Name	Retention Time (min)	Purity1 Angle	Purity1 Threshold	PDA/FLR Match1 Spect. Name	PDA/FLR Match1 Angle	PDA/FLR Match1 Threshold	PDA/FLR Match1 Lib. Name	Area (µV*sec)	% Area	Height (µV)	Int Type	Amount	Units	Peak Type	Peak Codes
1		3.811							1210281	99.41	465871	bb			Unknown	
2		4.019							7133	0.59	2394	bb			Unknown	I08

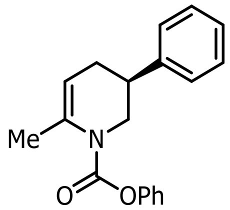

Phenyl-(S)-6-methyl-3-phenyl-3,4-dihydropyridine-1(2H)-carboxylate (3aq): The corresponding compound was prepared following general procedure **A** using phenyl boronic acid and phenyl 6-methylpyridine-1(2H)-carboxylate. The mixture was stirred at 70 °C for 20 hours. Purification by flash chromatography (2% acetone/petrol to 15% acetone/petrol) afforded compound **3aq** as viscous liquid (57% yield, 91% ee).

^{1}H NMR ($CDCl_3$, 400 MHz) (2 rotamers): δ (ppm) 7.39 – 7.24 (m, 8H), 7.22 – 7.14 (m, 1H), 6.94 (d, *J* = 7.8 Hz, 1H), 5.23 – 5.16 (m, 1H), 4.19 (dd, *J* = 12.5, 3.0 Hz, 1H), 3.66 – 3.54 (m, 1H), 3.19 (qd, *J* = 8.4, 3.2 Hz, 1H), 2.51 (dddd, *J* = 18.3, 6.1, 4.0, 2.0 Hz, 1H), 2.41 – 2.28 (m, 1H), 2.24 (q, *J* = 2.0 Hz, 3H). **^{13}C NMR** (101 MHz, $CDCl_3$) (2 rotamers): δ (ppm) 152.8, 151.1, 143.3, 135.8, 129.3, 128.8, 127.3, 126.9, 125.5, 121.8, 112.8, 50.8, 39.7, 30.6, 22.0.

HRMS (ESI): m/z calcd for $C_{19}H_{19}O_2NNa^+$ $[M + Na]^+$ 316.1308 found 316.1308.

IR (ν_{max}/cm^{-1}) 1722, 1493, 1385, 1204, 752, 702, 755, 701.

SFC Conditions: Chiralpak IC; Gradient 1; 95.5:4.5 er (major enantiomer t_R = 3.86 min; minor enantiomer t_R = 3.53 min), **91% ee**. **$[\alpha]^{25}_D$ =** −40.7 (c = 2.0, $CHCl_3$).

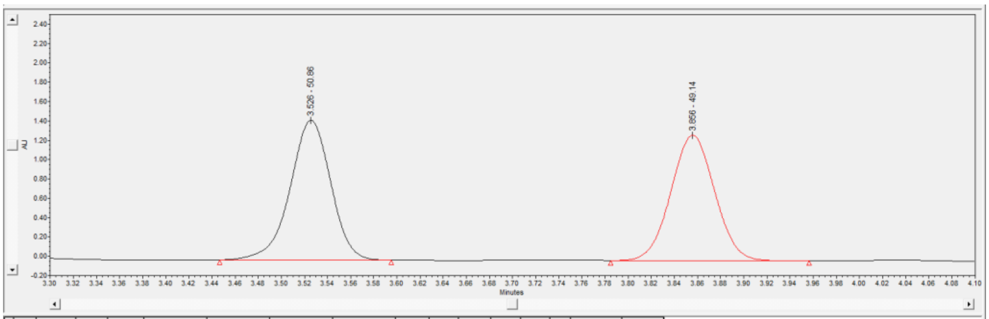

	Name	Retention Time (min)	Purity1 Angle	Purity1 Threshold	PDA/FLR Match1 Spect. Name	PDA/FLR Match1 Angle	PDA/FLR Match1 Threshold	PDA/FLR Match1 Lib. Name	Area (µV*sec)	% Area	Height (µV)	Int Type	Amount	Units	Peak Type	Peak Codes
1		3.526							3533186	50.86	1445404	bb			Unknown	
2		3.856							3413785	49.14	1297495	bb			Unknown	

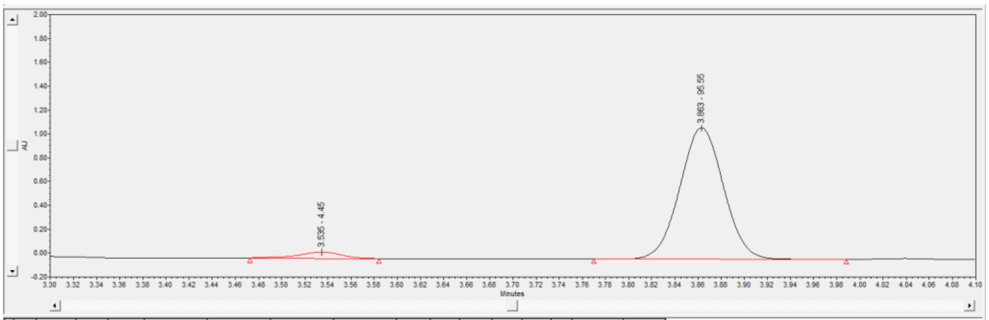

	Name	Retention Time (min)	Purity1 Angle	Purity1 Threshold	PDA/FLR Match1 Spect. Name	PDA/FLR Match1 Angle	PDA/FLR Match1 Threshold	PDA/FLR Match1 Lib. Name	Area (µV*sec)	% Area	Height (µV)	Int Type	Amount	Units	Peak Type	Peak Codes
1		3.535							134587	4.45	52571	bb			Unknown	
2		3.863							2891258	95.55	1100724	bb			Unknown	

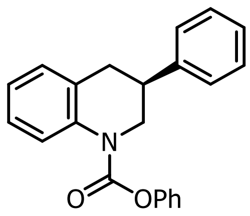

Phenyl-(S)-3-phenyl-3,4-dihydroquinoline-1(2H)-carboxylate (3as): The corresponding compound was prepared following general procedure **A** using phenyl boronic acid and phenyl quinoline-1(2H)-carboxylate. The mixture was stirred at 30 °C for 48 hours. Purification by flash chromatography (2% acetone/petrol to 15% acetone/petrol) afforded compound **3as** as viscous liquid (85% yield, 90% ee).

^{1}H NMR ($CDCl_3$, 400 MHz) (2 rotamers): δ (ppm) 7.87 (d, *J* = 7.7 Hz, 1H), 7.41 – 7.28 (m, 7H), 7.22 (ddd, *J* = 9.0, 6.5, 4.5 Hz, 3H), 7.15 – 7.03 (m, 3H), 4.36 (ddd, *J* = 12.7, 4.1, 1.2 Hz, 1H), 3.89 – 3.79 (m, 1H), 3.33 (tt, *J* = 9.6, 4.9 Hz, 1H), 3.23 (dd, *J* = 16.4, 5.7 Hz, 1H), 3.12 (dd, *J* = 16.4, 9.6 Hz, 1H). **^{13}C NMR** (101 MHz, $CDCl_3$) (2 rotamers): δ (ppm) 153.3, 151.2, 142.6, 137.8, 129.4, 129.0, 128.9, 127.4, 127.2, 126.4, 125.6, 124.4, 124.1, 121.8, 51.1, 40.3, 34.7.

HRMS (ESI): m/z calcd for $C_{22}H_{20}O_2N^+$ $[M + H]^+$ 330.1489 found 330.1491.

IR (ν_{max}/cm^{-1}) 1722, 1492, 1380, 1197, 1130, 755, 701.

SFC Conditions: Chiralpak IC; Gradient 1; 95:5 er (major enantiomer t_R = 5.11 min; minor enantiomer t_R = 4.74 min), **90% ee**. **$[\alpha]^{25}_D$ =** −7.9 (c = 2.0, $CHCl_3$).

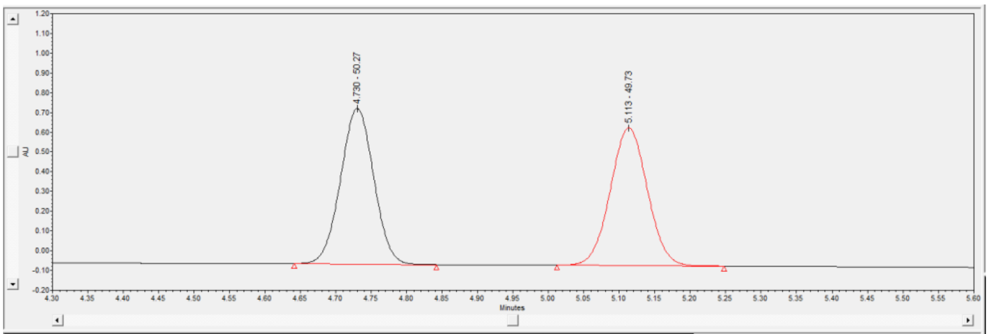

	Name	Retention Time (min)	Purity1 Angle	Purity1 Threshold	PDA/FLR Match1 Spect. Name	PDA/FLR Match1 Angle	PDA/FLR Match1 Threshold	PDA/FLR Match1 Lib. Name	Area (µV*sec)	% Area	Height (µV)	Int Type	Amount	Units	Peak Type	Peak Codes
1		4.730							2567707	50.27	787753	bb			Unknown	
2		5.113							2540524	49.73	697698	bb			Unknown	

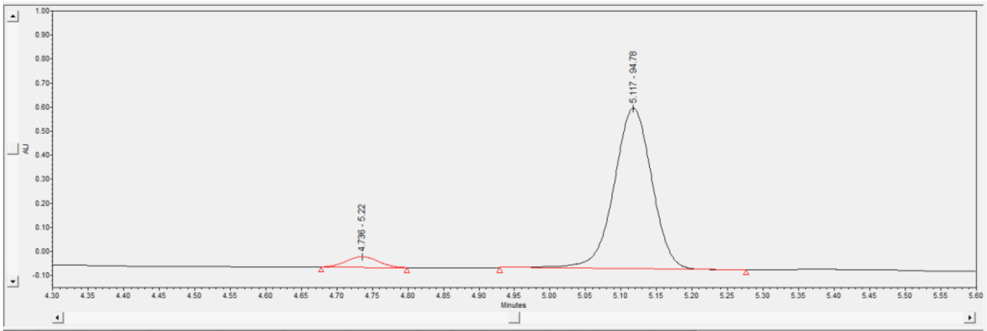

	Name	Retention Time (min)	Purity1 Angle	Purity1 Threshold	PDA/FLR Match1 Spect. Name	PDA/FLR Match1 Angle	PDA/FLR Match1 Threshold	PDA/FLR Match1 Lib. Name	Area (µV*sec)	% Area	Height (µV)	Int Type	Amount	Units	Peak Type	Peak Codes
1		4.736							136352	5.22	44125	bb			Unknown	
2		5.117							2475261	94.78	668180	bb			Unknown	

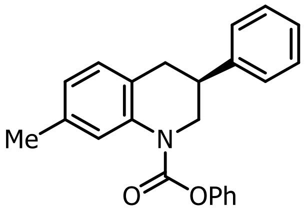

Phenyl-(S)-6-methyl-3-phenyl-3,4-dihydroquinoline-1(2H)-carboxylate (3at): The corresponding compound was prepared following general procedure **A** using phenyl boronic acid and phenyl 6-methylquinoline-1(2H)-carboxylate. The mixture was stirred at 30 °C for 48 hours. Purification by flash chromatography (5% acetone/petrol to 25% acetone/petrol) afforded compound **3at** as viscous liquid (87% yield, 85% ee).

^{1}H NMR ($CDCl_3$, 500 MHz) (2 rotamers): δ (ppm) 7.71 (s, 1H), 7.39 – 7.32 (m, 4H), 7.32 – 7.27 (m, 2H), 7.23 – 7.18 (m, 1H), 7.09 (d, *J* = 7.8 Hz, 1H), 7.03 (d, *J* = 7.8 Hz, 2H), 6.93 (dd, *J* = 7.8, 1.7 Hz, 1H), 4.32 (ddd, *J* = 12.8, 4.1, 1.2 Hz, 1H), 3.84 (t, *J* = 11.2 Hz, 1H), 3.31 (tdd, *J* = 9.6, 5.8, 4.1 Hz, 1H), 3.18 (dd, *J* = 16.4, 5.8 Hz, 1H), 3.07 (dd, *J* = 16.4, 9.2 Hz, 1H), 2.35 (s, 3H). **^{13}C NMR** (126 MHz, $CDCl_3$) (2 rotamers): δ (ppm) 153.34, 151.21, 142.73, 137.63, 136.16, 129.39, 128.91, 128.80, 127.41, 127.16, 126.34, 125.61, 125.39, 124.42, 121.88, 51.11, 40.34, 34.27, 21.47.

HRMS (ESI): m/z calcd for $C_{23}H_{22}O_2N^+$ $[M + H]^+$ 344.1645 found 344.1641.

IR (ν_{max}/cm^{-1}) 1723, 1508, 1494, 1455, 1385, 1335, 1269, 1199, 1164, 1119, 752, 701, 645.

SFC Conditions: Chiralpak IC; Gradient 1; 92.5:7.5 er (major enantiomer t_R = 4.94 min; minor enantiomer t_R = 4.63 min), **85% ee**. **$[\alpha]^{25}_D$ =** +8.2 (c = 2.0, $CHCl_3$).

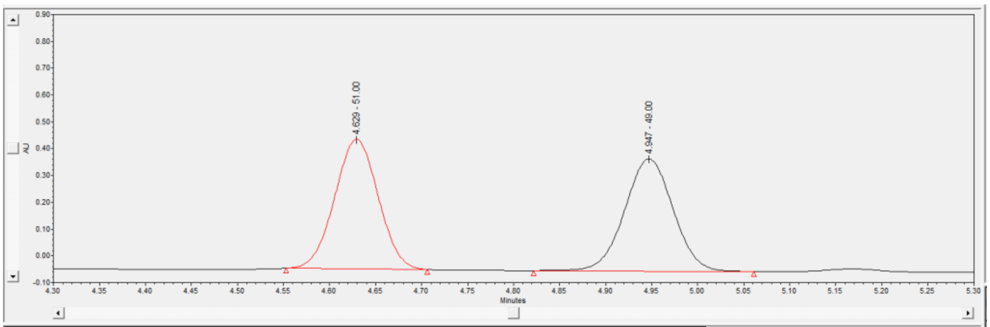

	Name	Retention Time (min)	Purity1 Angle	Purity1 Threshold	PDA/FLR Match1 Spect. Name	PDA/FLR Match1 Angle	PDA/FLR Match1 Threshold	PDA/FLR Match1 Lib. Name	Area (µV*sec)	% Area	Height (µV)	Int Type	Amount	Units	Peak Type	Peak Codes
1		4.629							1590892	51.00	483180	bb			Unknown	
2		4.947							1528713	49.00	418044	bb			Unknown	

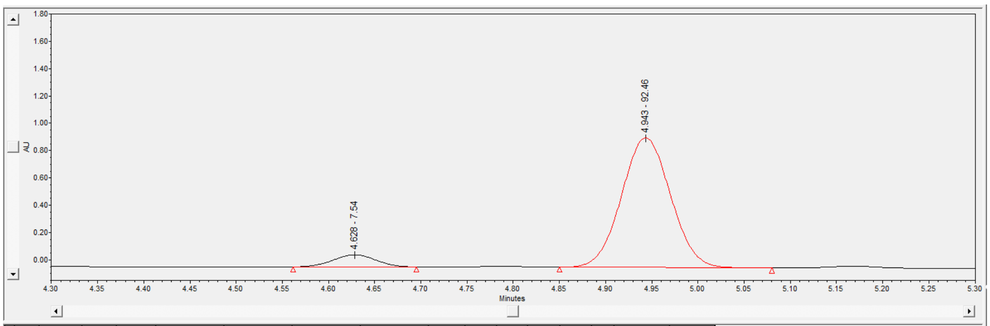

	Name	Retention Time (min)	Purity1 Angle	Purity1 Threshold	PDA/FLR Match1 Spect. Name	PDA/FLR Match1 Angle	PDA/FLR Match1 Threshold	PDA/FLR Match1 Lib. Name	Area (µV*sec)	% Area	Height (µV)	Int Type	Amount	Units	Peak Type	Peak Codes
1		4.628							286237	7.54	88622	bb			Unknown	
2		4.943							3511233	92.46	949410	bb			Unknown	

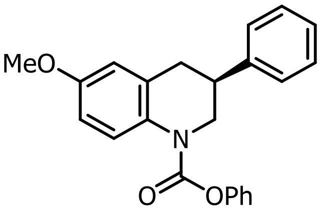

Phenyl-(S)-6-methoxy-3-phenyl-3,4-dihydroquinoline-1(2H)-carboxylate (3au): The corresponding compound was prepared following general procedure **A** using phenyl boronic acid and phenyl 6-methoxyquinoline-1(2H)-carboxylate. The mixture was stirred at 30 °C for 48 hours. Purification by flash chromatography (5% acetone/petrol to 25% acetone/petrol) afforded compound **3au** as viscous liquid (82% yield, 85% ee).

^{1}H NMR ($CDCl_3$, 500 MHz) (2 rotamers): δ (ppm) 7.76 (s, 1H), 7.39 – 7.27 (m, 7H), 7.19 (t, *J* = 7.4 Hz, 1H), 7.07 – 7.00 (m, 2H), 6.80 (dd, *J* = 9.0, 2.8 Hz, 1H), 6.73 (d, *J* = 2.8 Hz, 1H), 4.31 (dd, *J* = 12.7, 4.0 Hz, 1H), 3.86– 3.67 (m, 4H), 3.32 (tt, *J* = 9.4, 5.1 Hz, 1H), 3.19 (dd, *J* = 16.6, 5.8 Hz, 1H), 3.09 (dd, *J* = 16.6, 9.4 Hz, 1H). **^{13}C NMR** (126 MHz, $CDCl_3$) (2 rotamers): δ (ppm) 156.3, 153.3, 151.2, 142.6, 131.1, 129.4, 128.9, 127.4, 127.2, 125.5, 125.2, 121.8, 115.4, 113.6, 112.2, 55.6, 51.0, 40.4, 34.9.

HRMS (ESI): m/z calcd for $C_{23}H_{21}O_3N^+$ $[M + H]^+$ 360.1594 found 360.1590.

IR (ν_{max}/cm^{-1}) 1719, 1503, 1386, 1318, 1272, 1194, 1164, 1118, 749, 701, 690, 645.

SFC Conditions: Chiralpak IB; Gradient 1; 92.5:7.5 er (major enantiomer t_R = 4.77 min; minor enantiomer t_R = 4.58 min), **85% ee. $[\alpha]^{25}_D$ =** +9.0 (c = 2.0, $CHCl_3$).

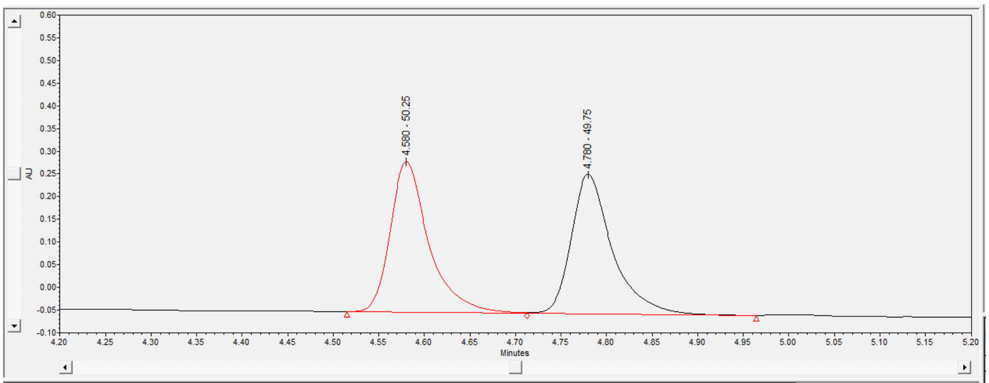

	Name	Retention Time (min)	Purity1 Angle	Purity1 Threshold	PDA/FLR Match1 Spect. Name	PDA/FLR Match1 Angle	PDA/FLR Match1 Threshold	PDA/FLR Match1 Lib. Name	Area (µV*sec)	% Area	Height (µV)	Int Type	Amount	Units	Peak Type	Peak Codes
1		4.580							970937	50.25	331860	bv			Unknown	
2		4.780							961113	49.75	307220	vb			Unknown	

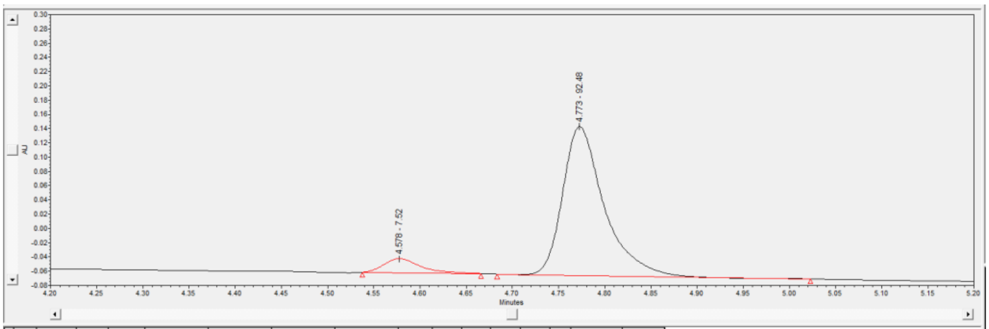

	Name	Retention Time (min)	Purity1 Angle	Purity1 Threshold	PDA/FLR Match1 Spect. Name	PDA/FLR Match1 Angle	PDA/FLR Match1 Threshold	PDA/FLR Match1 Lib. Name	Area (µV*sec)	% Area	Height (µV)	Int Type	Amount	Units	Peak Type	Peak Codes
1		4.578							53277	7.52	19606	bb			Unknown	
2		4.773							655151	92.48	209031	bb			Unknown	

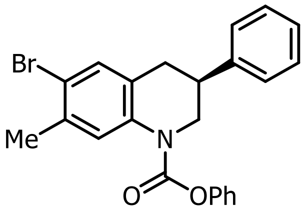

Phenyl-(S)-6-bromo-7-methyl-3-phenyl-3,4-dihydroquinoline-1(2H)-carboxylate (3av): The corresponding compound was prepared following general procedure **A** using phenyl boronic acid and phenyl 6-bromo-7-methylquinoline-1(2H)-carboxylate. The mixture was stirred at 70 °C for 20 hours. Purification by flash chromatography (2% acetone/petrol to 25% acetone/petrol) afforded compound **3av** as viscous liquid (80% yield, 86% ee).

^{1}H NMR ($CDCl_3$, 500 MHz) (2 rotamers): δ (ppm) 7.84 (s, 1H), 7.41 – 7.27 (m, 8H), 7.25 – 7.20 (m, 1H), 7.02 (d, J = 8.0 Hz, 2H), 4.30 (ddd, J = 12.8, 4.0, 1.2 Hz, 1H), 3.88 (t, J = 11.1 Hz, 1H), 3.31 (tdd, J = 9.4, 5.8, 3.9 Hz, 1H), 3.17 (dd, J = 16.5, 5.8 Hz, 1H), 3.07 (dd, J = 16.5, 9.0 Hz, 1H), 2.40 (s, 3H). **^{13}C NMR** (126 MHz, $CDCl_3$) (2 rotamers): δ (ppm) 153.1, 151.0, 142.1, 136.9, 135.8, 132.1, 129.4, 129.0, 128.6, 127.32, 127.3, 125.9, 125.7, 121.8, 119.9, 51.0, 39.8, 33.8, 22.9.

HRMS (ESI): m/z calcd for $C_{23}H_{21}O_2NBr^+$ $[M + H]^+$ 422.0750 found 422.0740.

IR (ν_{max}/cm^{-1}) 1724, 1492, 1383, 1203, 1163, 757, 700.

SFC Conditions: Chiralpak ID; Gradient 1; 93:7 er (major enantiomer t_R = 4.59 min; minor enantiomer t_R = 4.98 min), **86% ee. [α]$^{25}_{D}$ =** +45.8 (c = 2.0, $CHCl_3$).

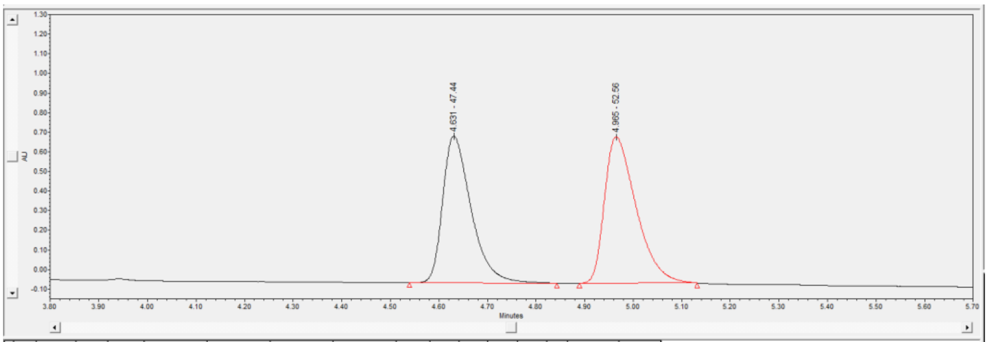

	Name	Retention Time (min)	Purity1 Angle	Purity1 Threshold	PDA/FLR Match1 Spect. Name	PDA/FLR Match1 Angle	PDA/FLR Match1 Threshold	PDA/FLR Match1 Lib. Name	Area (µV*sec)	% Area	Height (µV)	Int Type	Amount	Units	Peak Type	Peak Codes
1		4.631							3052541	47.44	748952	bb			Unknown	
2		4.965							3382177	52.56	746892	bb			Unknown	

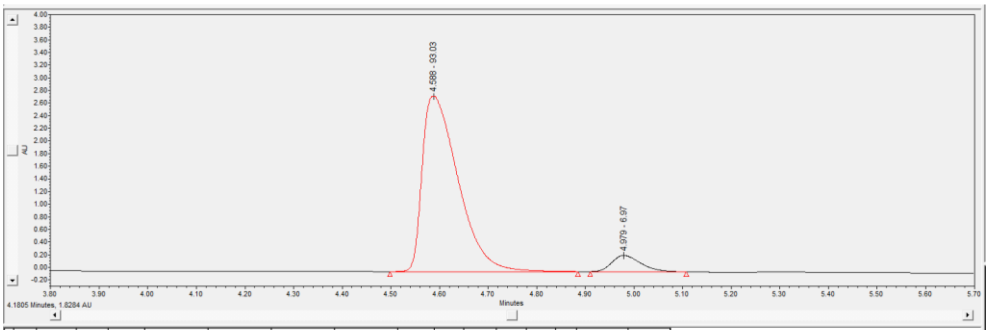

	Name	Retention Time (min)	Purity1 Angle	Purity1 Threshold	PDA/FLR Match1 Spect. Name	PDA/FLR Match1 Angle	PDA/FLR Match1 Threshold	PDA/FLR Match1 Lib. Name	Area (µV*sec)	% Area	Height (µV)	Int Type	Amount	Units	Peak Type	Peak Codes
1		4.588							14216454	93.03	2775991	bb			Unknown	
2		4.979							1064329	6.97	258374	bb			Unknown	

4. Derivatization of Chiral Tetrahydropyridines

Synthesis of trisubstituted piperidine (4):

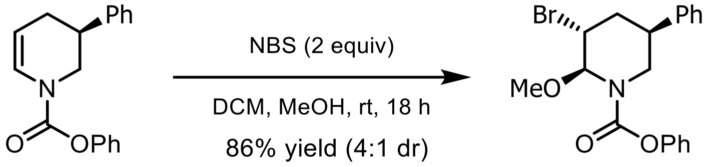

A solution of N-boromosuccinimide (75 mg, 0.42 mmol) in methanol (1 mL) was added to a solution of dihydropyridine **3a** (58 mg, 0.21 mmol) in DCM (1 mL) at 0 °C then the mixture was allowed to warm to room temperature. After 16 h, the mixture was poured into a saturated aqueous solution of NaHCO3. The aqueous phase was extracted three times with DCM and the solvent was removed in vacuo. The residue was purified by flash chromatography on silica gel (5% acetone/petrol to 20% acetone/petrol) affording **4** (86% yield, 4:1 dr) as yellow oil.

^{1}H NMR ($CDCl_3$, 500 MHz) (2 rotamers * 2 diastereomers): δ (ppm) 7.44 – 7.10 (m, 10H), 5.75 – 5.58 (m, 1H), 4.56 – 4.12 (m, 2H), 3.61 – 3.37 (m, 4H), 3.32 – 3.07 (m, 1H), 2.71 – 2.54 (m, 1H), 2.46 – 2.11 (m, 1H). **^{13}C NMR** (126 MHz, $CDCl_3$) (2 rotamers, peak picked major diastereomer): δ (ppm) 154.8, 154.4, 151.3, 151.2, 141.6, 141.5, 129.5, 129.4, 128.9, 128.8, 127.5, 127.34, 127.26, 125.8, 125.6, 121.9, 121.7, 85.4, 84.7, 55.8, 55.4, 49.3, 48.7, 45.1, 44.3, 36.7, 36.3, 34.0, 33.9. **$[\alpha]^{25}_D$ =** +42.5 (c = 2.0, $CHCl_3$).

HRMS (ESI): m/z calcd for $C_{19}H_{21}O_3NBr^+$ $[M + H]^+$ 390.0699 found 390.0721.

IR (ν_{max}/cm^{-1}) 2935, 1726, 1596, 1495, 1455, 1415, 1351, 1267, 1206, 1163, 1069, 941, 876, 833, 756, 729, 701, 689.

Access towards (-) Preclamol:

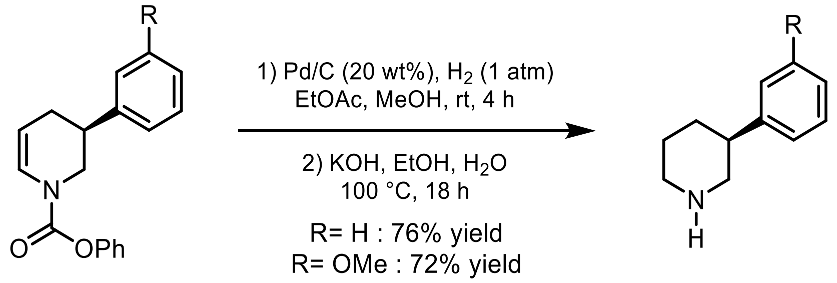

A solution of dihydropyridine (0.5 mmol) in MeOH (1 mL) was added to a vial containing Pd/C (20 wt.%) in EtOAc (1 mL). The reaction mixture was purged with hydrogen (1 atm) balloon and stirred at room temperature. After 15 min of purging, the mixture was stirred under hydrogen atmosphere for 4 h. Upon completion of reaction, the mixture was diluted with Et_2O (5 mL) before passing through a plug of celite. The plug was washed will additional 10 mL of Et_2O and the solvents were removed in vacuo. Purification by flash chromatography (2% acetone/petrol to 20% acetone/petrol) afforded protected piperidine as viscous liquid.

A sealed flask protected piperidine (obtained above) and KOH (5 mmol, 10 equiv) in EtOH (3 mL) and H_2O (1 mL) was heated at 100 °C. After 18h, the mixture was warmed to room

temperature and diluted with H_2O (10 mL). The aqueous layer was extracted with DCM (2 × 10 mL). The combined organic layer was dried over anhydrous Na_2SO_4, filtered, and the solvent was removed in vacuo to afford the corresponding amine.

The hydrochloride salts were isolated by adding 4N HCL (in dioxane) to the solution of corresponding amine in DCM (2 ml). After 15 minutes, the mixture was diluted with Et_2O (15 ml), precipitated solid was filtered then dried under vacuum to provide the corresponding product as off white solid.

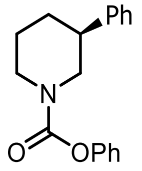

Phenyl (S)-3-phenyl piperidine-1-carboxylate (S13): The corresponding compound was prepared following the procedure described above using dihydropyridine (**3a**) as viscous liquid. **^{1}H NMR** ($CDCl_3$, 500 MHz) (2 rotamers): δ (ppm) 7.42 – 7.06 (m, 10H), 4.48 – 4.25 (m, 2H), 3.12 – 2.75 (m, 3H), 2.17 – 2.07 (m, 1H), 1.94 – 1.83 (m, 1H), 1.81 – 1.58 (m, 2H). **^{13}C NMR** (126 MHz, $CDCl_3$) (2 rotamers): δ (ppm) 153.8, 151.6, 143.1, 129.3, 128.7, 127.2, 126.9, 125.3, 121.9, 51.4, 51.0, 45.2, 44.7, 43.0, 42.5, 31.7, 25.9, 25.4. **$[\alpha]^{25}_D$ =** −78.3 (c = 2.0, $CHCl_3$).

IR (ν_{max}/cm^{-1}) 2936, 1719, 1595, 1495, 1425, 1256, 1234, 1199, 1163, 1127, 1072, 1026, 981, 907, 831, 749, 701, 689, 670.

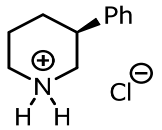

(S)-3-Phenylpiperidine hydrochloride salt (5): The corresponding compound was prepared following the procedure described as yellow solid (76% yield over 2 steps). **^{1}H NMR** (500 MHz, $CDCl_3$): δ (ppm) 9.73– 7.17 (m, 2H), 7.35 – 7.17 (m, 5H), 3.67 – 3.45 (m, 2H), 3.27 (t, *J* = 12.2 Hz, 1H), 2.92 (dq, *J* = 22.4, 11.4, 10.9 Hz, 2H), 2.14 (dd, *J* = 33.3, 14.1 Hz, 2H), 2.00 (d, *J* = 14.1 Hz, 1H), 1.73 – 1.61 (m, 1H). **^{13}C NMR** (126 MHz, $CDCl_3$) δ 140.9, 129.0, 127.6, 127.0, 49.4, 44.1, 39.6, 30.4, 22.7. **$[\alpha]^{25}_D$ =** +6.7 (c = 0.3, CH_3OH). The spectroscopic data satisfactorily matched previously reported data.[4]

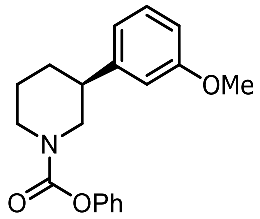

Phenyl (S)-3-(3-methoxyphenyl) piperidine-1-carboxylate (S14): The corresponding compound was prepared following the procedure described above using dihydropyridine (**3r**) as viscous liquid. **^{1}H NMR** ($CDCl_3$, 500 MHz) (2 rotamers): δ (ppm) 7.40 – 7.34 (m, 2H), 7.30 – 7.10 (m, 4H), 6.92 – 6.77 (m, 3H), 4.50 – 4.26 (m, 2H), 3.82 (s, 3H), 3.09 – 2.75 (m, 3H), 2.16 – 2.06 (m, 1H), 1.93 – 1.83 (m, 1H), 1.78 – 1.66 (m, 2H). **^{13}C NMR** (126 MHz, $CDCl_3$)

(2 rotamers): δ (ppm) ^{13}C NMR (126 MHz, $CDCl_3$) δ 159.9, 153.9, 151.6, 144.8, 129.7, 129.3, 125.3, 121.9, 119.5, 113.4, 113.2, 112.0, 111.8, 55.3, 51.3, 50.9, 45.2, 44.7, 43.0, 42.7, 31.7, 31.6, 25.8, 25.3. **$[\alpha]^{25}_D$ =** −59.8 (c = 2.0, $CHCl_3$).

HRMS (ESI): m/z calcd for $C_{19}H_{22}O_3N^+$ $[M + H]^+$ 312.1594 found 312.1605.

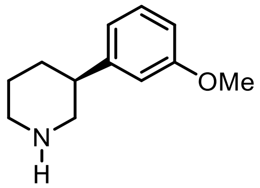

(S)-3-(3-methoxyphenyl) piperidine (6): The corresponding compound was prepared following the procedure described above as yellow oil (72% yield over 2 steps).

(6): ^{1}H NMR (400 MHz, $CDCl_3$): δ (ppm) 10.02 – 9.36 (m, 2H), 7.25 – 7.15 (m, 1H), 6.83 – 6.67 (m, 3H), 3.76 (s, 3H), 3.49 (t, *J* = 10.2 Hz, 2H), 3.20 (tt, *J* = 12.5, 3.5 Hz, 1H), 2.89 (q, *J* = 16.7, 14.2 Hz, 2H), 2.23 – 1.89 (m, 3H), 1.62 (qd, *J* = 12.5, 3.2 Hz, 1H). **$[\alpha]^{25}_D$ =** +10.6 (c = 1.0, CH_3OH). The spectroscopic data satisfactorily matched previously reported data.[4–6]

(6.HCl): ^{1}H NMR (500 MHz, $CDCl_3$): δ (ppm) 9.98 – 9.40 (m, 2H), 7.25 – 7.15 (m, 1H), 6.83 – 6.67 (m, 3H), 3.76 (s, 3H), 3.49 (t, *J* = 10.2 Hz, 2H), 3.20 (tt, *J* = 12.6, 3.5 Hz, 1H), 2.89 (q, *J* = 16.7, 14.2 Hz, 2H), 2.23 – 1.89 (m, 3H), 1.62 (qd, *J* = 12.6, 3.2 Hz, 1H). **^{13}C NMR** (126 MHz, $CDCl_3$) δ 160.0, 142.5, 130.0, 119.2, 112.91, 112.85, 55.3, 49.2, 44.0, 39.7, 30.3, 22.7. **$[\alpha]^{25}_D$ =** +8.6 (c = 2.1, CH_3OH). The spectroscopic data satisfactorily matched previously reported data.[4–6]

HRMS (ESI): m/z calcd for $C_{12}H_{18}ON^+$ $[M + H]^+$ 192.1383 found 192.1396.

IR (ν_{max}/cm^{-1}) 2935, 2854, 2361, 1698, 1602, 1585, 1492, 1468, 1436, 1263, 1192, 1160, 1048, 856, 784, 754, 700.

Access towards Niraparib:

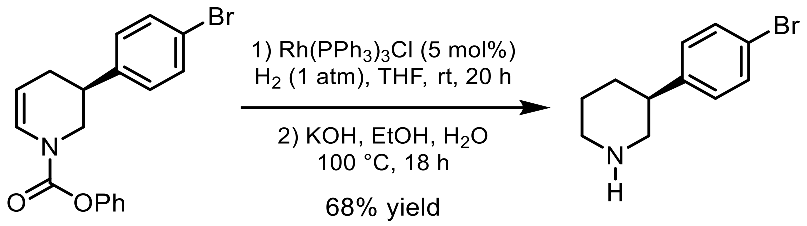

[$Rh(PPh_3)_3Cl$] (23 mg, 0.025 mmol, 5 mol%) was added to a 10 mL round bottom flask, sealed with a rubber septum, dissolved in THF (2 mL) and purged with hydrogen (1 atm) balloon while stirred at room temperature. After 10 min, a solution of **3k** (180 mg, 0.5 mmol, 1 equiv) in THF (2 mL) was added via syringe and again purged with hydrogen (1 atm) balloon while stirred at room temperature. After 15 min of purging, the mixture was stirred under hydrogen atmosphere for 18 h. Upon completion of reaction, the mixture was diluted with Et_2O (5 mL) before passing through a plug of silica. The plug was washed will additional 10 mL of Et_2O and the solvents were removed in vacuo. Purification by flash chromatography (5% acetone/petrol to 25% acetone/petrol) afforded protected piperidine as viscous liquid.

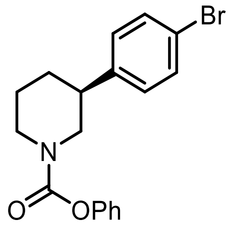

Phenyl (S)-3-(4-bromophenyl) piperidine-1-carboxylate (S15): **^{1}H NMR** ($CDCl_3$, 500 MHz) (2 rotamers): δ (ppm) 7.48 – 7.42 (m, 2H), 7.39 – 7.32 (m, 2H), 7.20 (t, *J* = 7.3 Hz, 1H), 7.17 – 7.06 (m, 4H), 4.43 – 4.27 (m, 2H), 3.09 – 2.71 (m, 3H), 2.11 – 2.03 (m, 1H), 1.91 – 1.82 (m, 1H), 1.77 – 1.64 (m, 2H). **^{13}C NMR** (126 MHz, $CDCl_3$) (2 rotamers): δ (ppm) 153.9, 151.5, 142.0, 131.8, 129.4, 129.0, 128.7, 127.8, 125.4, 121.9, 51.1, 50.7, 45.1, 44.6, 42.4, 42.0, 31.6, 25.8, 25.2. **$[\alpha]^{25}_D$ =** −84.3 (c = 2.0, $CHCl_3$).

HRMS (ESI): m/z calcd for $C_{18}H_{19}O_2BrN^+$ $[M + H]^+$ 360.0594 found 360.0611.

IR (ν_{max}/cm^{-1}) 2940, 2860, 2361, 1722, 1594, 1492, 1465, 1426, 1255, 1235, 1208, 1164, 1129, 1075, 1010, 982, 818, 754, 717, 691.

A sealed flask containing protected piperidine **S15** (obtained above) and KOH (5 mmol, 10 equiv) in EtOH (3 mL) and H_2O (1 mL) was heated at 100 °C. After 18h, the mixture was warmed to room temperature and diluted with H_2O (10 mL). The aqueous layer was extracted with DCM (2 × 10 mL). The combined organic layer was dried over anhydrous Na_2SO_4, filtered, and the solvent was removed in vacuo to afford the corresponding amine (**7**) as yellow oil (82 mg, 68% yield over 2 steps).

The hydrochloride salts were isolated by adding 4N HCL (in dioxane) to the solution of corresponding amine in DCM (2 ml). After 15 minutes, the mixture was diluted with Et_2O (15 ml), precipitated solid was filtered then dried under vacuum to provide the corresponding product as off white solid (94 mg, 68% yield over 2 steps).

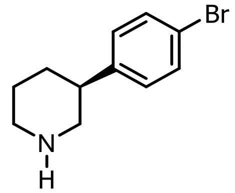

(7): ^{1}H NMR (500 MHz, $CDCl_3$): δ (ppm) 7.36 – 7.29 (m, 2H), 7.06 – 6.97 (m, 2H), 3.04 (t, *J* = 11.8 Hz, 2H), 2.59 – 2.48 (m, 3H), 1.93 – 1.82 (m, 1H), 1.71 (dt, *J* = 9.1, 2.5 Hz, 1H), 1.65 – 1.41 (m, 3H). **$[\alpha]^{25}_{D}$ =** +8.7 (c = 1.0, CH_3OH). The spectroscopic data satisfactorily matched previously reported data.[7,8]

(7.HCl): ^{1}H NMR (500 MHz, $CDCl_3$): δ (ppm) 9.77 (d, *J* = 99.8 Hz, 1H), 7.44 (d, *J* = 8.3 Hz, 2H), 7.06 (d, *J* = 8.3 Hz, 2H), 3.50 (q, *J* = 13.8, 13.3 Hz, 2H), 3.23 (t, *J* = 12.3 Hz, 1H), 2.86 (q, *J* = 11.8 Hz, 2H), 2.25 – 1.93 (m, 4H), 1.67 – 1.55 (m, 1H). **^{13}C NMR** (126 MHz, $CDCl_3$) δ 139.76, 132.18, 128.74, 121.52, 49.14, 43.97, 39.13, 30.25, 22.65. **$[\alpha]^{25}_{D}$ =** +4.3 (c = 1.0, CH_3OH).

HRMS (ESI): m/z calcd for $C_{11}H_{15}BrN^+$ $[M + H]^+$ 240.0382 found 240.0392.

IR (ν_{max}/cm^{-1}) 2933, 2856, 2361, 1697, 1490, 1468, 1438, 1257, 1237, 1202, 1137, 1076, 1010, 982, 818, 769, 818, 754, 717, 691.

5. References

(1) Kohari, Y.; Okuyama, Y.; Kwon, E.; Furuyama, T.; Kobayashi, N.; Otuki, T.; Kumagai, J.; Seki, C.; Uwai, K.; Dai, G.; Iwasa, T.; Nakano, H. Enantioselective Diels-Alder Reaction of 1,2-Dihydropyridines with Aldehydes Using β-Amino Alcohol Organocatalyst. *J. Org. Chem.* **2014**, *79* (20), 9500–9511.

(2) Krow, G. R.; Huang, Q.; Szczepanski, S. W.; Hausheer, F. H.; Carroll, P. J. Stereoselectivity in Diels-Alder Reactions of Diene-Substituted N-Alkoxycarbonyl-1,2-Dihydropyridines. *J. Org. Chem.* **2007**, *72* (9), 3458–3466.

(3) Heusler, A.; Fliege, J.; Wagener, T.; Glorius, F. Substituted Dihydropyridine Synthesis by Dearomatization of Pyridines. *Angew. Chemie Int. Ed.* **2021**, *60* (25), 13793–13797.

(4) Colpaert, F.; Mangelinckx, S.; De Kimpe, N. Asymmetric Synthesis of Chiral N -Sulfinyl 3-Alkyl- and 3-Arylpiperidines by α-Alkylation of N -Sulfinyl Imidates with 1-Chloro-3-Iodopropane. *J. Org. Chem.* **2011**, *76* (1), 234–244.

(5) Amat, M.; Cantó, M.; Llor, N.; Escolano, C.; Molins, E.; Espinosa, E.; Bosch, J. Dynamic Kinetic Resolution of Racemic γ-Aryl-δ-Oxoesters. Enantioselective Synthesis of 3-Arylpiperidines. *J. Org. Chem.* **2002**, *67* (15), 5343–5351.

(6) Zhang, S. J.; Sun, W. W.; Yu, Q. Y.; Cao, P.; Dong, X. P.; Wu, B. Stereoselective Synthesis of (−)-3-PPP through Palladium-Catalysed Unactivated C(Sp3)–H Arylation at the C-3 Position of l-Pipecolinic Acid. *Tetrahedron Lett.* **2017**, *58* (7), 606–609.

(7) Wallace, D. J.; Baxter, C. A.; Brands, K. J. M.; Bremeyer, N.; Brewer, S. E.; Desmond, R.; Emerson, K. M.; Foley, J.; Fernandez, P.; Hu, W.; Keen, S. P.; Mullens, P.; Muzzio, D.; Sajonz, P.; Tan, L.; Wilson, R. D.; Zhou, G.; Zhou, G. Development of a Fit-for-Purpose Large-Scale Synthesis of an Oral PARP Inhibitor. *Org. Process Res. Dev.* **2011**, *15* (4), 831–840.

(8) Chung, C. K.; Bulger, P. G.; Kosjek, B.; Belyk, K. M.; Rivera, N.; Scott, M. E.; Humphrey, G. R.; Limanto, J.; Bachert, D. C.; Emerson, K. M. Process Development of C-N Cross-Coupling and Enantioselective Biocatalytic Reactions for the Asymmetric Synthesis of Niraparib. *Org. Process Res. Dev.* **2014**, *18* (1), 215–227.

6. NMR Spectra

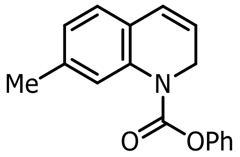

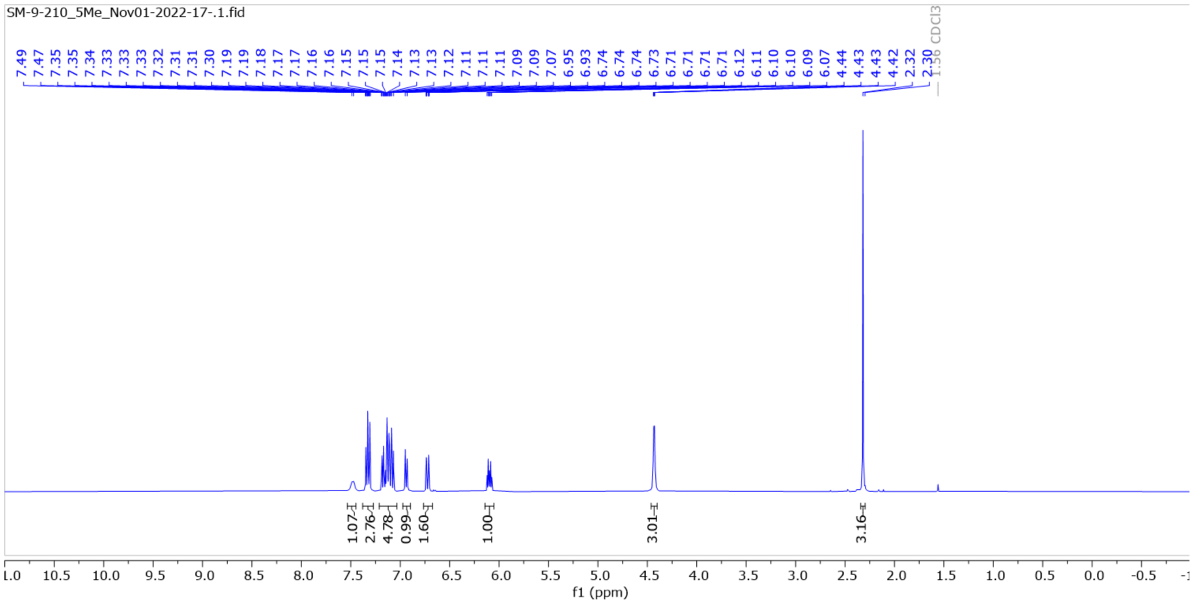

^{1}H NMR (400 MHz, $CDCl_3$) of **S10**

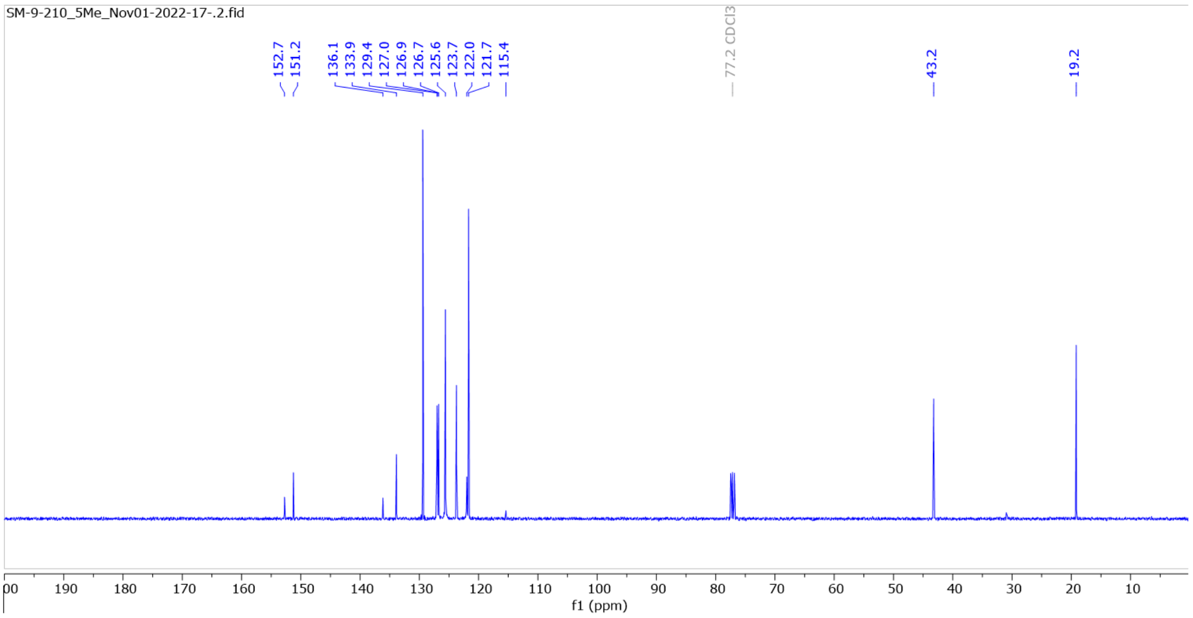

^{13}C NMR (101 MHZ, $CDCl_3$) of **S10**

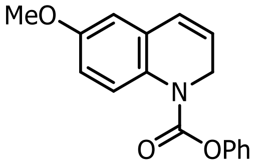

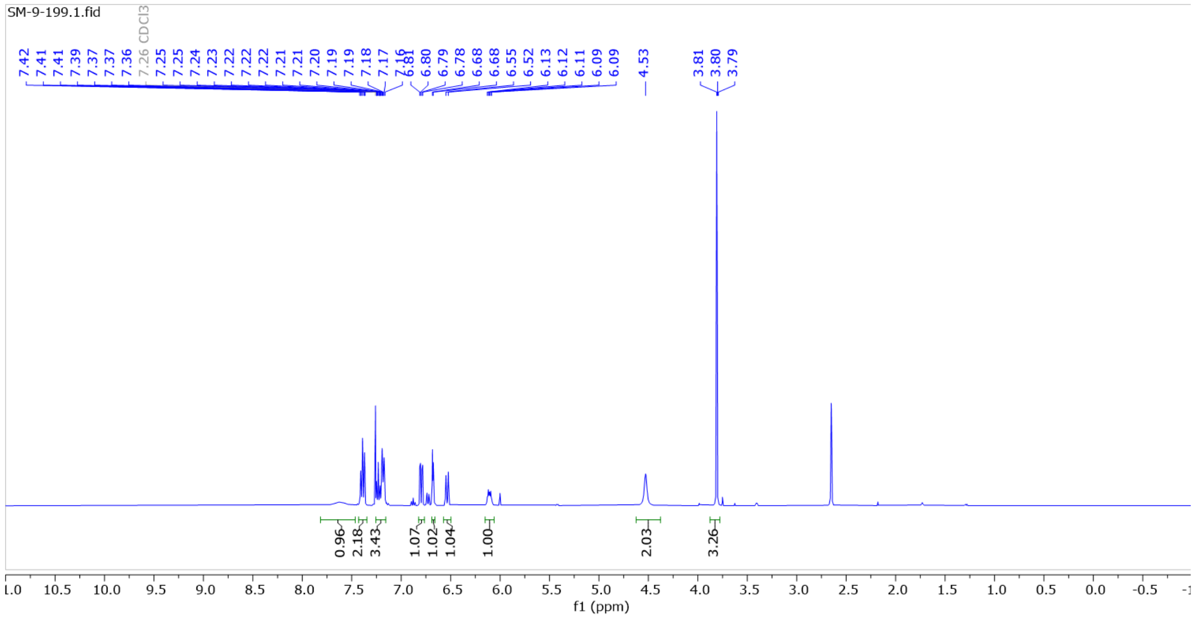

^{1}H NMR (400 MHz, $CDCl_3$) of **S11**

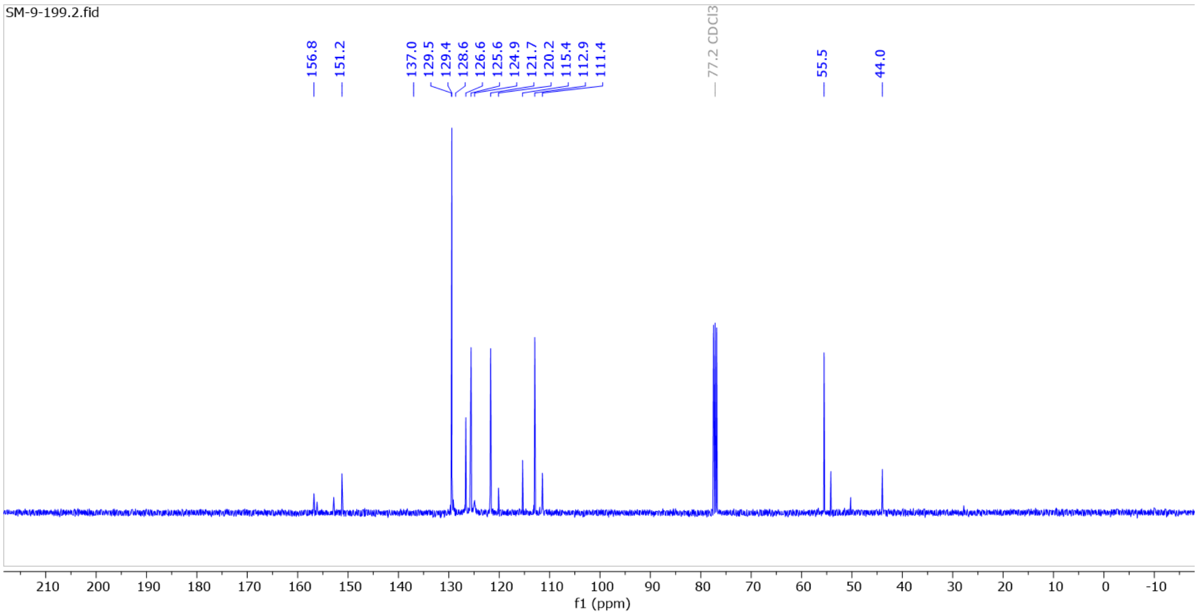

^{13}C NMR (101 MHZ, $CDCl_3$) of **S11**

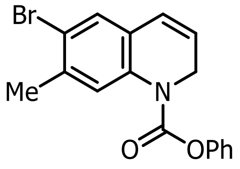

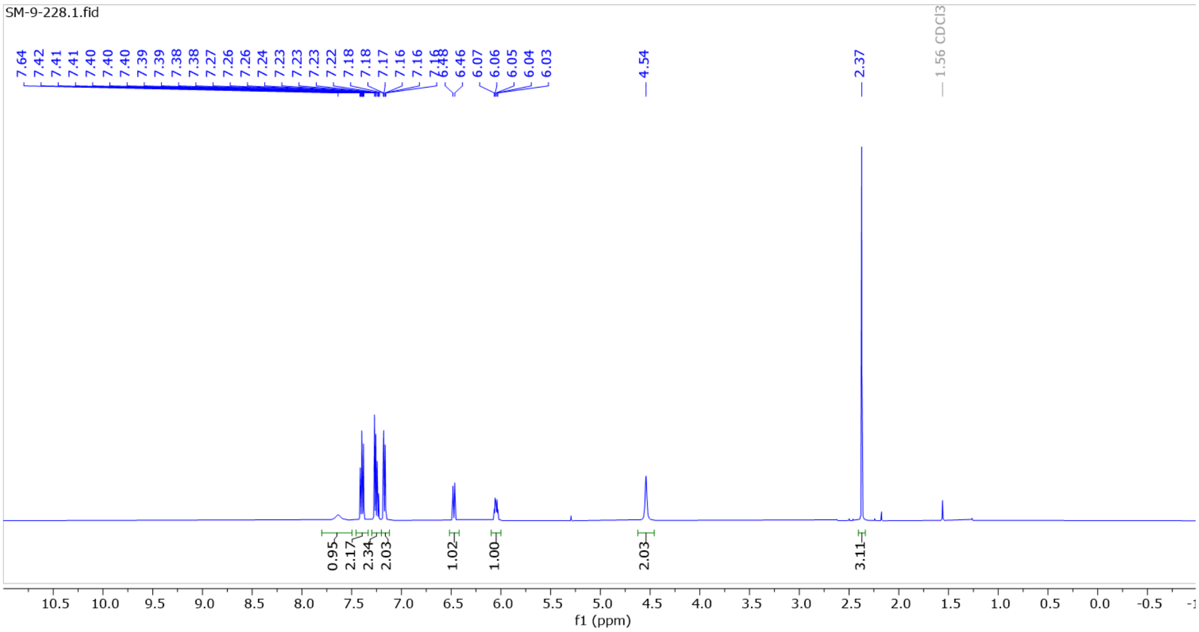

^{1}H NMR (400 MHz, $CDCl_3$) of **S12**

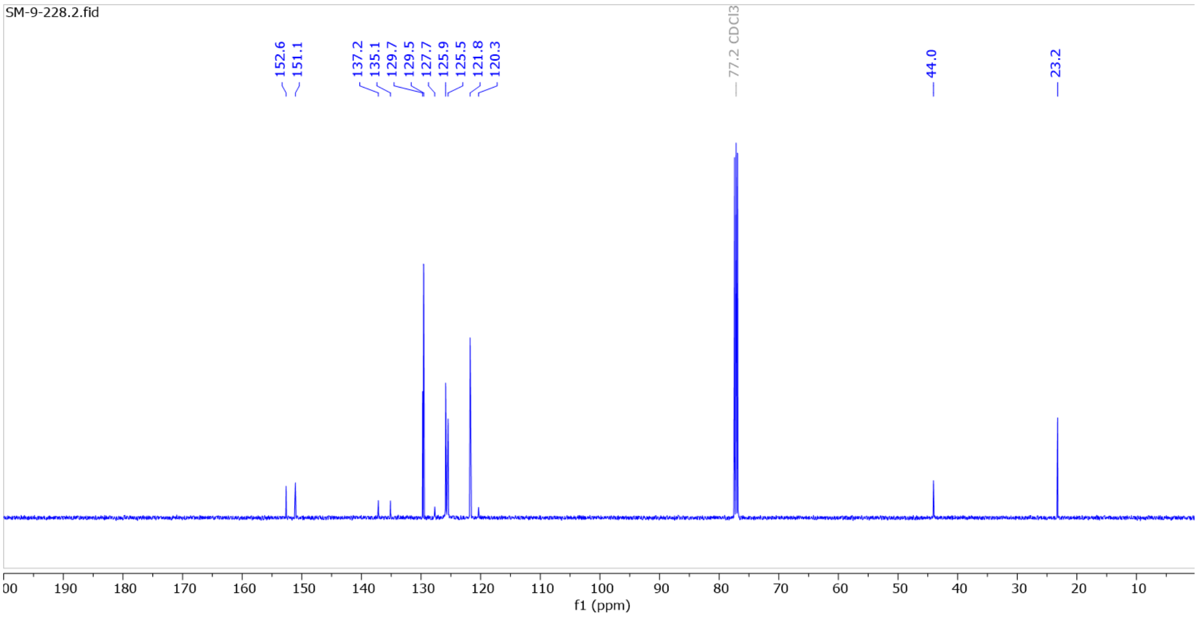

^{13}C NMR (101 MHZ, $CDCl_3$) of **S12**

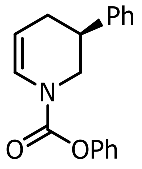

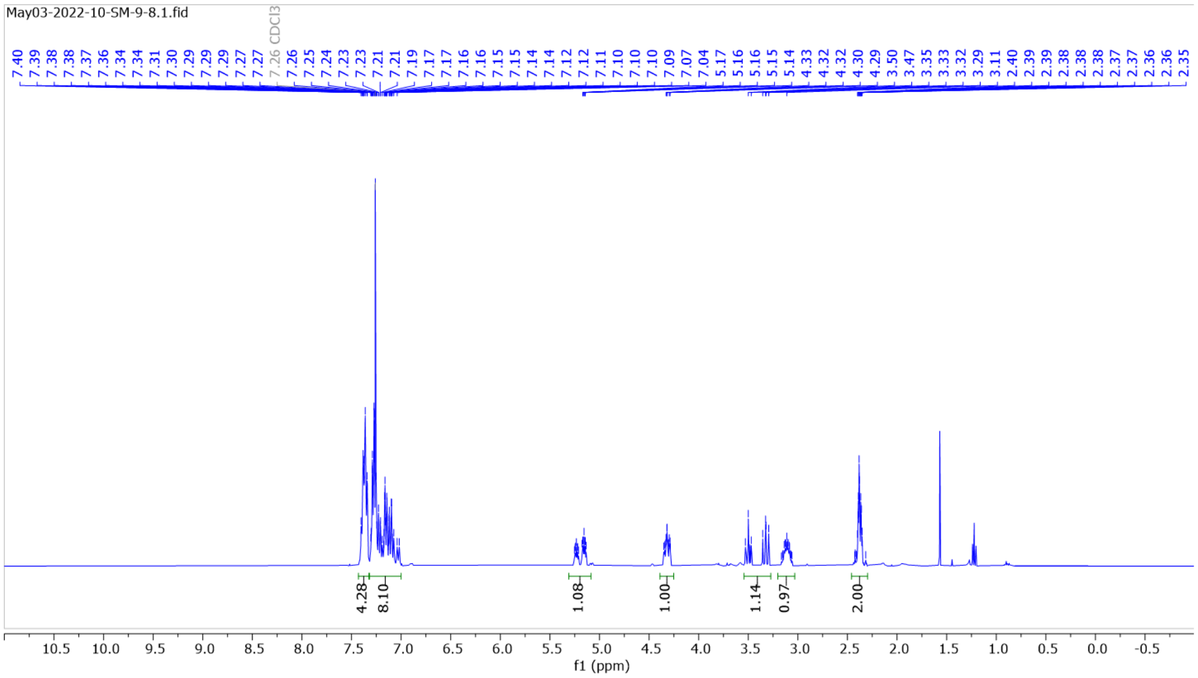

^{1}H NMR (400 MHz, $CDCl_3$) of **3a**

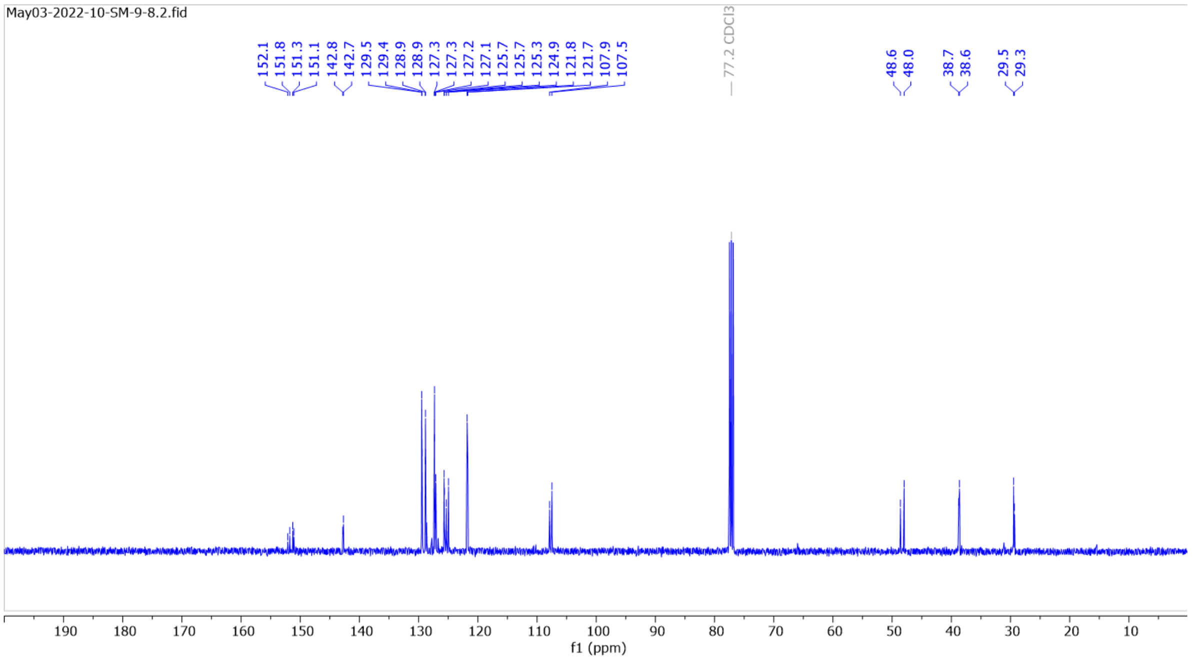

^{13}C NMR (101 MHZ, $CDCl_3$) of **3a**

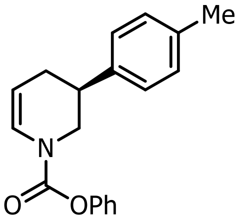

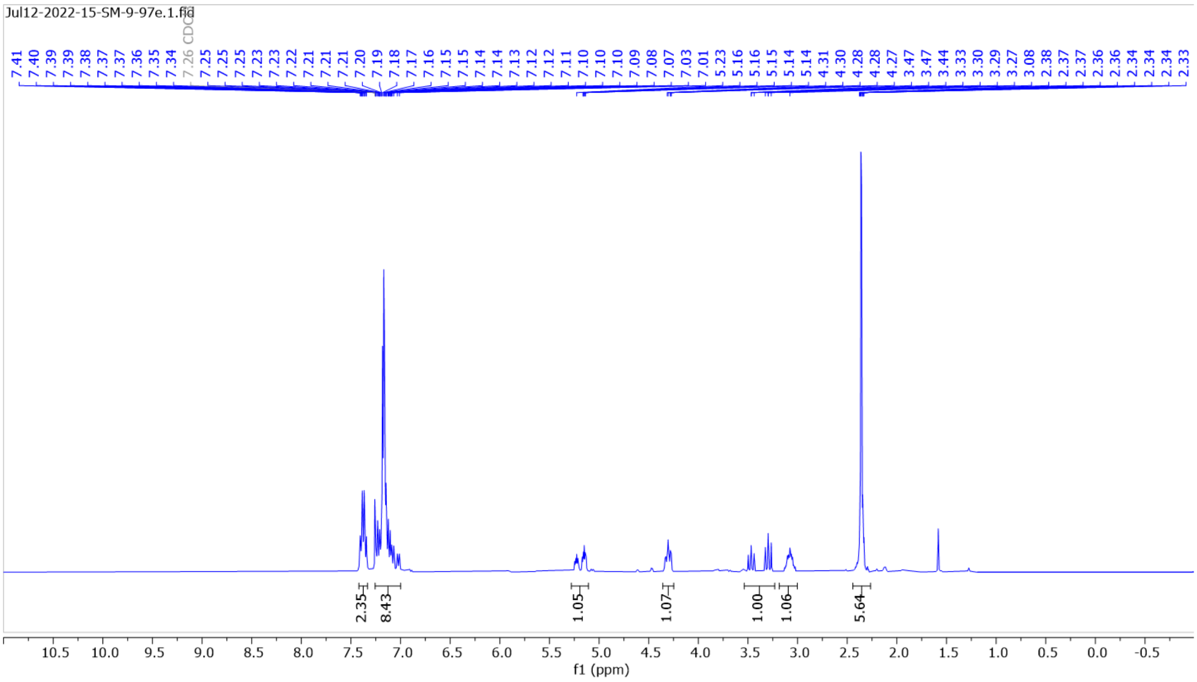

^{1}H NMR (400 MHZ, $CDCl_3$) of **3b**

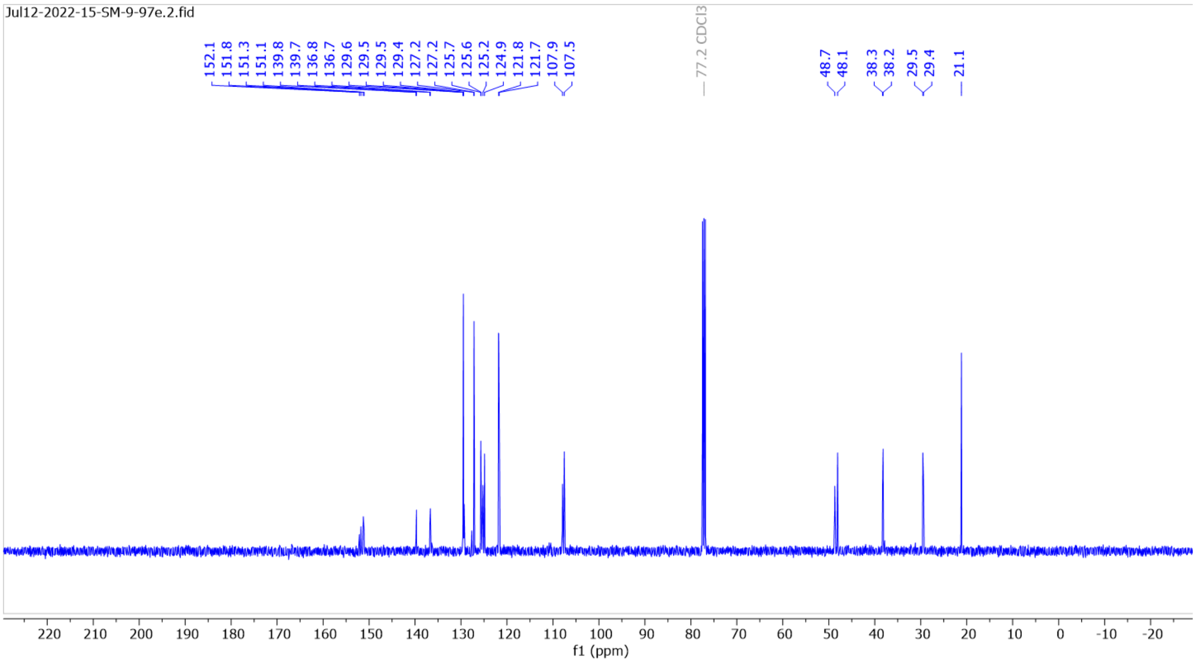

^{13}C NMR (101 MHZ, $CDCl_3$) of **3b**

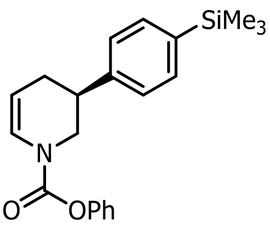

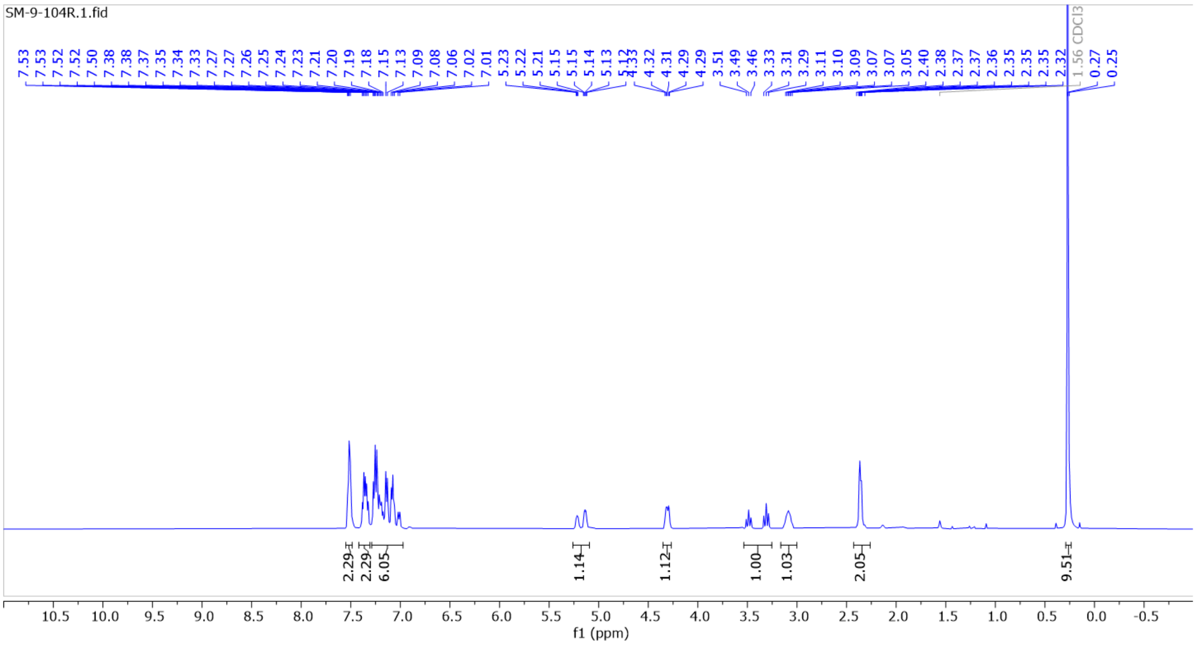

^{1}H NMR (500 MHZ, $CDCl_3$) of **3c**

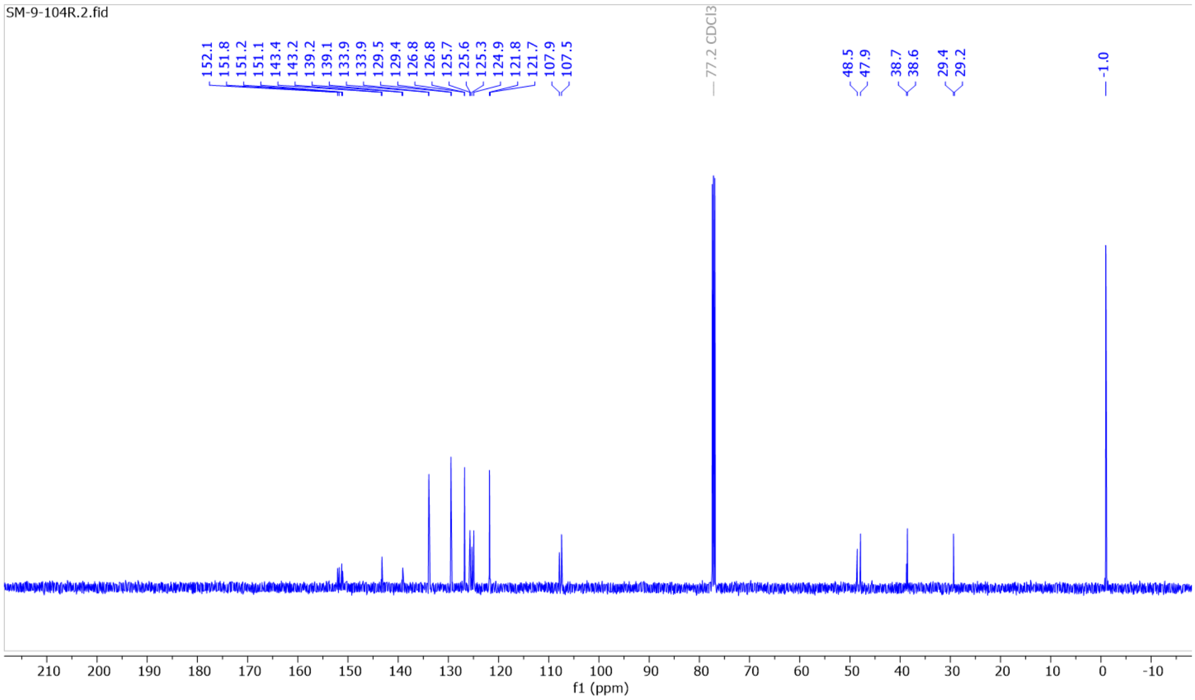

^{13}C NMR (126 MHZ, $CDCl_3$) of **3c**

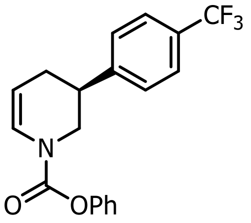

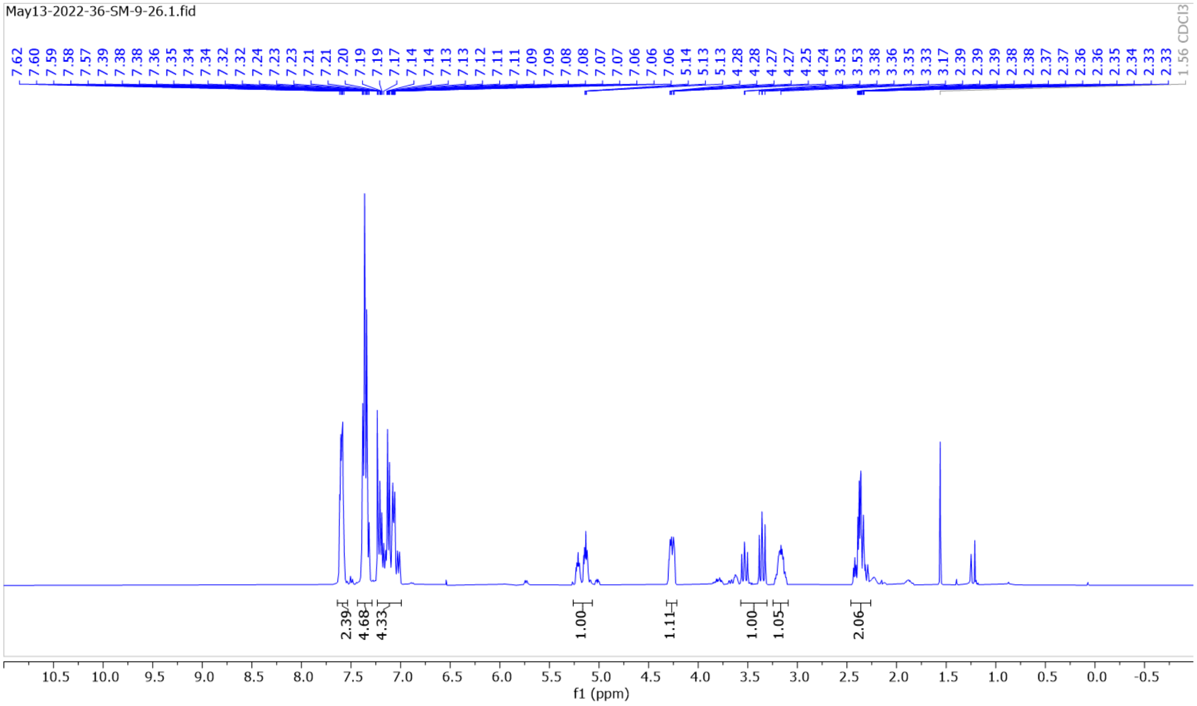

^{1}H NMR (400 MHZ, $CDCl_3$) of **3d**

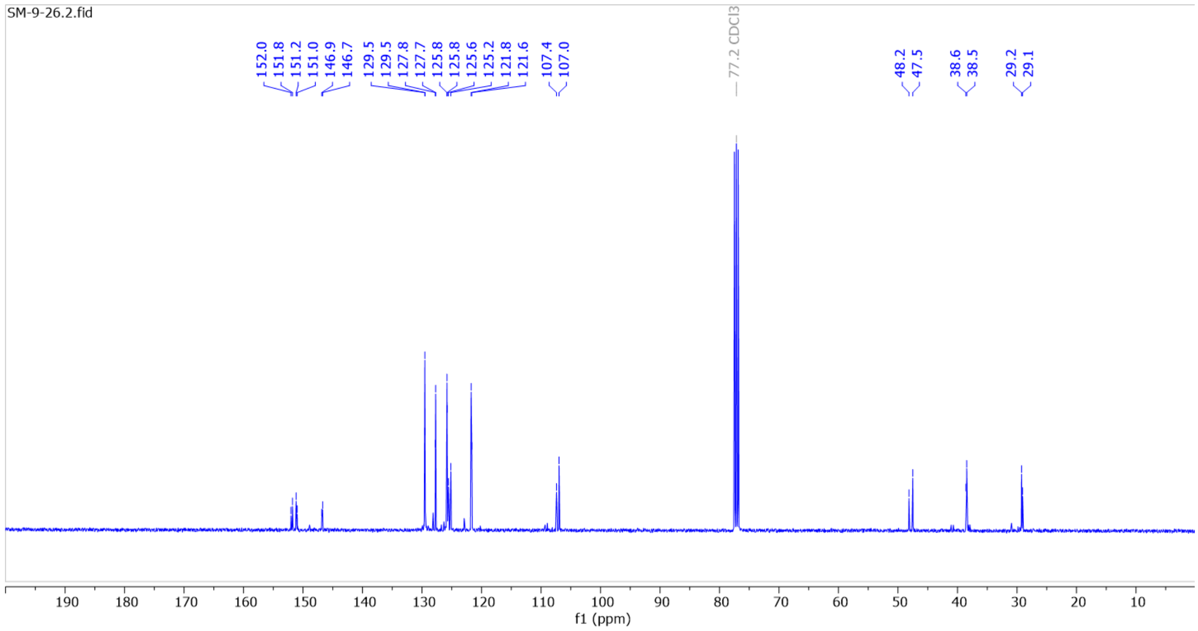

^{13}C NMR (101 MHZ, $CDCl_3$) of **3d**

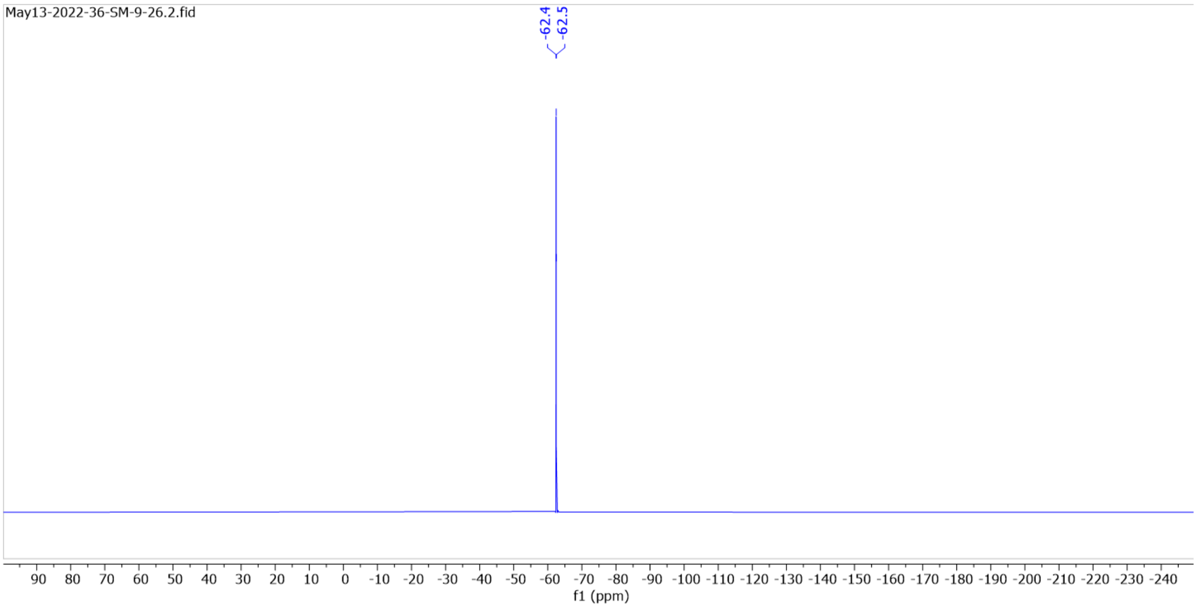

^{19}F NMR (376 MHZ, $CDCl_3$) of **3d**

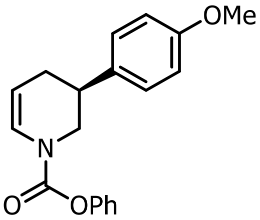

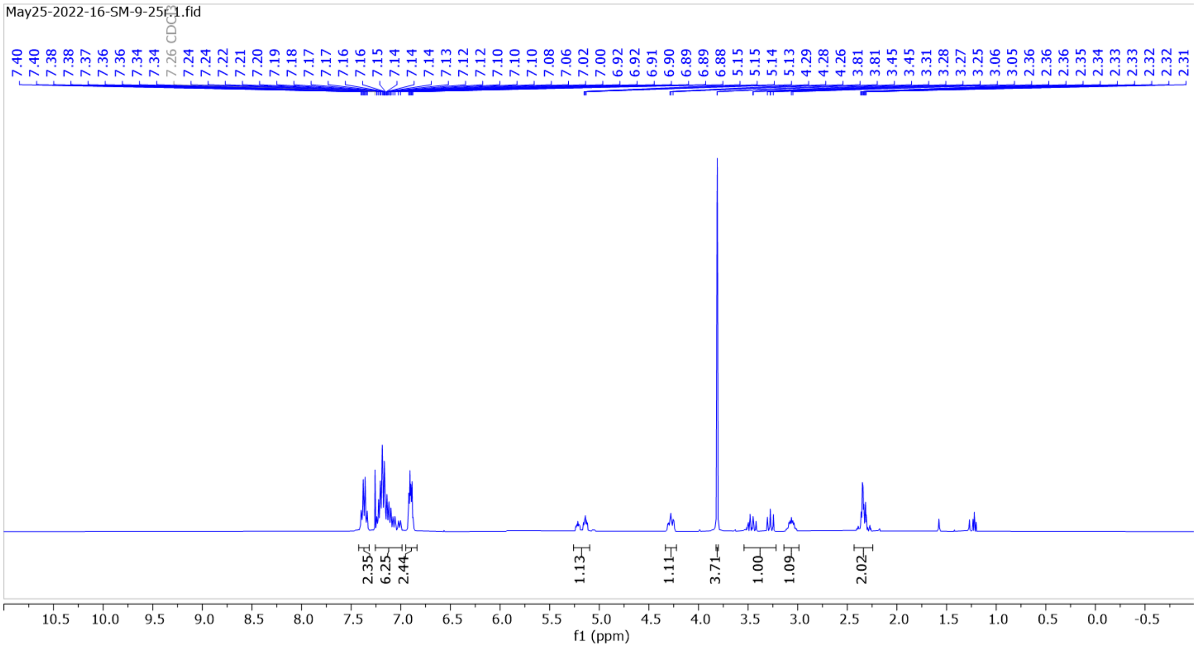

^{1}H NMR (400 MHZ, $CDCl_3$) of **3e**

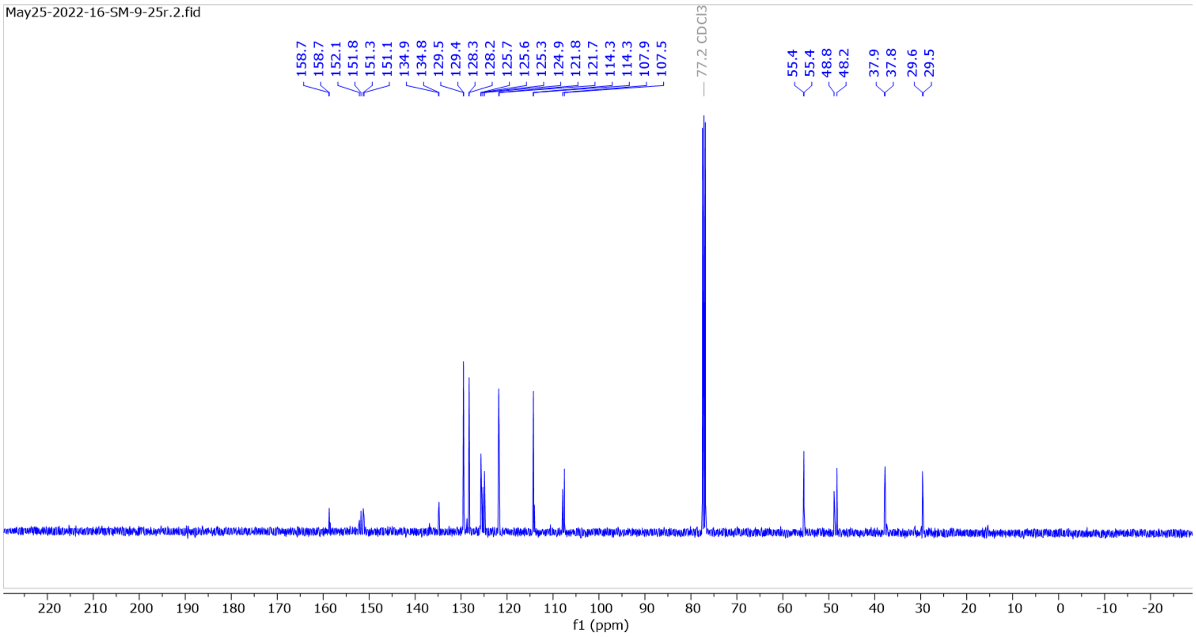

^{13}C NMR (101 MHZ, $CDCl_3$) of **3e**

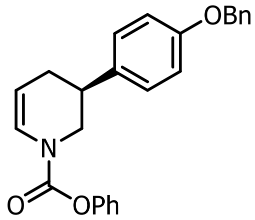

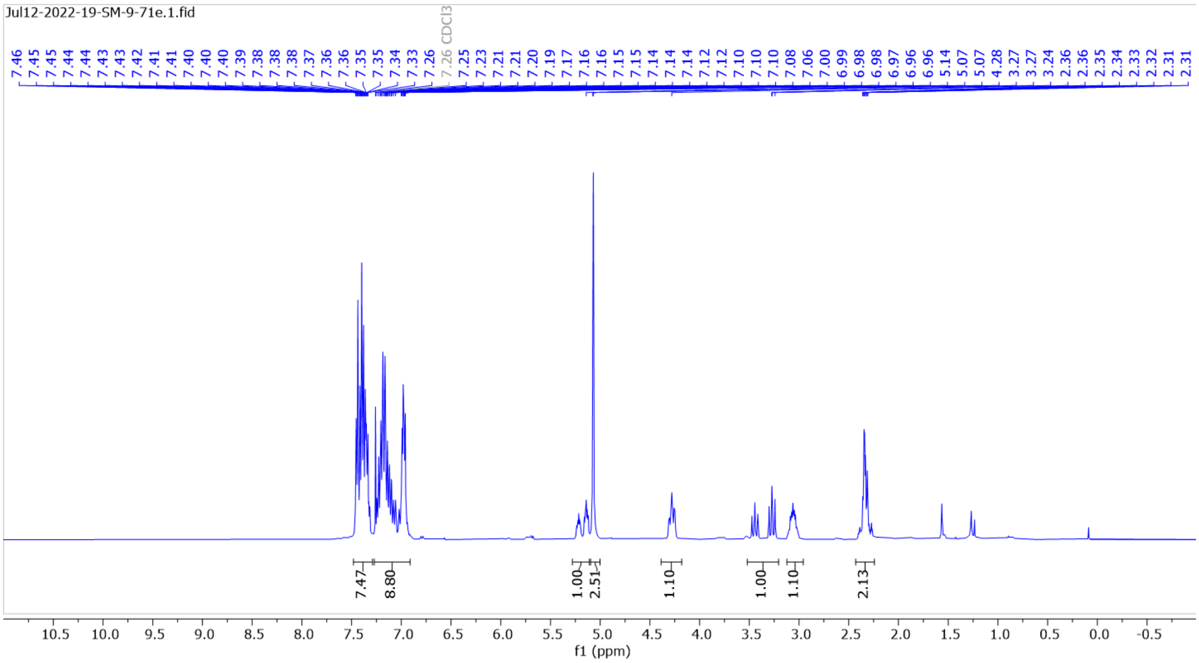

^{1}H NMR (400 MHZ, $CDCl_3$) of **3f**

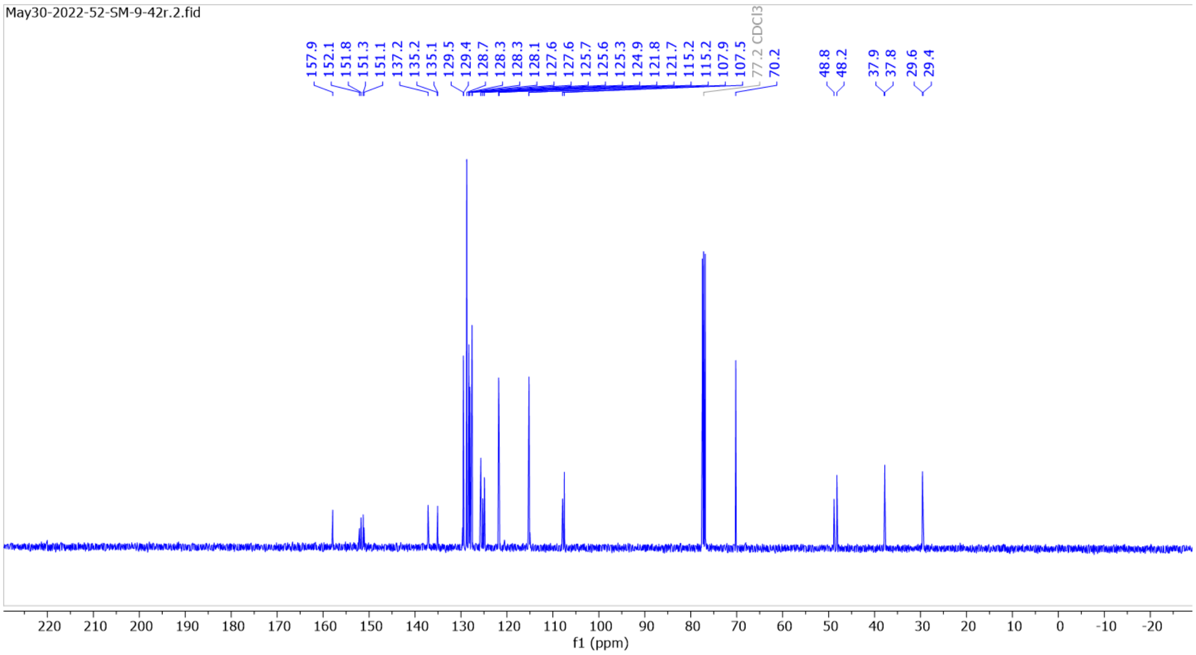

^{13}C NMR (101 MHZ, $CDCl_3$) of **3f**

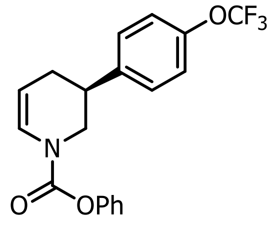

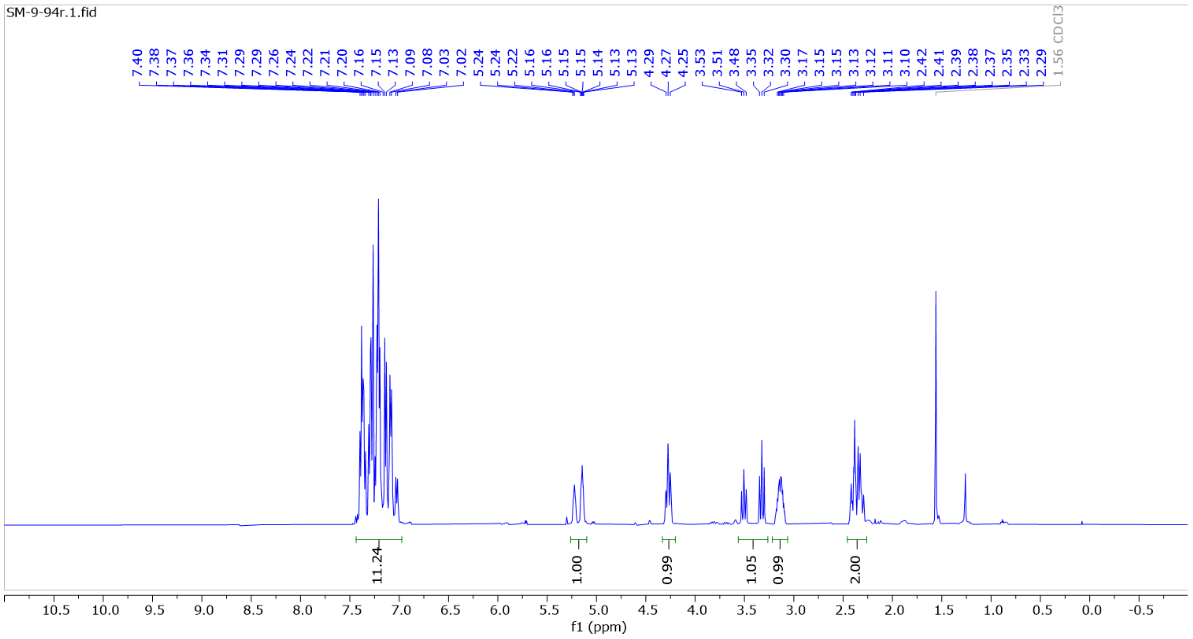

^{1}H NMR (500 MHZ, $CDCl_3$) of **3g**

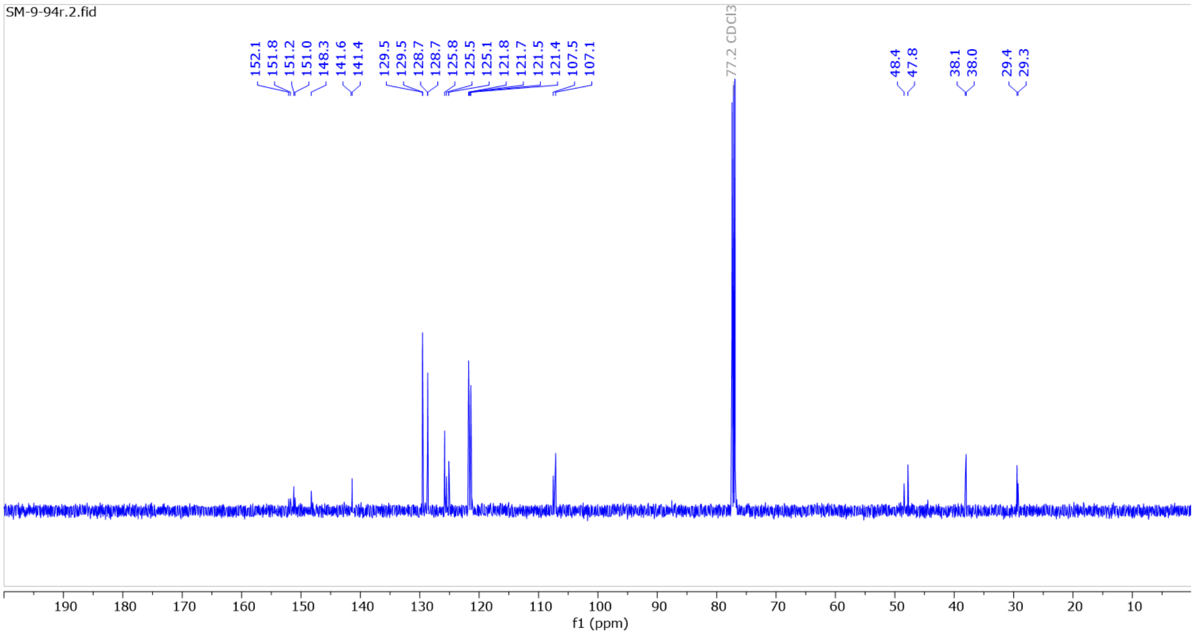

^{13}C NMR (126 MHZ, $CDCl_3$) of **3g**

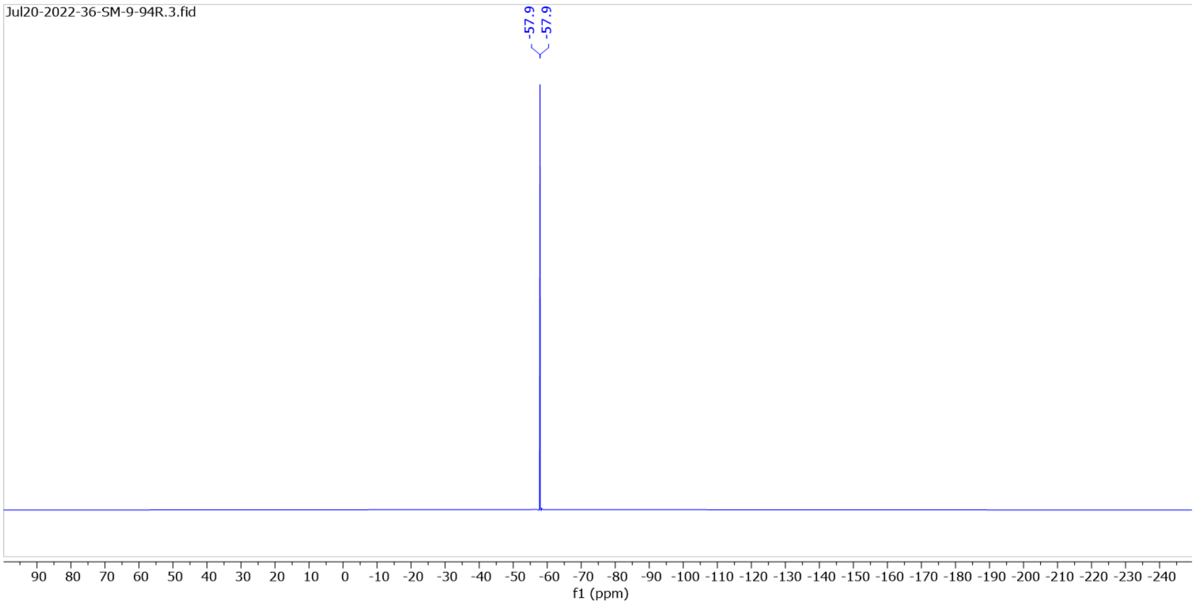

^{19}F NMR (376 MHZ, $CDCl_3$) of **3g**

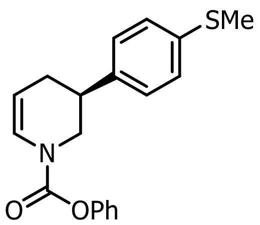

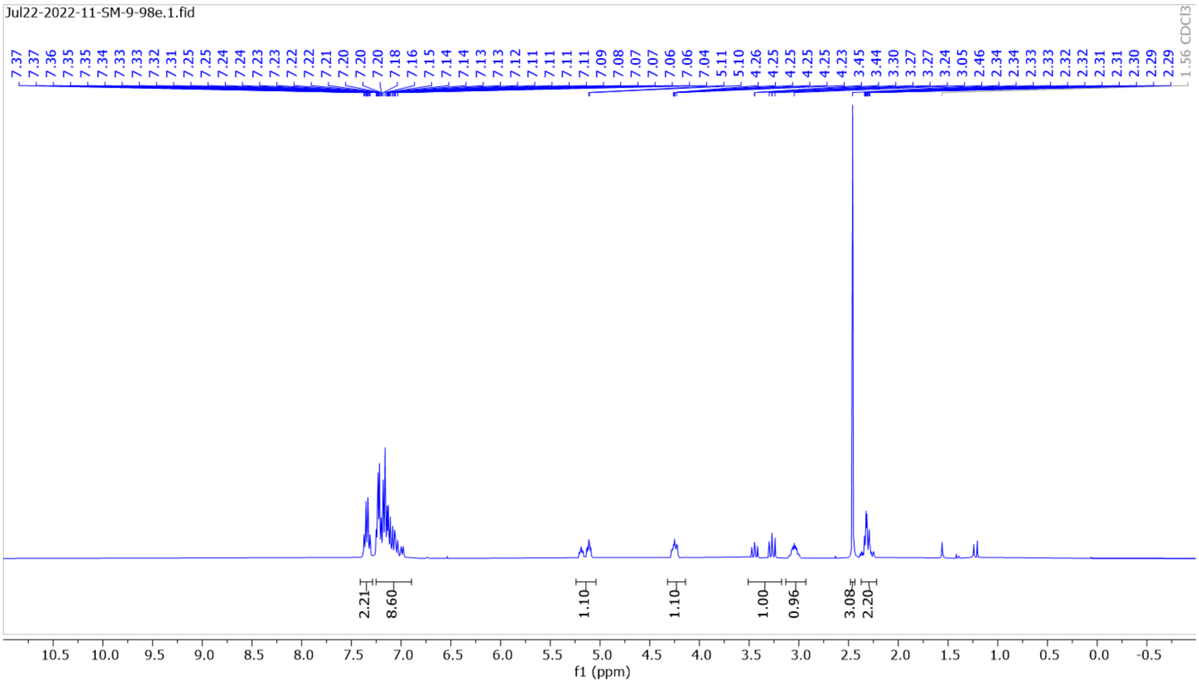

^{1}H NMR (400 MHZ, $CDCl_3$) of **3h**

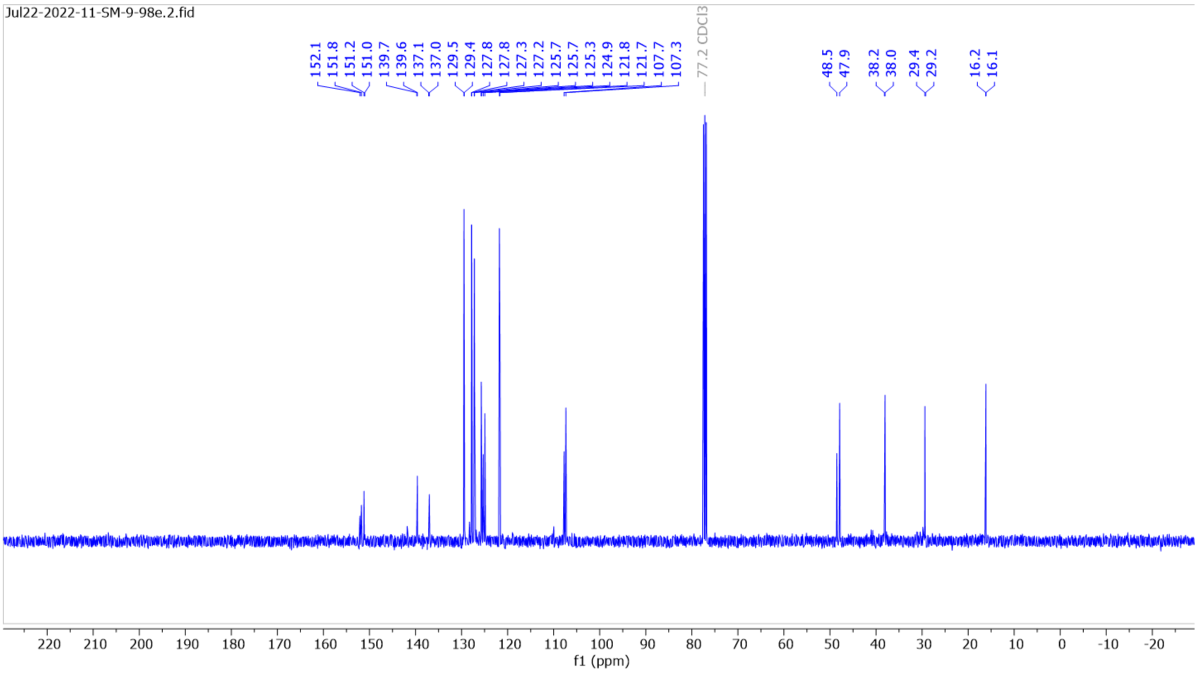

^{13}C NMR (101 MHZ, $CDCl_3$) of **3h**

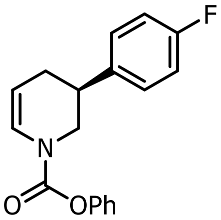

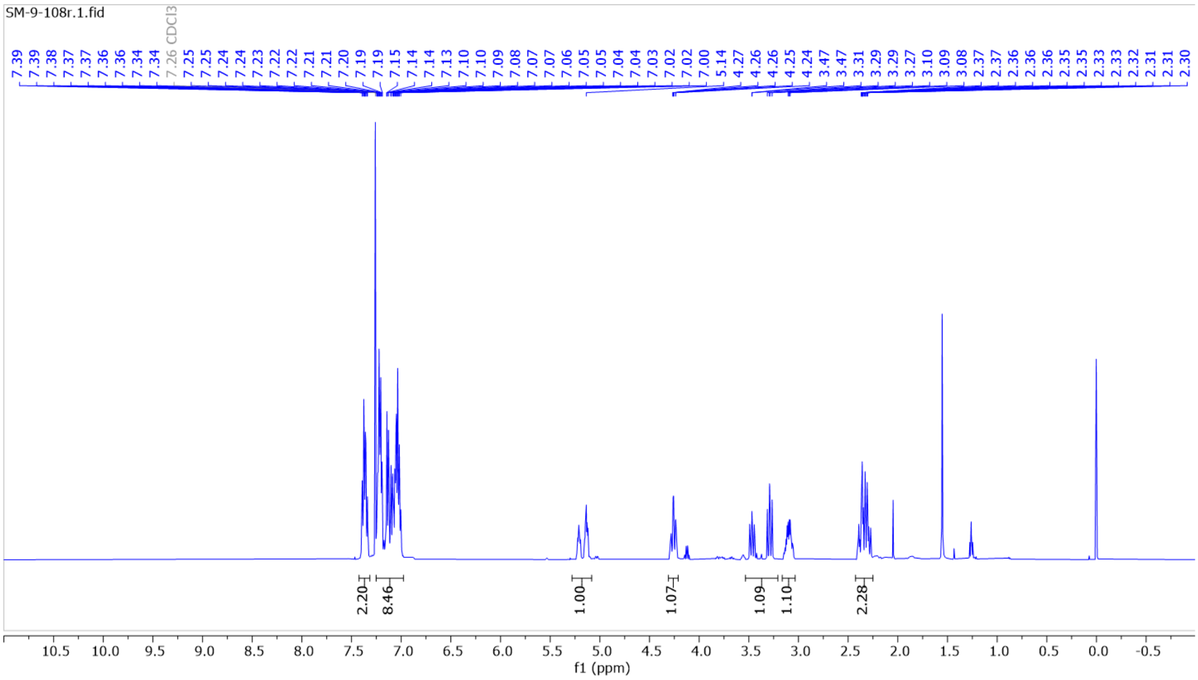

^{1}H NMR (500 MHZ, $CDCl_3$) of **3i**

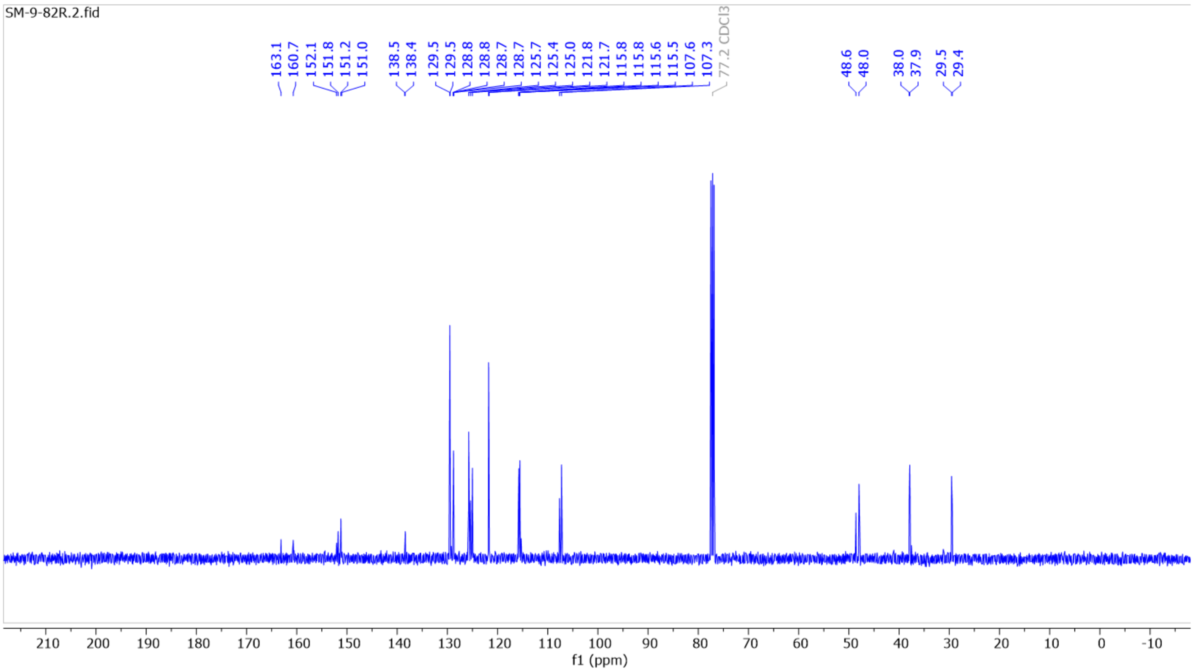

^{13}C NMR (126 MHZ, $CDCl_3$) of **3i**

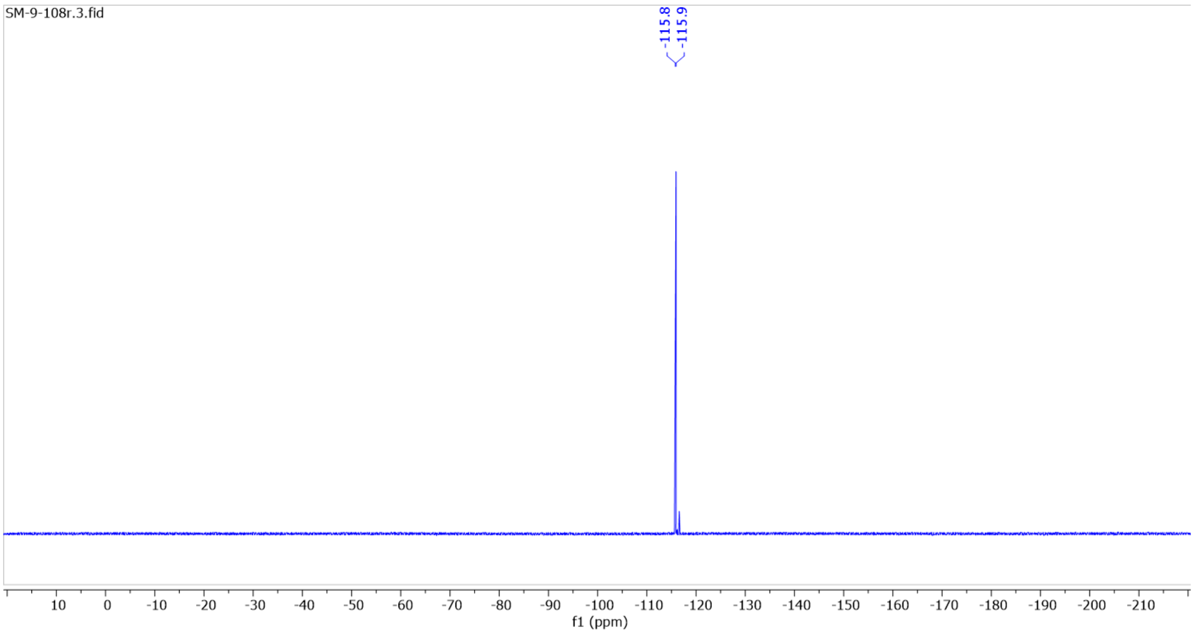

^{19}F NMR (471 MHZ, $CDCl_3$) of **3i**

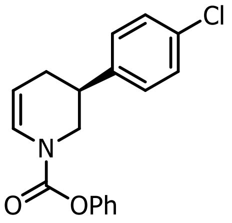

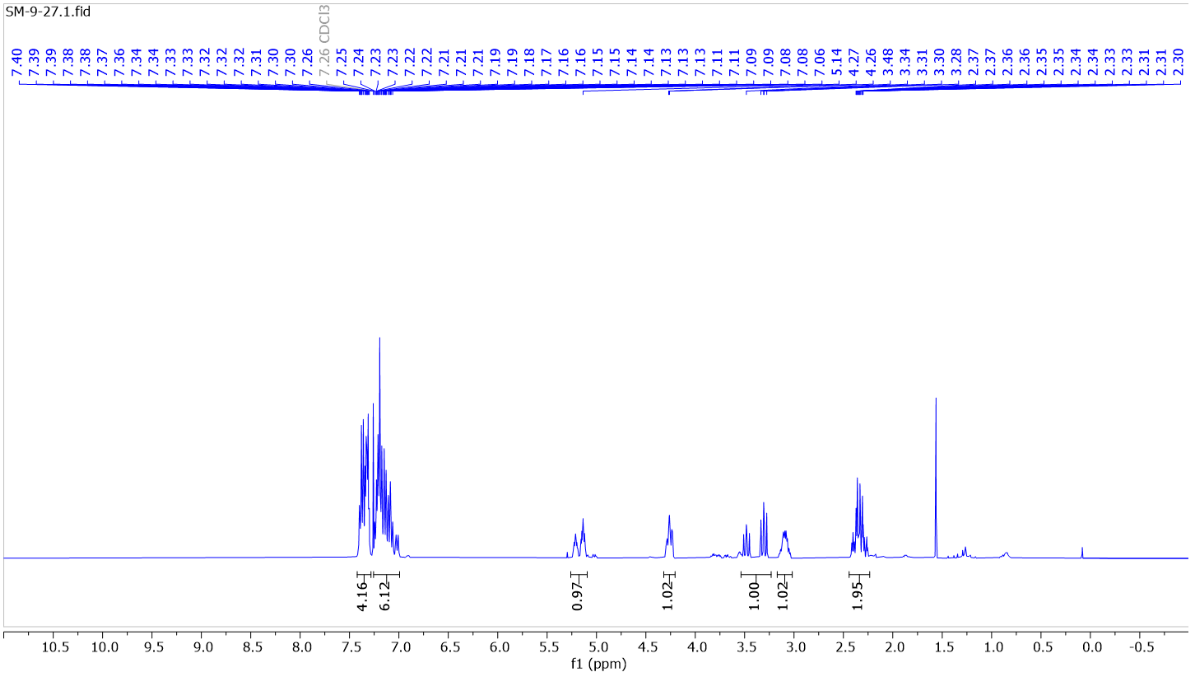

^{1}H NMR (400 MHZ, $CDCl_3$) of **3j**

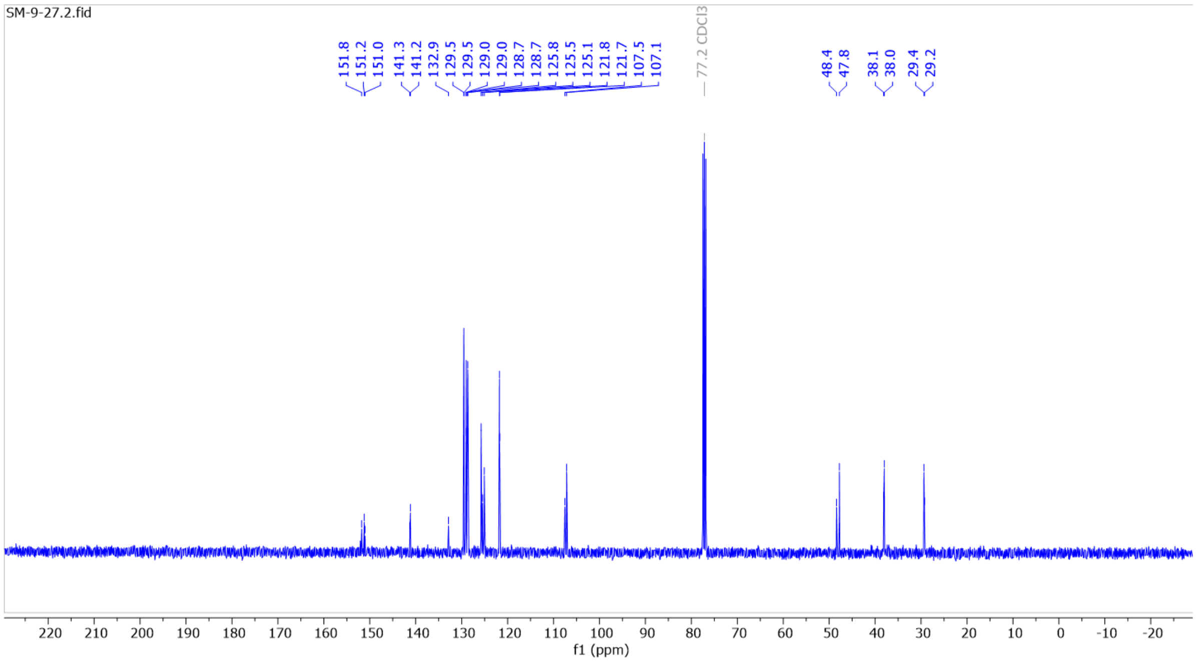

^{13}C NMR (101 MHZ, $CDCl_3$) of **3j**

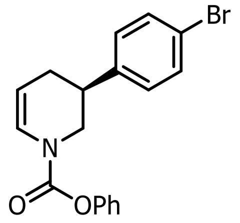

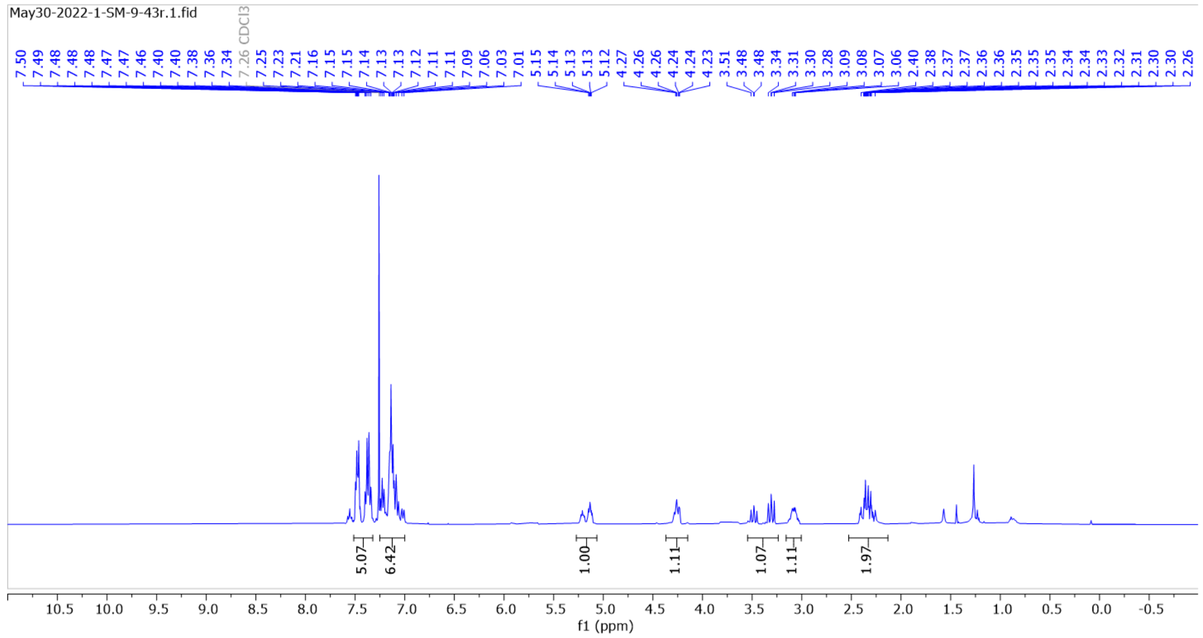

^{1}H NMR (400 MHZ, $CDCl_3$) of **3k**

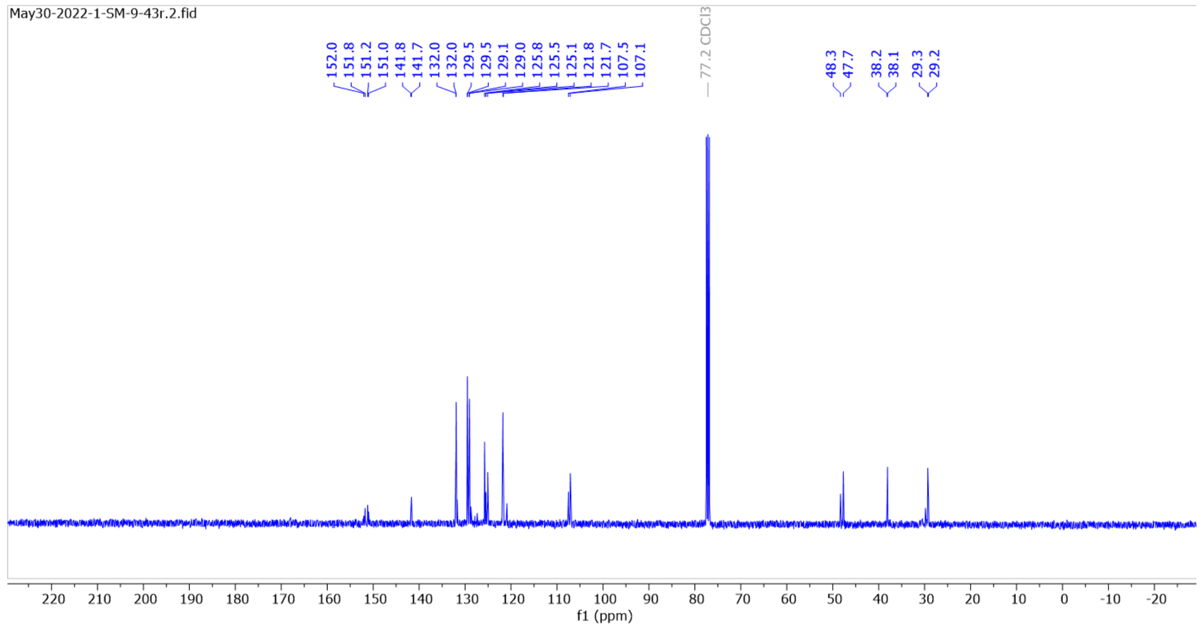

^{13}C NMR (101 MHZ, $CDCl_3$) of **3k**

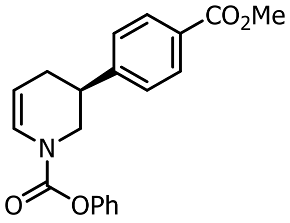

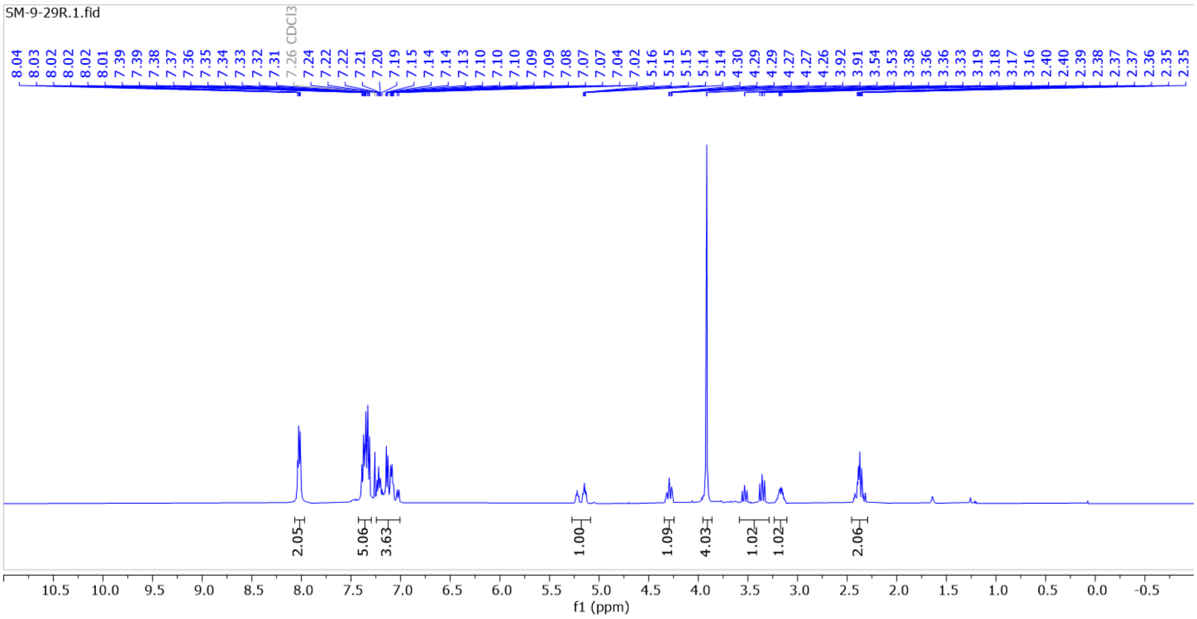

^{1}H NMR (500 MHZ, $CDCl_3$) of **3l**

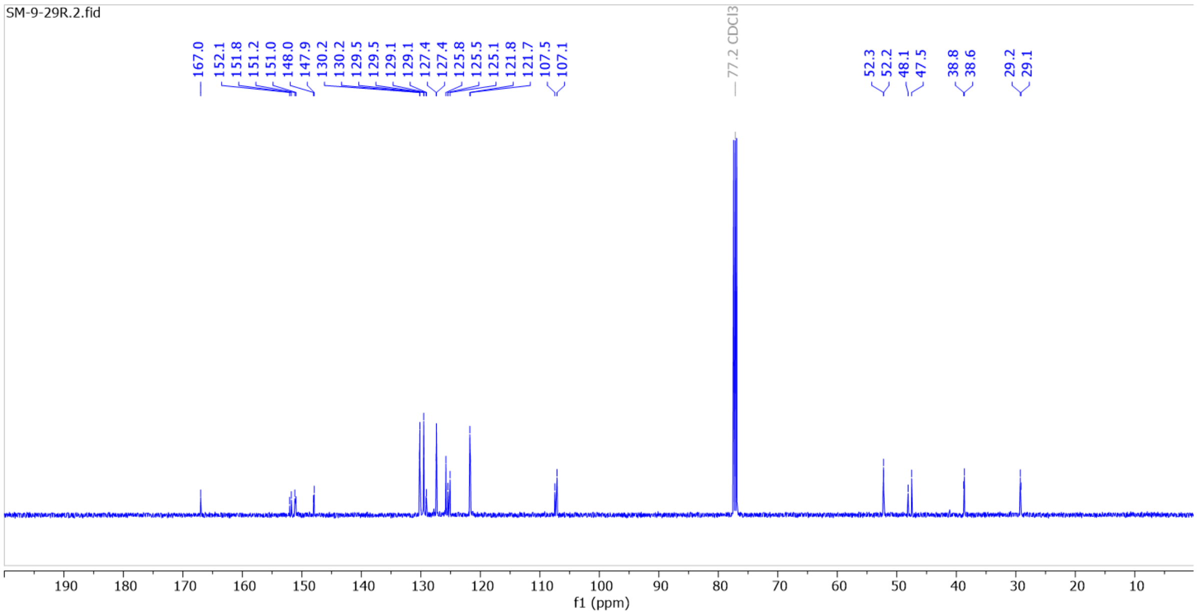

^{13}C NMR (126 MHZ, $CDCl_3$) of **3l**

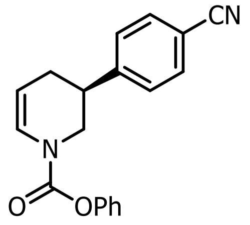

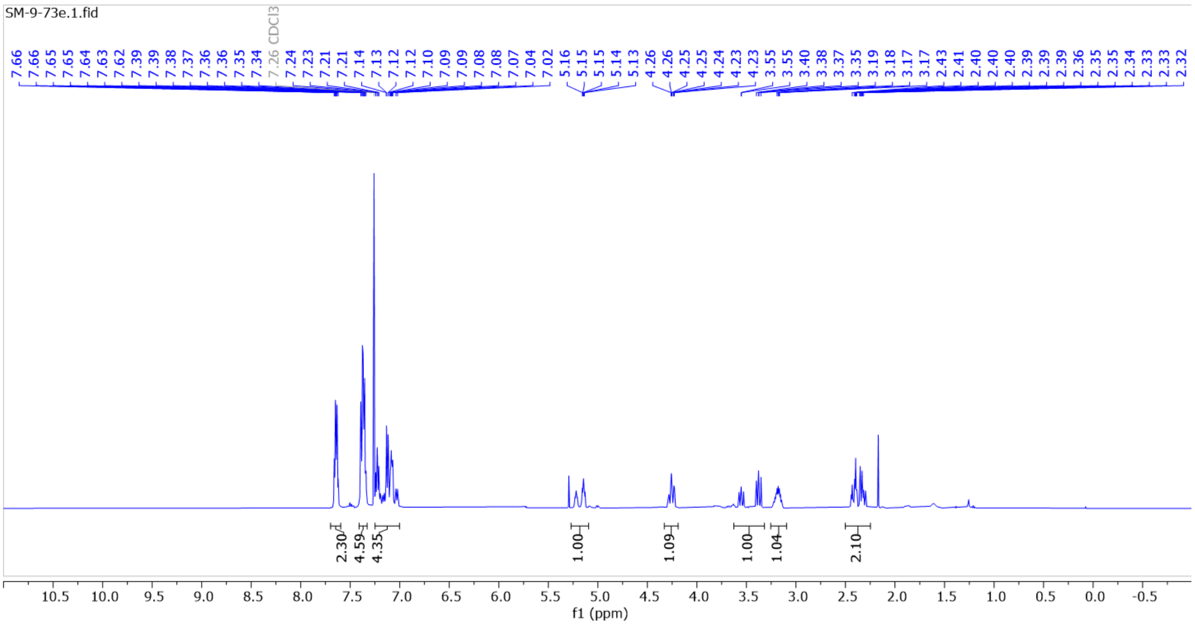

^{1}H NMR (500 MHZ, $CDCl_3$) of **3m**

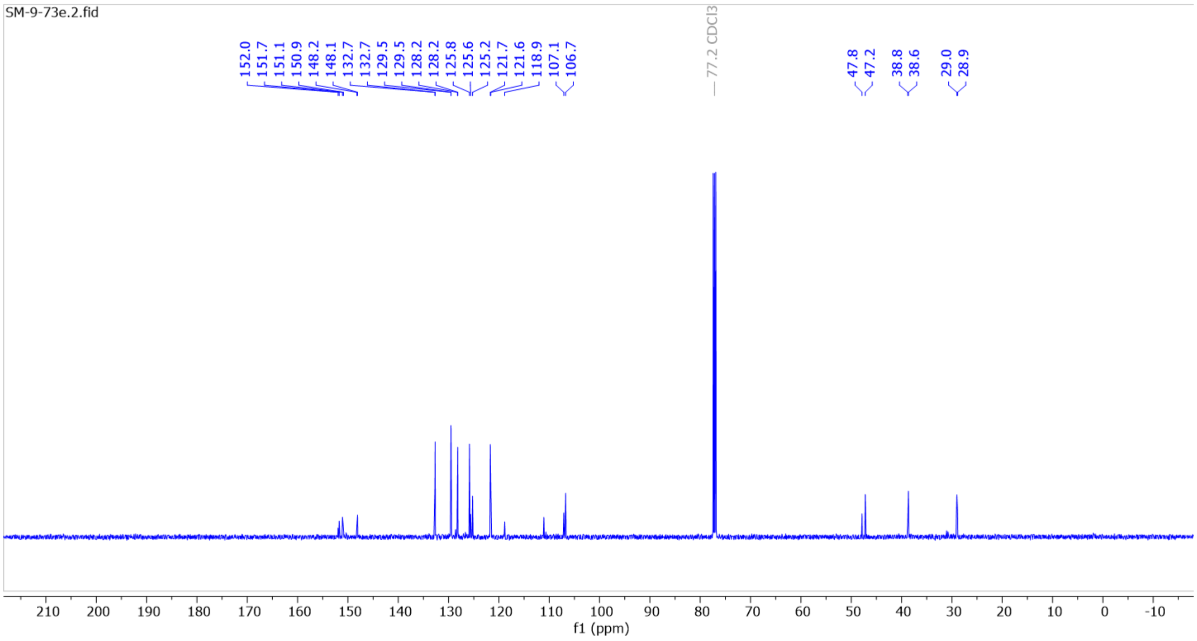

^{13}C NMR (126 MHZ, $CDCl_3$) of **3m**

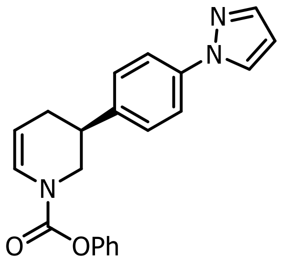

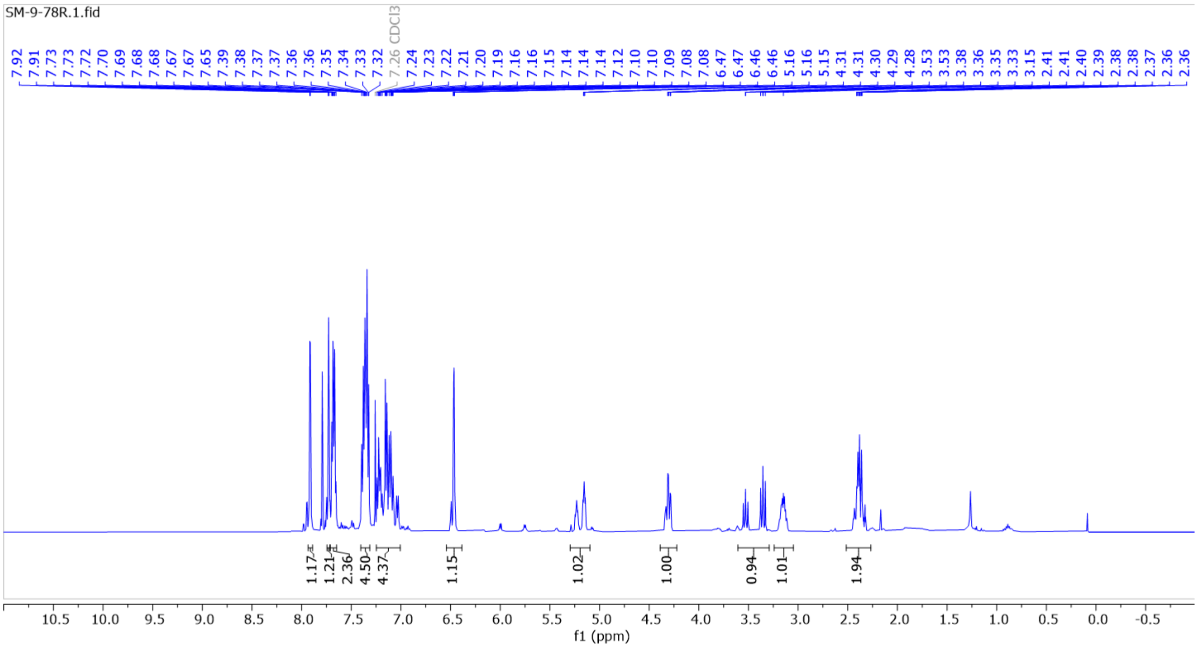

^{1}H NMR (500 MHZ, $CDCl_3$) of **3n**

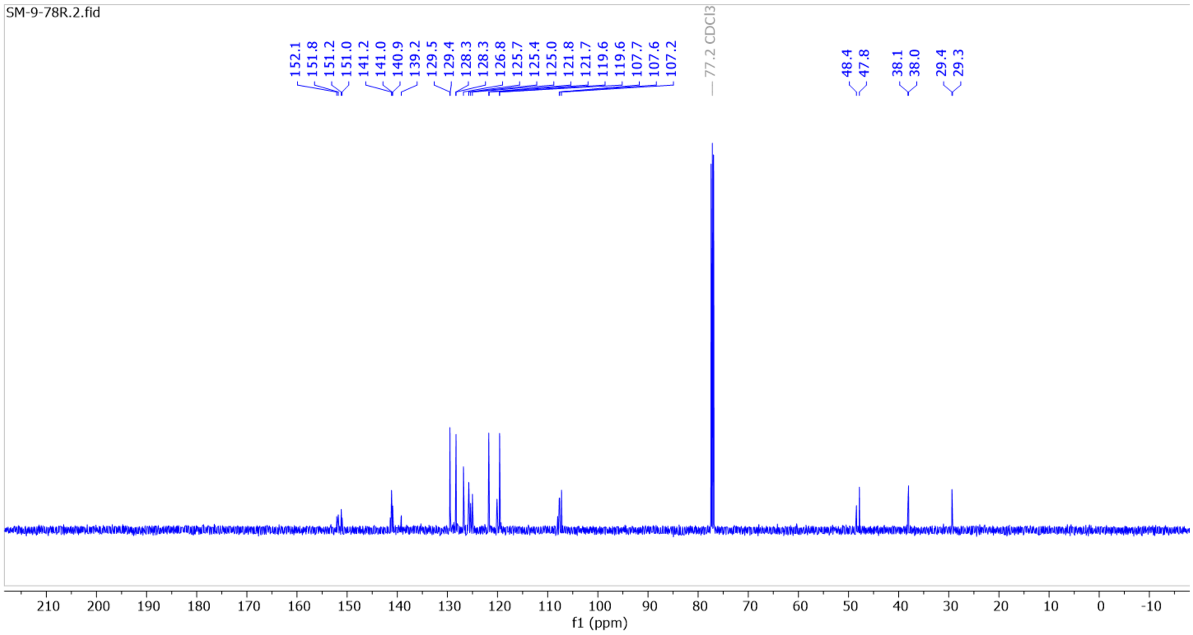

^{13}C NMR (126 MHZ, $CDCl_3$) of **3n**

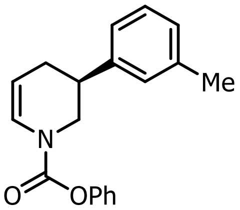

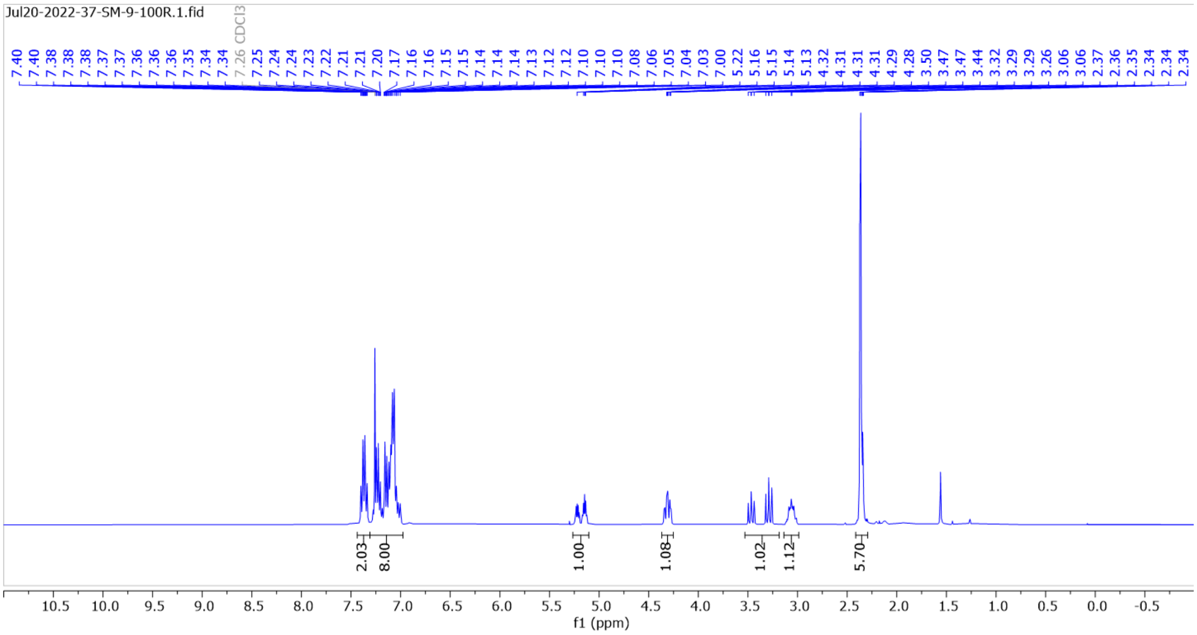

^{1}H NMR (400 MHZ, $CDCl_3$) of **3o**

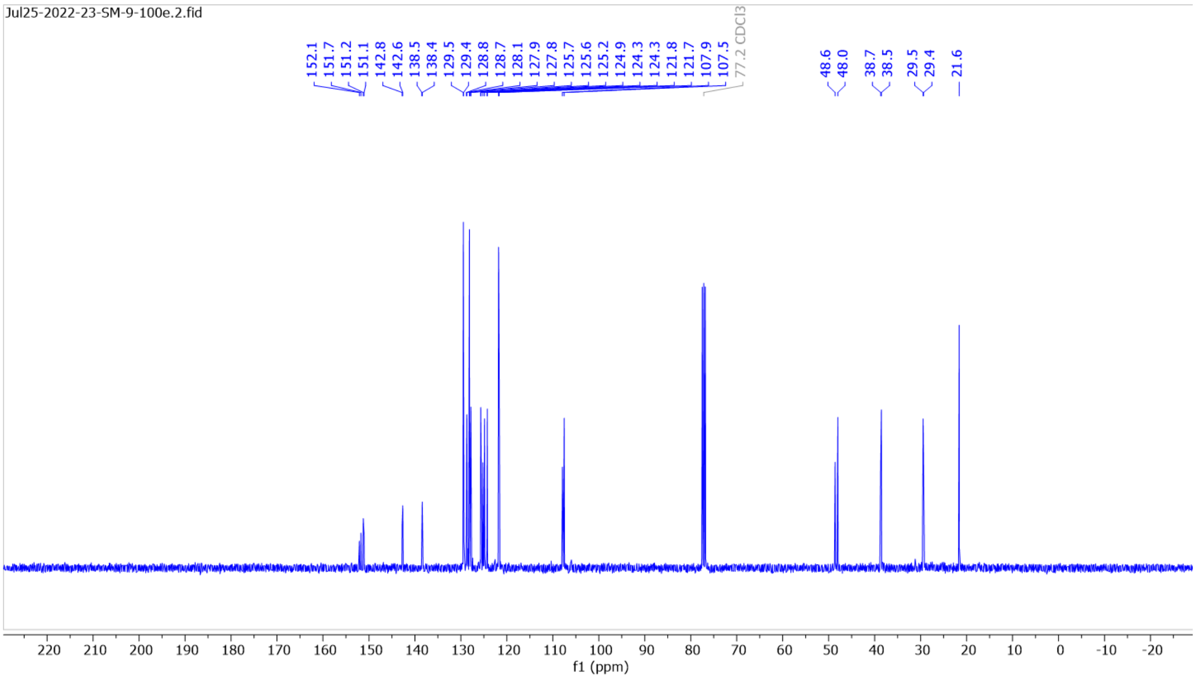

^{13}C NMR (101 MHZ, $CDCl_3$) of **3o**

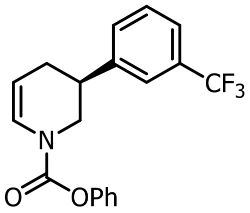

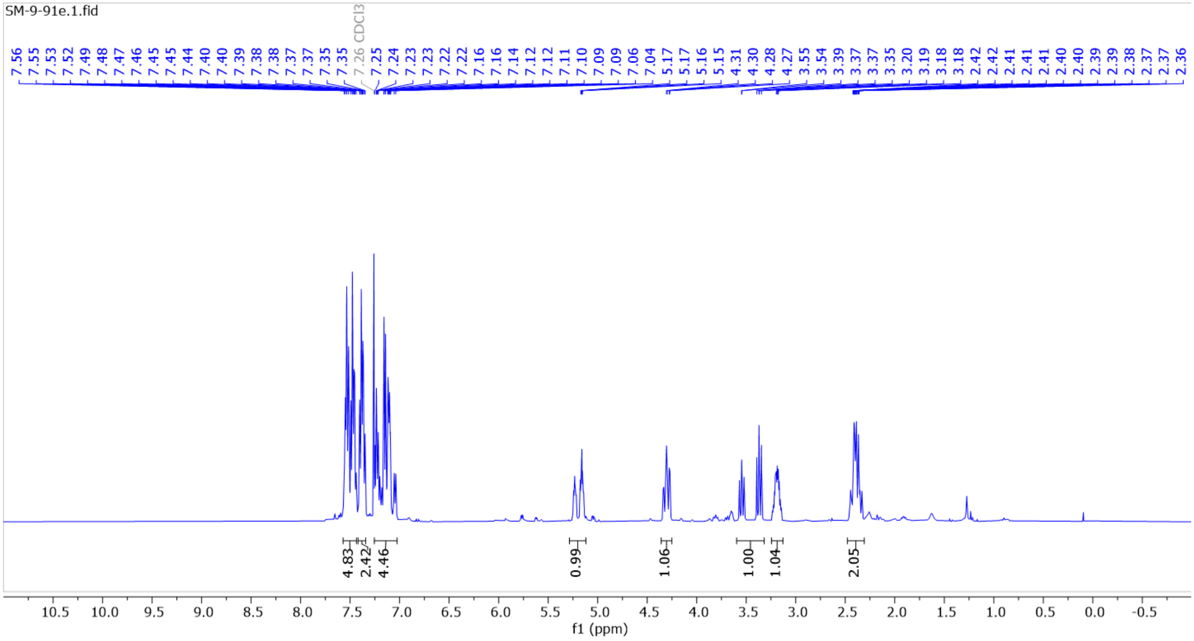

^{1}H NMR (500 MHZ, $CDCl_3$) of **3p**

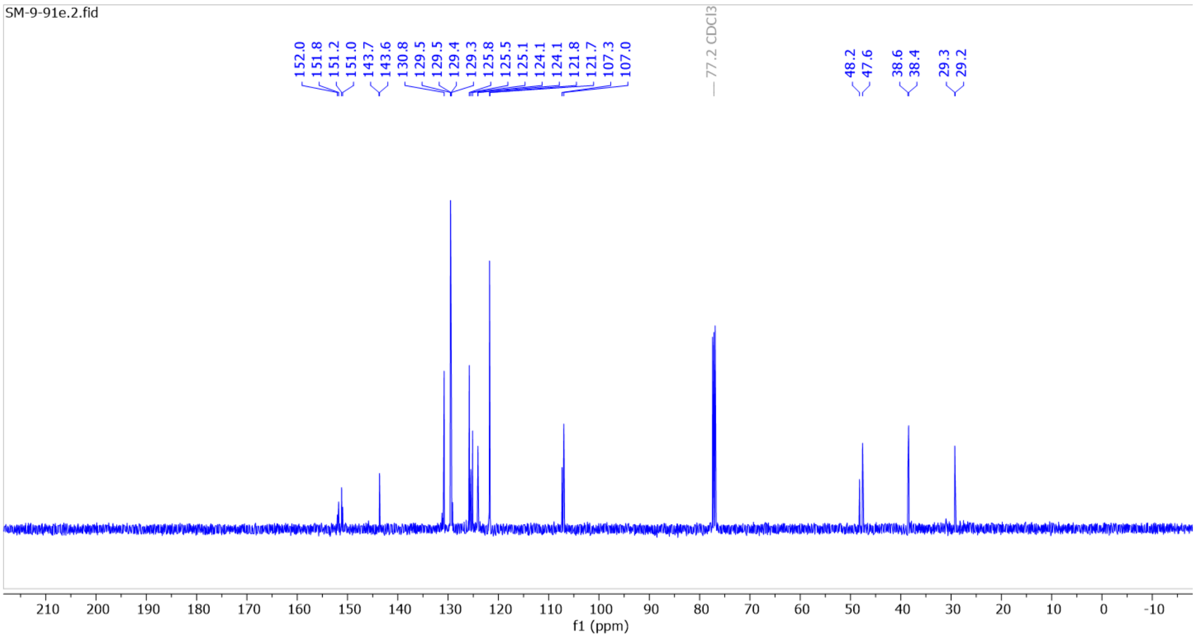

^{13}C NMR (126 MHZ, $CDCl_3$) of **3p**

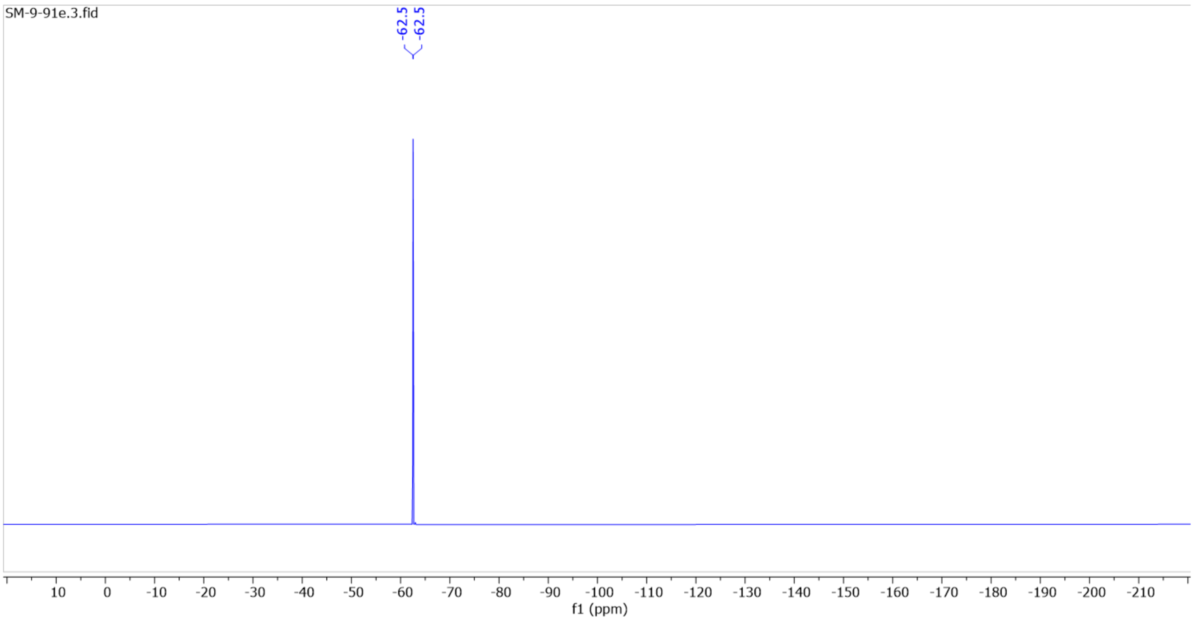

^{19}F NMR (471 MHZ, $CDCl_3$) of **3p**

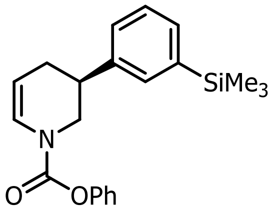

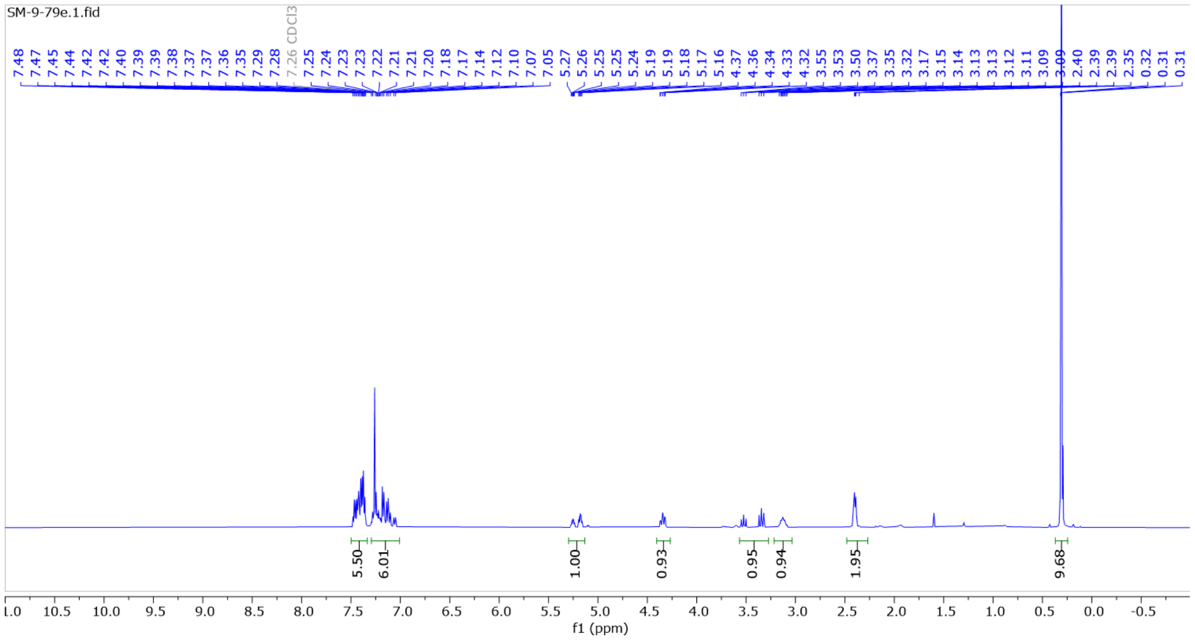

^{1}H NMR (500 MHZ, $CDCl_3$) of **3q**

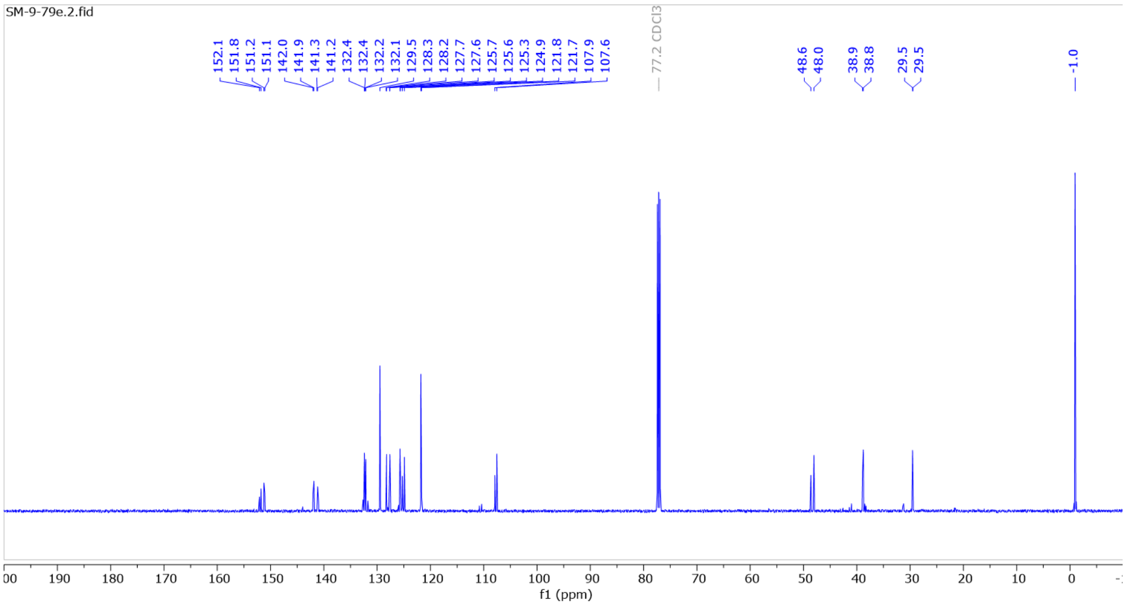

^{13}C NMR (101 MHZ, $CDCl_3$) of **3q**

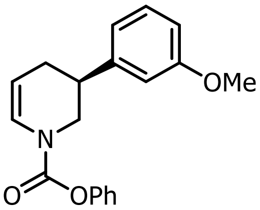

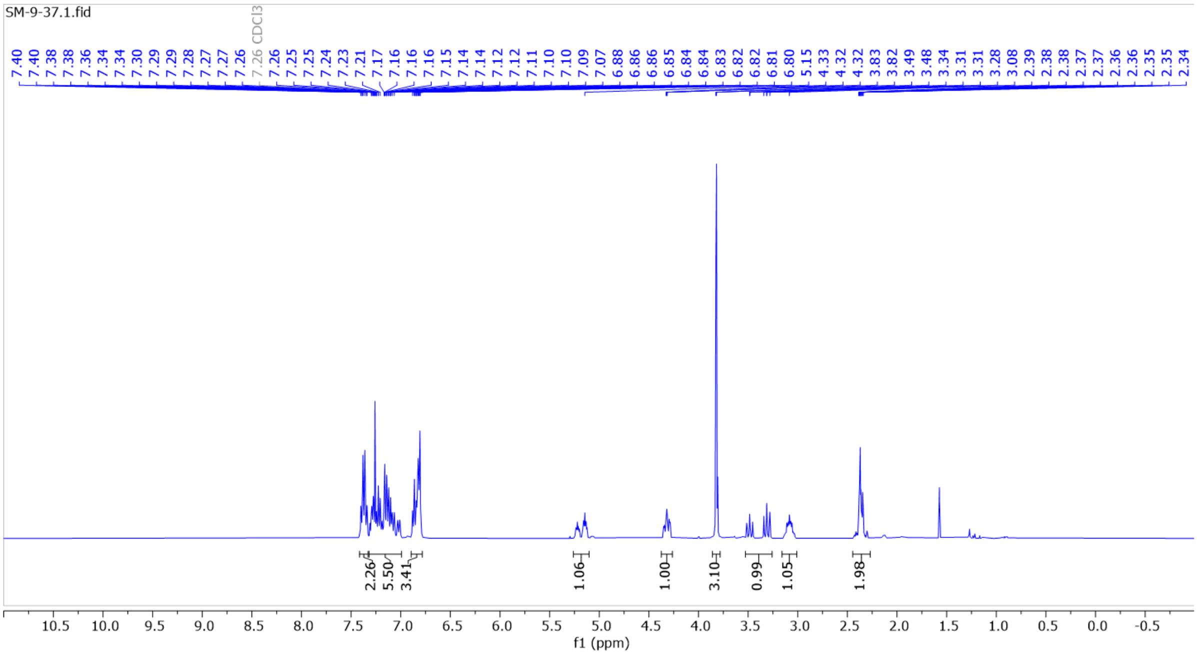

^{1}H NMR (400 MHZ, $CDCl_3$) of **3r**

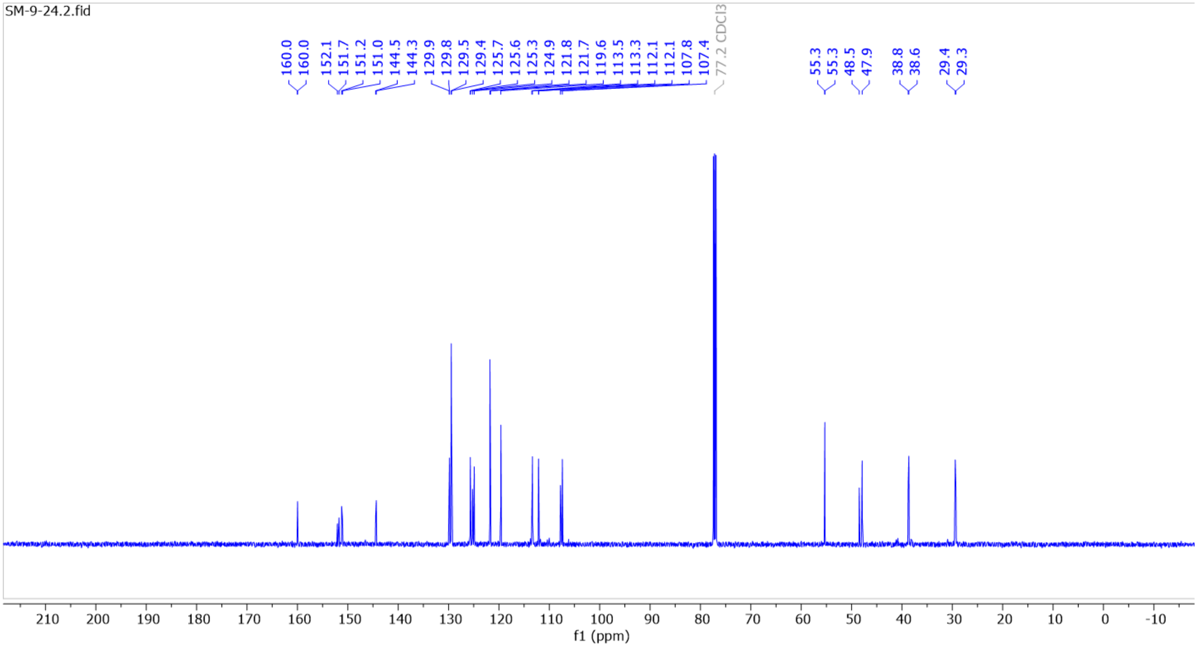

^{13}C NMR (126 MHZ, $CDCl_3$) of **3r**

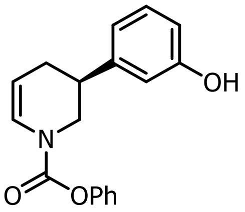

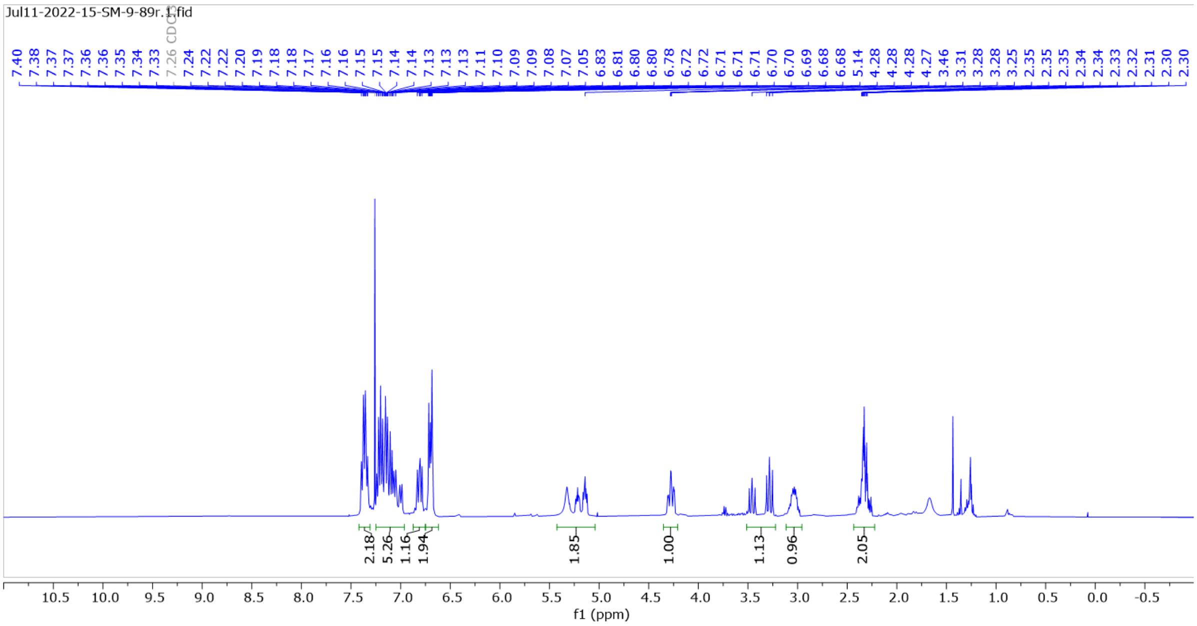

^{1}H NMR (400 MHZ, $CDCl_3$) of **3s**

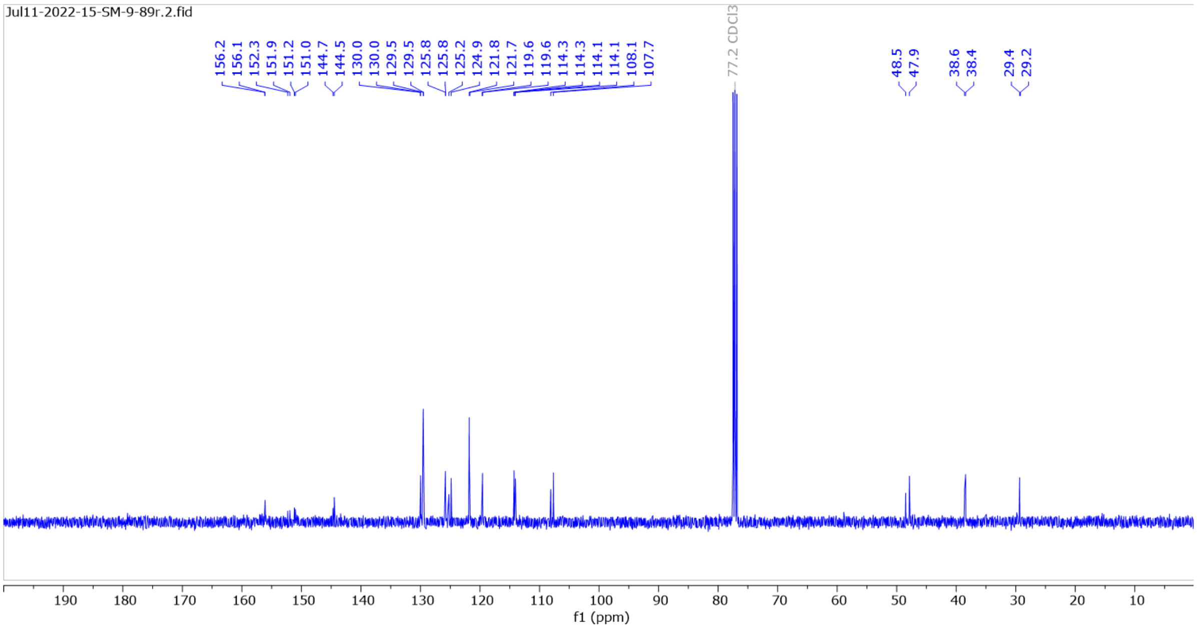

^{13}C NMR (101 MHZ, $CDCl_3$) of **3s**

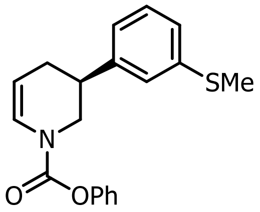

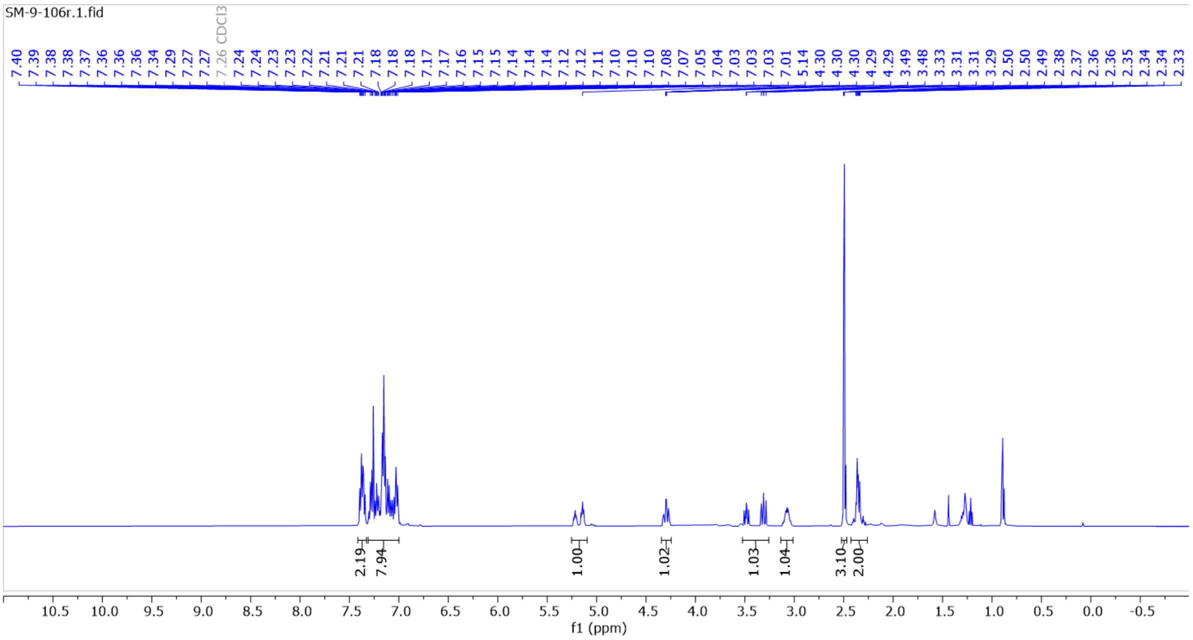

^{1}H NMR (500 MHZ, $CDCl_3$) of **3t**

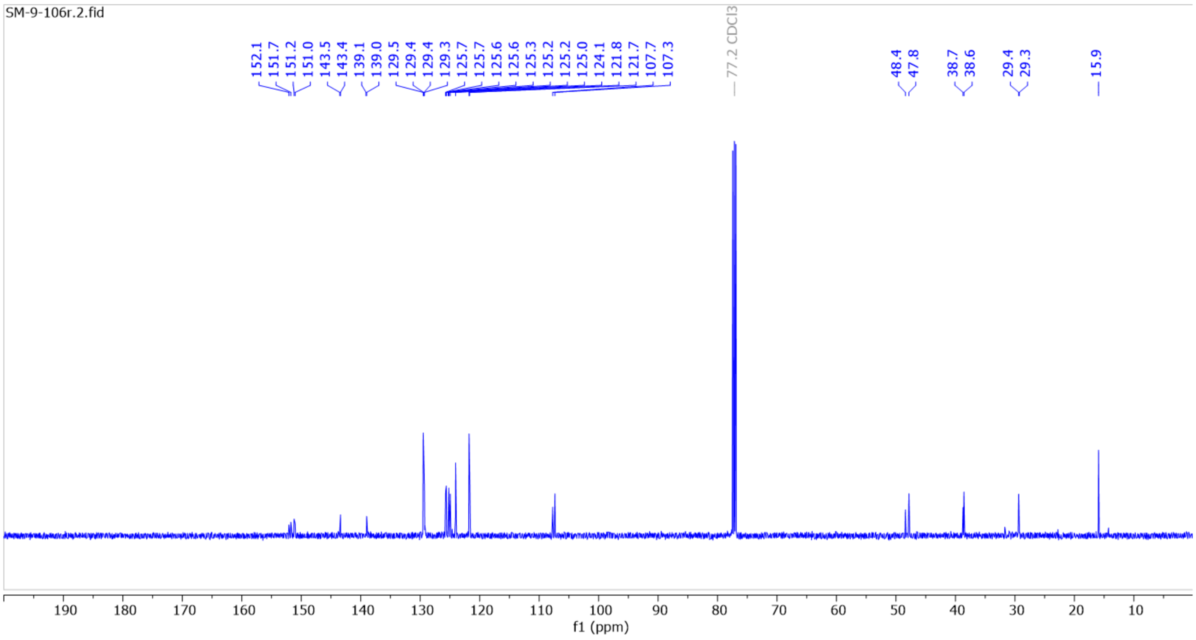

^{13}C NMR (126 MHZ, $CDCl_3$) of **3t**

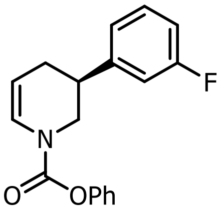

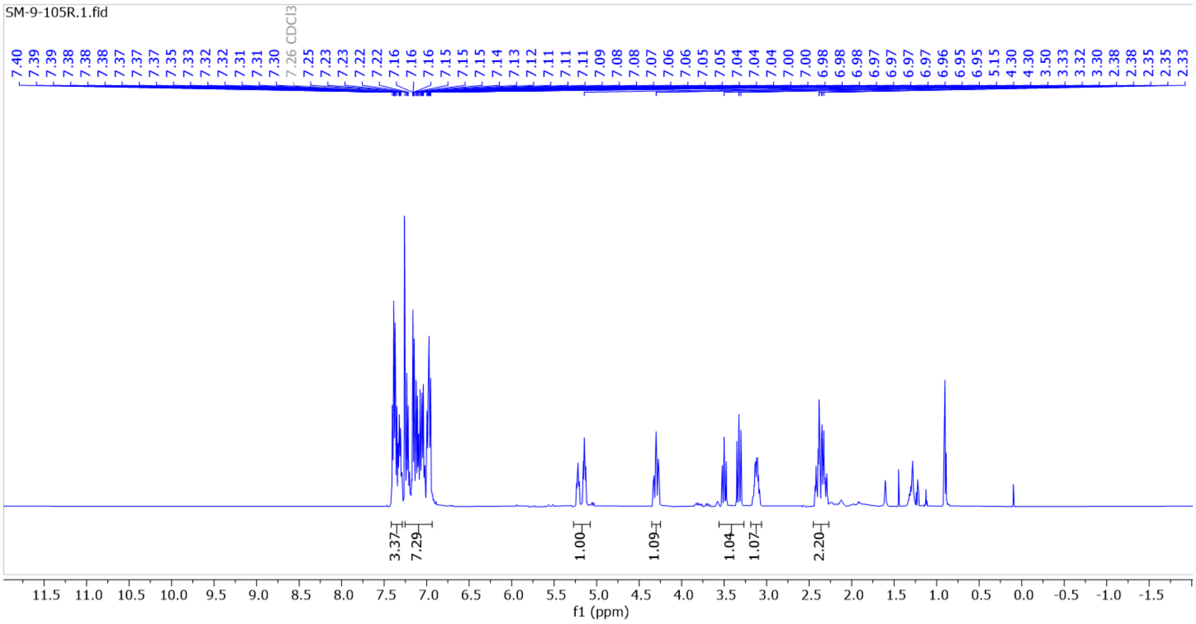

^{1}H NMR (500 MHZ, $CDCl_3$) of **3u**

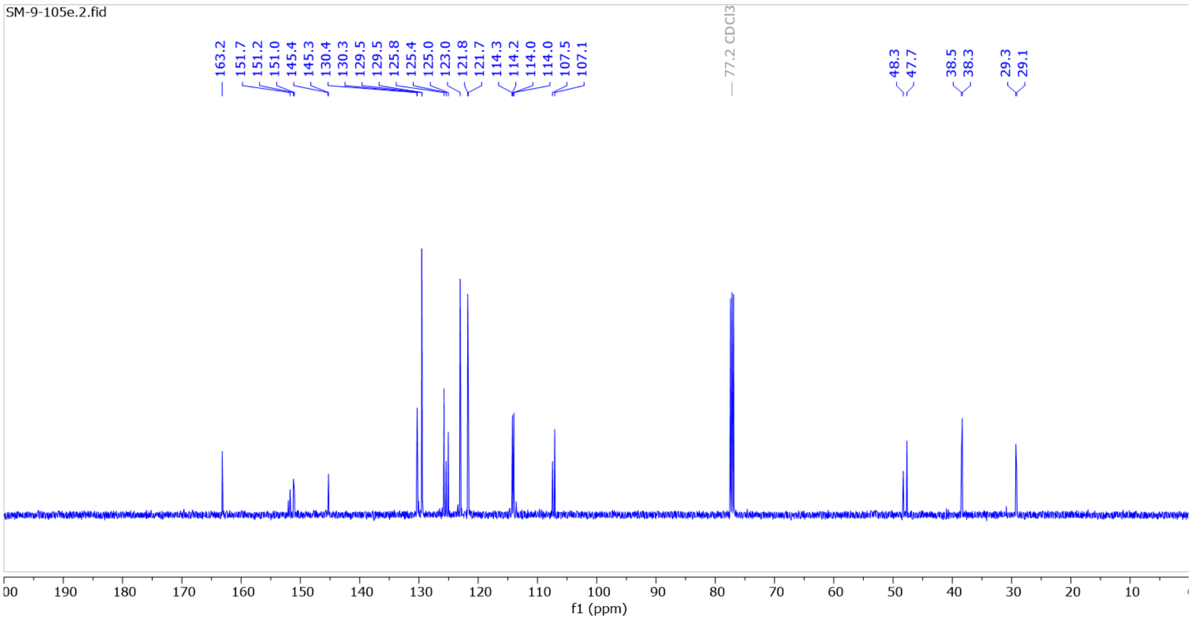

^{13}C NMR (126 MHZ, $CDCl_3$) of **3u**

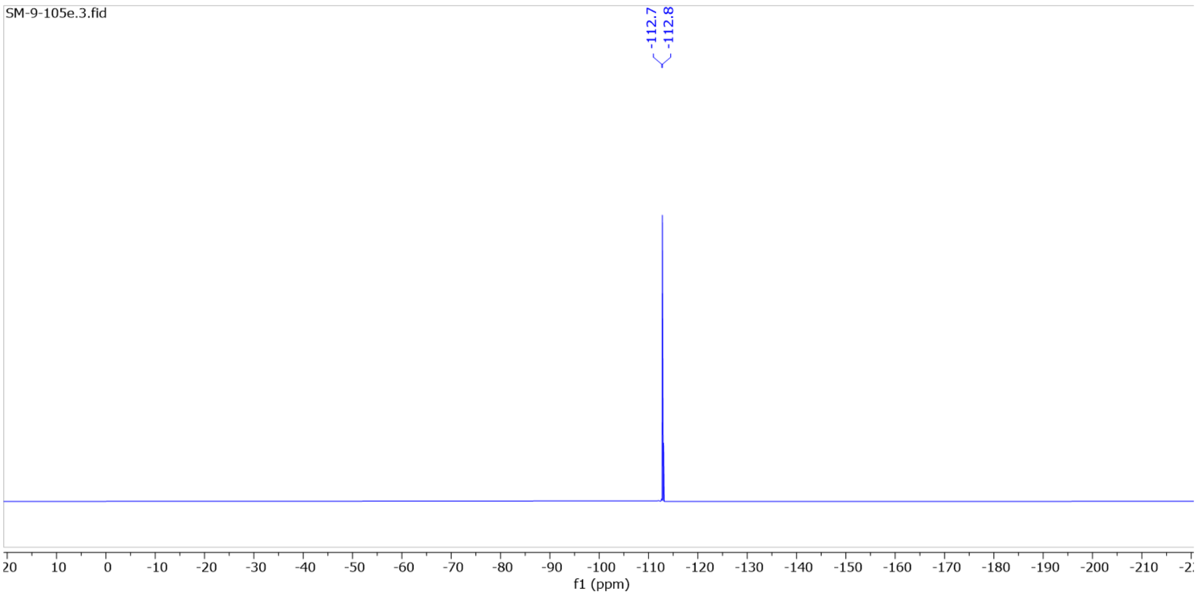

^{19}F NMR (471 MHZ, $CDCl_3$) of **3u**

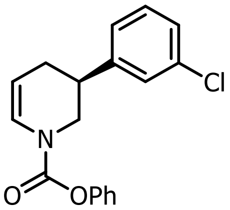

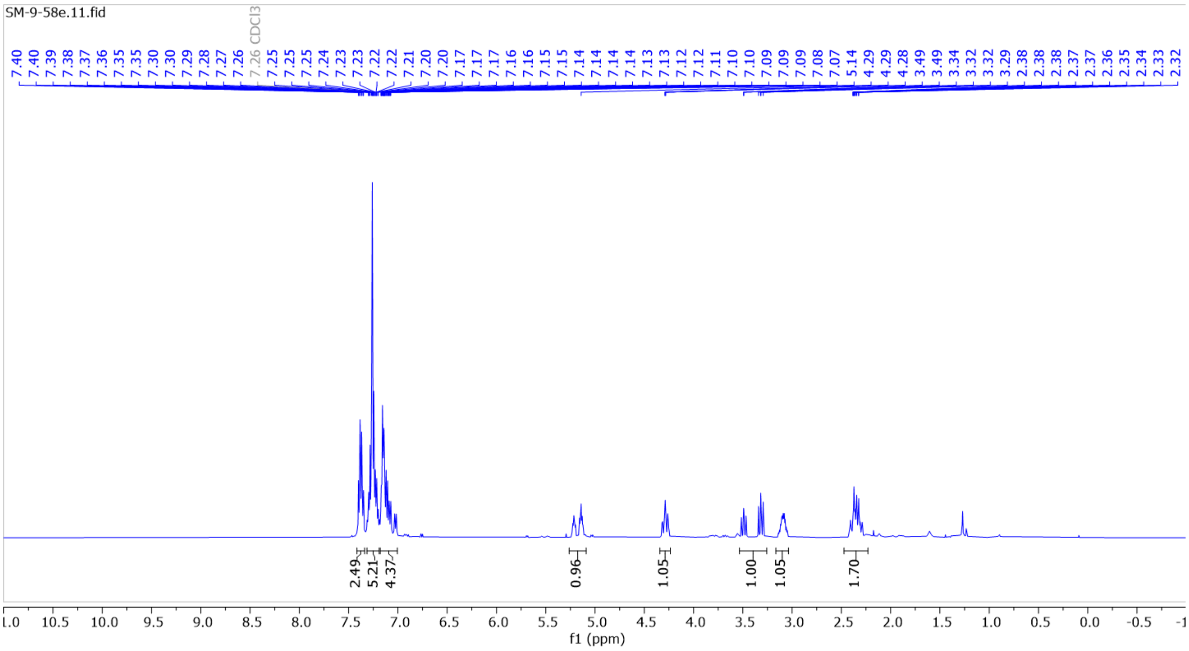

^{1}H NMR (500 MHZ, $CDCl_3$) of **3v**

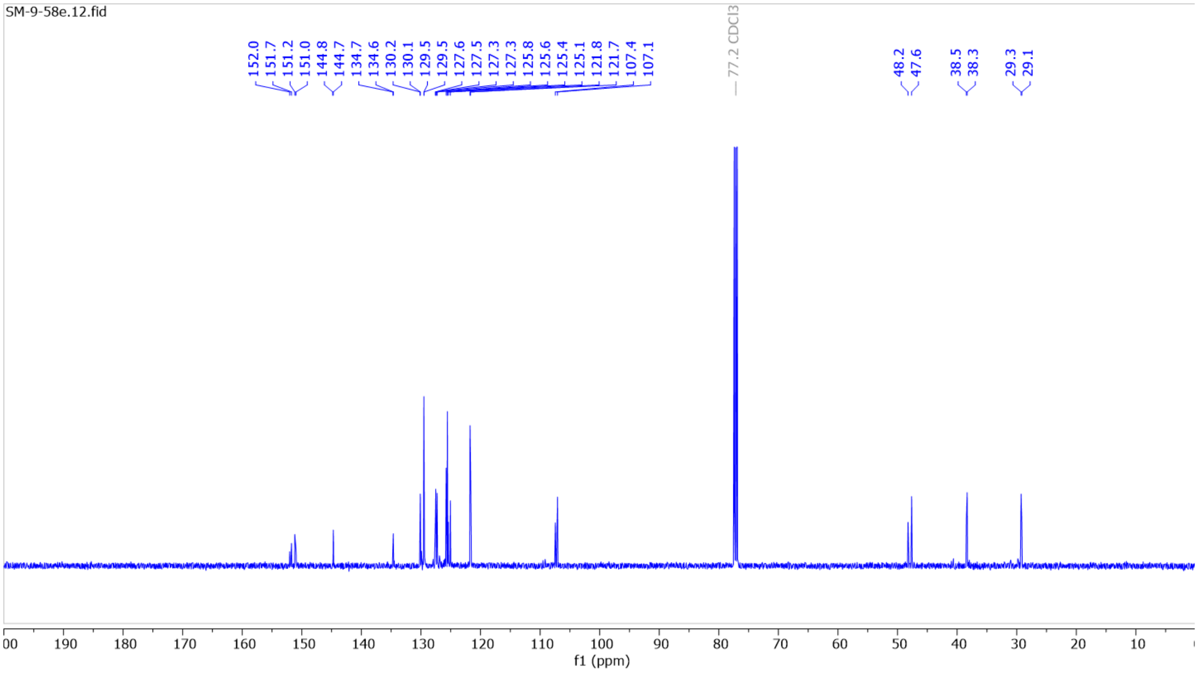

^{13}C NMR (126 MHZ, $CDCl_3$) of **3v**

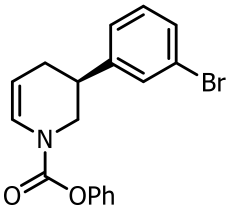

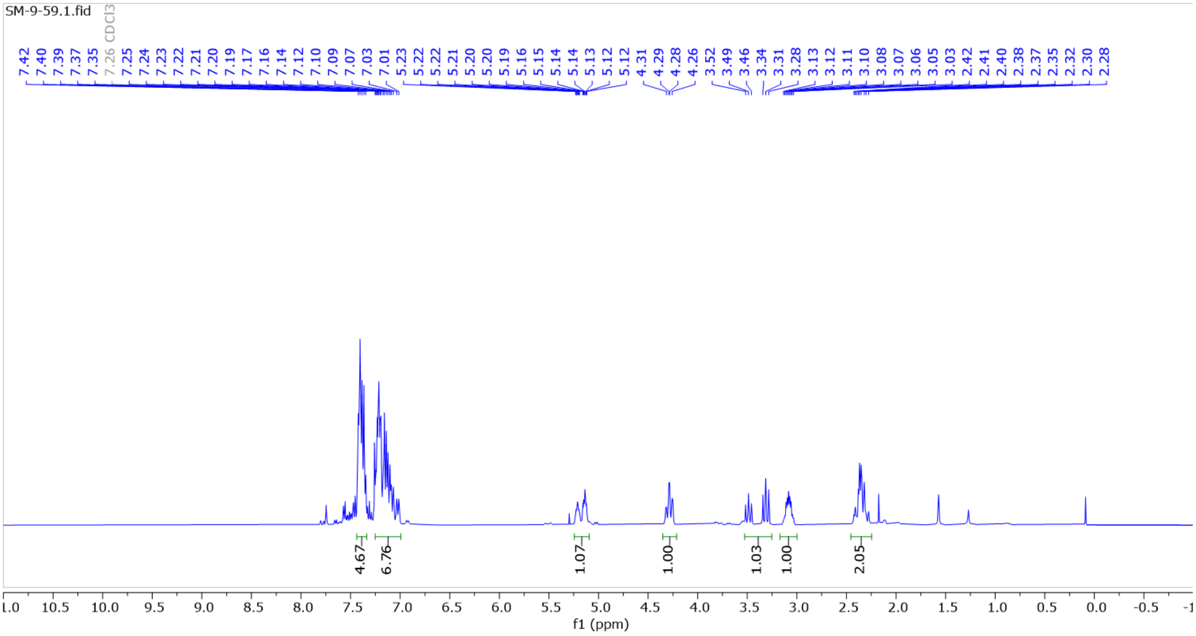

^{1}H NMR (400 MHZ, $CDCl_3$) of **3w**

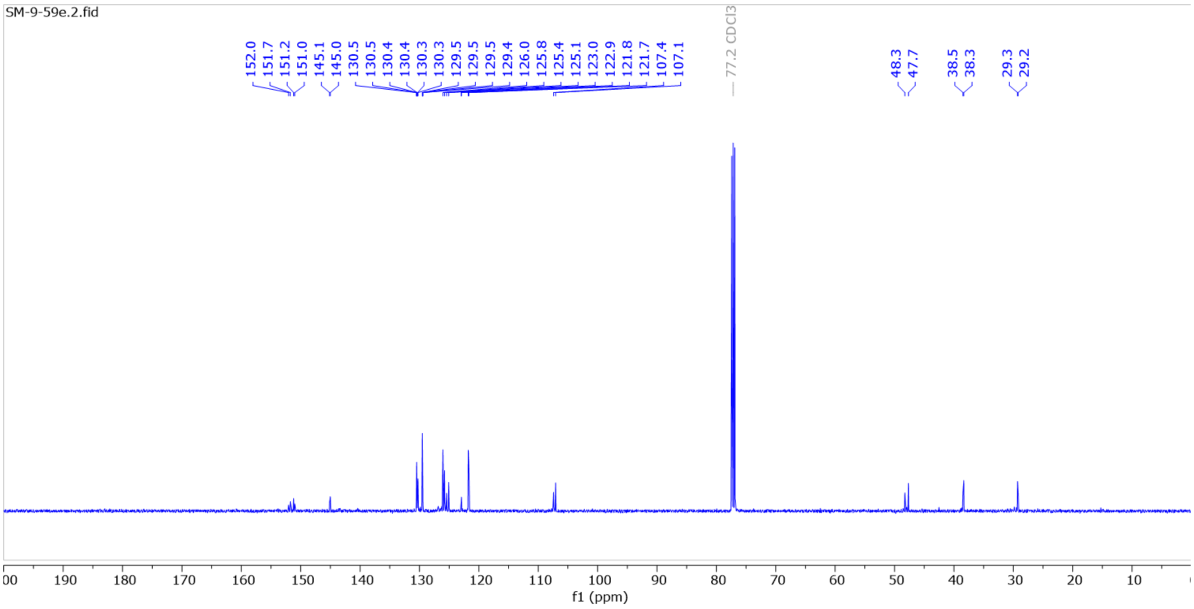

^{13}C NMR (126 MHZ, $CDCl_3$) of **3w**

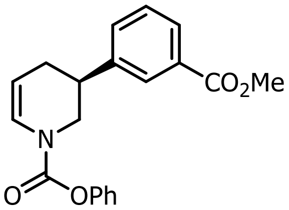

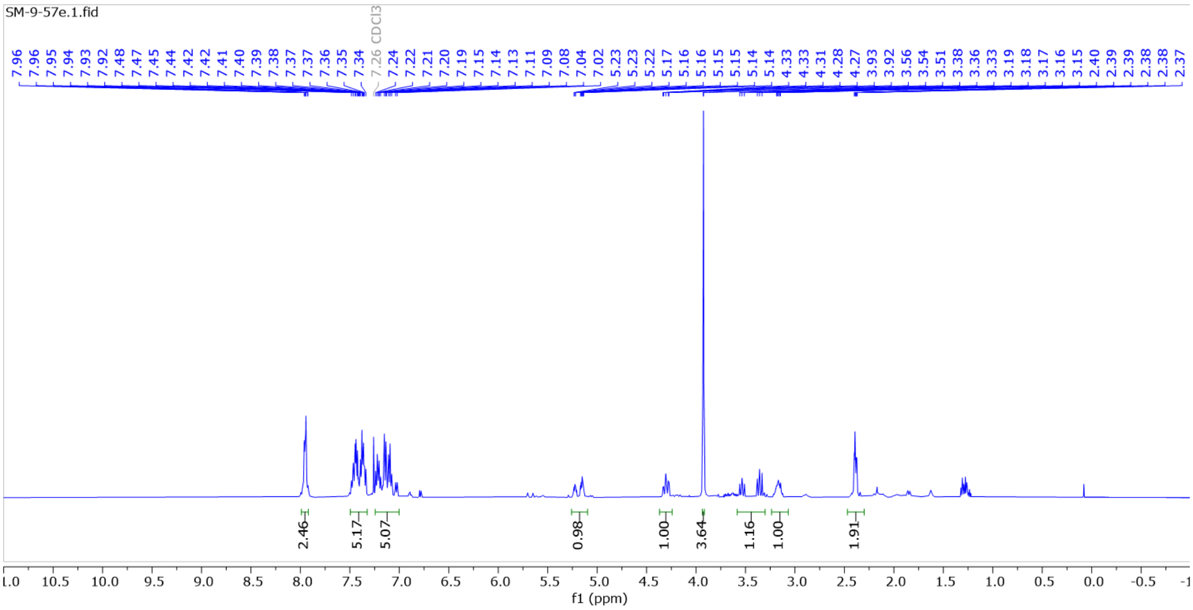

^{1}H NMR (500 MHZ, $CDCl_3$) of **3x**

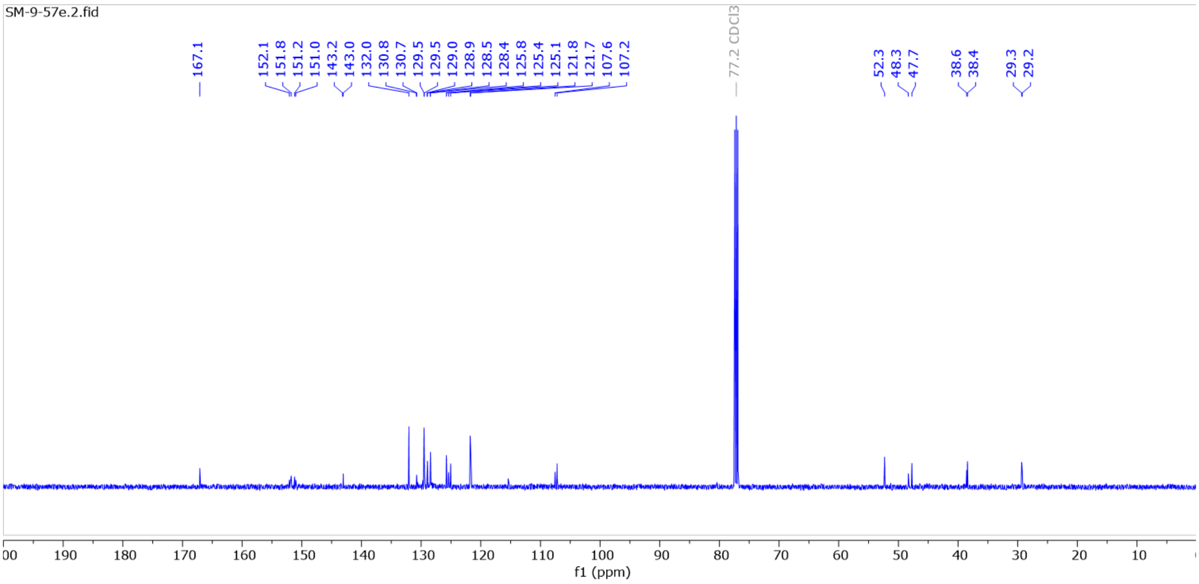

^{13}C NMR (126 MHZ, $CDCl_3$) of **3x**

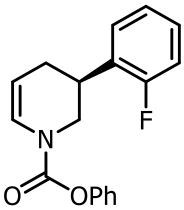

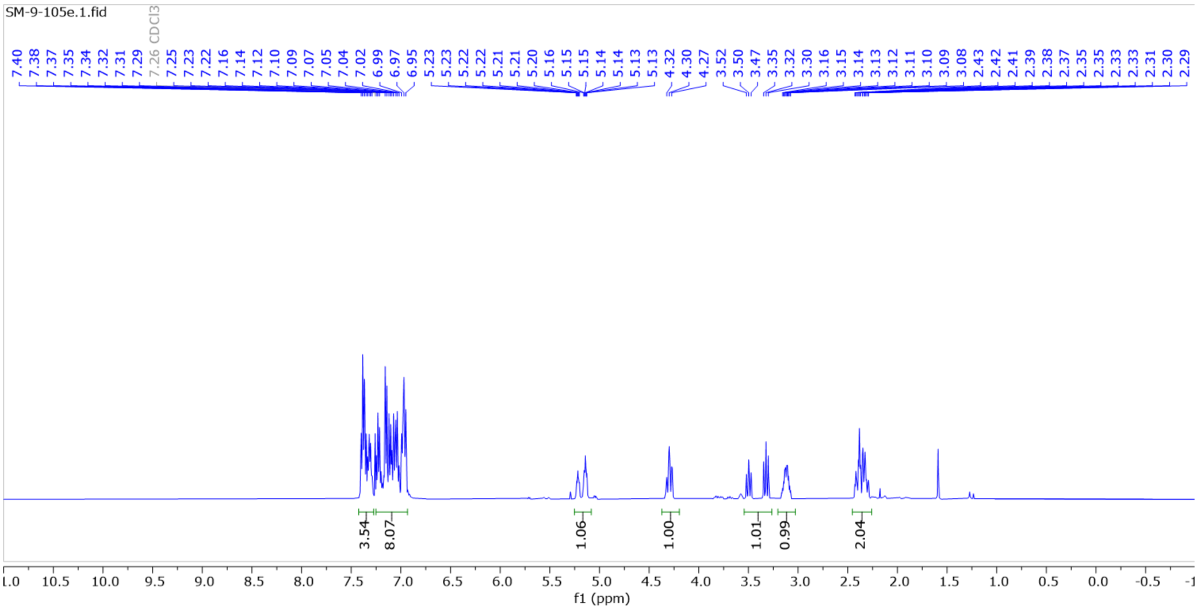

^{1}H NMR (500 MHZ, $CDCl_3$) of **3y**

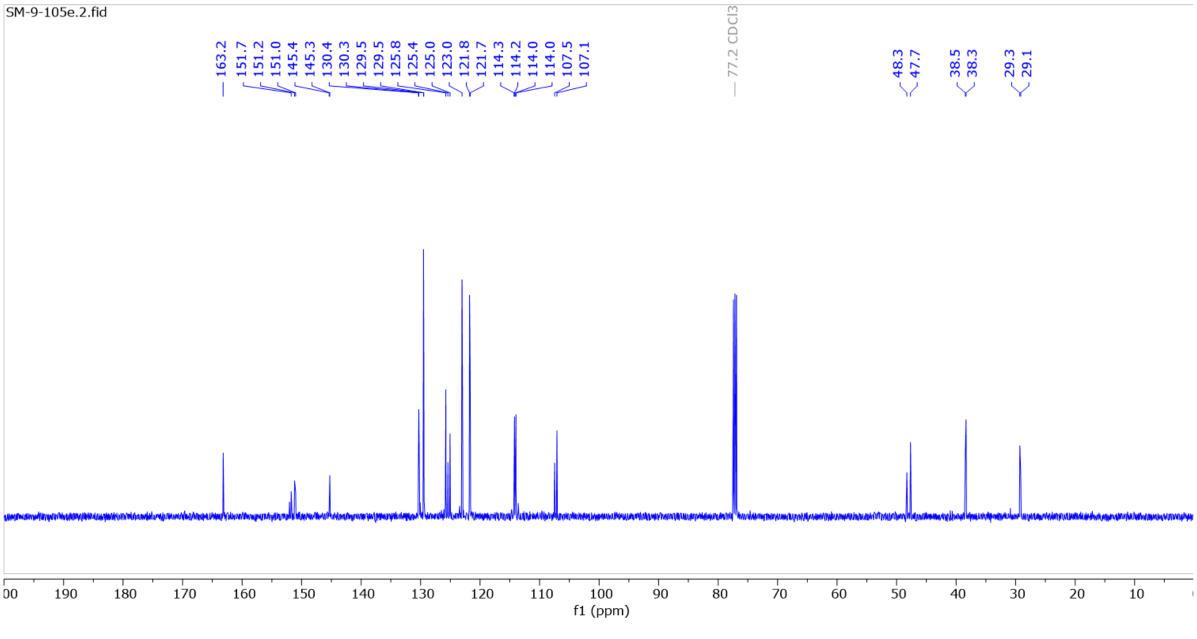

^{13}C NMR (101 MHZ, $CDCl_3$) of **3y**

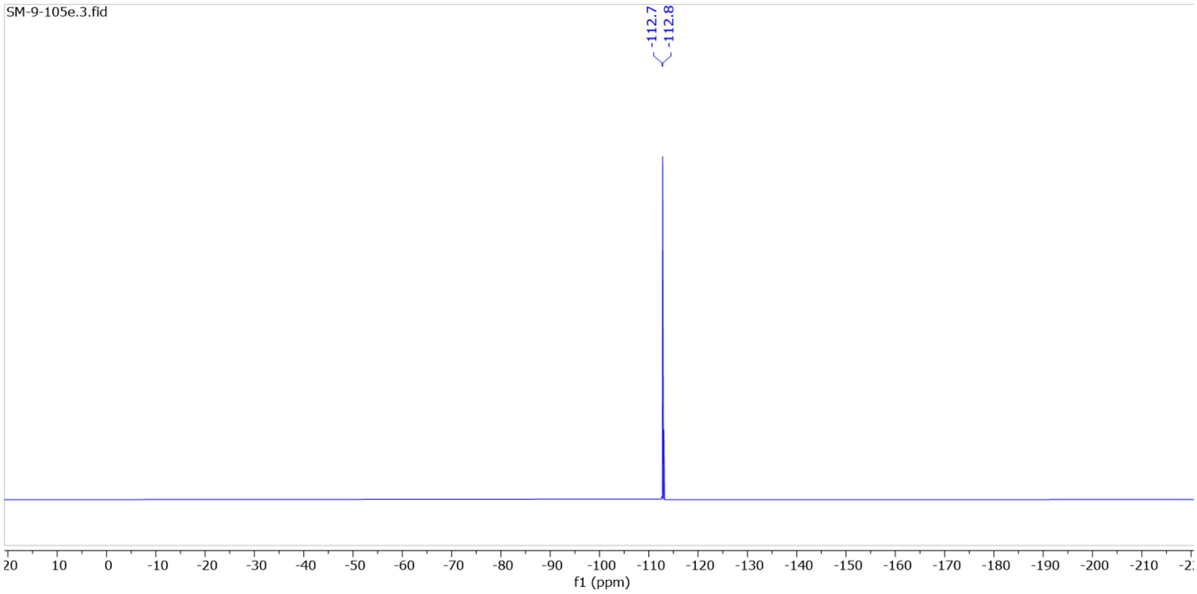

^{19}F NMR (471 MHZ, $CDCl_3$) of **3y**

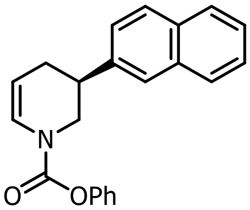

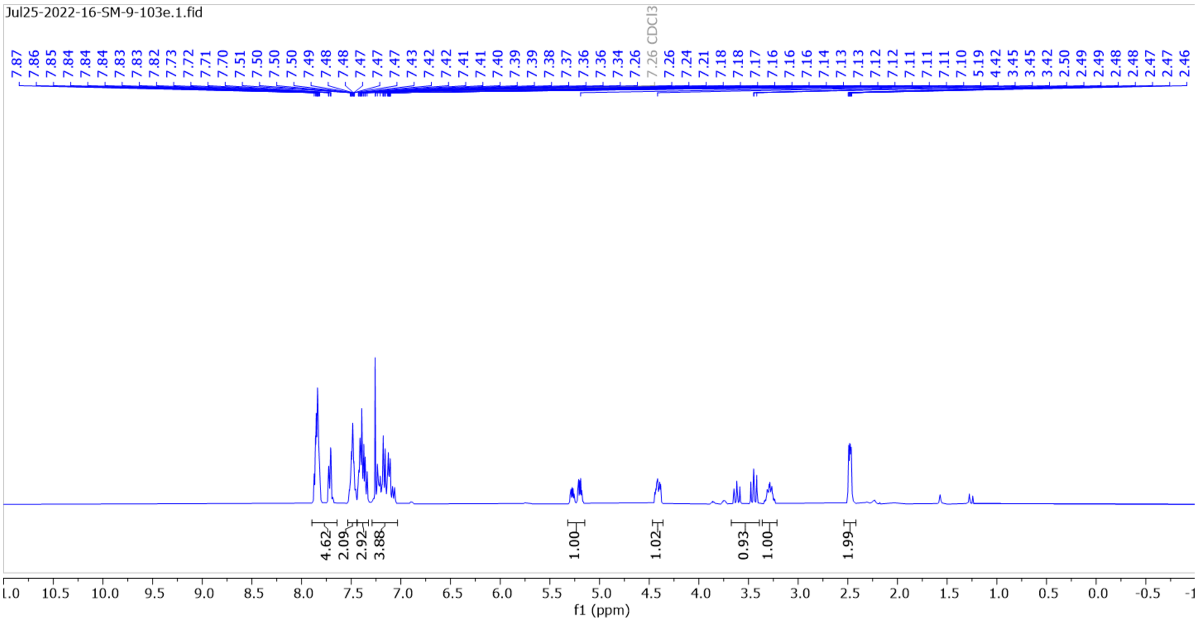

^{1}H NMR (400 MHZ, $CDCl_3$) of **3z**

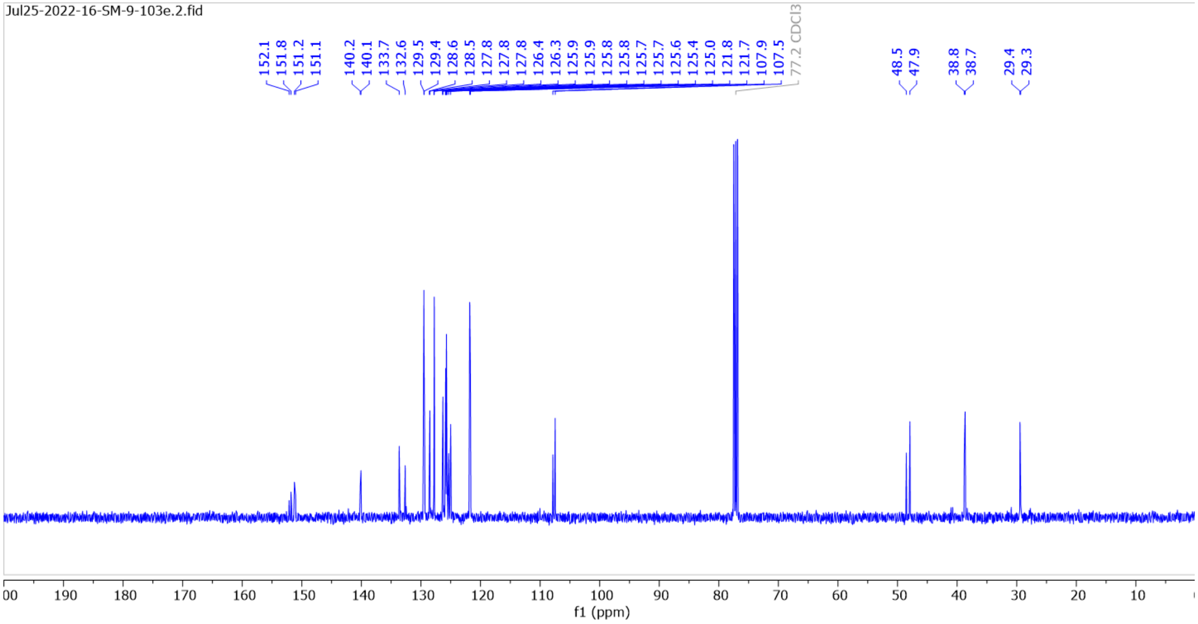

^{13}C NMR (101 MHZ, $CDCl_3$) of **3z**

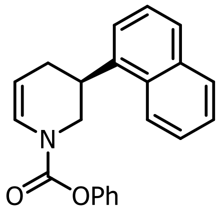

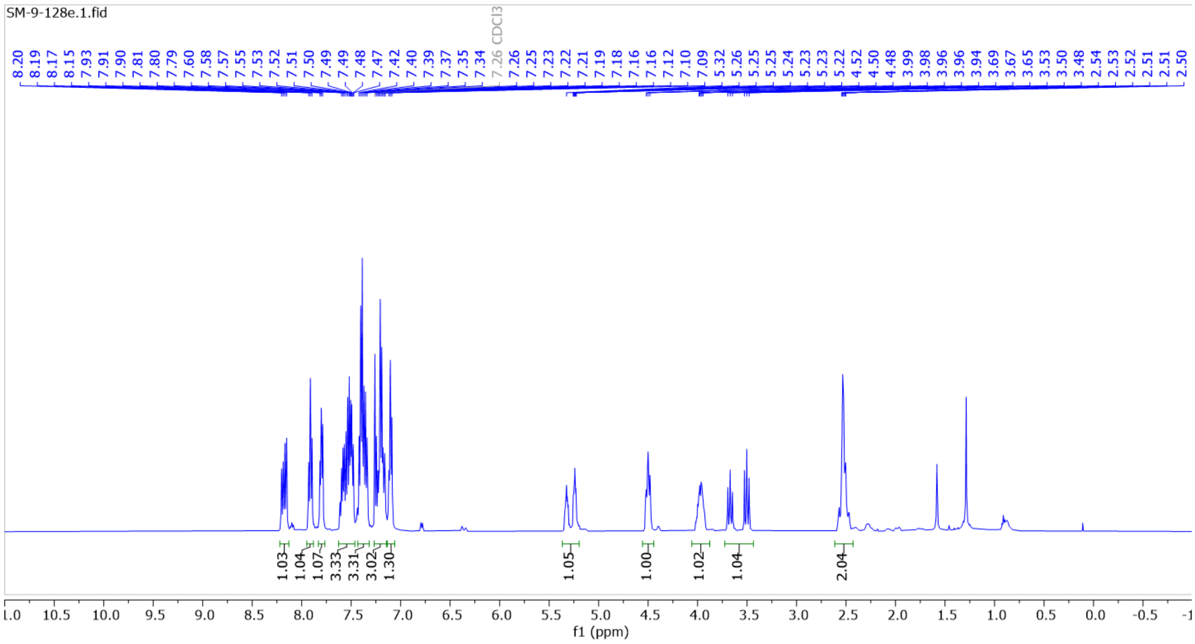

^{1}H NMR (500 MHZ, $CDCl_3$) of **3aa**

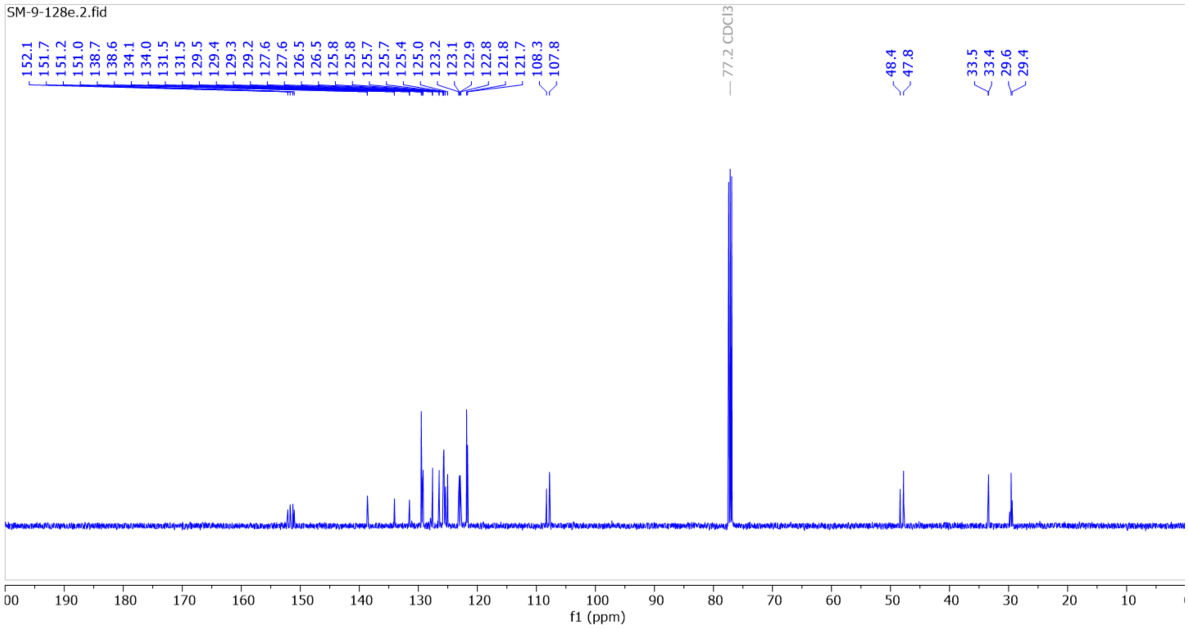

^{13}C NMR (126 MHZ, $CDCl_3$) of **3aa**

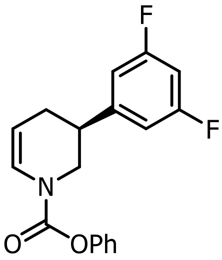

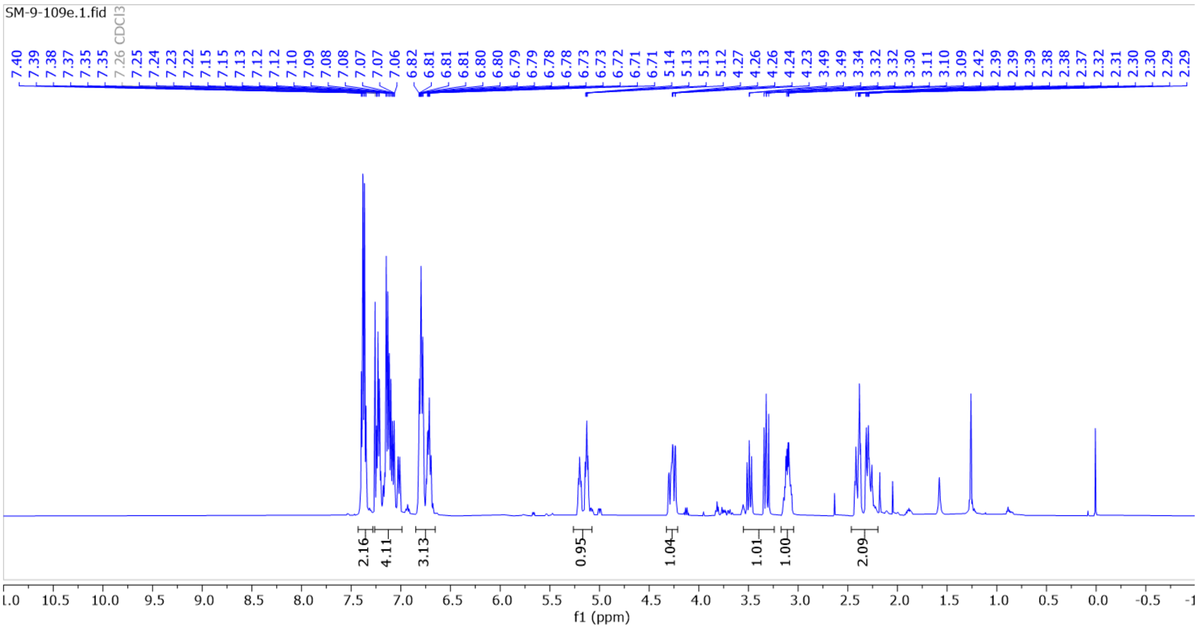

^{1}H NMR (500 MHZ, $CDCl_3$) of **3ab**

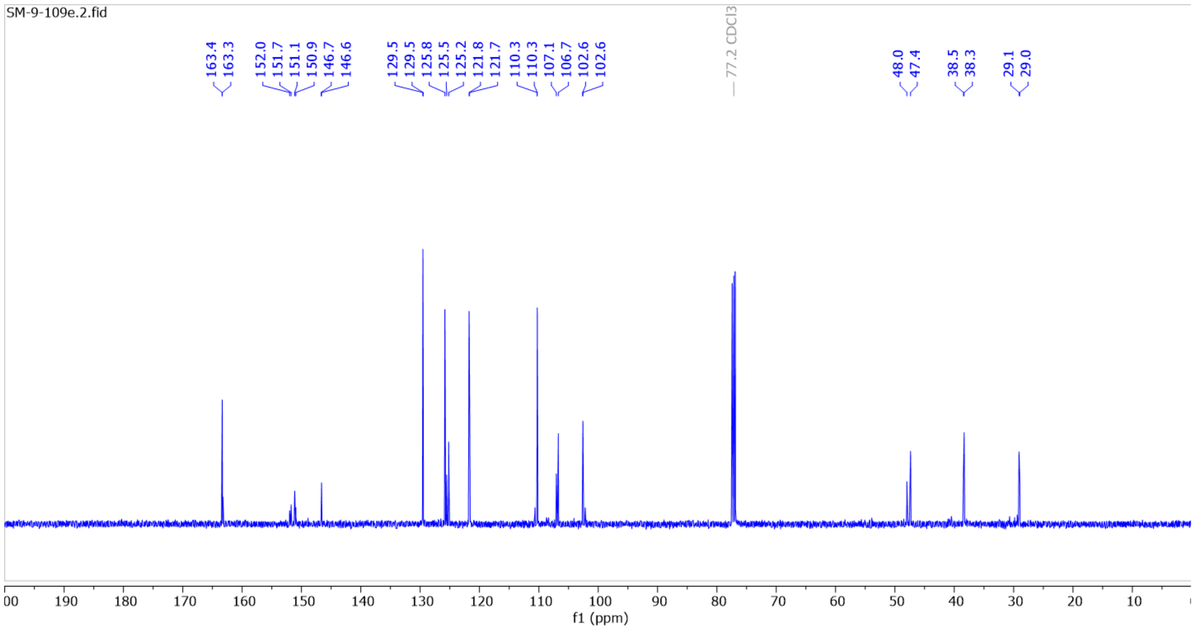

^{13}C NMR (126 MHZ, $CDCl_3$) of **3ab**

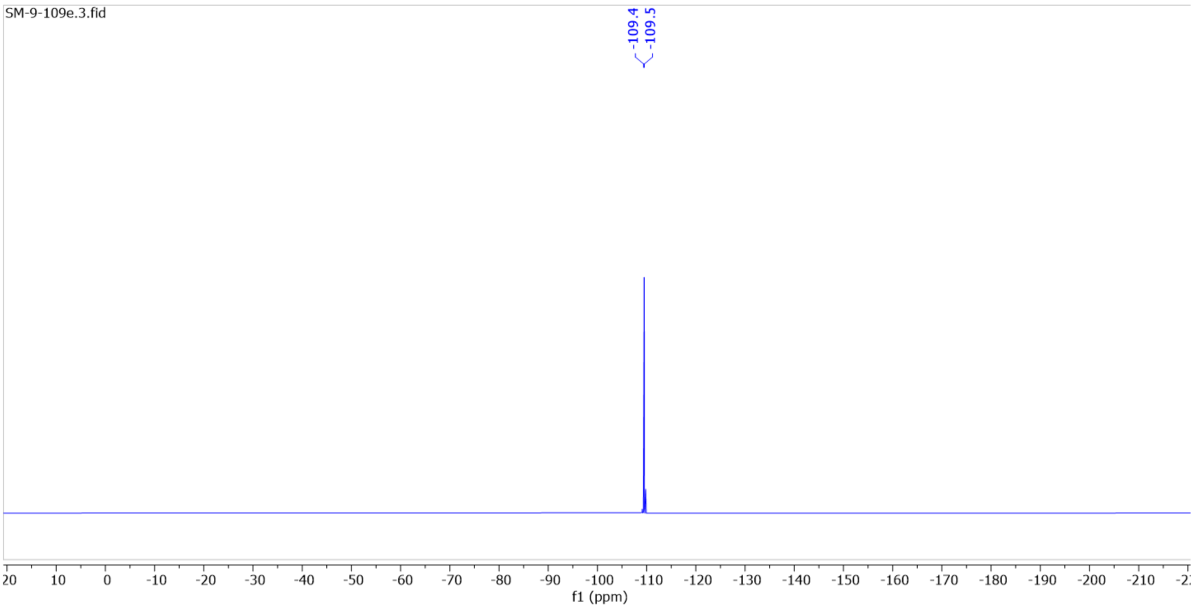

^{19}F NMR (471 MHZ, $CDCl_3$) of **3ab**

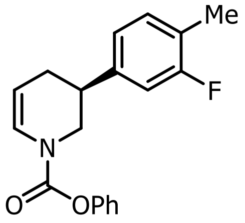

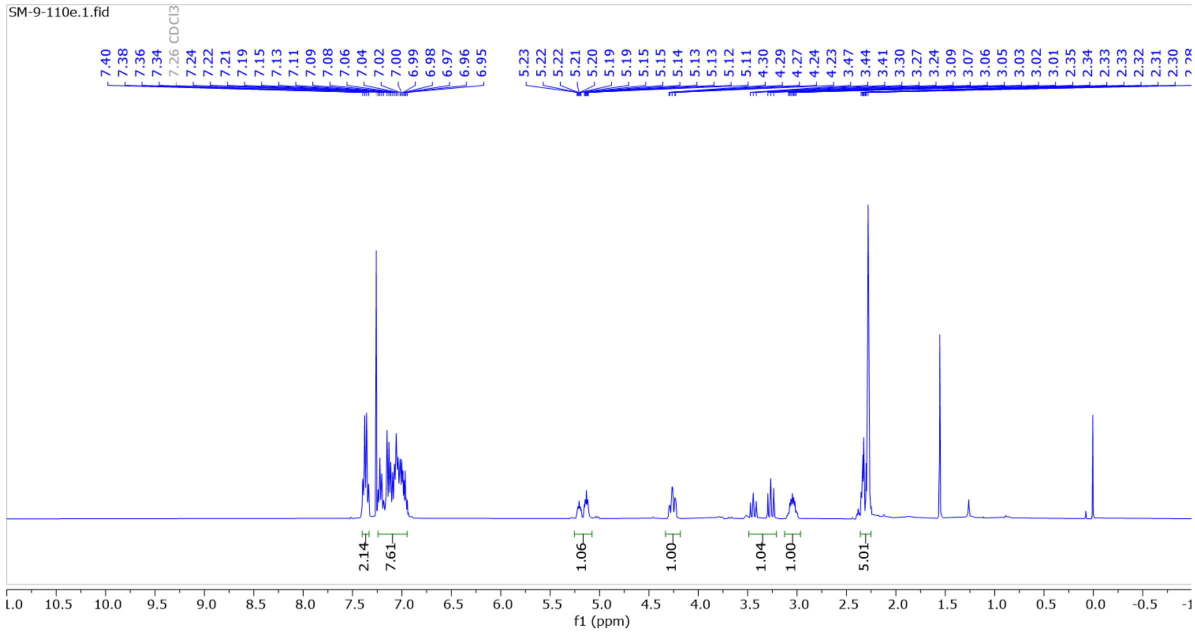

^{1}H NMR (400 MHZ, $CDCl_3$) of **3ac**

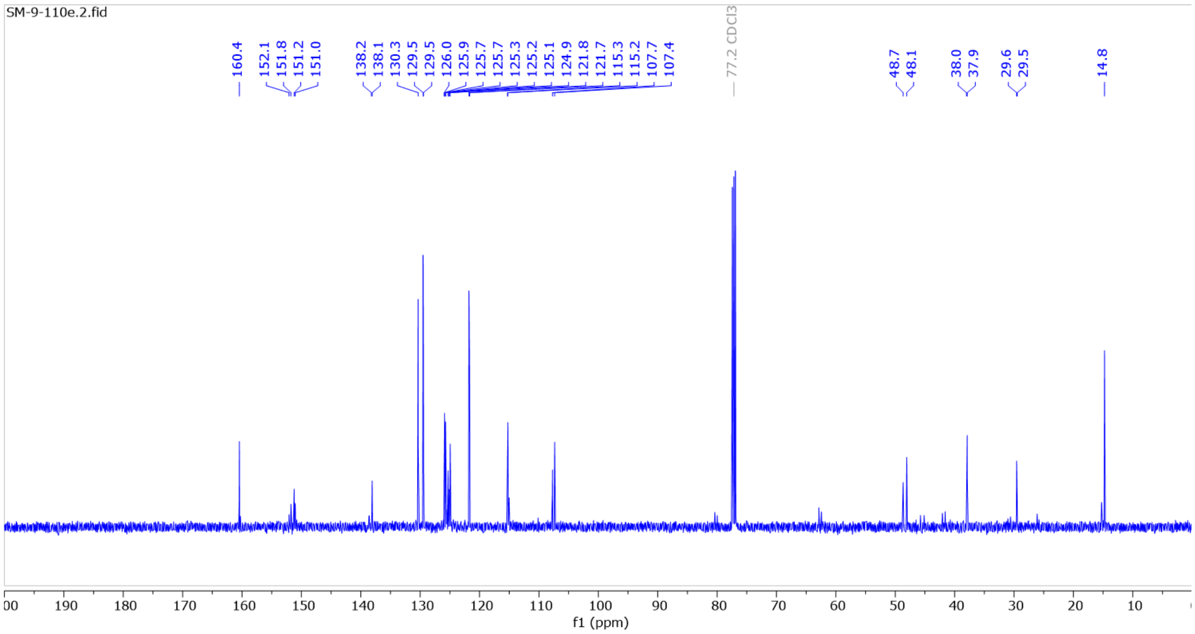

^{13}C NMR (126 MHZ, $CDCl_3$) of **3ac**

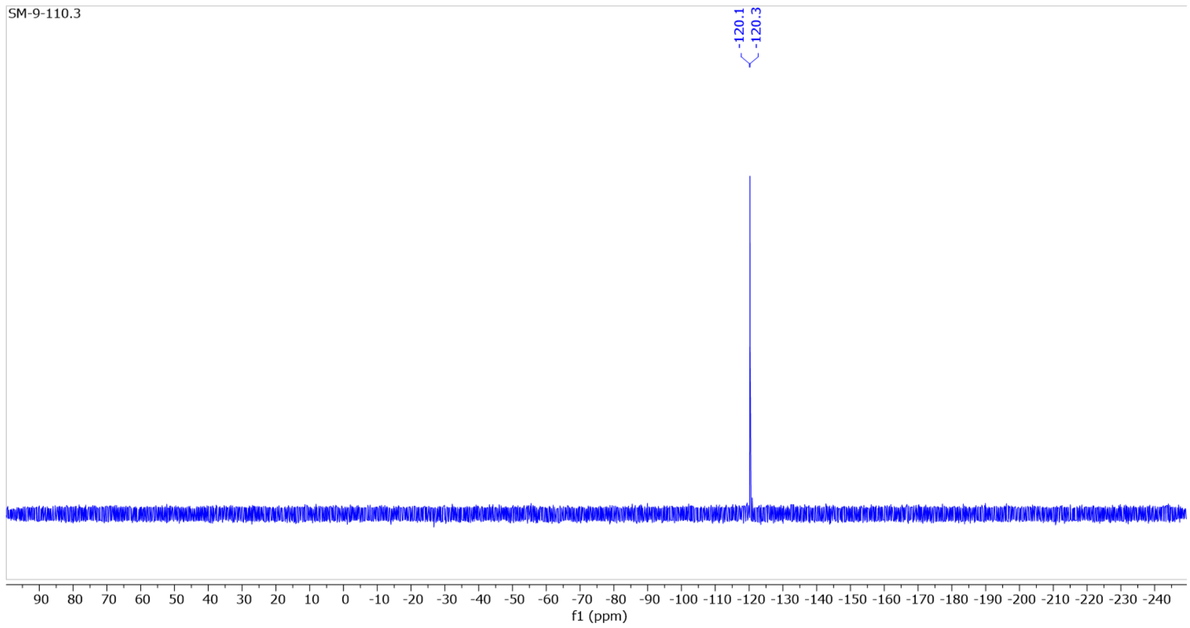

^{19}F NMR (471 MHZ, $CDCl_3$) of **3ac**

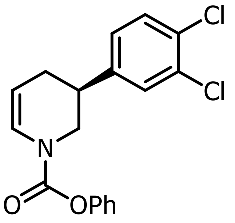

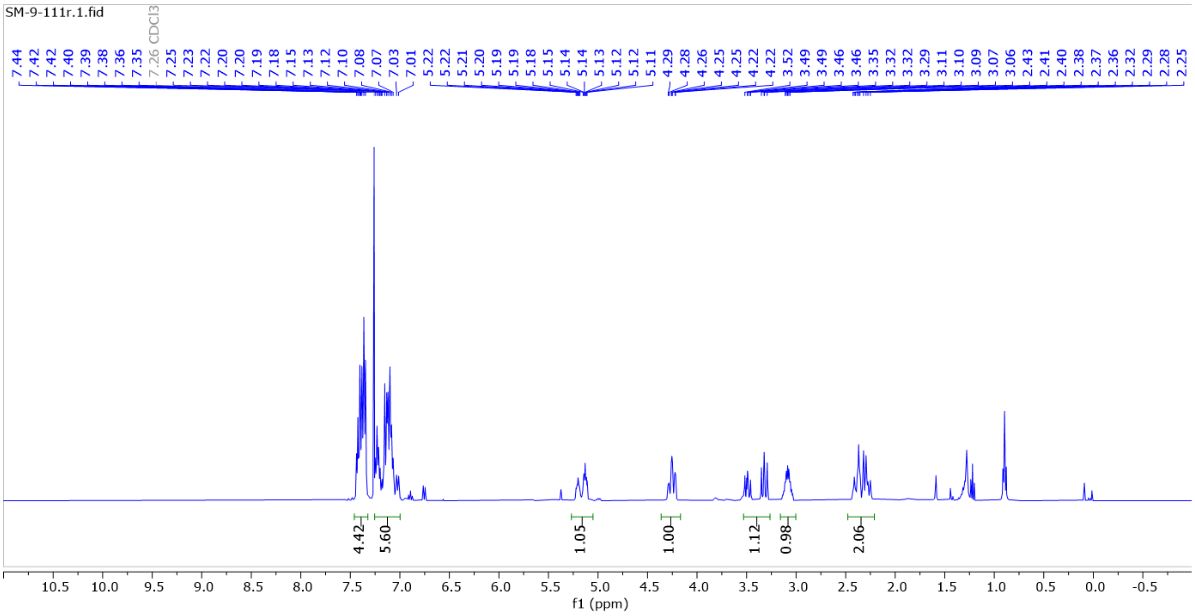

^{1}H NMR (400 MHZ, $CDCl_3$) of **3ad**

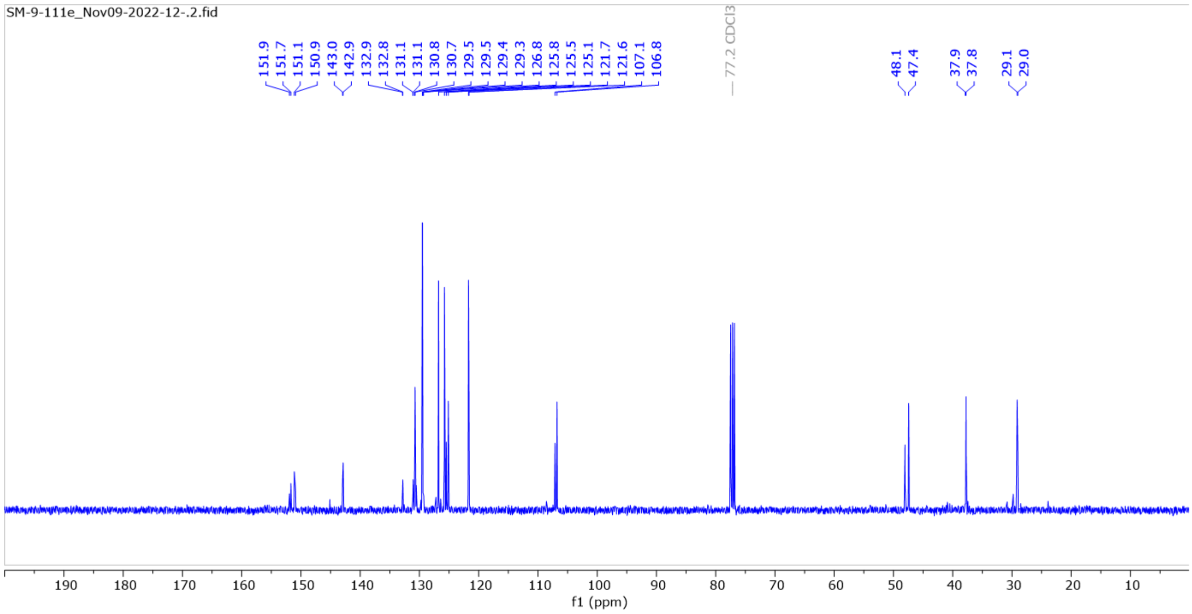

^{13}C NMR (101 MHZ, $CDCl_3$) of **3ad**

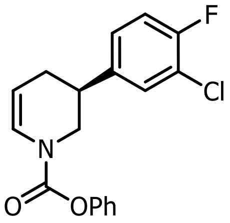

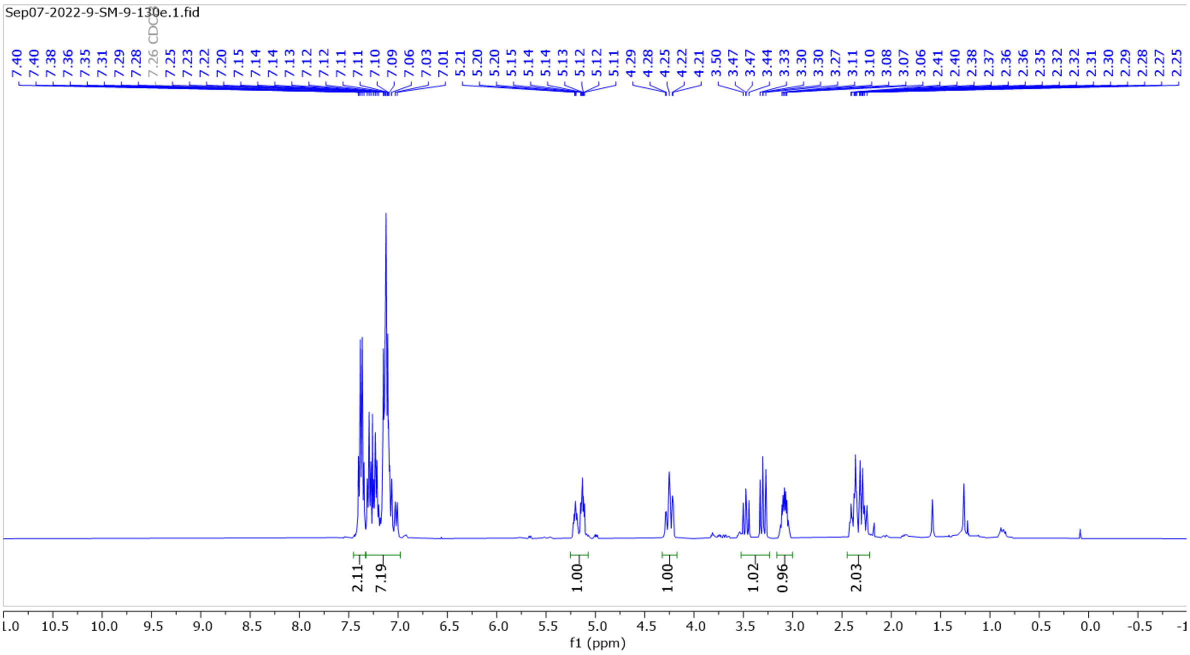

^{1}H NMR (400 MHZ, $CDCl_3$) of **3ae**

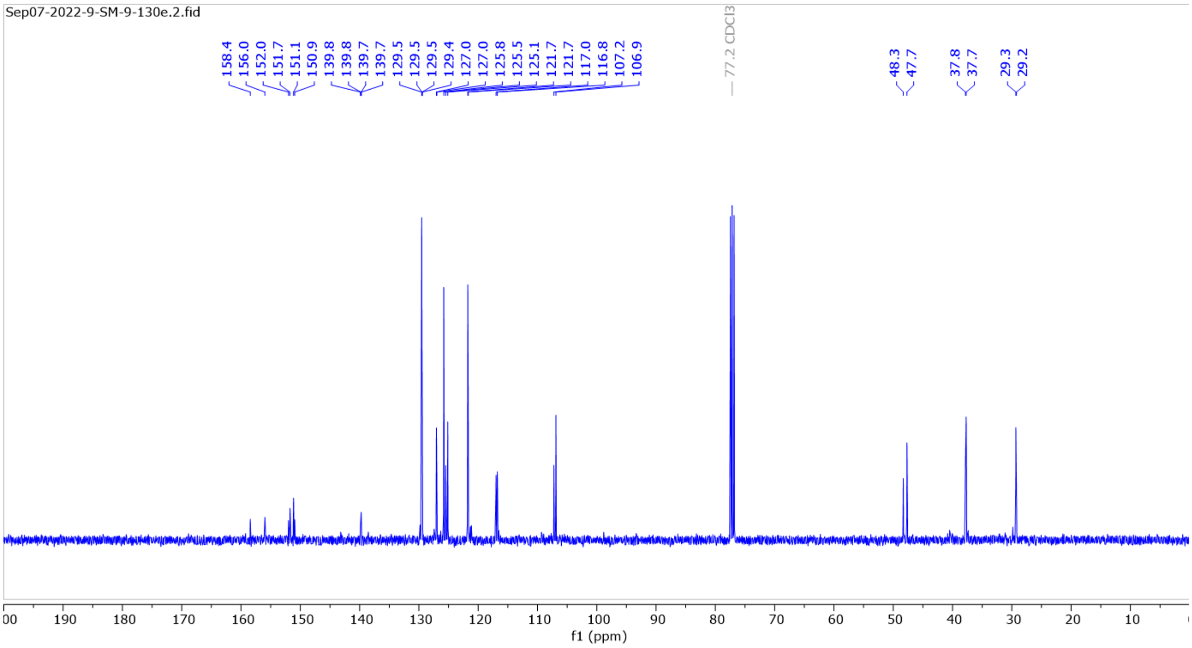

^{13}C NMR (101 MHZ, $CDCl_3$) of **3ae**

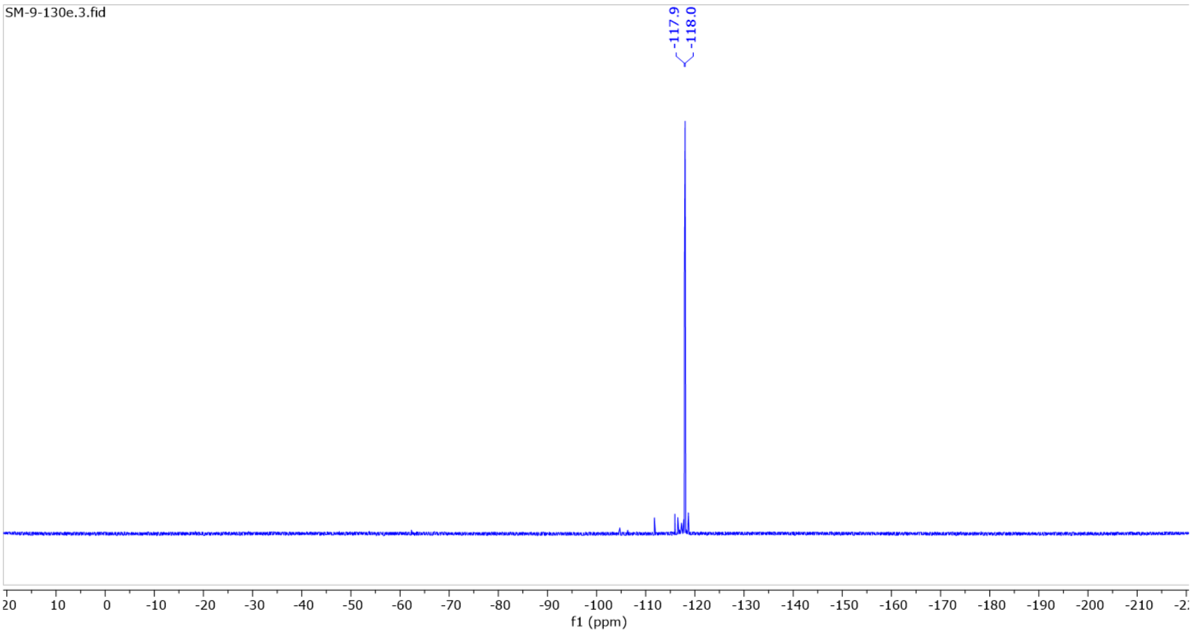

^{19}F NMR (471 MHZ, $CDCl_3$) of **3ae**

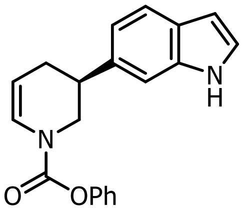

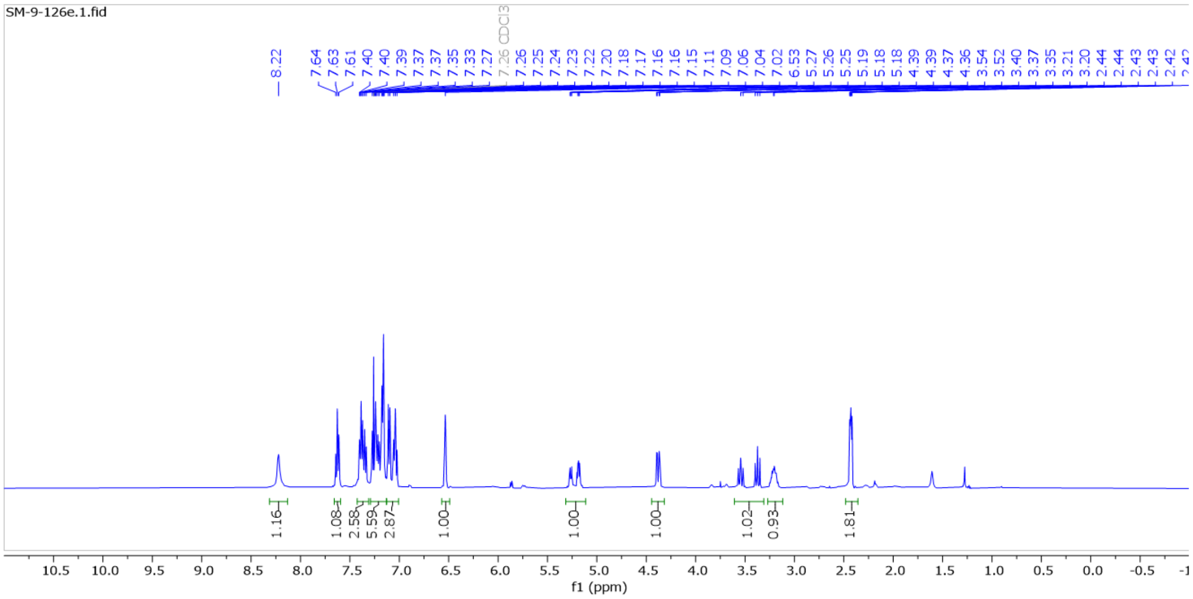

^{1}H NMR (500 MHZ, $CDCl_3$) of **3af**

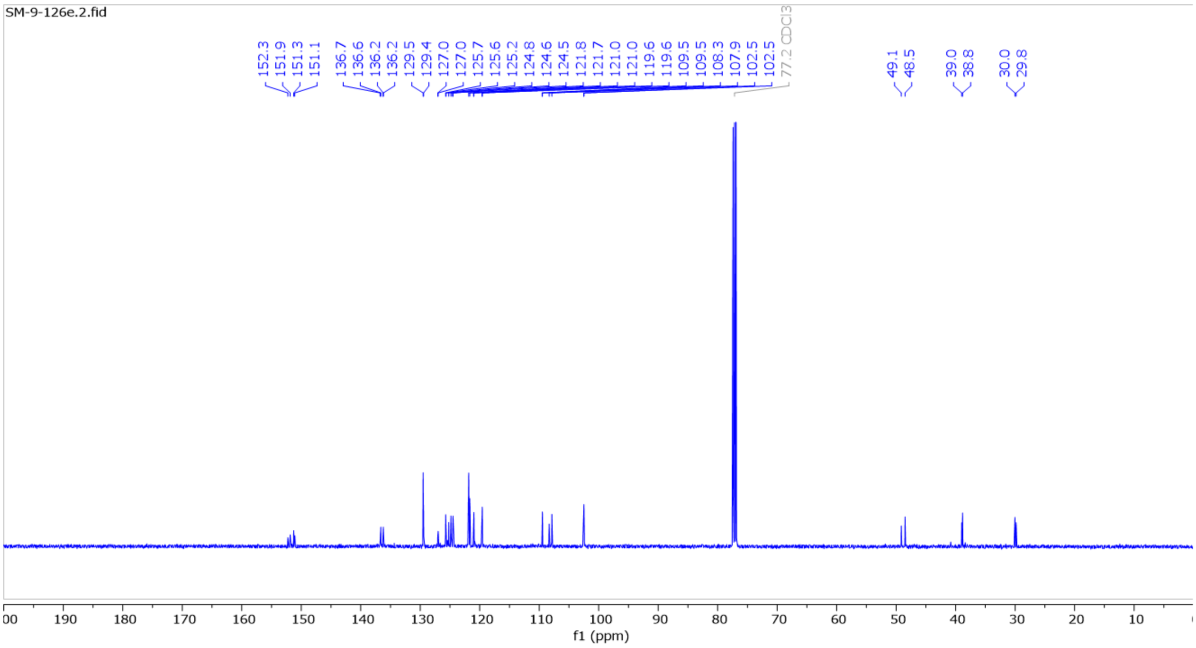

^{13}C NMR (126 MHZ, $CDCl_3$) of **3af**

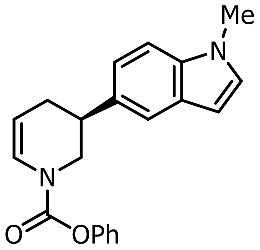

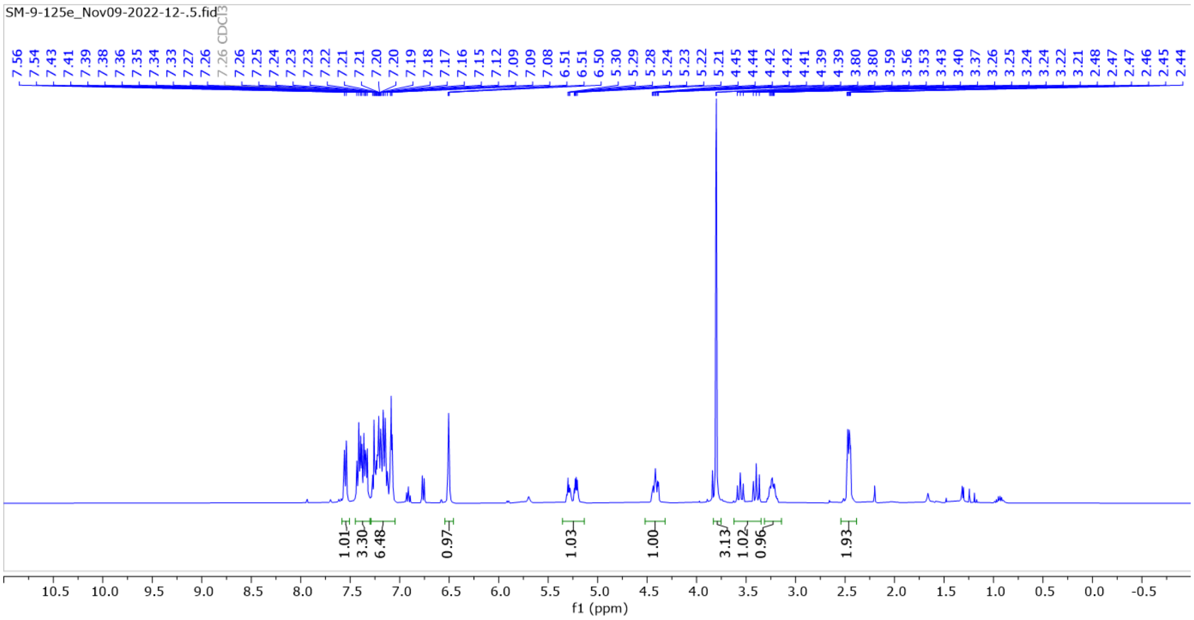

^{1}H NMR (400 MHZ, $CDCl_3$) of **3ag**

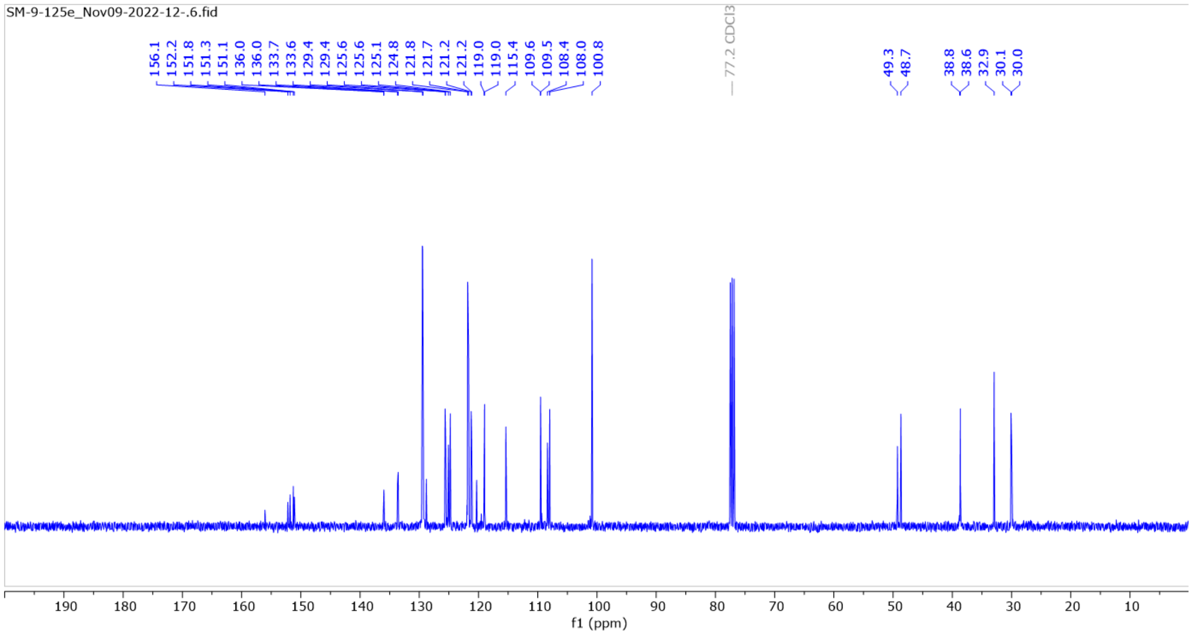

^{13}C NMR (101 MHZ, $CDCl_3$) of **3ag**

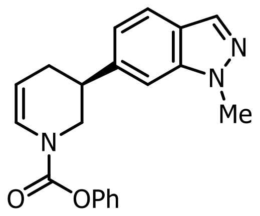

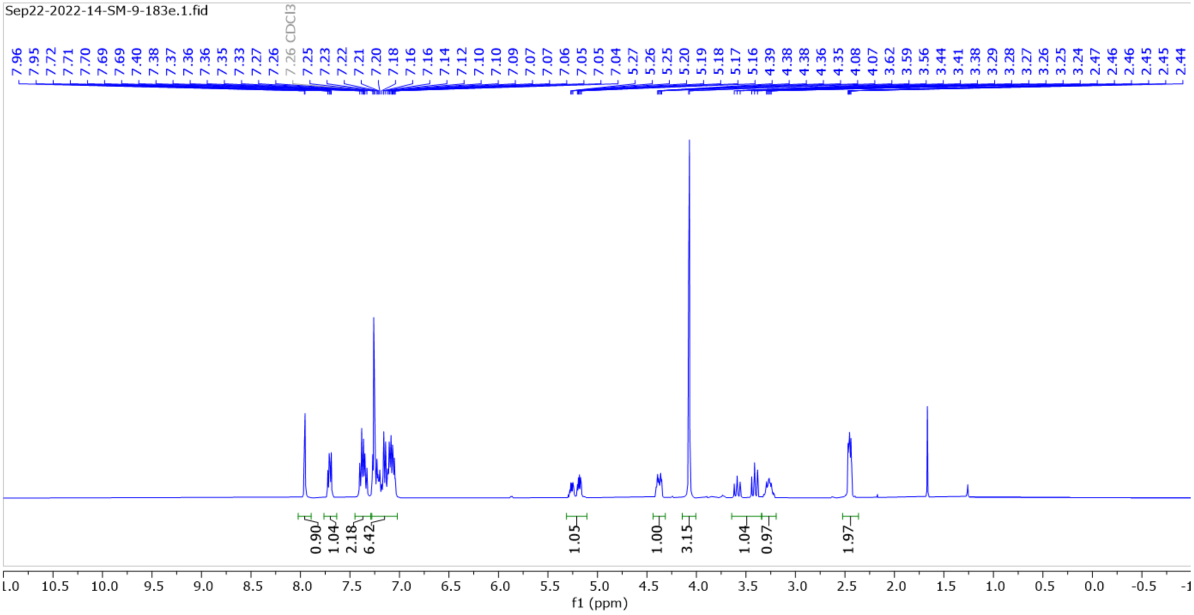

^{1}H NMR (400 MHZ, $CDCl_3$) of **3ah**

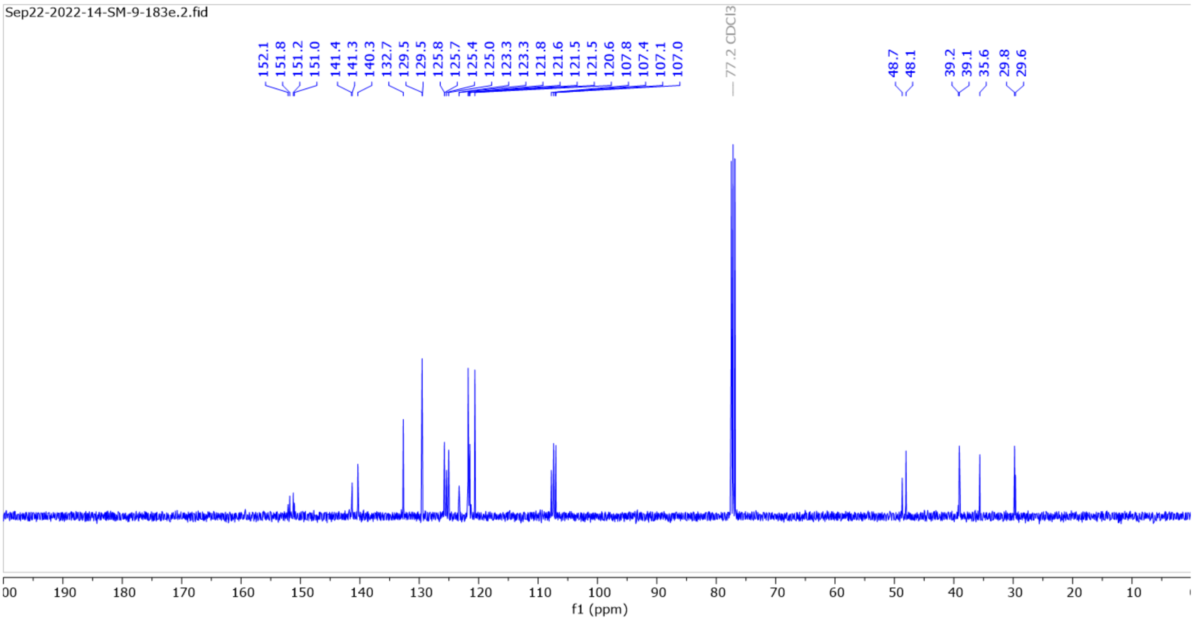

^{13}C NMR (101 MHZ, $CDCl_3$) of **3ah**

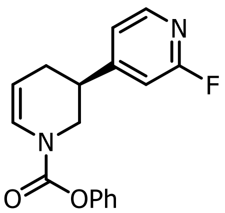

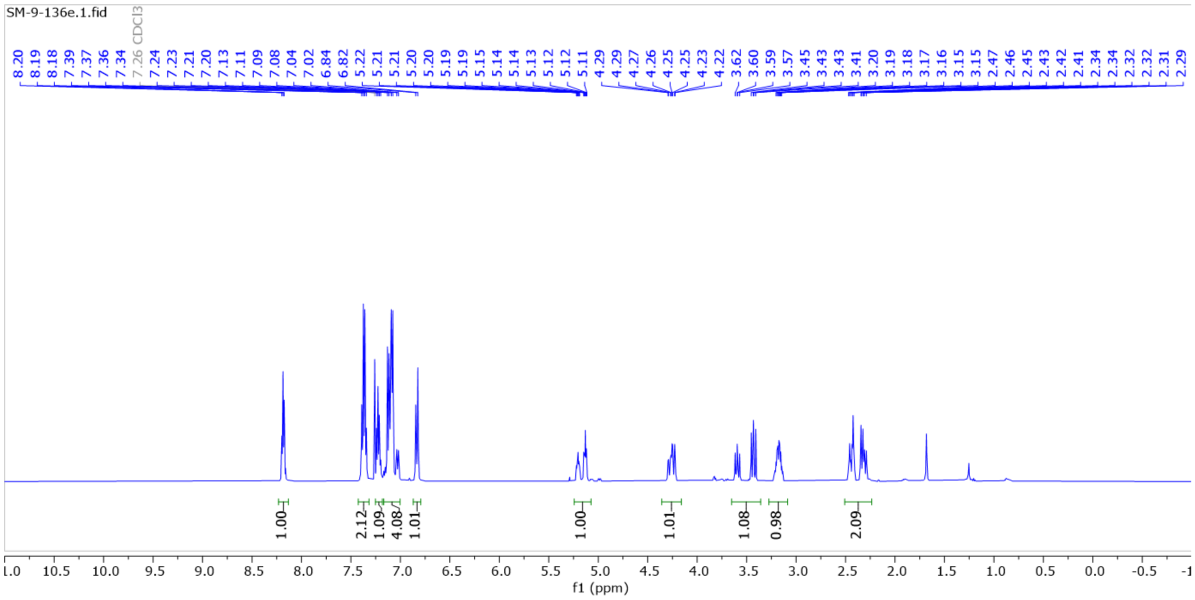

^{1}H NMR (500 MHZ, $CDCl_3$) of **3ai**

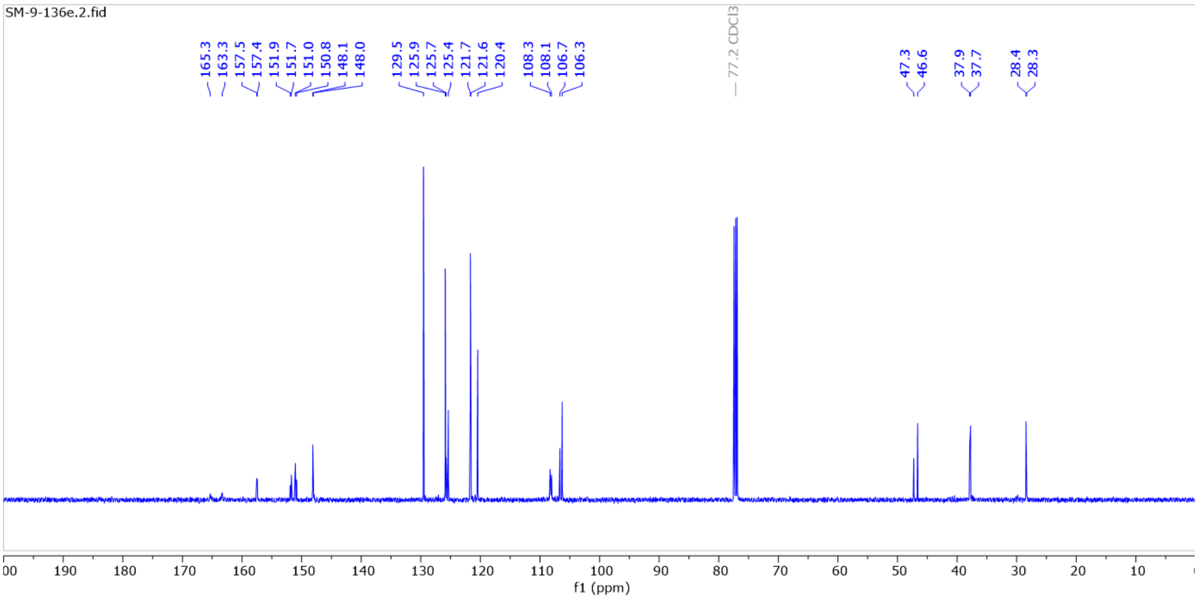

^{13}C NMR (126 MHZ, $CDCl_3$) of **3ai**

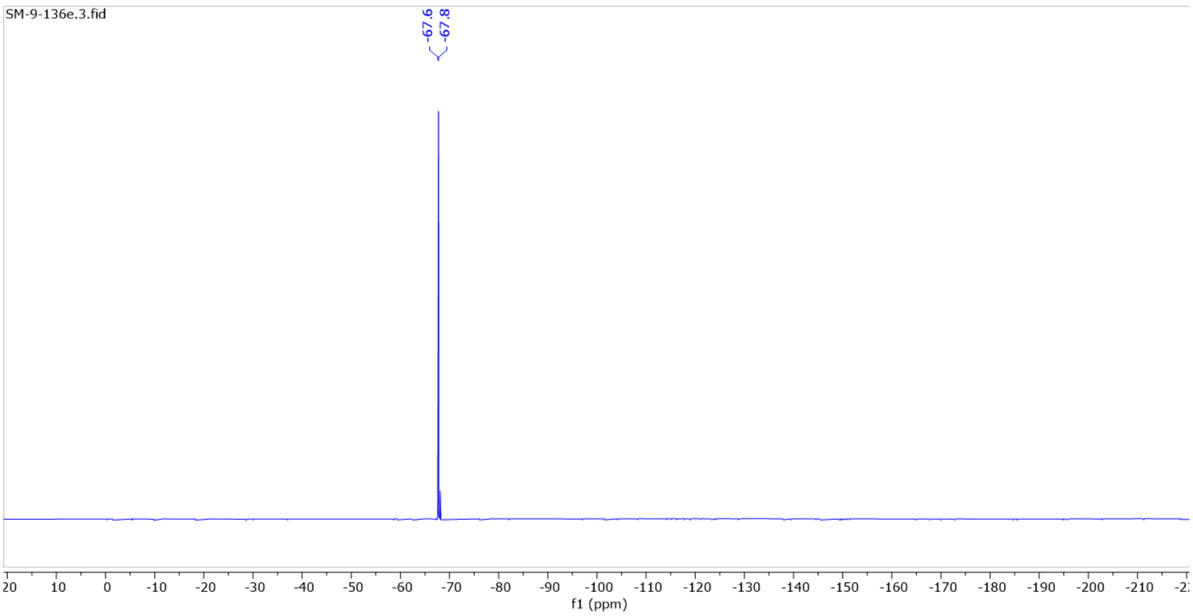

^{19}F NMR (471 MHZ, $CDCl_3$) of **3ai**

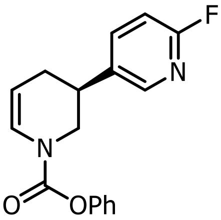

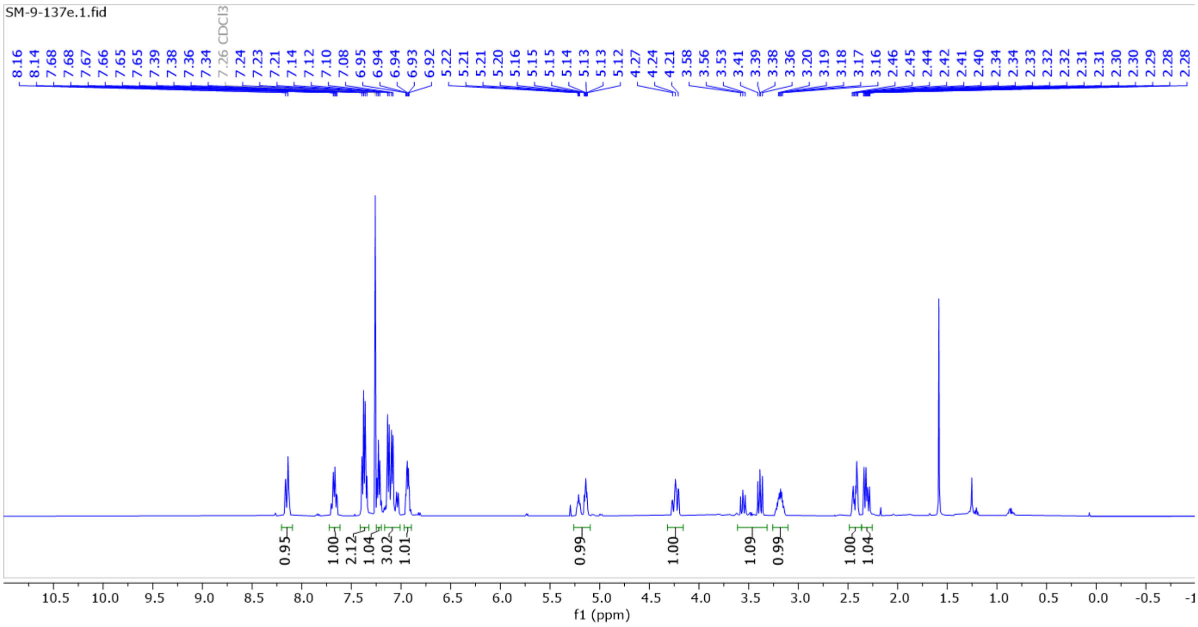

^{1}H NMR (500 MHZ, $CDCl_3$) of **3aj**

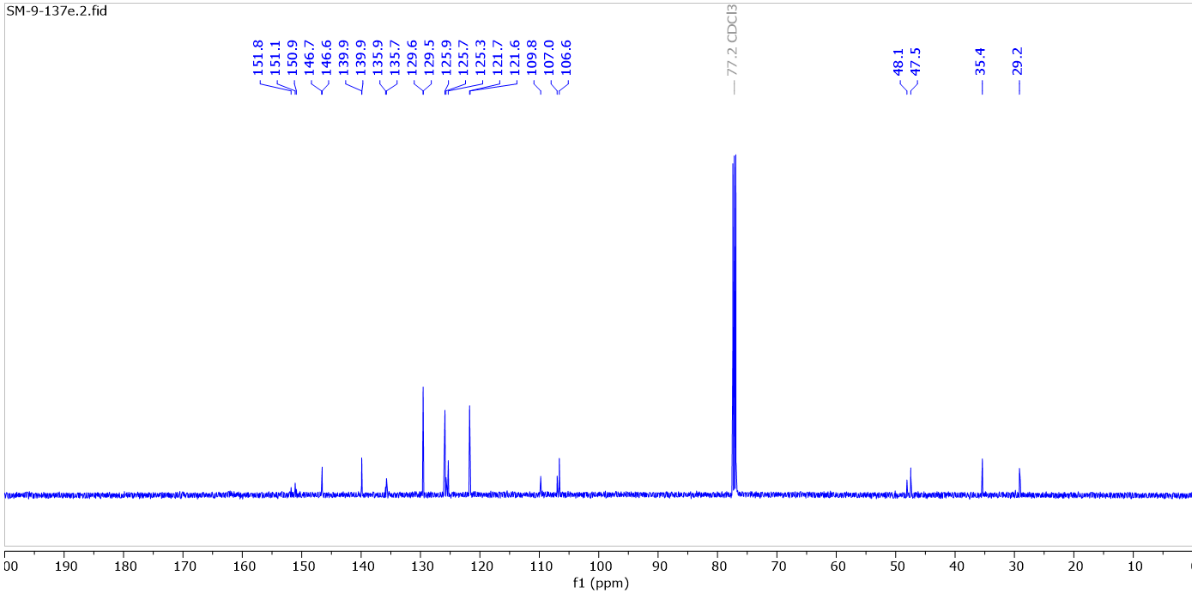

^{13}C NMR (126 MHZ, $CDCl_3$) of **3aj**

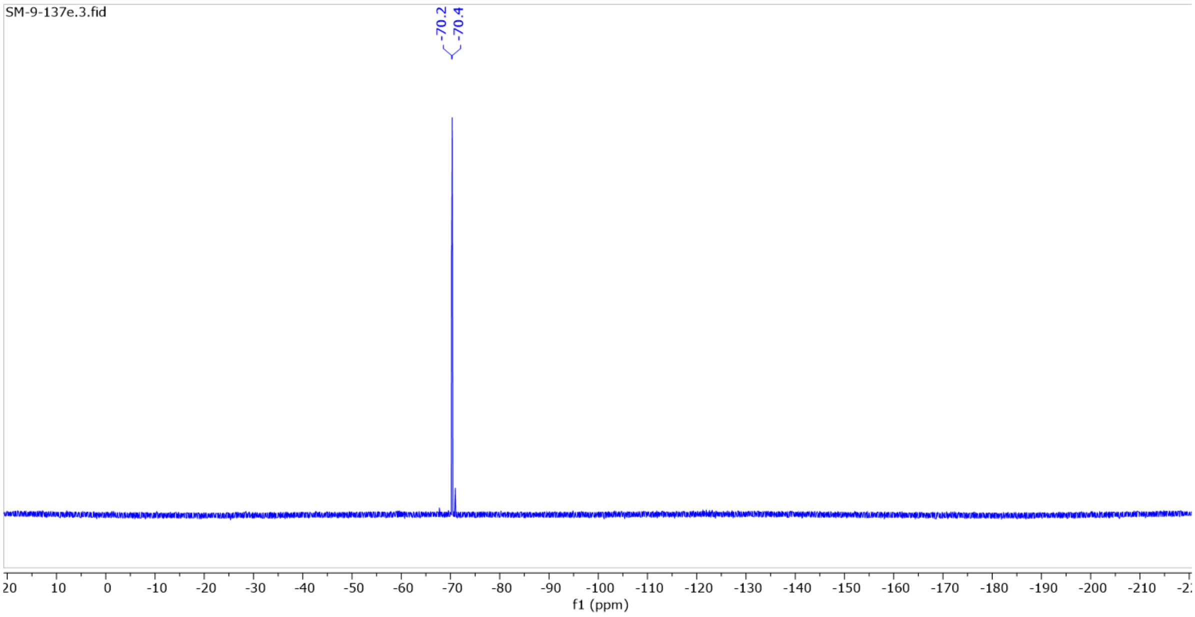

^{19}F NMR (471 MHZ, $CDCl_3$) of **3aj**

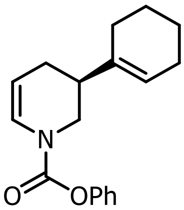

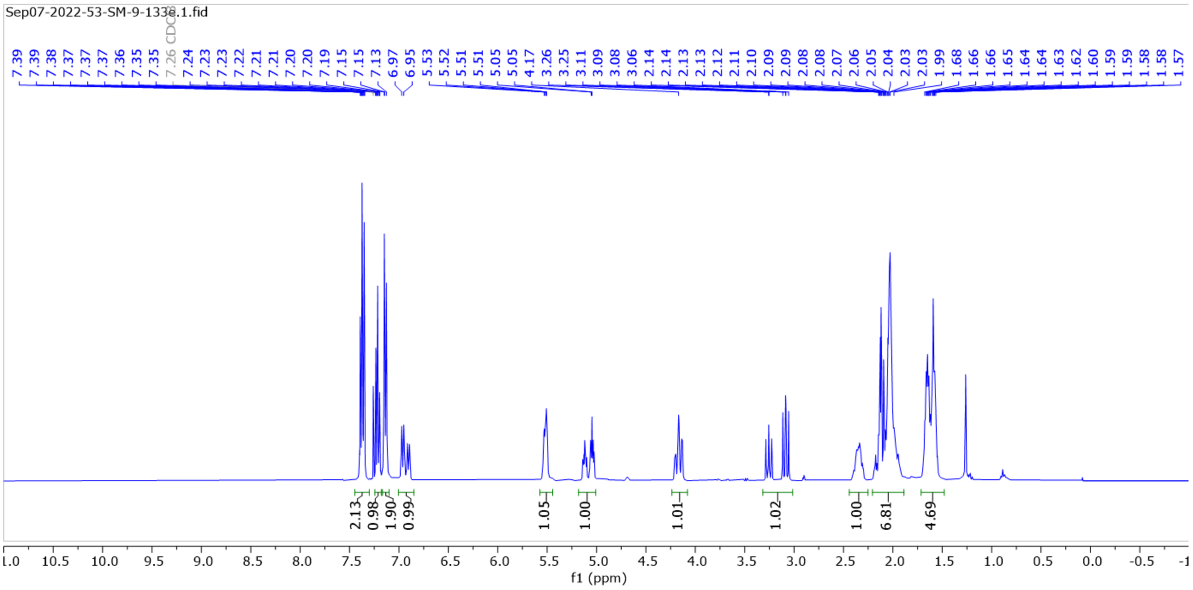

^{1}H NMR (400 MHZ, $CDCl_3$) of **3ak**

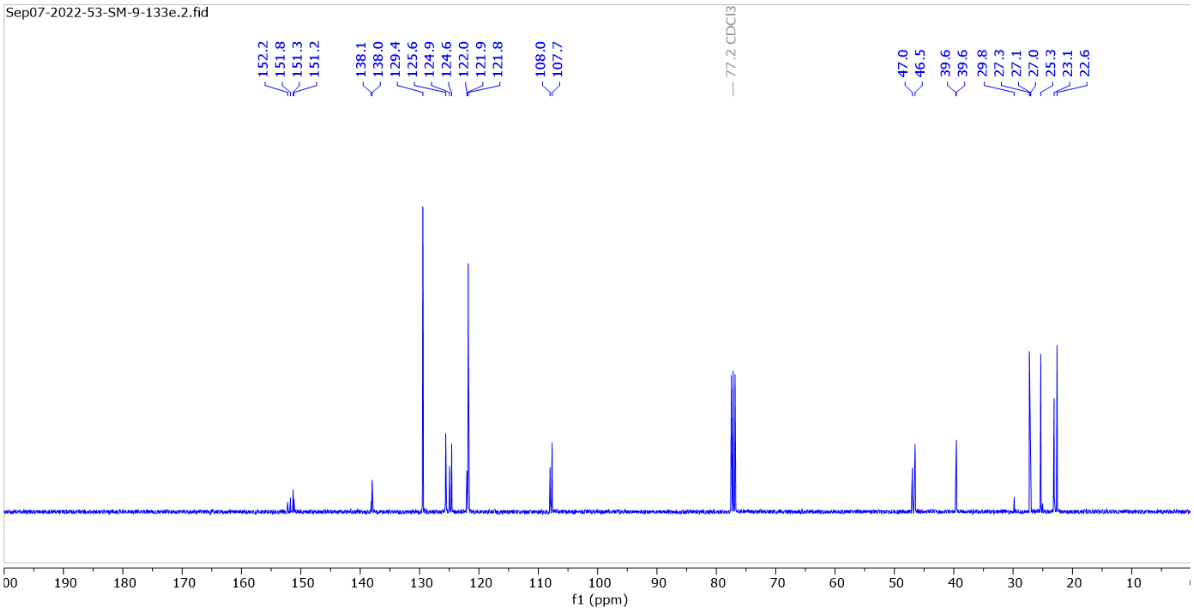

^{13}C NMR (101 MHZ, $CDCl_3$) of **3ak**

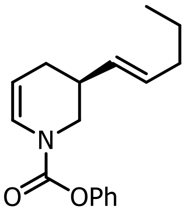

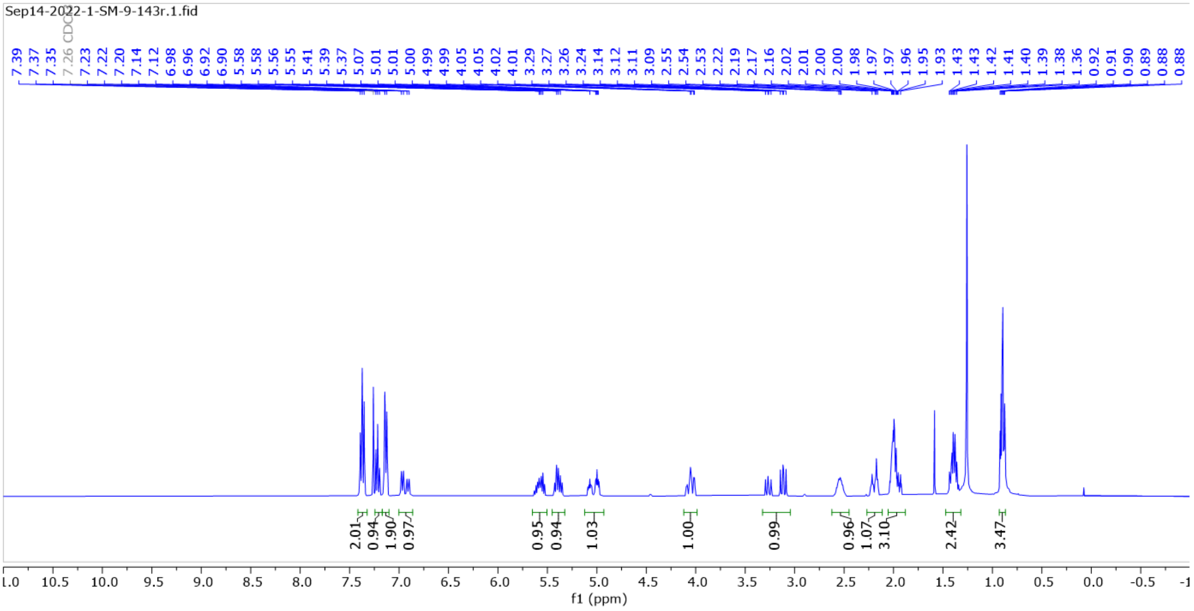

^{1}H NMR (400 MHZ, $CDCl_3$) of **3al**

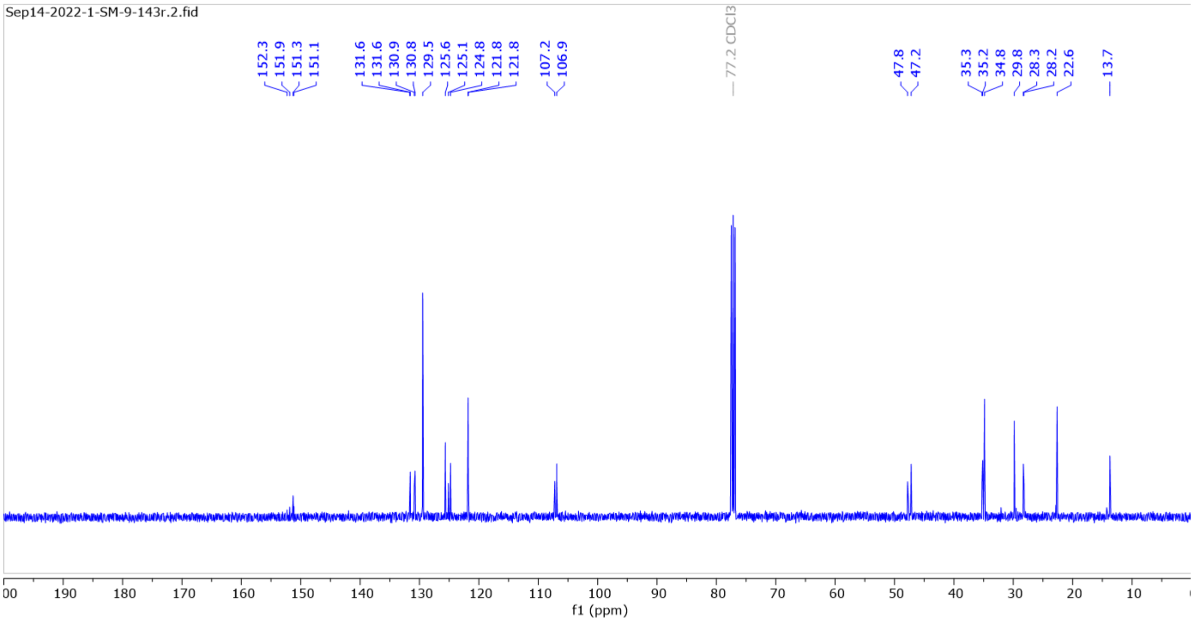

^{13}C NMR (101 MHZ, $CDCl_3$) of **3al**

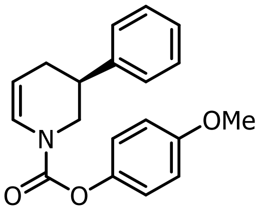

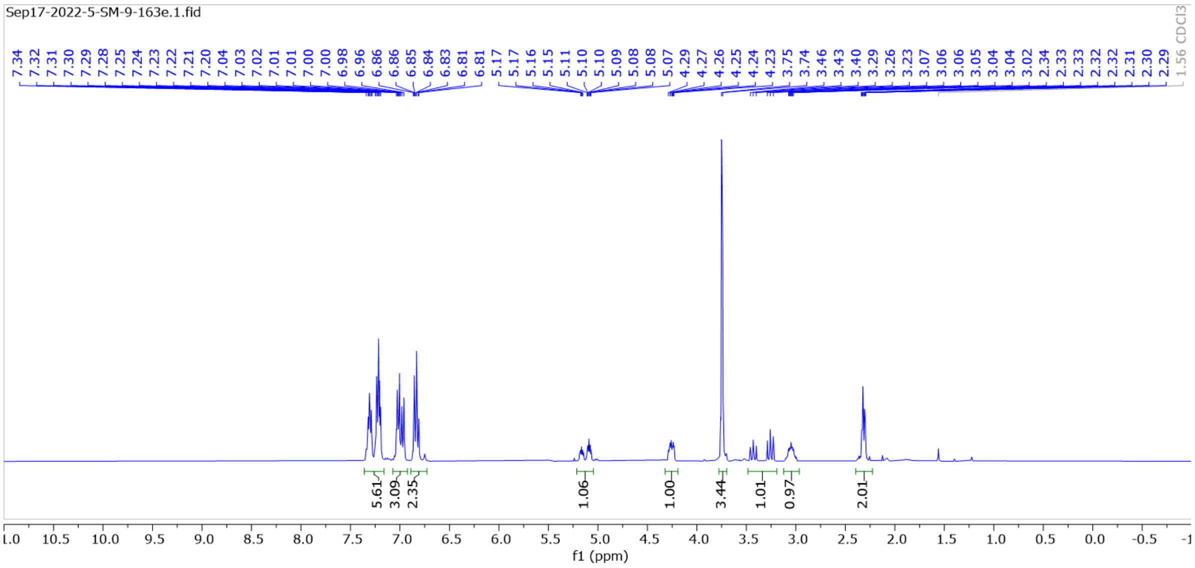

^{1}H NMR (400 MHZ, $CDCl_3$) of **3am**

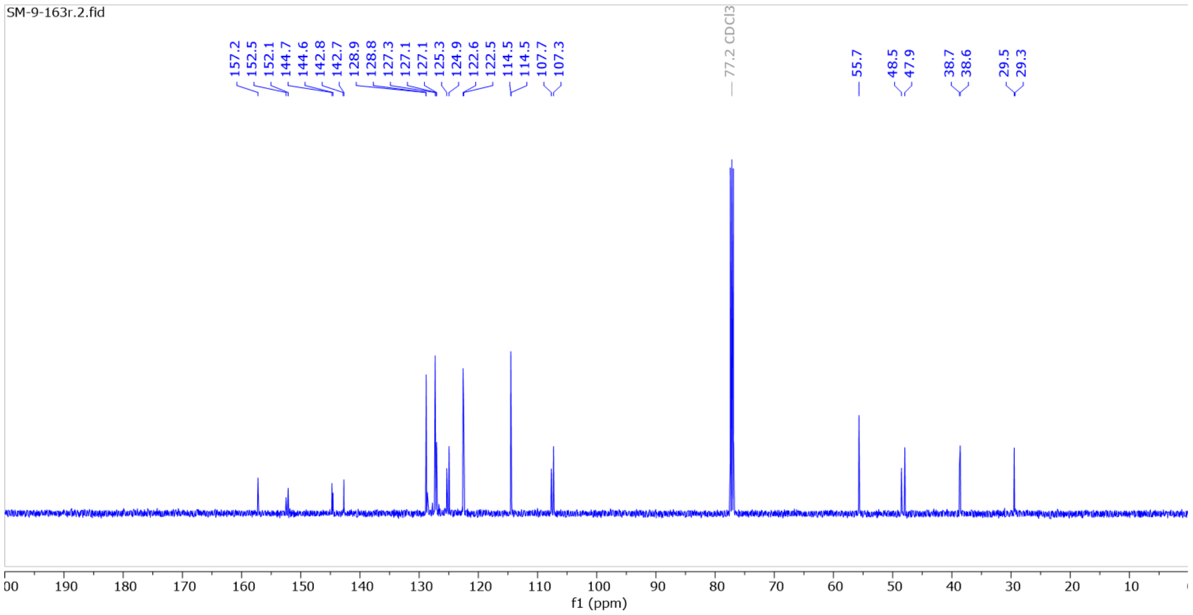

^{13}C NMR (126 MHZ, $CDCl_3$) of **3am**

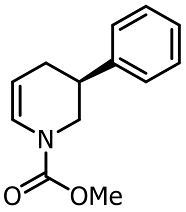

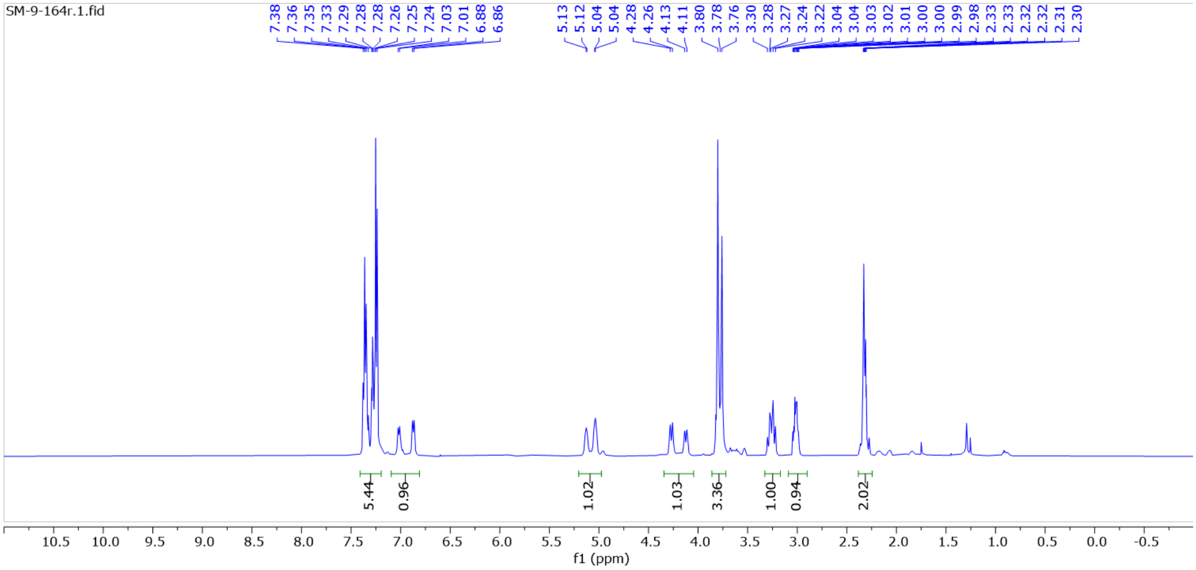

^{1}H NMR (500 MHZ, $CDCl_3$) of **3an**

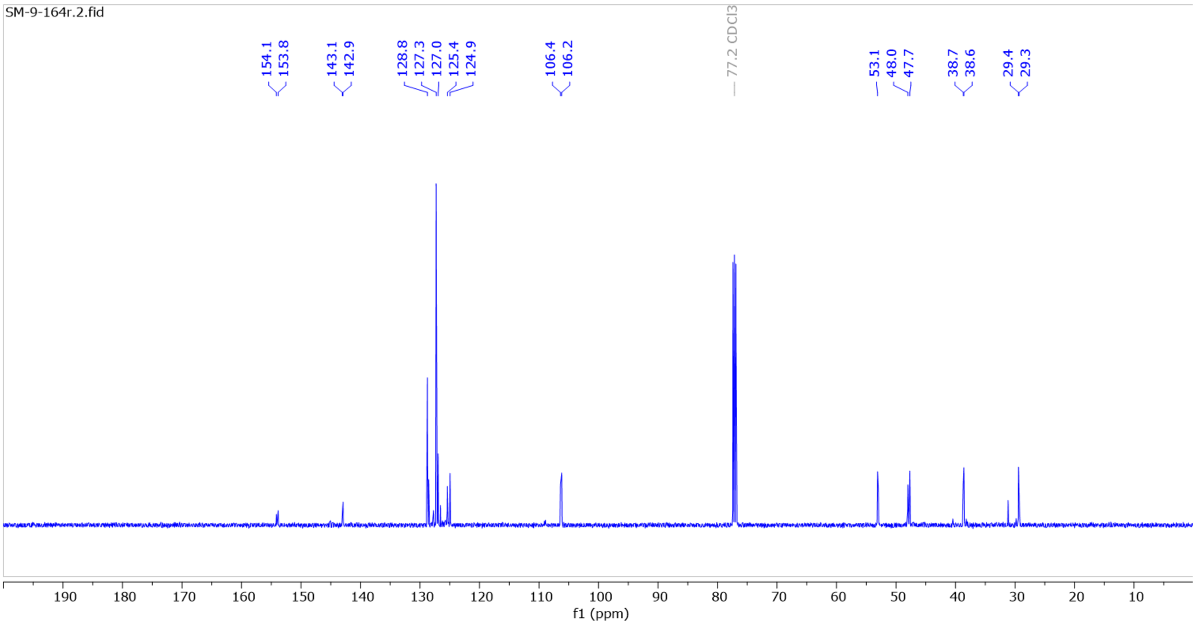

^{13}C NMR (126 MHZ, $CDCl_3$) of **3an**

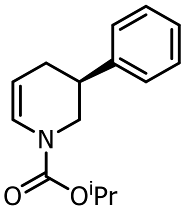

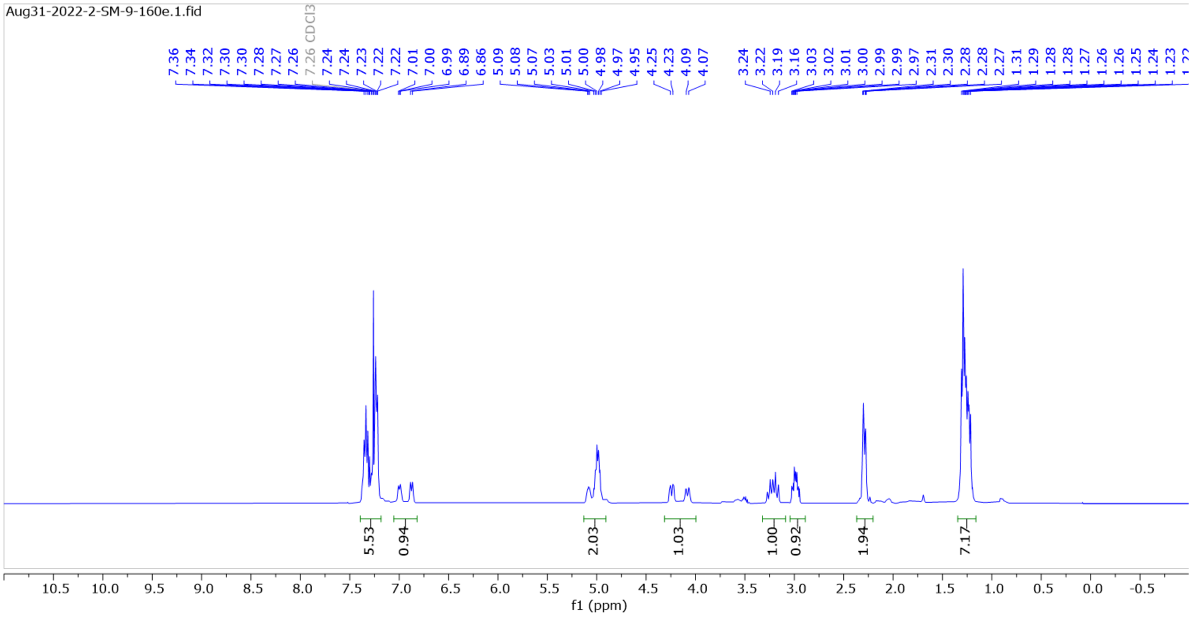

^1H NMR (400 MHZ, $CDCl_3$) of **3ao**

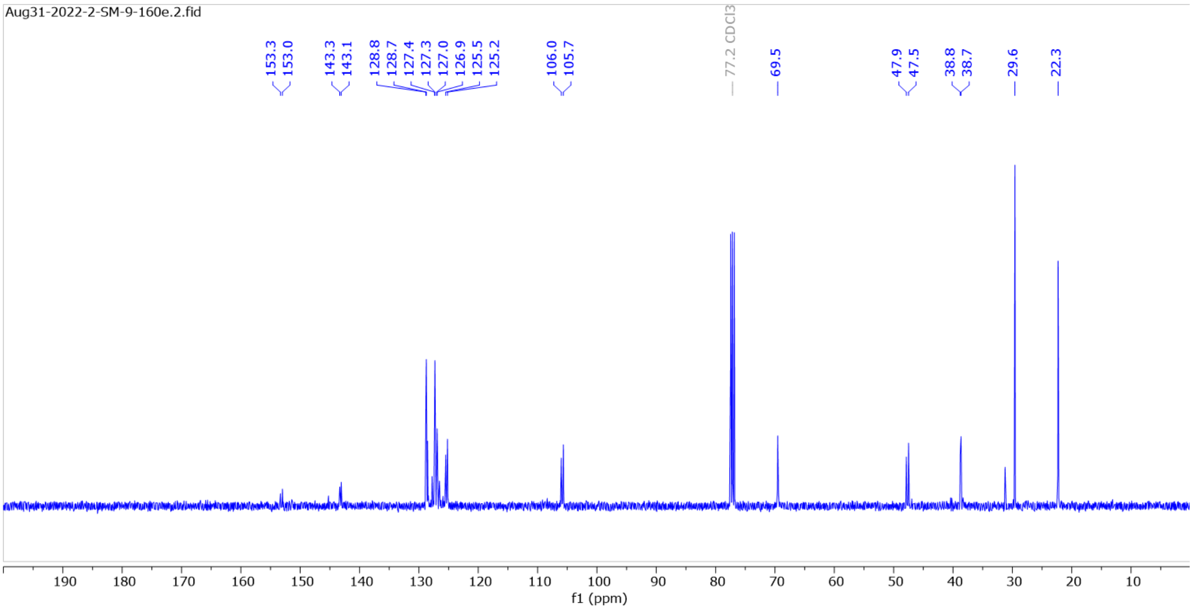

^{13}C NMR (101 MHZ, $CDCl_3$) of **3ao**

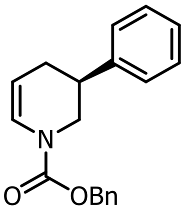

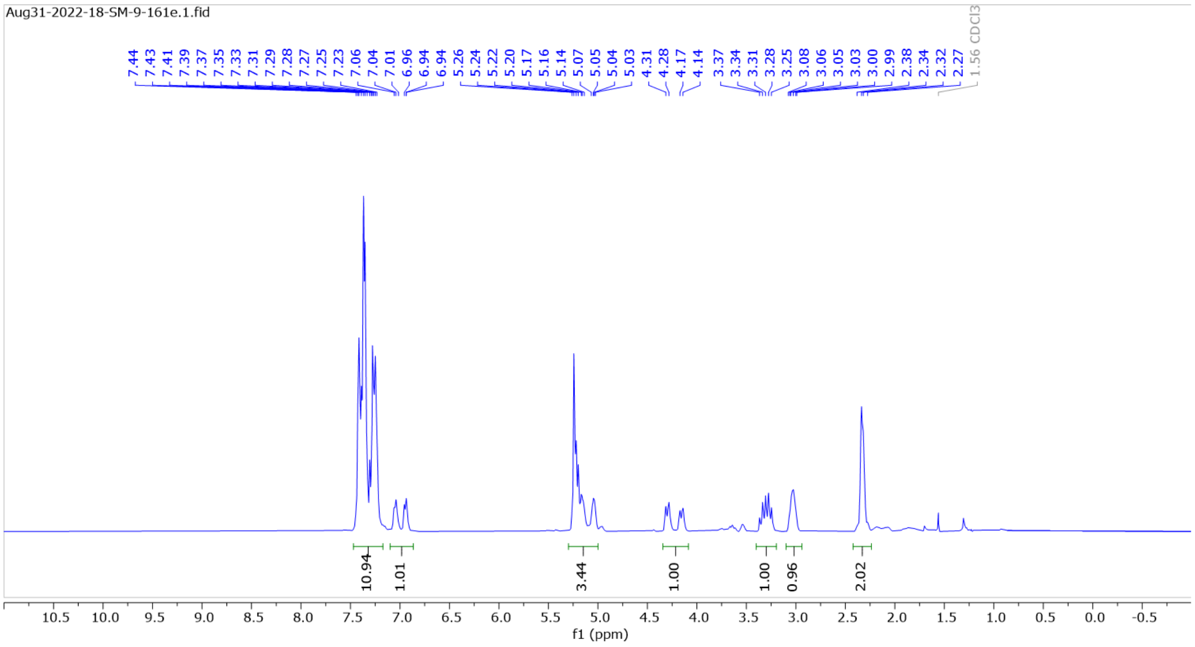

^{1}H NMR (400 MHZ, $CDCl_3$) of **3ap**

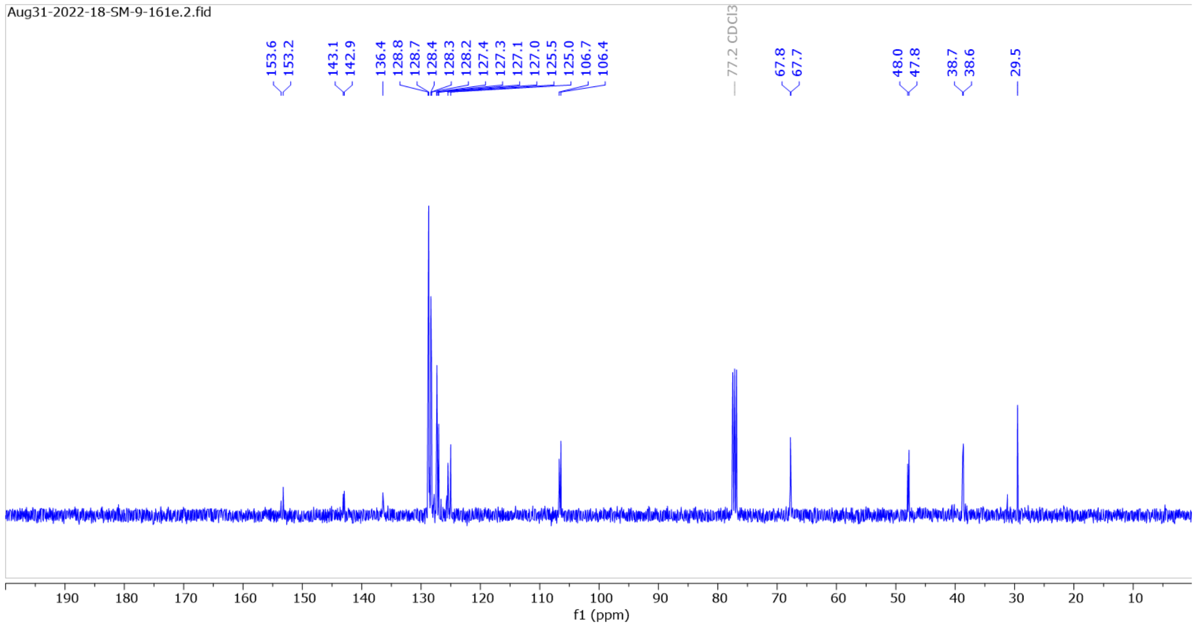

^{13}C NMR (101 MHZ, $CDCl_3$) of **3ap**

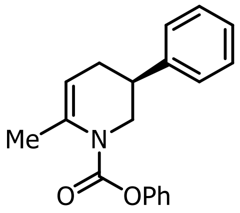

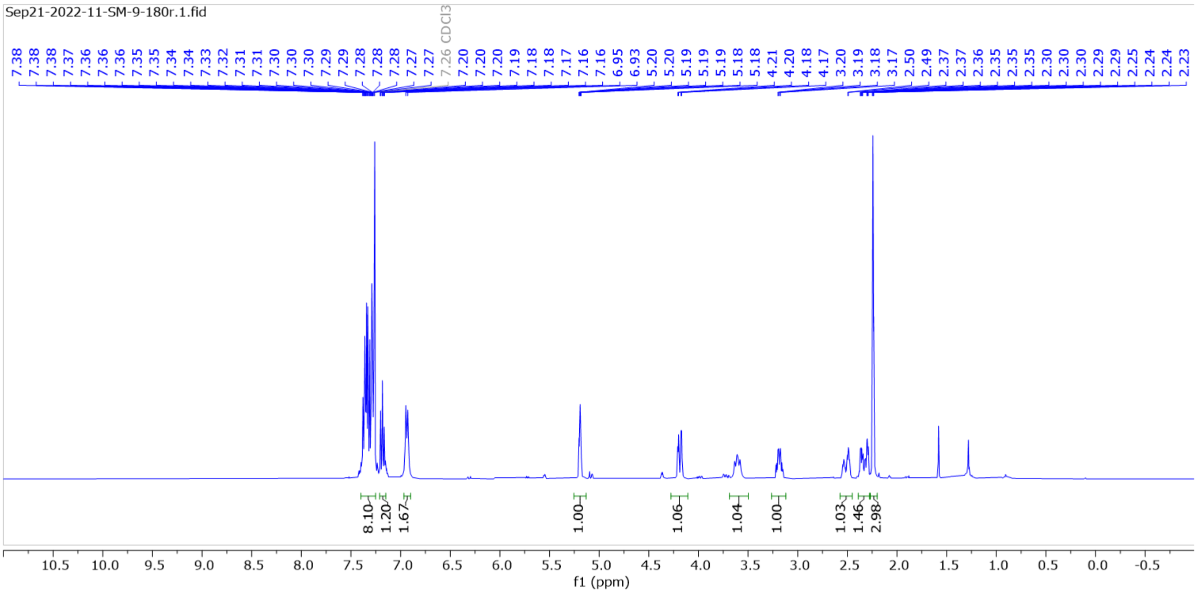

^{1}H NMR (400 MHZ, $CDCl_3$) of **3aq**

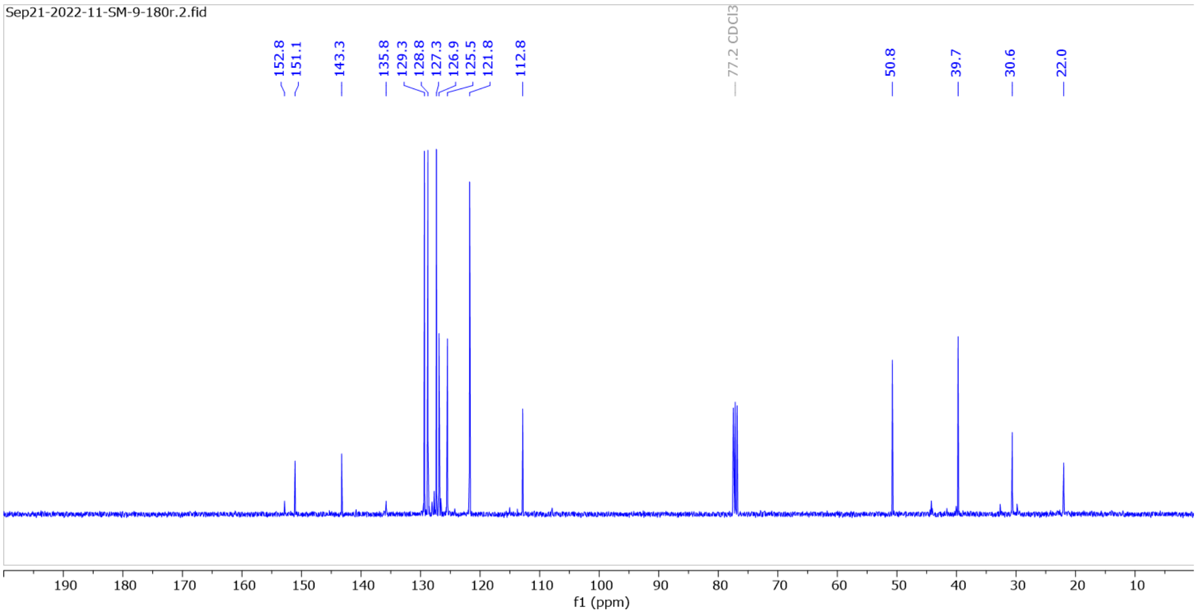

^{13}C NMR (101 MHZ, $CDCl_3$) of **3aq**

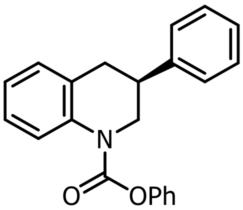

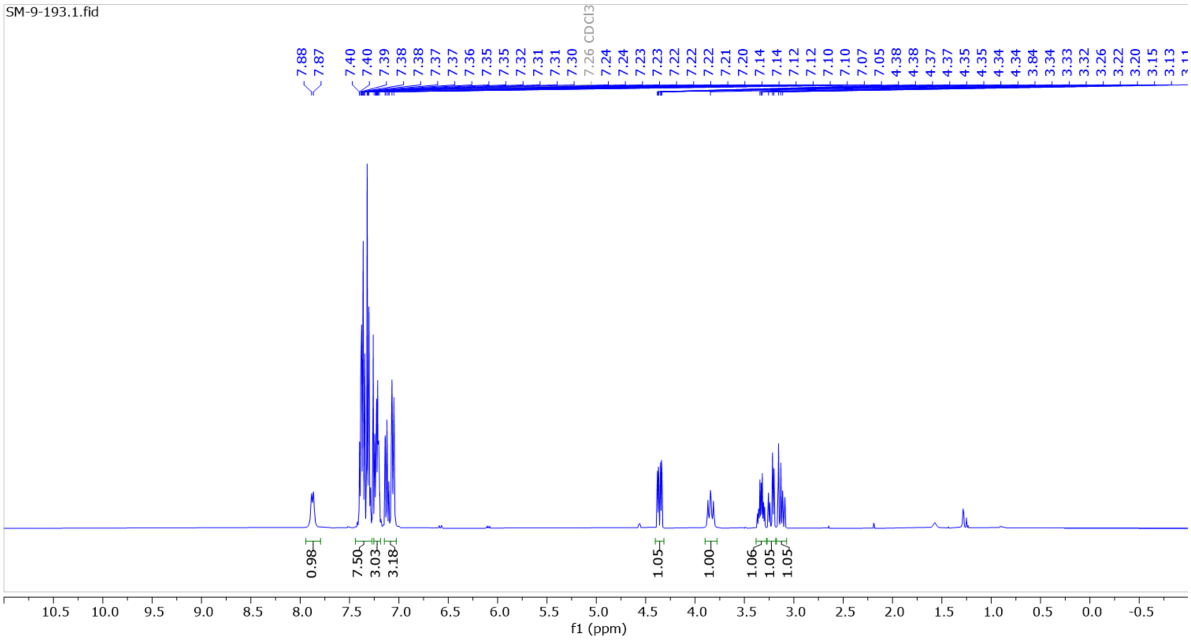

^{1}H NMR (400 MHZ, $CDCl_3$) of **3as**

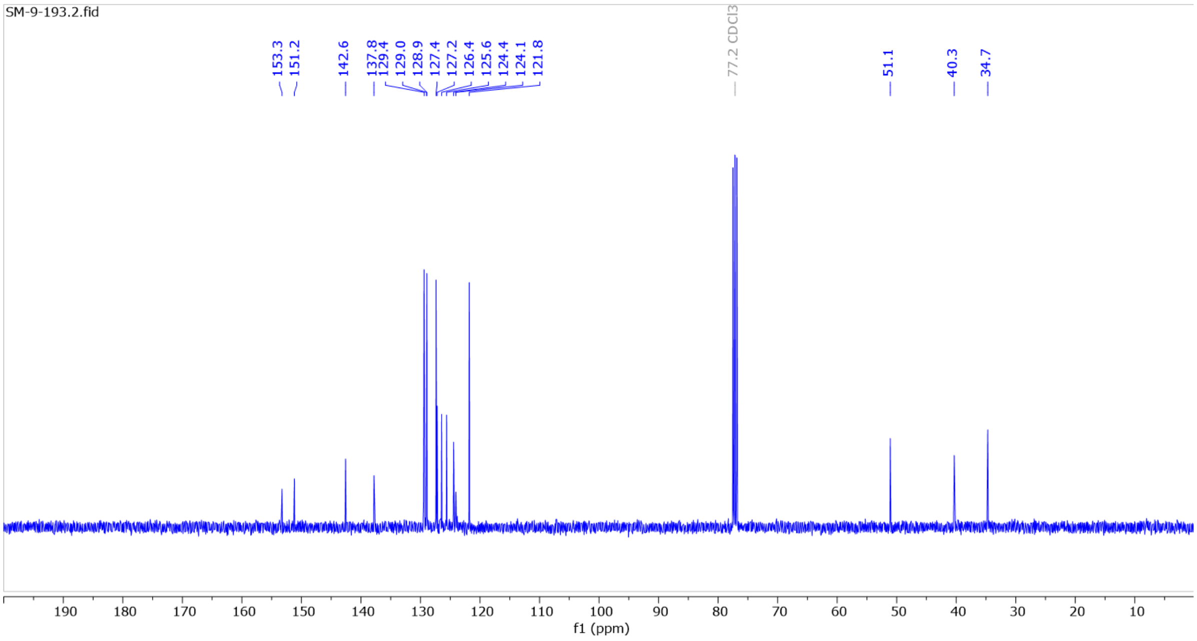

^{13}C NMR (101 MHZ, $CDCl_3$) of **3as**

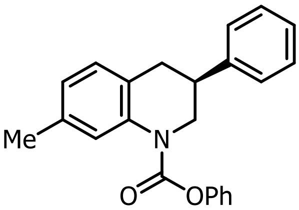

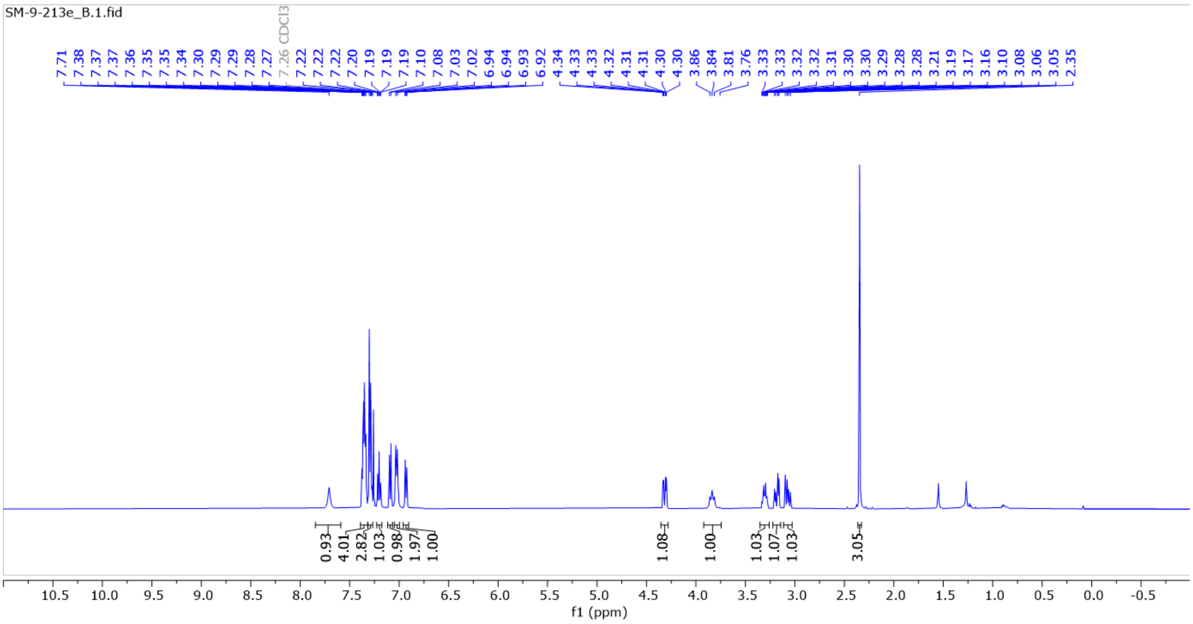

^{1}H NMR (500 MHZ, $CDCl_3$) of **3at**

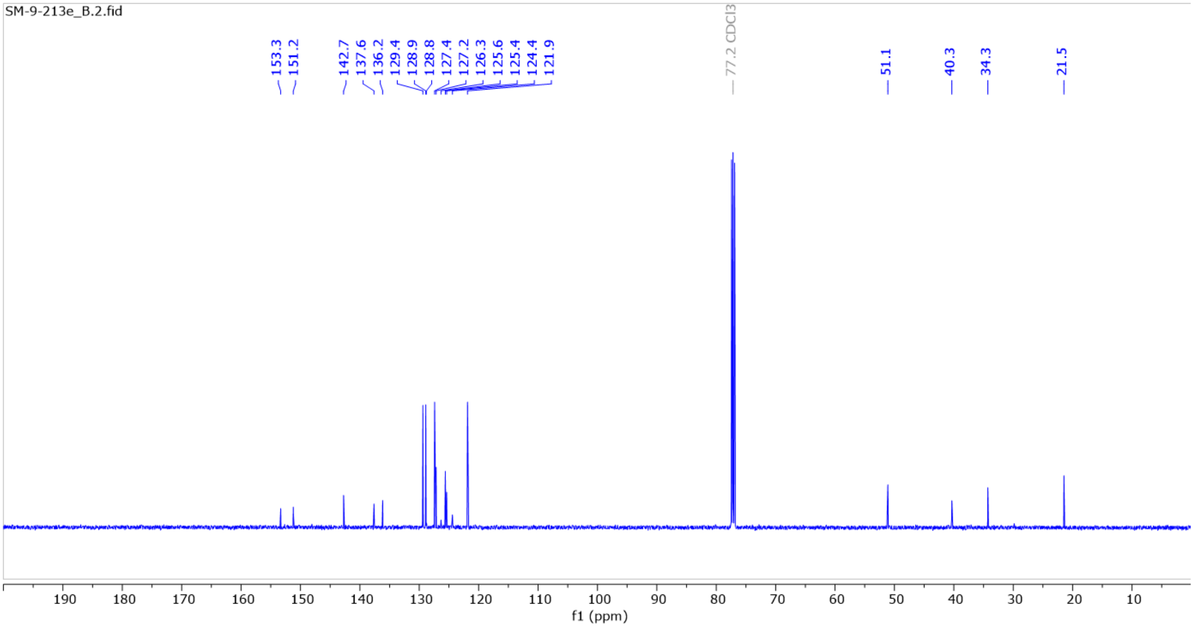

^{13}C NMR (126 MHZ, $CDCl_3$) of **3at**

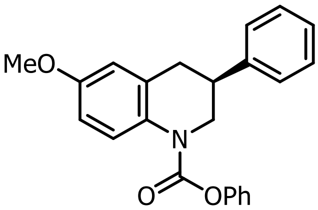

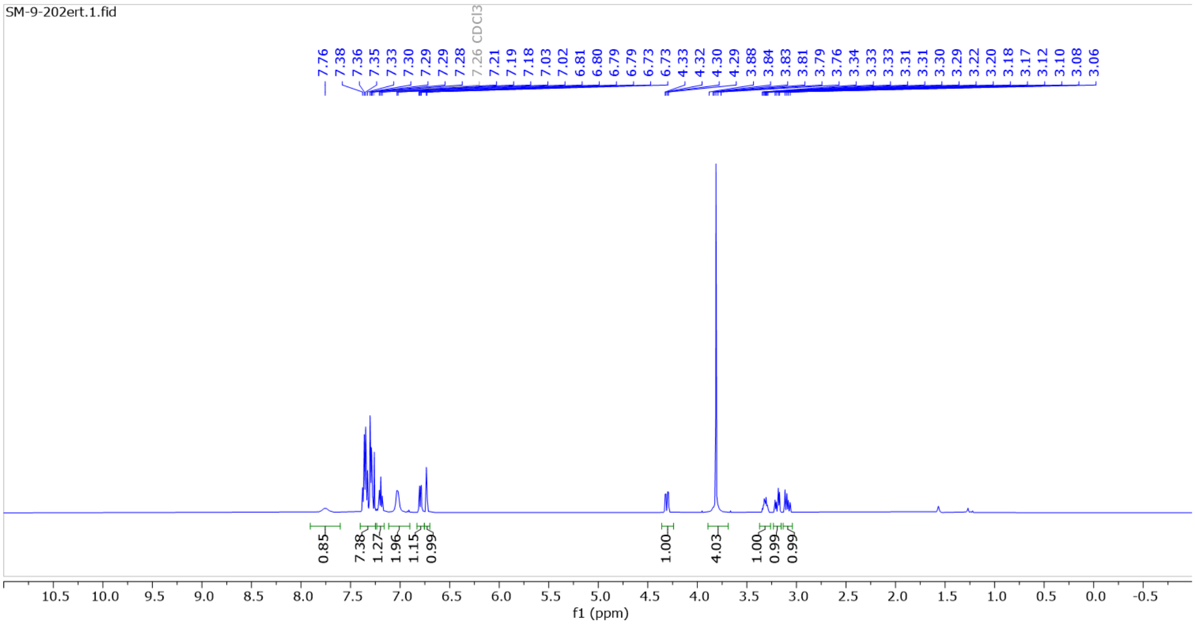

^{1}H NMR (500 MHZ, $CDCl_3$) of **3au**

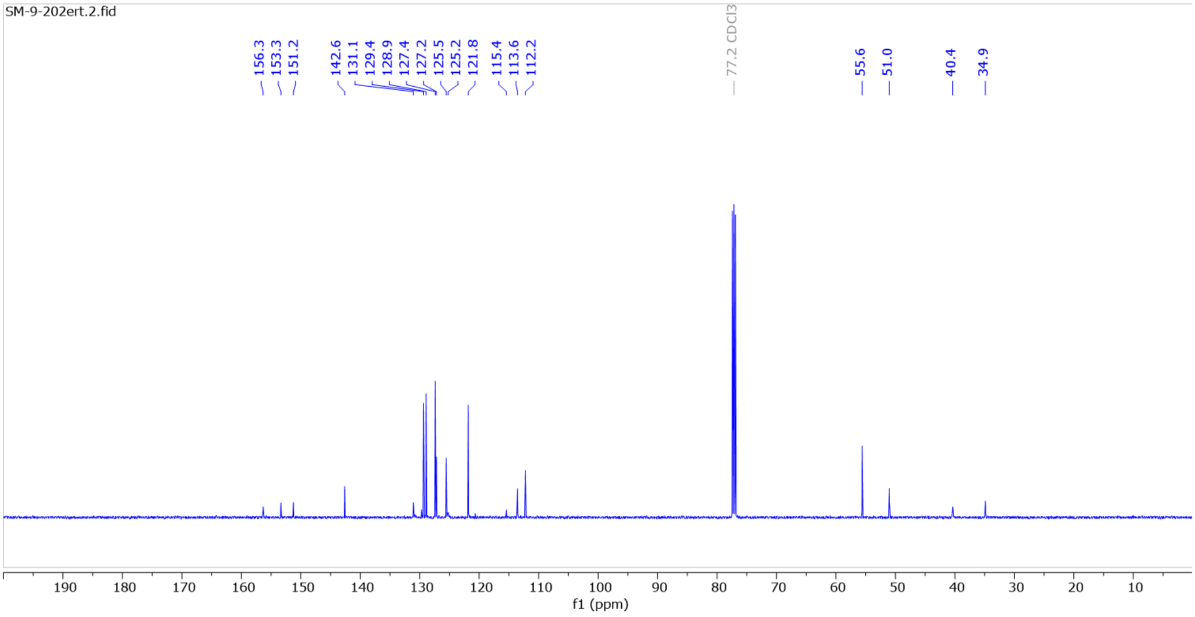

^{13}C NMR (126 MHZ, $CDCl_3$) of **3au**

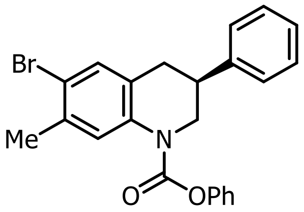

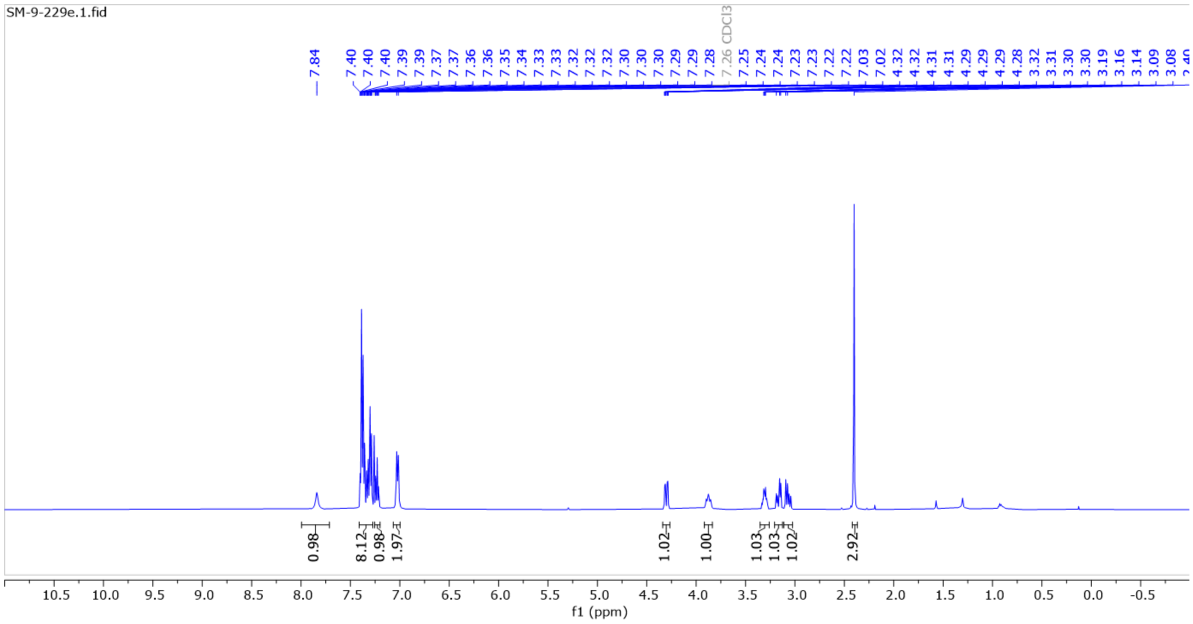

^{1}H NMR (500 MHZ, $CDCl_3$) of **3av**

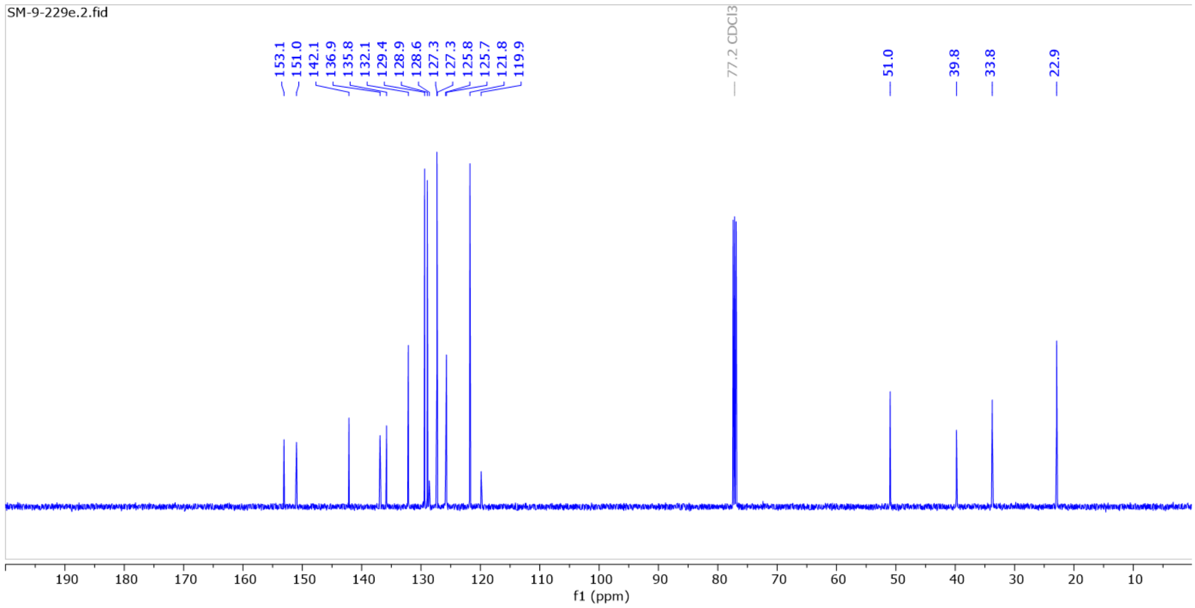

^{13}C NMR (126 MHZ, $CDCl_3$) of **3av**

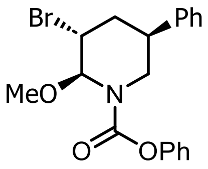

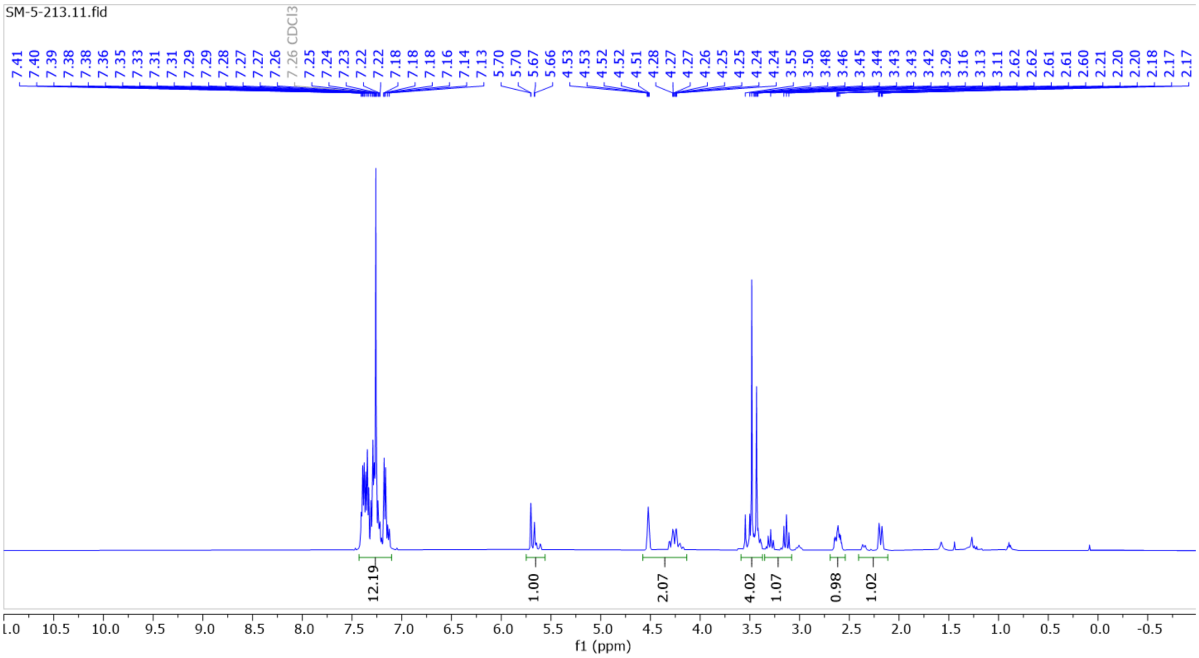

^{1}H NMR (500 MHZ, CDCl$_3$) of **4**

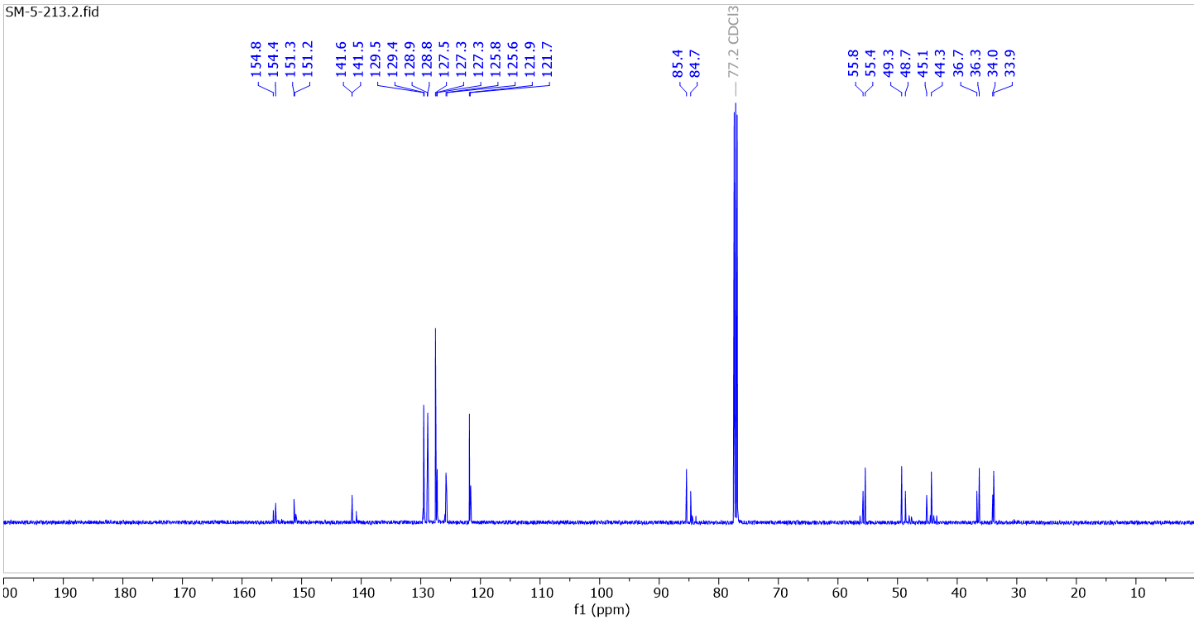

^{13}C NMR (126 MHZ, CDCl$_3$) of **4**

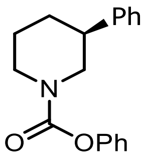

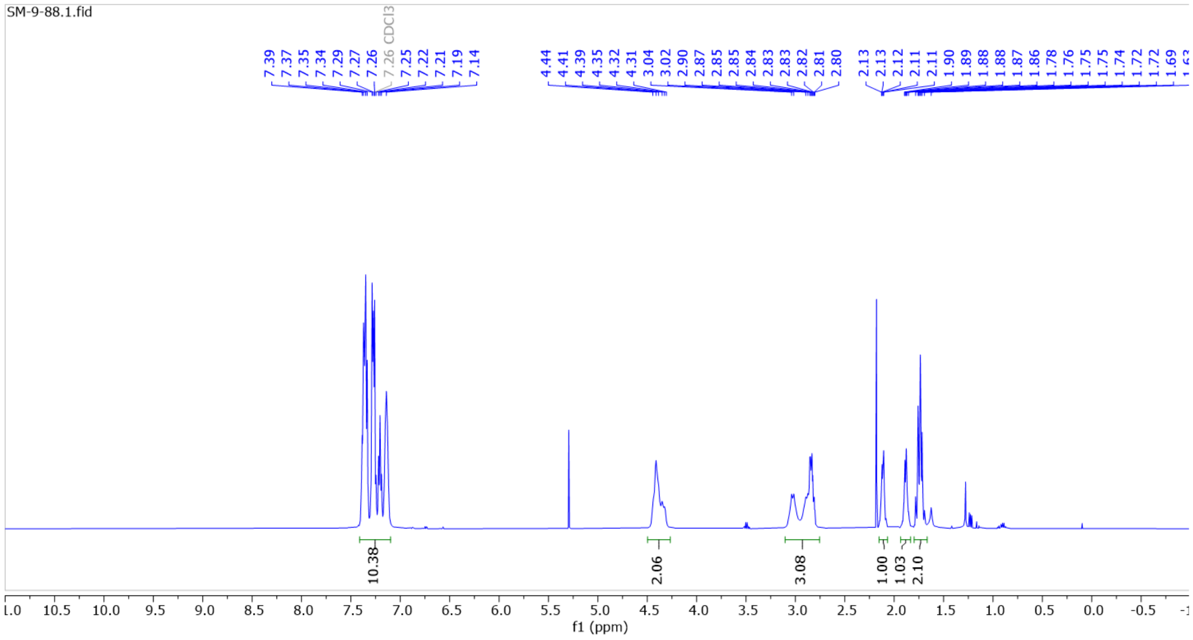

^{1}H NMR (400 MHZ, $CDCl_3$) of **S13**

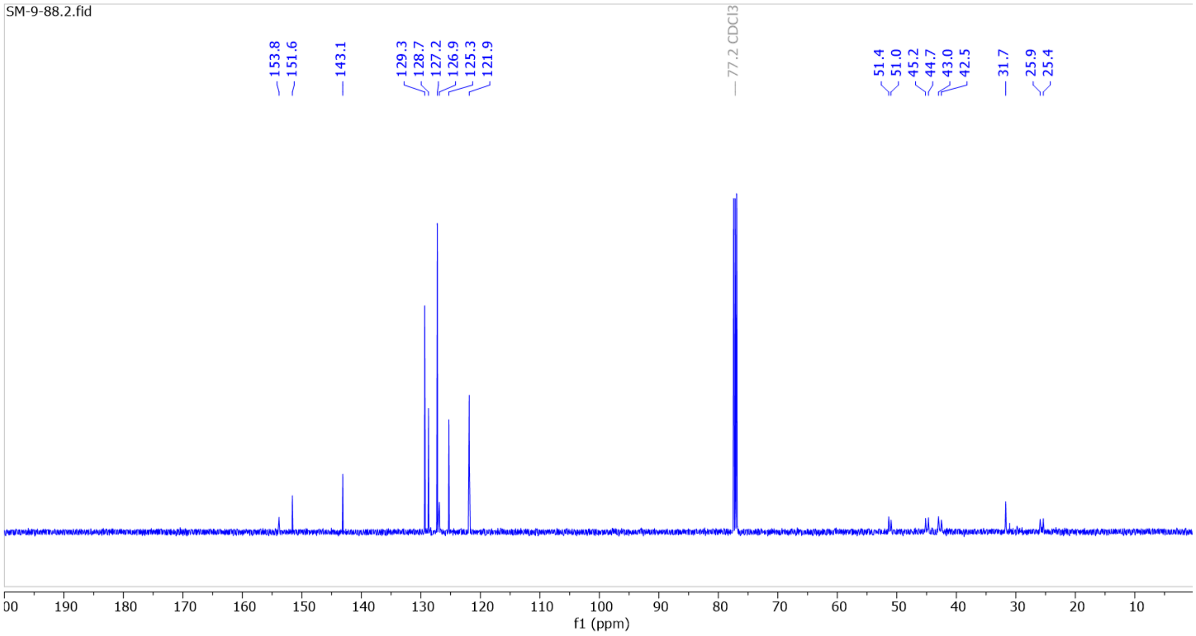

^{13}C NMR (101 MHZ, $CDCl_3$) of **S13**

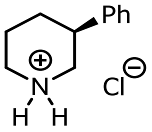

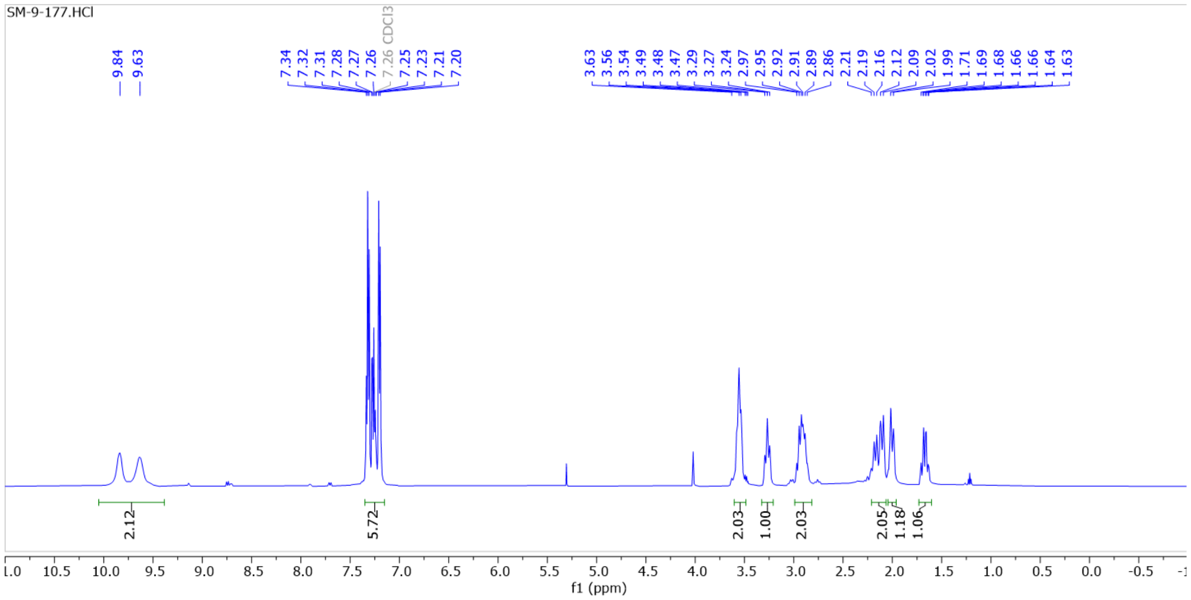

^{1}H NMR (400 MHZ, $CDCl_3$) of **5**

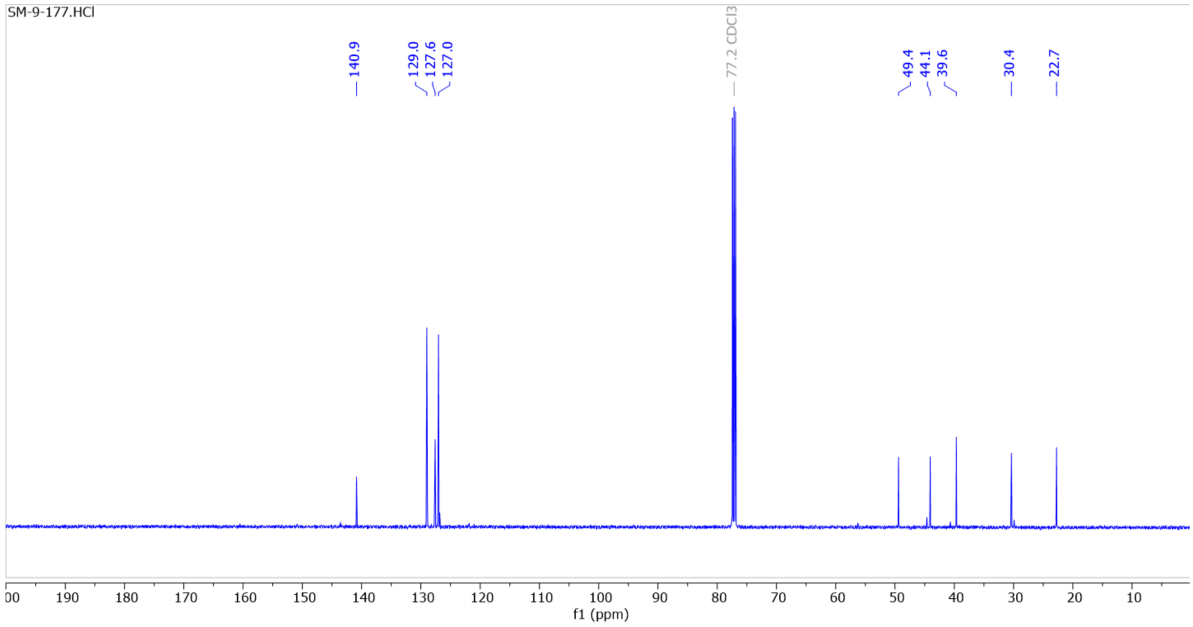

^{13}C NMR (101 MHZ, $CDCl_3$) of **5**

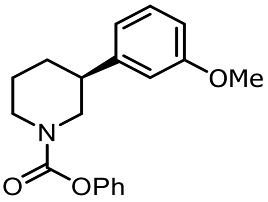

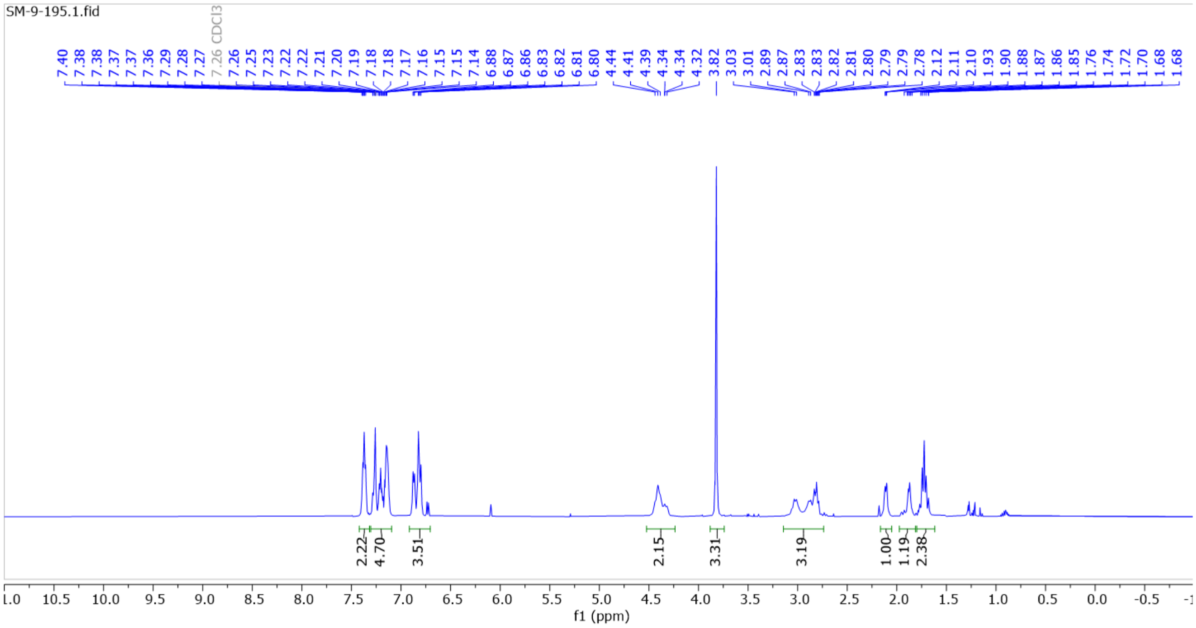

^{1}H NMR (400 MHZ, $CDCl_3$) of **S14**

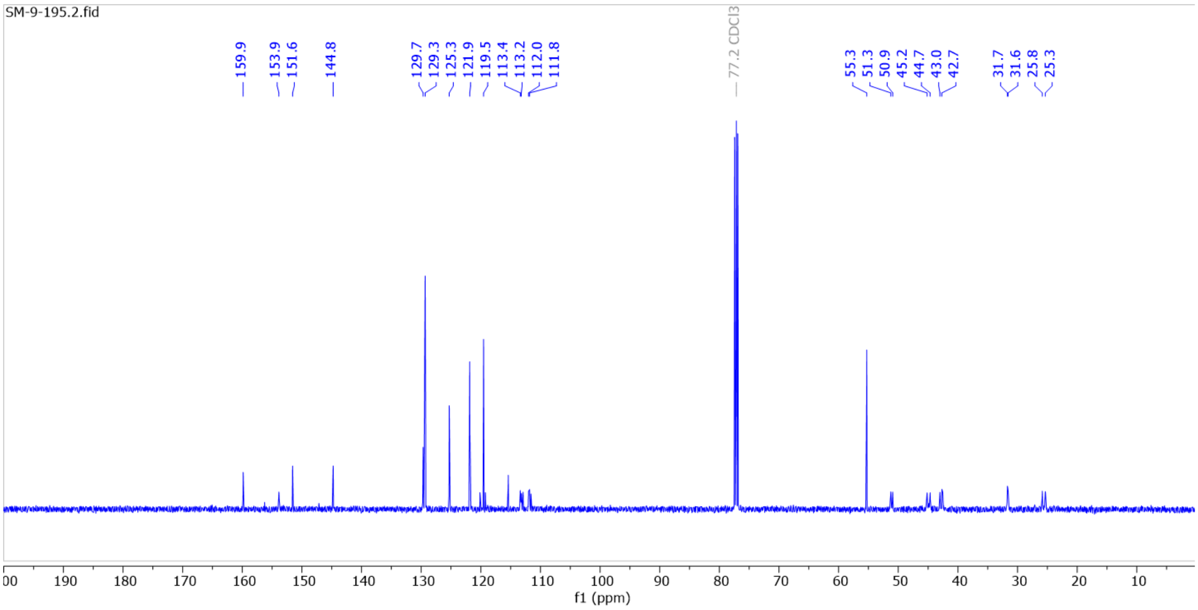

^{13}C NMR (101 MHZ, $CDCl_3$) of **S14**

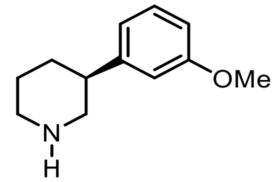

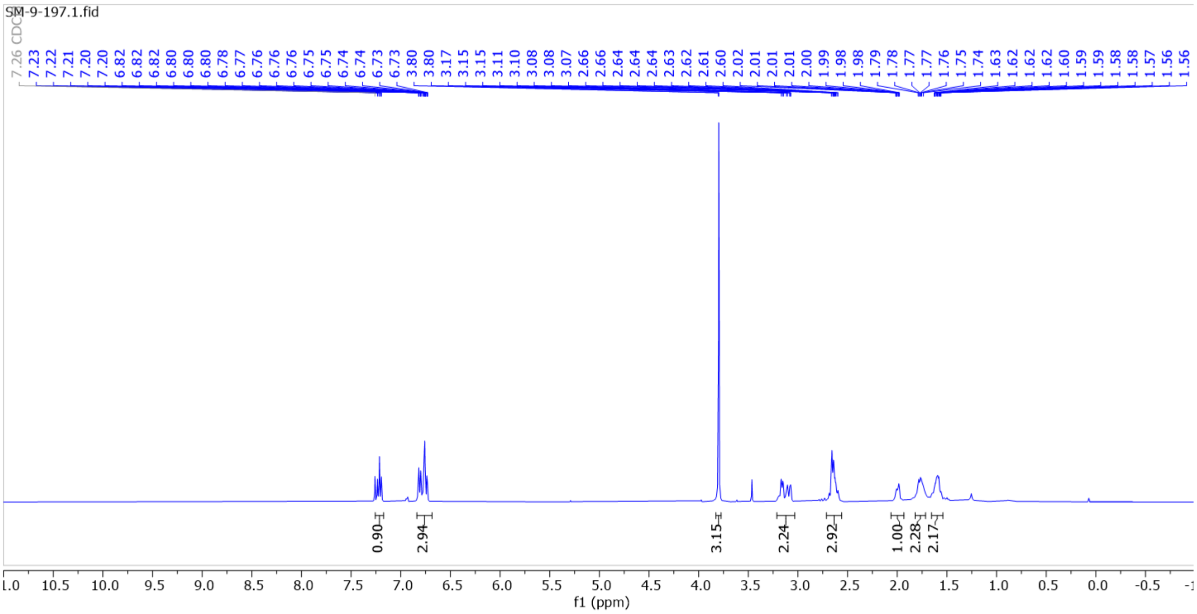

^{1}H NMR (400 MHZ, $CDCl_3$) of **6**

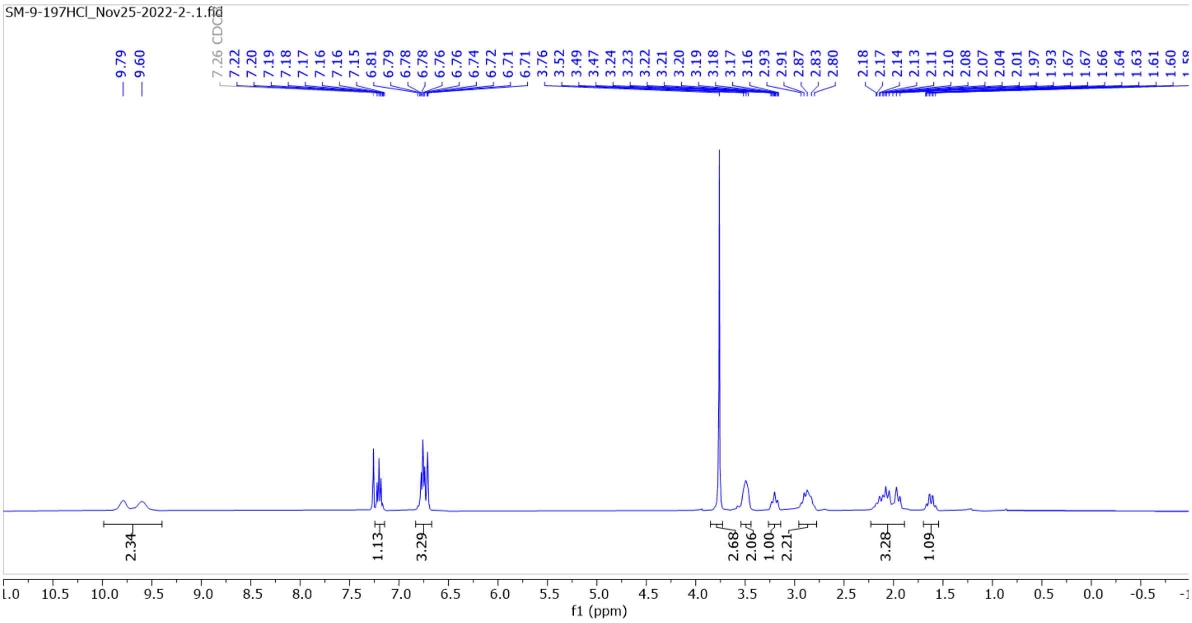

^{1}H NMR (400 MHZ, $CDCl_3$) of **6.HCl**

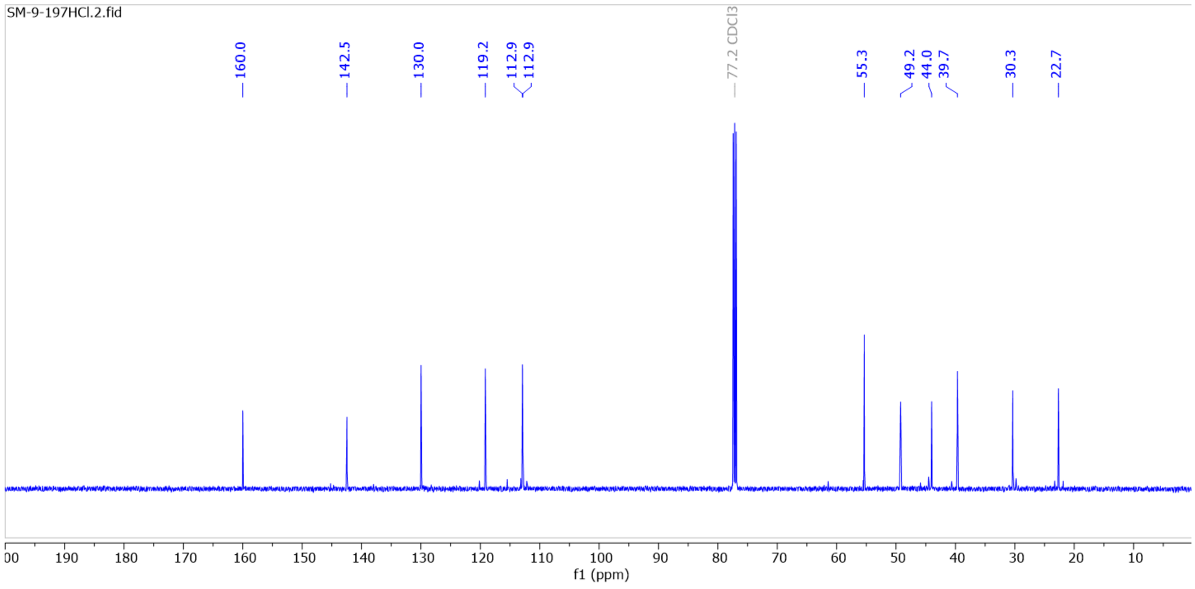

^{13}C NMR (126 MHZ, $CDCl_3$) of **6.HCl**

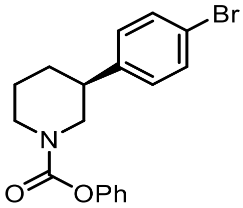

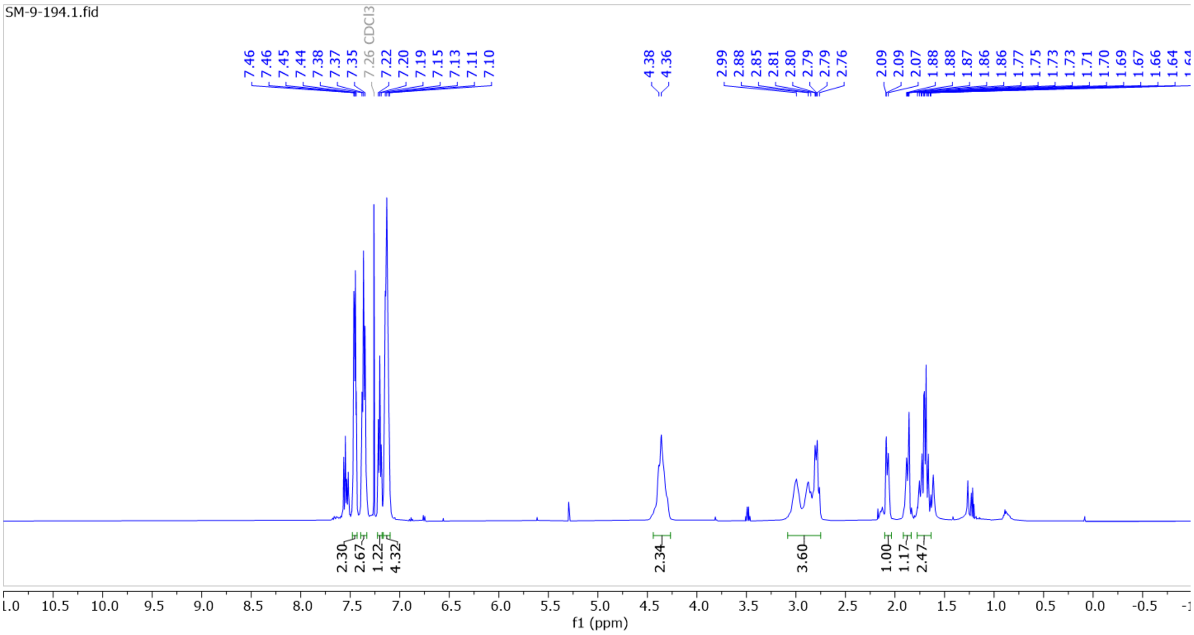

^{1}H NMR (500 MHZ, $CDCl_3$) of **S15**

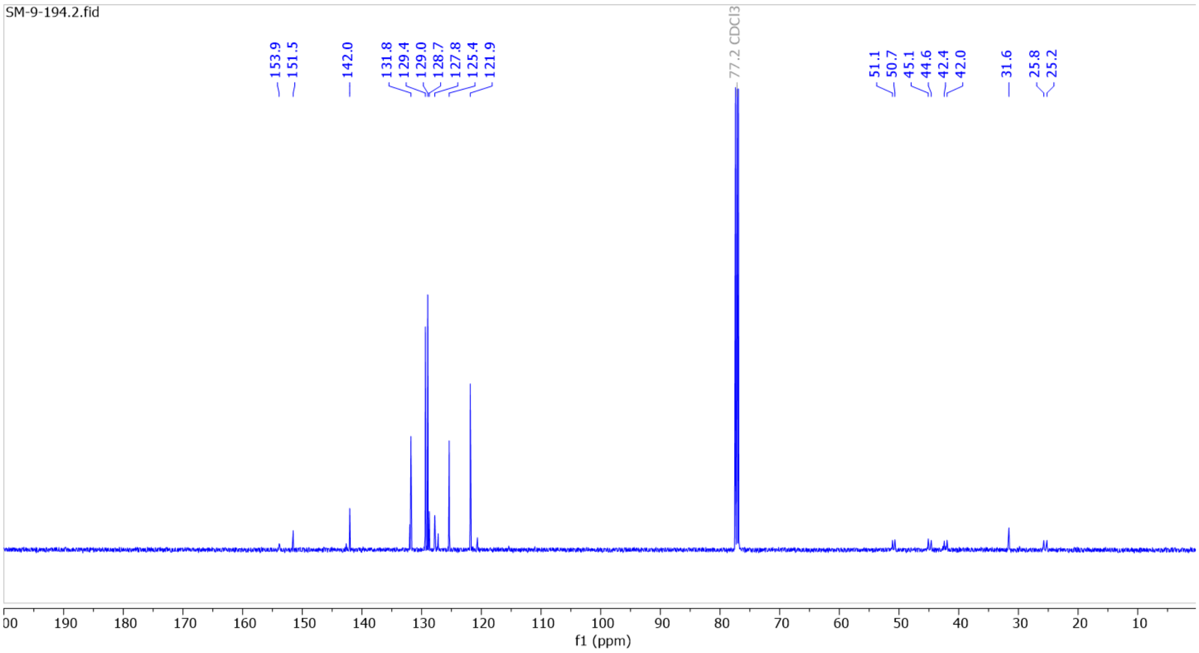

^{13}C NMR (126 MHZ, $CDCl_3$) of **S15**

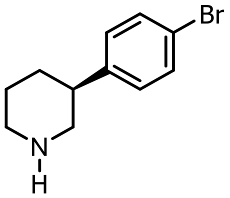

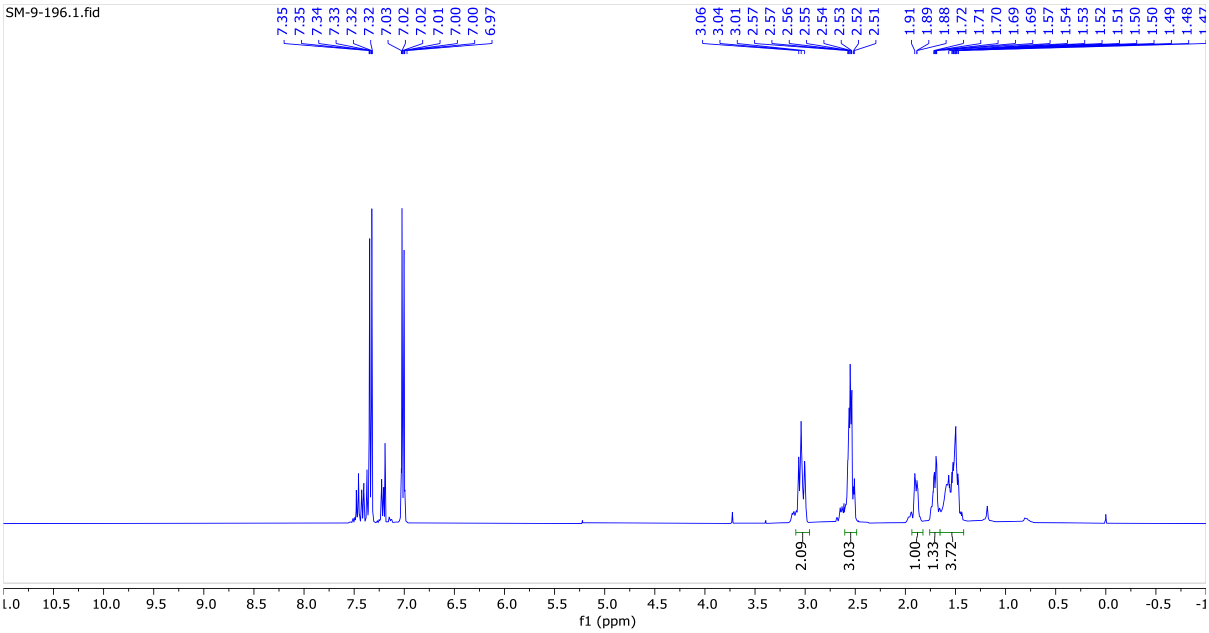

^{1}H NMR (400 MHZ, $CDCl_3$) of **7**

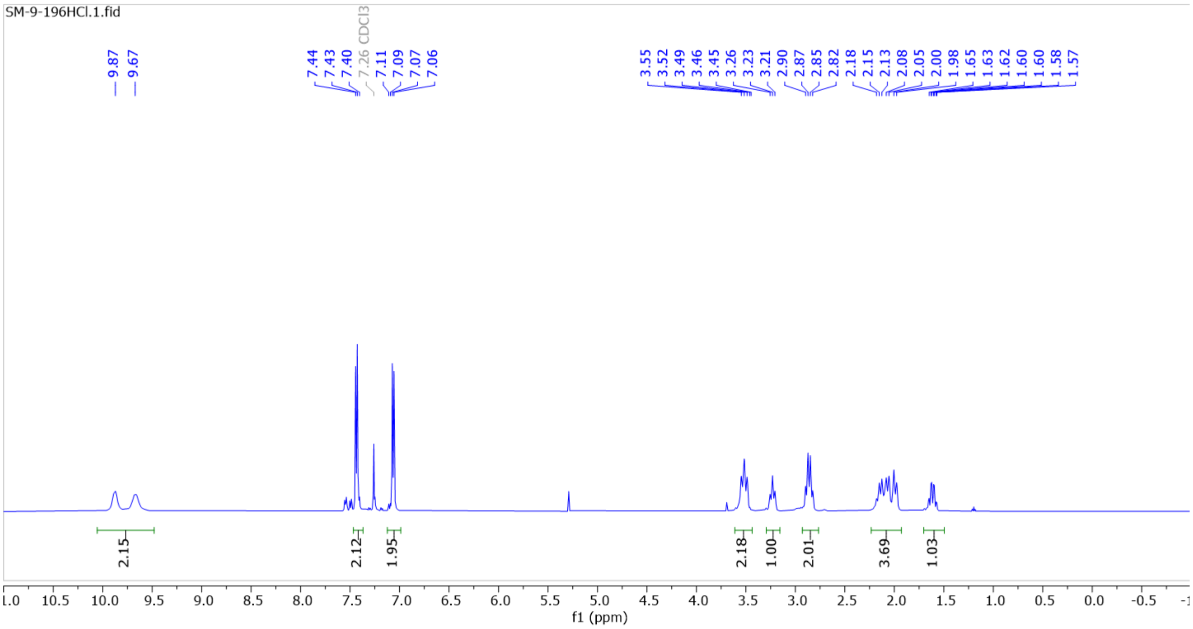

^{1}H NMR (500 MHZ, $CDCl_3$) of **7.HCl**

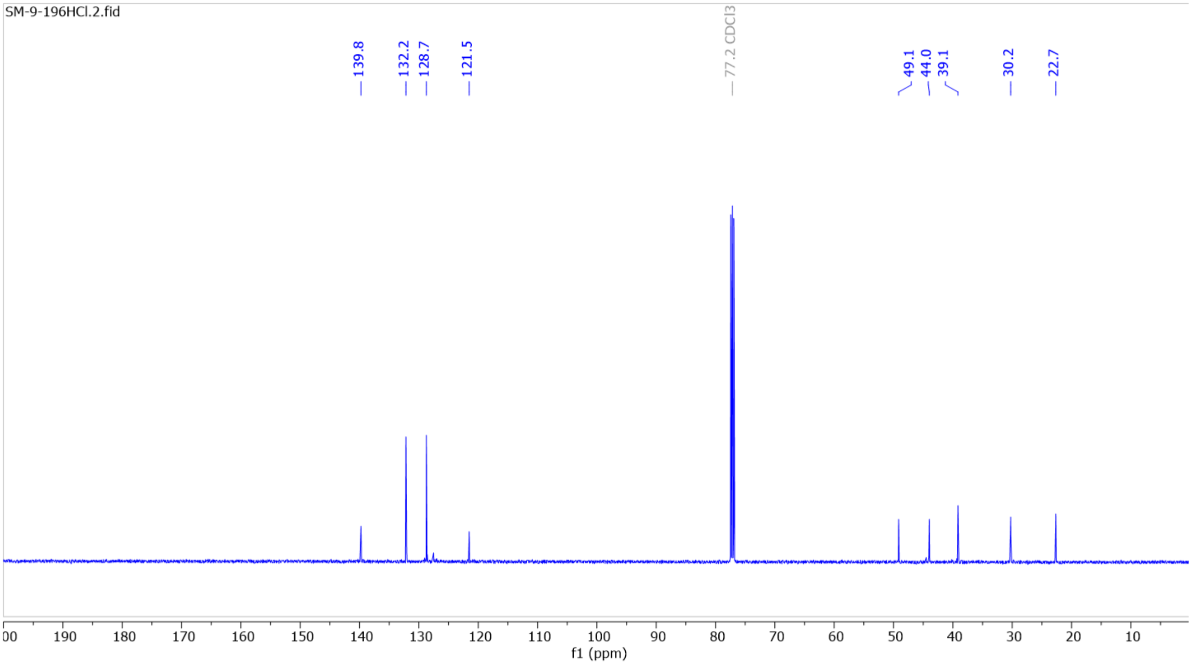

^{13}C NMR (126 MHZ, $CDCl_3$) of **7.HCl**
